# Design, Synthesis, and Pharmacological Characterization of a Potent Soluble Epoxide Hydrolase Inhibitor for the Treatment of Acute Pancreatitis

**DOI:** 10.1021/acs.jmedchem.3c00831

**Published:** 2023-06-19

**Authors:** Simona Musella, Danilo D’Avino, Lukas Klaus Peltner, Veronica Di Sarno, Ida Cerqua, Fabrizio Merciai, Vincenzo Vestuto, Tania Ciaglia, Gerardina Smaldone, Francesca Di Matteo, Simone Di Micco, Valeria Napolitano, Giuseppe Bifulco, Giacomo Pepe, Eduardo Maria Sommella, Manuela Giovanna Basilicata, Giovanna Aquino, Isabel M. Gomez-Monterrey, Pietro Campiglia, Carmine Ostacolo, Fiorentina Roviezzo, Oliver Werz, Antonietta Rossi, Alessia Bertamino

**Affiliations:** † Department of Pharmacy, University Federico II of Naples, Via D. Montesano 49, 80131 Naples, Italy; ‡ Department of Pharmacy, University of Salerno, Via G. Paolo II 132, 84084 Fisciano, Salerno, Italy; § Department of Pharmaceutical/Medicinal Chemistry, Institute of Pharmacy, Friedrich-Schiller-University, Philosophenweg 14, D-07743 Jena, Germany; ∥ European Biomedical Research Institute (EBRIS), Via S. De Renzi 50, 84125 Salerno, Italy; $ PhD Program in Drug Discovery and Development, University of Salerno, 84084 Fisciano, SA, Italy

## Abstract

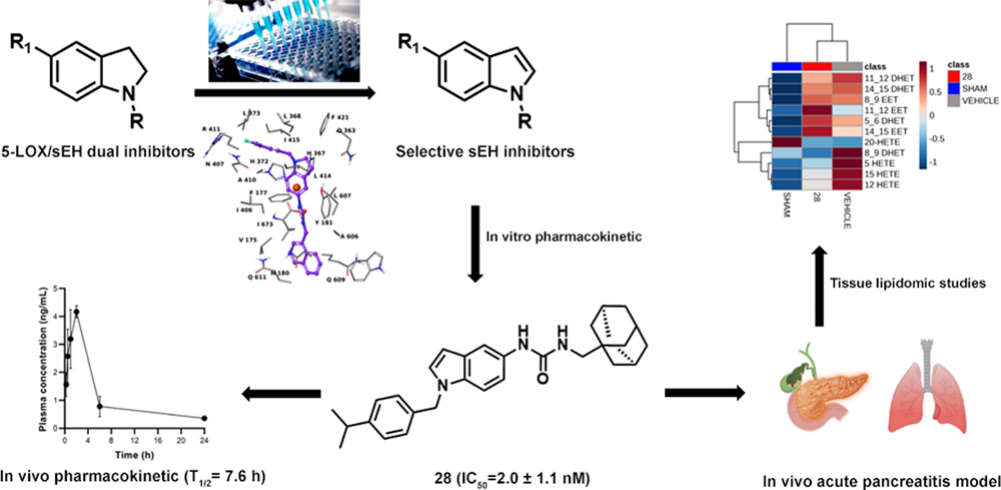

Acute pancreatitis (AP) is a potentially life-threatening illness characterized by an exacerbated inflammatory response with limited options for pharmacological treatment. Here, we describe the rational development of a library of soluble epoxide hydrolase (sEH) inhibitors for the treatment of AP. Synthesized compounds were screened *in vitro* for their sEH inhibitory potency and selectivity, and the results were rationalized by means of molecular modeling studies. The most potent compounds were studied *in vitro* for their pharmacokinetic profile, where compound **28** emerged as a promising lead. In fact, compound **28** demonstrated a remarkable *in vivo* efficacy in reducing the inflammatory damage in cerulein-induced AP in mice. Targeted metabololipidomic analysis further substantiated sEH inhibition as a molecular mechanism of the compound underlying anti-AP activity *in vivo*. Finally, pharmacokinetic assessment demonstrated a suitable profile of **28***in vivo*. Collectively, compound **28** displays strong effectiveness as sEH inhibitor with potential for pharmacological AP treatment.

## Introduction

**Figure 1:**
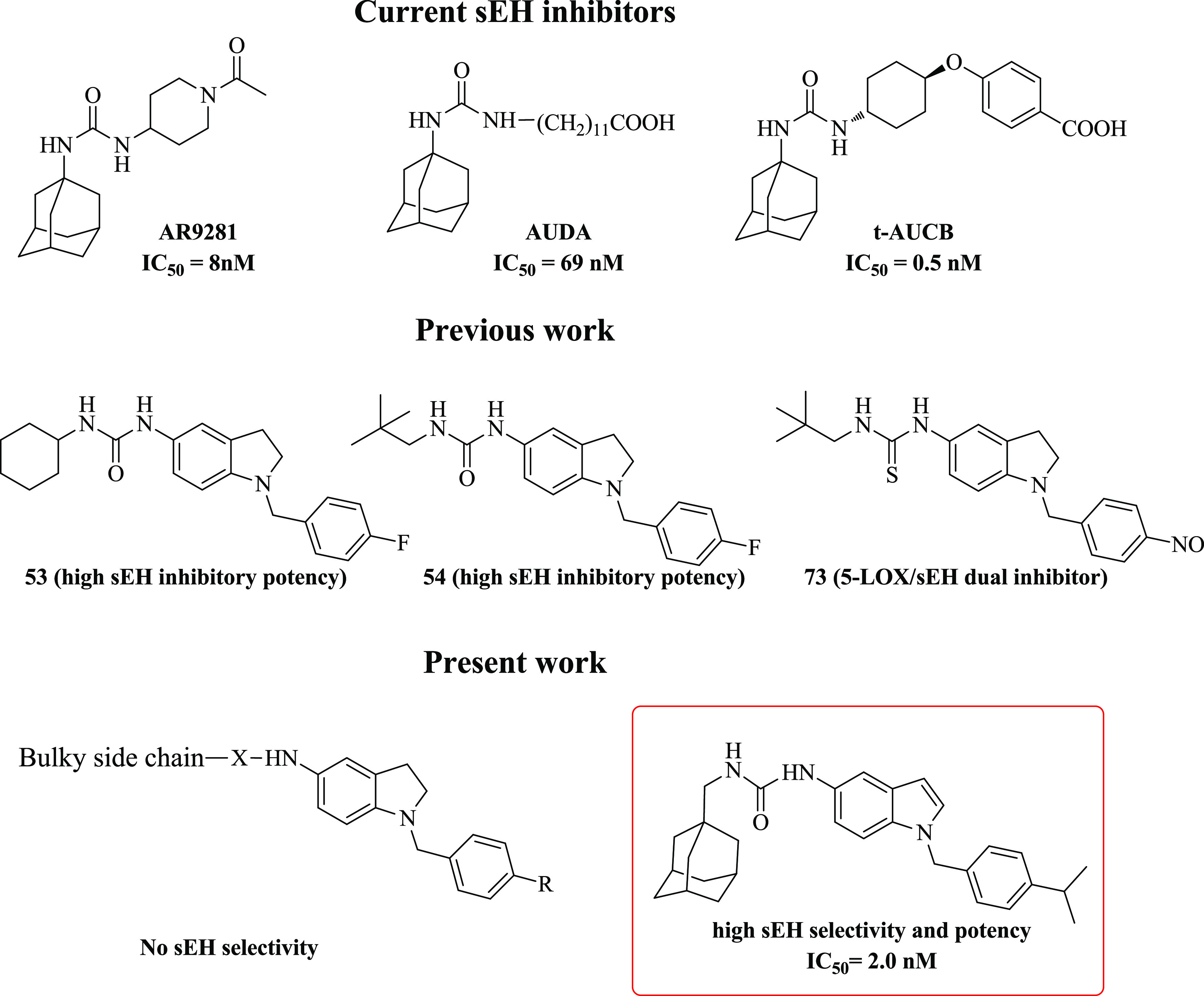
Rational design for sEH selective inhibitors.^20−23^

Inflammatory cascade represents a well-characterized pathway, composed of both pro- and anti-inflammatory mediators, whose balance establishes the course of the inflammatory response. Cyclooxygenases (COX), lipoxygenases (LOX), and cytochrome P450 (CYP) are the enzymatic triad deputed to arachidonic acid (AA) metabolism to bioactive lipid mediators (LMs). COX and 5-LOX initialize the generation of the pro-inflammatory prostaglandins (PGs) and leukotrienes (LTs), respectively, while CYP enzymes produce the pro-resolving cis-epoxyeicosatrienoic acids (EETs). EETs proved to exhibit many important functions, positively regulating cellular proliferation, inflammation, hemostasis, and other intracellular signaling pathways.^1^ The EET-mediated pathways are counter-balanced by soluble epoxide hydrolase (sEH) that converts EETs in their corresponding vic-dihydroxyeicosatrienoic acids (DHETs), devoid of the above-mentioned beneficial activities. Thus, sEH inhibition represents a promising strategy as alternative anti-inflammatory approach. In the last years, sEH inhibitors gained huge attention in several fields, such as inflammatory-based disorders,^2−4^ cancer,^5^ heart failure,^6^ Alzheimer’s disease and other neurodegenerative disorders,^7,8^ liver diseases,^9^ and pain.^10^ Moreover, sEH modulation has been recently highlighted as a promising strategy in the treatment of acute pancreatitis (AP).^11,12^ AP is a multifactorial pathology highly impacting worldwide, characterized by increased incidence, especially in Western countries.^13,14^ The severity grade of AP is extremely variable, encompassing mild symptomatology, treatable by pharmacological therapy, and more serious, potentially fatal, cases with a 20% incidence.^15^ It has been reported that sEH inhibitors, beyond their direct anti-inflammatory activity,^16^ are able to exert a direct regulation over ER stress, thus potentially playing a pivotal role in the treatment of AP.^17^ sEH is a cytosolic and peroxisomal bifunctional homodimeric enzyme, with both epoxide hydrolase and phosphatase activity.^18^ In the last decades, different sEH inhibitors have been disclosed and many of them are characterized by an adamantyl urea moiety directly bound to a flexible aliphatic chain (Figure 1).^19^

We have recently described the pharmacological characterization of a dual 5-LOX/sEH inhibitor (compound **73**, Figure 1) showing a remarkable potency in zymosan-induced peritonitis and in ovalbumin-induced asthma murine models.^20^

These preliminary results led us to highlight the structural determinants leading to 5-LOX and sEH inhibition. In our previous paper, beyond the 5-LOX/sEH dual inhibitor **73** (Figure 1), we also identified compounds **53** and **54** (Figure 1) showing similar activities. The two compounds bear an aliphatic bulky urea side chain directly bound to the indoline scaffold exhibiting potent sEH inhibition (IC_50s_ = 0.061 ± 0.003 and 0.10 ± 0.01 μM, for compounds **53** and **54**, respectively) despite maintaining a remarkable inhibitory activity on isolated 5-LOX (IC_50s_ = 0.28 ± 0.02 and 0.18 ± 0.05 μM, for compounds **53** and **54**, respectively), coherently with our molecular modeling studies. To further explore the structural determinants regulating sEH selective inhibition, in the present paper, a second library of compounds has been designed, synthesized, and pharmacologically evaluated. Initially, our investigation was focused on the bulky side chain and the linker with the indoline core. Moreover, *in silico* studies suggested that an increase in rigidity of the indoline scaffold would have strongly modified compounds selectivity in favor of the sEH enzyme, rather than 5-LOX. For these reasons, we replaced the indoline scaffold with indole and carbazole in order to maintain the chemical features of the original nucleus while improving its rigidity. Finally, we investigated the effects exerted by specific substituents at the N-1 position, using different aliphatic and aromatic chains. The results obtained confirmed the original hypothesis (Figure 1). In accordance with previous reports,^21−23^ a bulky group properly linked to a urea group is crucial for sEH inhibition, while increasing the planarity of the central scaffold is crucial for sEH selectivity in this series of compounds, differently from the previously reported sEH inhibitors that are mostly characterized by a high degree of flexibility (Figure 1).

Starting from *in vitro* preliminary data we selected the most potent derivatives (compounds **27**, **28**, **30**, and **36**) to assess their pharmacokinetic properties *in vitro*. This approach led to the identification of compound **28** as the most metabolically stable analogue. Thus, derivative **28** was challenged in an AP murine *in vivo* model. Compound **28** showed to significantly reverse the cerulein-induced injury, also reversing the pancreatitis-associated lung and hepatic injury. The efficacy of **28** in the AP murine model, also relies on its suitable pharmacokinetic properties, as assessed by *in vivo* pharmacokinetic studies. Finally, to further confirm the molecular pathways underlying the *in vivo* efficacy of compound **28**, a targeted lipidomics study was performed investigating the levels of multiple EETs, DHETs, and HETEs in pancreatic homogenates. Collectively, our results further highlight the potential of compound **28** and sEH inhibitors as anti-inflammatory agents for the treatment of AP.

## Results and Discussion

### Chemistry

**Scheme 1:**
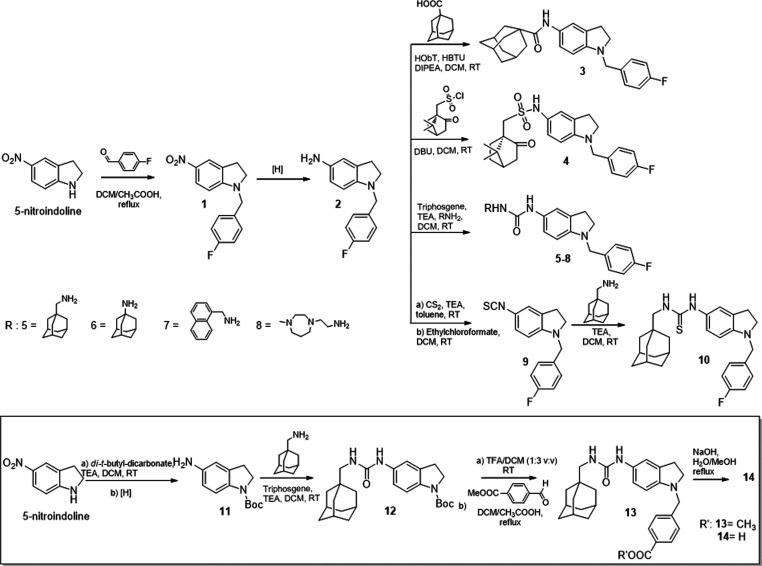
Synthesis of Indoline Derivatives **3**–**8**, **10**, and **14**

Compounds **3–8**, **10**, and **14** were synthesized according to Scheme 1. Starting from 5-nitroindoline, reductive amination with 4-fluorobenzaldehyde afforded intermediate **1** (92% yield), which was reduced by continuous flow hydrogenation giving the aminoindoline **2** in 95% yield.

Intermediate **2** was then used as starting material in four different synthetic pathways. Coupling reaction with 1-adamantanecarboxylic acid, using 1-hydroxybenzotriazole (HOBt), HBTU, and diisopropylethylamine (DIPEA) led to final compound **3** in 61% yield, while the treatment with ((1*S*)-7,7-dimethyl-2-oxobicyclo[2.2.1]heptan-1-yl)methanesulfonyl chloride, in basic medium, provided sulfonamide final derivative **4** (35% yield).

Upon reaction of **2** with triphosgene, triethylamine (TEA), and a proper aliphatic amine, ureido compounds **5–8** were obtained in 38–48% of yields. Otherwise, the amino group of compound **2** was converted into isothiocyanate, by reaction with carbon disulfide in toluene and TEA, generating intermediate **9**, that was treated with 1-(adamantan-1-yl)methanamine to give final compound **10** (38% yield).

The carboxylic derivative **14** was synthesized starting from 5-nitroindoline that was converted to its N-1 Boc-protected analogue and then reduced to its corresponding 5-amino derivative **11**, which upon treatment with triphogene, TEA, and 1-(adamantan-1-yl)methanamine leads to the ureido compound **12** in 45% yield. Intermediate **13** was obtained, in 55% yield, after N-1 Boc deprotection and reductive amination with methyl 4-formylbenzoate. The ester group hydrolysis using NaOH gave the final compound **14** in 67% yield.

**Scheme 2:**
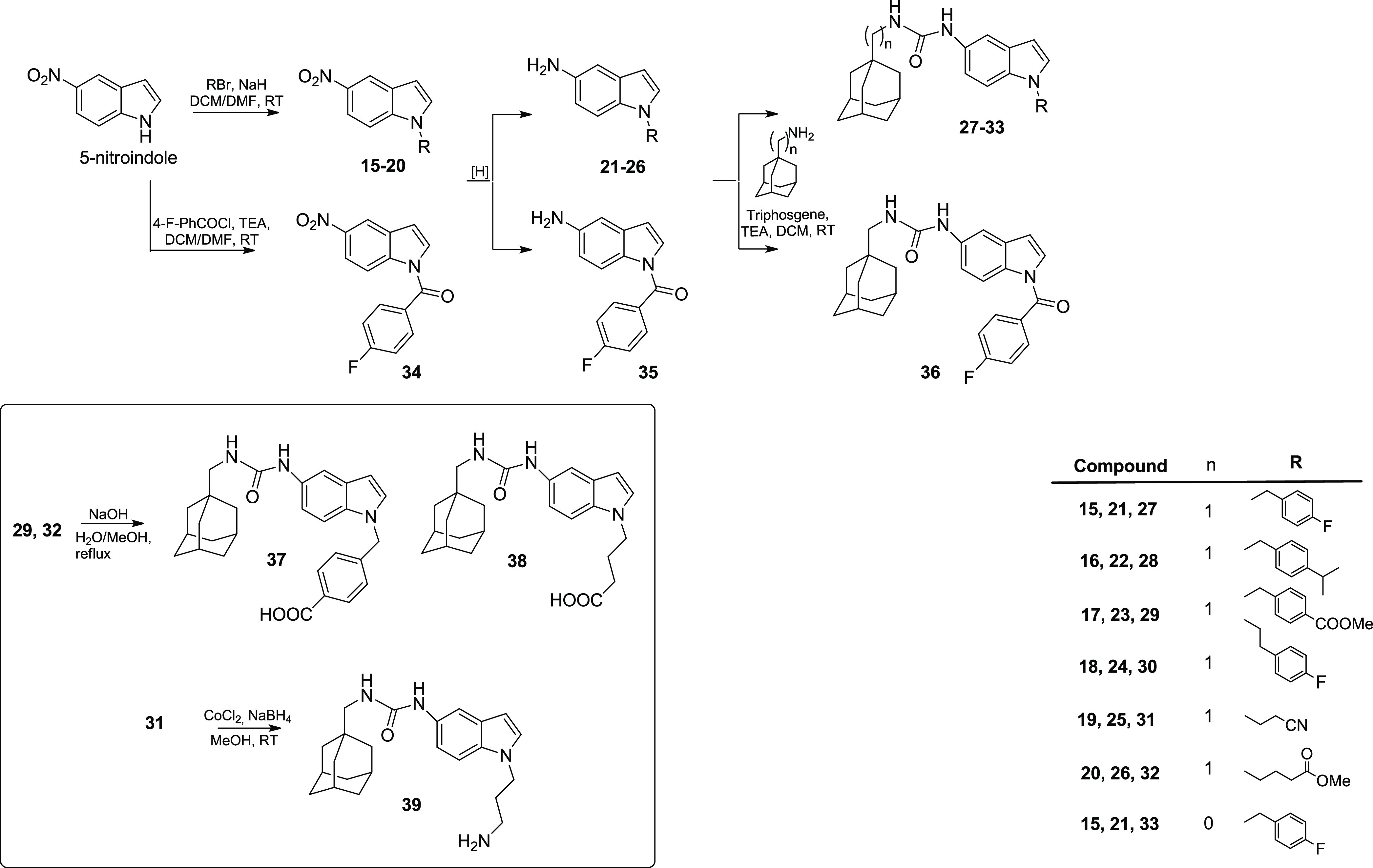
Synthesis of Indole Derivatives **27**–**33** and **36–39**

Indole final analogues were synthesized as depicted in Scheme 2.

Starting from 5-nitroindole, alkylation using different alkyl bromides and sodium hydride provided N-1 alkyl intermediates **15–20** in 62–85% yields. Continuous flow hydrogenation led to amino indoles **21–26** (55–80%) which were treated with 1-(adamantan-1-yl)methanamine, triphosgene, and TEA to give final compounds **27–32** in 39–68% yields, while the reaction with 1-adamantylamine afforded derivative **33** in 45% yield.

Final derivative **36** was obtained starting from 5-nitroindole that was coupled with 4-fluorobenzoyl chloride in basic medium to give intermediate **34** (65% yield), whose reduction in the same conditions described above led to compound **35** in 69% yield. Subsequent treatment with 1-(adamantan-1-yl)methanamine, triphosgene, and TEA gave **36** in 42% yield. Compounds **37** and **38** were synthesized starting from compounds **29** and **32**, respectively, by alkaline hydrolysis of the ester moiety (yields 58–62%). Finally, compound **39** was attained in 46% yields by reducing the nitrile moiety of **31** using sodium borohydride and CoCl_2_ as catalyst.

**Scheme 3:**
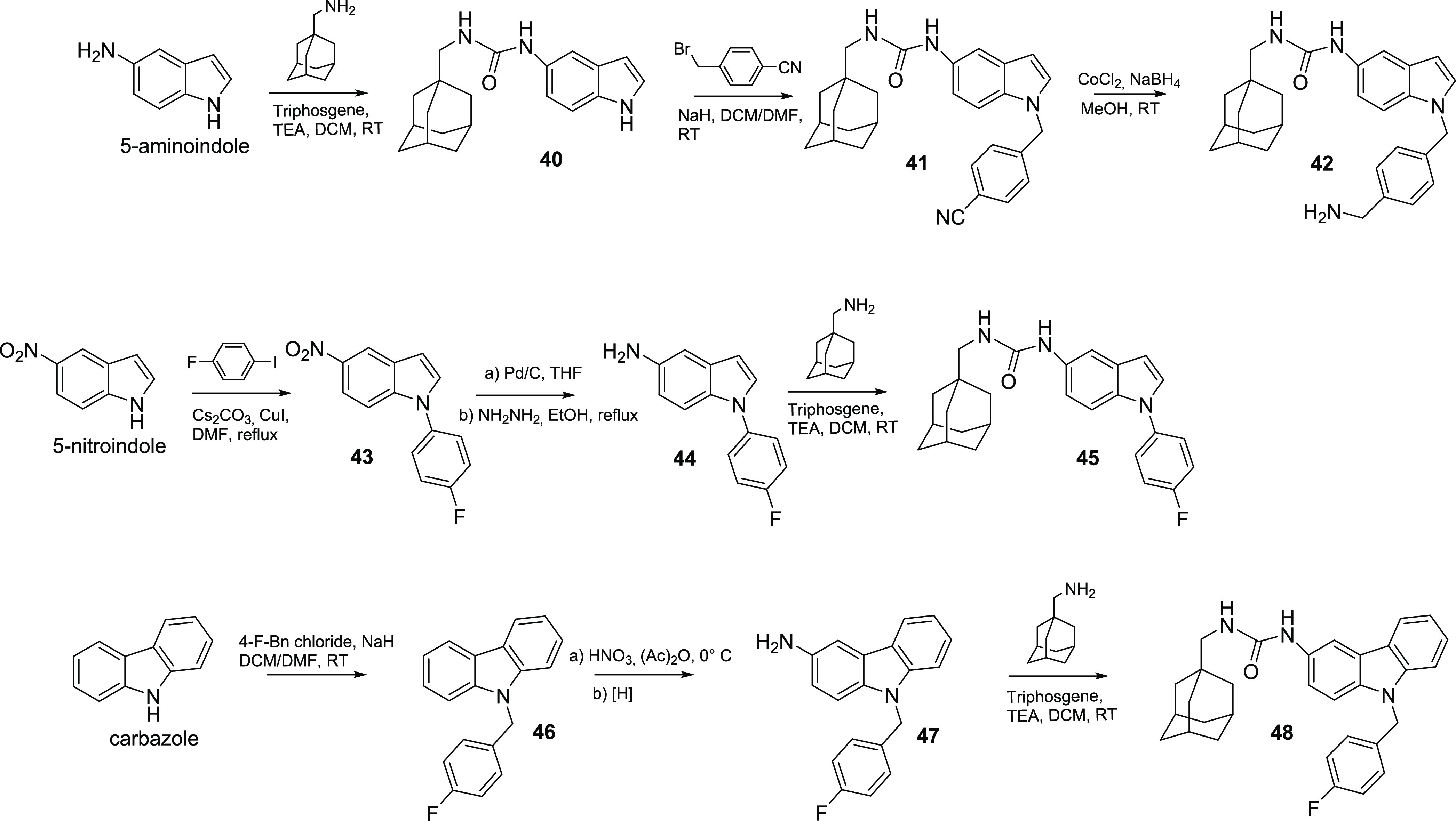
Synthesis of Indole Derivatives **42**, **45**, and Carbazole Compound **48**

The synthetic pathways used to synthesize final compounds **42**, **45**, and **48** are described in Scheme 3.

Treatment of 5-aminoindole with triphosgene, TEA, and 1-(adamantan-1-yl)methanamine provided the ureido intermediate **40** (72% yield). Alkylation of **40** with 4-(bromomethyl)benzonitrile afforded compound **41** (55% yield) that was reduced by CoCl_2_ and NaBH_4_ to give final derivative **42** in 48% yield.

Compound **45** was synthesized starting from 5-nitroindole using an alternative synthetic pathway. N-1 substitution using 4-fluoroiodobenzene, Cs_2_CO_3_ as a base, and CuI as a catalyst led to N1-aryl indole **43** in 35% yield. The following Pd/C-catalyzed reduction, in the presence of hydrazine afforded aminoindole intermediate **44** in 65% yield, which was reacted with triphosgene, TEA, and 1-(adamantan-1-yl)methanamine to give final derivative **45** (32% yield).

The synthesis of carbazole intermediate **46** was performed by N-1 alkylation using NaH and 4-fluorobenzyl chloride (97% yield). The nitration procedure using HNO_3_ in acetic anhydride and the following reduction of the nitro group afforded the amino carbazole compound **47** in 90% yield. Finally, treatment of **47** with triphosgene, TEA, and 1-(adamantan-1-yl)methanamine furnished compound **48** (65% yield).

### Molecular Docking and Evaluation of 5-LOX and sEH Inhibition

**Figure 2:**
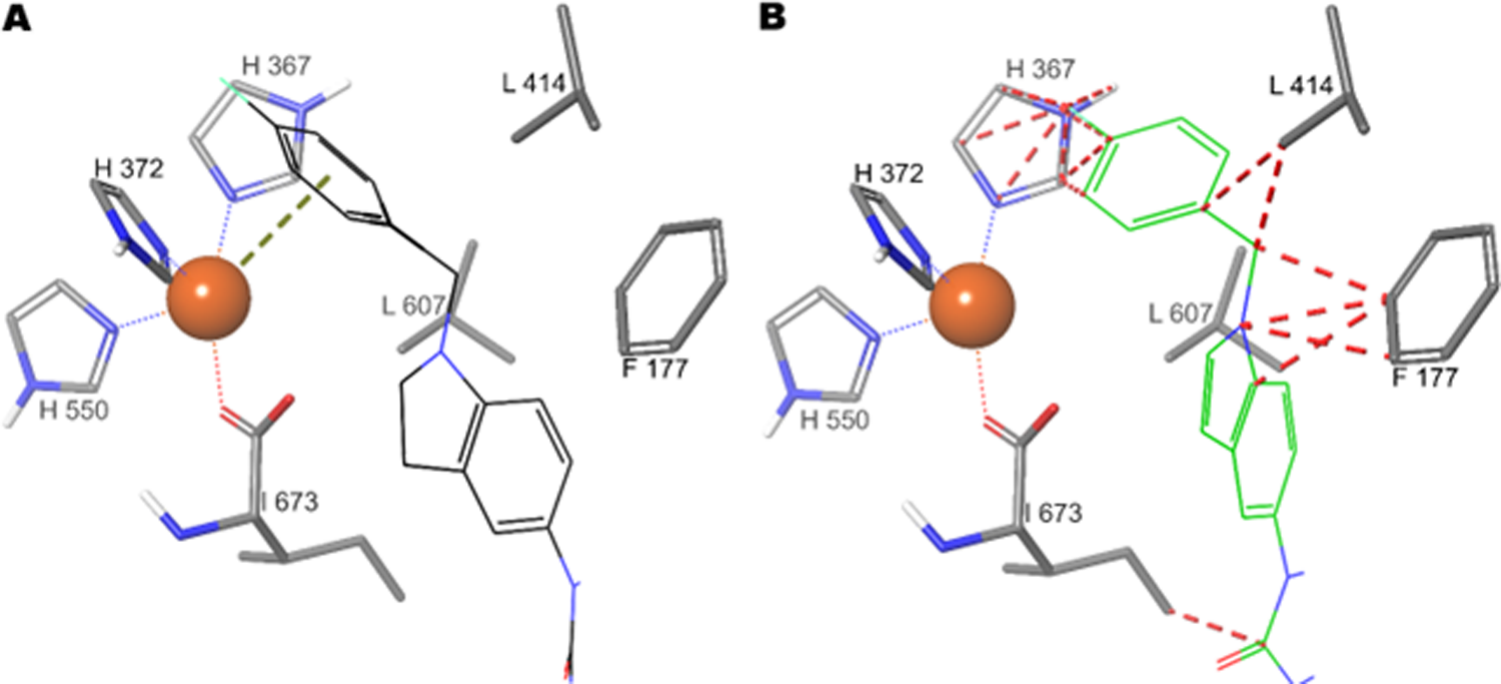
Schematic representation of the accommodation into the 5-LOX (PDB ID: 3O8Y) catalytic site of indoline (A) and indole (B). The protein is depicted in tube, while ligands in wire. The green and red dashed lines represent the π–cation interaction and steric clashes, respectively.

In order to deepen the SAR on our previously developed indoline-based dual inhibitors^20^ targeting 5-LOX and sEH, we designed a new collection of 21 small molecules. In detail, these new collection of compounds were designed by structurally modifying the indoline at N-1, C-2, C-3, and C-5 positions and replacing the indoline ring with indole or carbazole nuclei. All compounds were synthesized and tested *in vitro* to evaluate their inhibitory activity against isolated 5-LOX and sEH enzymes (Table 1). Interestingly, the *in vitro* assays showed that only compounds endowed with indoline ring effectively inhibit the 5-LOX activity, spanning from nanomolar to low micromolar range. On the contrary, the remaining congeners proved a very weak/absent 5-LOX inhibition. The experimental outcomes were corroborated by *in silico* analysis, which highlighted that the conversion from indoline to indole/or carbazole ring widens the planar geometry of the bicyclic moiety and consequently of the nitrogen-substituent bond, yielding a stiffness that does not allow an appropriate adaptation and consequent accommodation into the binding site. Indeed, no docked poses or distorted ones are found from calculations. In detail, we compared the binding pose of compound **6** and its indole analogue **33** in the 5-LOX catalytic pocket as illustrated in Figure 2. Specifically, we observed that the 5-LOX inhibition was mainly obtained through the π–cation interaction of 4-fluorobenzyl group with catalytic iron filling the open position of coordination sphere and by hampering the access to the catalytic site by the rest of the molecule. The geometry of the saturated moiety of indoline confers a proper conformation to the 4-fluorobenzyl group to fill and reach the catalytic center (Figure 2A). Unlike the indoline, the planar geometry of indole entails a conformational rigidity to the 4-flurobenzyl group which gives steric clashes with catalytic residues and consequently is pushed away from the Fe^2+^ ion (Figure 2B).

**Table 1:**
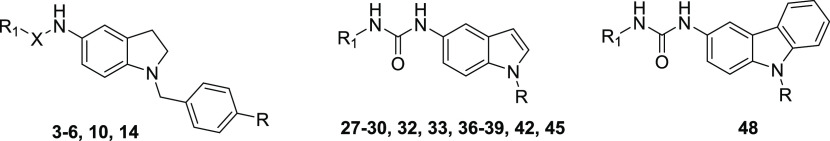
Inhibition of 5-LOX Product Formation in Human Activated PMNL and Inhibition of Human Isolated 5-LOX and sEH^a^

compound	R	R_1_	X	5-LOX residual activity in PMNL (%) at 10 μM	5-LOX IC_50_ in PMNL (μM)	IC_50_ for isolated 5-LOX (nM)	IC_50_ for isolated sEH (nM)
**3**	F	adamantyl-	C=O	53.2 ± 9.9	-	74 ± 18	285.1 ± 31.2
**4**	F	10-camphorylCH_2_-	SO_2_	2.5 ± 2.5	2.56 ± 0.56	171 ± 66	1800.1 ± 460.2
**5**	F	adamantyl-CH_2_NH-	C=O	79.7 ± 2.8	-	78 ± 53	11.1 ± 6.0
**6**	F	adamantyl-NH-	C=O	35.4 ± 3.1	0.24 ± 0.15	71 ± 16	7.22 ± 1.110
**7**	F	α-naphtylCH_2_NH-	C=O	56.3 ± 5.1	-	148 ± 28	36.1 ± 7.5
**8**	F	1-CH_3_-diazepyl(CH_2_)_2_NH-	C=O	71.1 ± 6.6	-	>1000	>10 000
**10**	F	adamantylCH_2_NH-	C=S	-	-	>1000	287.8 ± 219.5
**14**	COOH	adamantylCH_2_NH-	C=O	57.7 ± 9.6	-	>1000	24.1 ± 4.1
**27**	4-F-PhCH_2_-	adamantylCH_2_-	-	59.4 ± 6.7	-	>1000	1.752 ± 0.593
**28**	4-CH(CH_3_)_2_-PhCH_2_-	adamantylCH_2_-	-	93.6 ± 4.8	-	>1000	2.0 ± 1.1
**29**	4-COOMe-PhCH_2_-	adamantylCH_2_-	-	65.1 ± 9.4	-	>1000	11.4 ± 3.6
**30**	4-F-Ph(CH_2_)_2_-	adamantylCH_2_-	-	65.1 ± 6.2	-	>1000	1.1 ± 0.4
**32**	COOMe(CH_2_)_3_-	adamantylCH_2_-	-	30.1 ± 4.8	6.83 ± 0.84	>1000	21.3 ± 11.9
**33**	4-F-PhCH_2_-	adamantyl-	-	97.8 ± ± 0.3	-	>1000	13.4 ± 3.1
**36**	4-F-PhCO-	adamantylCH_2_-	-	58.1 ± 5.5	-	>1000	2.6 ± 0.4
**37**	4-COOH-PhCH_2_-	adamantylCH_2_-	-	2.1 ± 1.1	4.40 ± 0.42	>1000	15.1 ± 7.7
**38**	COOH(CH_2_)_3_-	adamantylCH_2_-	-	41.2 ± 7.8	8.15 ± 1.86	>1000	53.3 ± 15.9
**39**	NH_2_(CH_2_)_3_-	adamantylCH_2_-	-	51.8 ± 13.0	-	>1000	27.2 ± 11.8
**42**	4-NH_2_-CH_2_PhCH_2_-	adamantylCH_2_-	-	6.6 ± 4.0	6.19 ± 2.41	>1000	14.9 ± 4.6
**45**	4-F-Ph-	adamantylCH_2_-	-	80.9 ± 1.7	-	>1000	4.4 ± 0.5
**48**	4-F-PhCH_2_-	adamantylCH_2_-	-	92.6 ± 4.0	-	>1000	20.0 ± 3.8

a Zileuton, used as positive control at 3 μM gave residual activity of 3.90 ± 4.14 and 14.24 ± 5.88% for 5-LOX in activated human polymorphonuclear leukocytes (PMNL) and isolated 5-LOX, respectively. AUDA used as positive control at 1 μM gave a residual activity of 3.90 ± 4.14% for isolated sEH.

**Figure 3:**
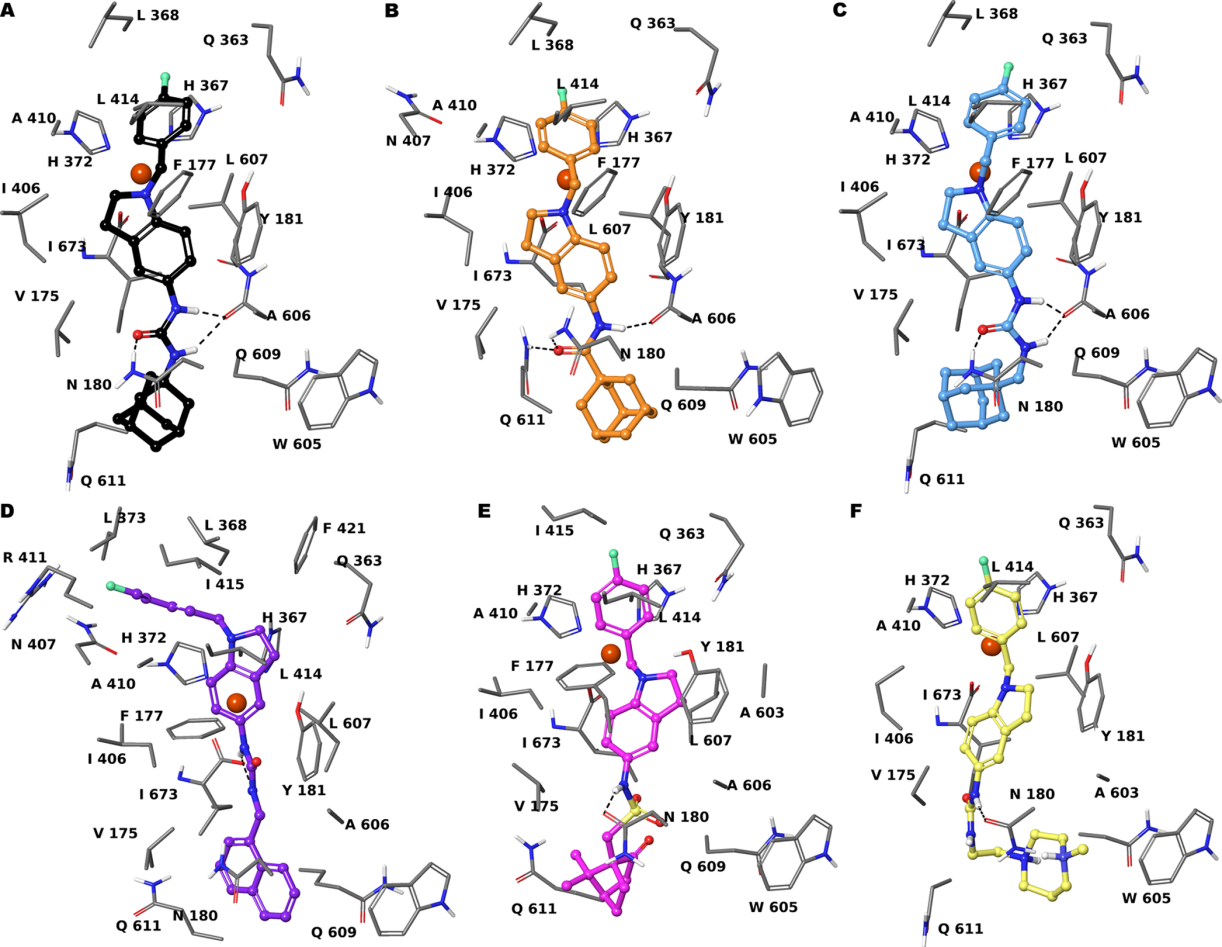
Three-dimensional model of the interactions given by **6** (A), **3** (B), **5** (C), **7** (D), **4** (E), and **8** (F) with 5-LOX (PDB ID: 3O8Y). The protein is depicted by tube (colored: C, gray; polar H, white; N, dark blue; O, red). The small molecules are represented by sticks (black for **6**, orange for **3**, faded azure for **5**, violet for **7**, magenta for **4**, faded yellow for **8**) and balls (colored: C, as for the sticks; polar H, white; N, dark blue; O, red; S, yellow). The dashed black lines indicate the hydrogen bonds between the ligand and protein.

The derivative **6** showed the highest inhibitory activity against isolated 5-LOX (IC_50_ = 71.0 ± 16.0 nM), followed by **3** (IC_50_ = 74.0 ± 18.0 nM) and **5** (IC_50_ = 78.0 ± 53.0 nM). The biological activities of **3**, **5**, and **6** were in agreement with structural observations from molecular docking (Figure 3). As illustrated above, the common 4-fluorobenzyl moiety of these compounds is engaged in a π–π stacking with the active site iron-binding H367 and H372 and a π–cation with Fe^2+^. Both 4-fluorobenzyl and indoline get close to the catalytic iron, obstructing the approach to the open position of ion coordination sphere. The indoline moiety also establishes van der Waals interactions with V175, F177, Y181, I406, L607, and I673 (Figure 3A–C). These compounds structurally differ in the linker between indoline and adamantane: an amide group in **3**, a methylene urea in **5**, and a urea group in **6**. For the docked pose of **5** and **6**, a π–π stacking between indoline and the side chain of the active site-concealing F177 was observed (Figure 3B,C). In **3**, this π-stacking was not found because the adamantyl group hampers a closer approach to the side chain of F177 due to the shorter linker length between indoline and adamantane. The urea group in both **5** and **6** donates two hydrogen bonds to the amide carbonyl of A606, while accepting a H-bond from the side chain of N180. The amide group of **3** accepts two hydrogen bonds from N180 and Q611 and donates a H-bond to the backbone CO of A606. The adamantane group gives van der Waals interactions with surrounding residues, but in **5** it is less enveloped by macromolecular counterparts due to the presence of methylene (Figure 3C). Compounds **4** and **7** showed a comparable inhibition of 5-LOX activity, even though lower than **6** (around 2.5 and 2 times lower than **6**, see Table 1). Compared to **6**, the substitution of adamantyl group with a naphthalene induced a different conformational arrangement of **7** (Figure 3D). Indeed, the indoline ring is 180° rotated with respect to **6**, and the urea group shifts toward the backbone CO of the iron-coordinating I673, donating two H-bonds. Compared to **6**, this shift causes the loss of a hydrogen bond and of some van der Waals contacts by the indoline ring, preserving a π-stacking with F177. Moreover, the 4-F-benzyl moved away from H367 losing a π–π interaction (Figure 3D). As for **7**, the indoline of **4** is 180° rotated and similar consideration could be inferred. It is noteworthy that **4** differs from **6** in the substitution of urea with a sulfonamide donating a H-bond to the side chain of N180. The (1*R*)-7,7-dimethylbicyclo[2.2.1]heptan-2-one group of **4** is superimposable to the adamantyl of **6** (Figure 3E). The docked pose of **8** showed the 1-ethylene-4-methyl-1,4-diazepane moiety quite folded to establish a hydrogen bond with backbone CO of Q609 with an unfavorable entropic loss (Figure 3F). Moreover, this induces a 90° rotation of the urea group with respect to **6**, with deficiency of a hydrogen bond with N180. Indeed, **8** presented an inhibitory profile in the low micromolar range. Compounds **14** and **10** did not show a proper fit into the 5-LOX binding cavity, justifying the very weak inhibition of enzymatic activity, higher than 1 μM as for compound **8**.

**Figure 4:**
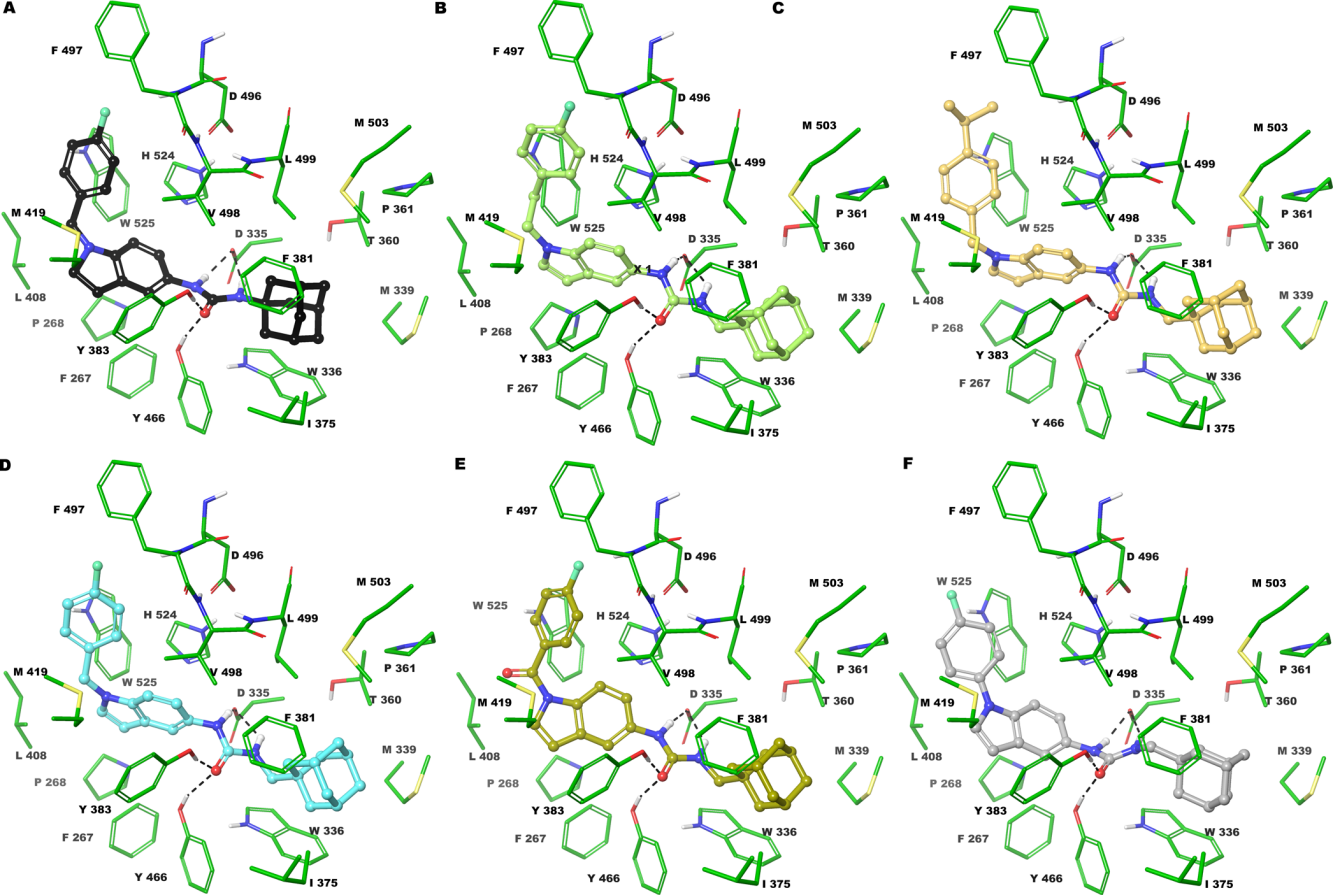
Three-dimensional model of the interactions given by **6** (A), **30** (B), **28** (C), **27** (D), **36** (E), and **45** (F) with sEH (PDB ID: 3I28). The protein is depicted by tube (colored: C, green; polar H, white; N, dark blue; O, red). The small molecules are represented by sticks (black for **6**, faded yellow-green for **30**, gold for **28**, cyan for **27**, kiwi for **36**; light gray for **45**) and balls (colored: C, as for the sticks; polar H, white; N, dark blue; O, red; S, yellow). The dashed black lines indicate the hydrogen bonds between the ligand and protein.

The *in vitro* tests against sEH enzyme highlighted that most of the compounds are active in the nanomolar range with different effectiveness. In particular, five out of 21 small molecules showed a very potent inhibitory effect (IC_50_ < 8 nM, Table 1), namely, **6**, **27**, **28**, **30**, and **36**. They appear well accommodated into the binding cavity filling equal space and establishing the same pattern of intermolecular interactions by their identical structural portions (Figure 4A–E). In detail, the urea group donates two hydrogen bonds to the side chains of D335 and accepts two H-bonds from Y383 and Y466. The adamantyl group gives van der Waals contacts with Y336, M339, T360, F381, Q384, L499, and M503. The indole ring (**27**, **28**, **30**, and **36**) establishes π–π interaction with H524 and W525 (Figure 4B–E). In **6**, the indoline is superimposable to indole of **27**, **28**, **30**, and **36** but does not present the π-stacking with W525 (Figure 4A), justifying its higher IC_50_ in this set of inhibitors (from 2.8 to 7 times higher than **27**, **28**, **30**, and **36**). Interestingly, all these observed intermolecular contacts are also detected by 34N co-crystallized with sEH.^24^ All synthesized compounds are endowed with a phenyl ring directly bound to bicyclic nitrogen (**45**) or through a linker: ethylene (**30**), methylene (**27**, **28**), or carbonyl group (**7**). Compared to **36** and **45**, we observed for **27**, **28**, and **30** a better π-stacking with W525 due to the higher flexibility of the linker, as demonstrated by their IC_50_ values against sEH (Figure 4B–D). Compounds **5**, **29**, **37**, and **42**, showed an inhibitory activity ranging from 10 to 20 nM, slightly lower than the analogues described so far. Compound **5** lacks a π-stacking interaction with W525 because it is endowed with an indoline ring (Figure S64A). Compounds **29**, **37**, and **42** contain a benzyl group at N1, para-substituted with a methyl ester, carboxylic acid, or methenamine group, respectively (Figure S64). The presence of this substituent in the para position of the benzyl group displaces the urea moiety and indole ring, losing a hydrogen bond with N335 and a π-stacking with H524 and W525. However, the methylamine of **42** is hydrogen-bonded with backbone CO of R410 and V416, whereas the carboxylate group of **37** accepts two H-bonds from the amide backbone of F497 and H524. Compound **29** is only H-bonded to the amide backbone of H524 but gives two π-stacking with W525. Interestingly, **33** differs from **6** in the indole ring which confers rigidity (Figure S64E). Thus, **33** rotates 90° its bicyclic portion to fit the binding cavity moving away from W525 but maintaining a π–π with this residue. Similar considerations could be made for **14**, which showed an IC_50_ = 24.4 ± 4.1 nM. Comparable inhibitory activities to **14**, were shown by **32**, **38**, and **39**, all structurally featured with linear alkyl chain at N1, ending with an ester, carboxylic, or amine group, respectively (Figure S65). The amine group of **39** gives an ionic interaction with D496, whereas the ester and carboxylic groups of **32** and **38** accept two hydrogen bonds from the backbone NH of F497 and H524. However, the linear alkyl chain showed few van der Waals contacts balancing its entropic loss upon binding. For derivative **7**, the presence of naphthalene moiety increases the distance from H524 even though the π-stacking is kept, and the loss of an H-bond with N335. Moreover, the indoline does not make the π–π interaction with the side chain of W525. Compound **48** differs from its congeners in the presence of carbazole instead of a bicyclic ring (indoline/or indole). This bulkier tricyclic moiety accommodates near Y383 establishing π–π interactions but losing the same contacts with H524 and W525 (Figure S65F). Moreover, its urea group gives three hydrogen bonds, despite a network of four H-bonds as for the more active analogues. The conversion of urea to thiourea provides only one hydrogen bond with D335 (**10**, Figure S66A). This observation agrees with our previous studies and with data reported in the literature, suggesting that the urea group is a crucial structural requirement to potently inhibit sEH.^20^ Moreover, it is here demonstrated that the amide group was not able to give the network of H-bonds observed for the urea group. Indeed, we noted three hydrogen bonds in **3** (Figure S66B). Moreover, the amide group is shorter than the urea group and the adamantyl moiety is too close to some residues such as L499 or W336 to give favorable contacts explaining its higher IC_50_ (285.1 ± 31.2). Compounds **4** and **8** are not well accommodated into the sEH binding cavity giving no optimal interactions with macromolecular counterparts, in accordance with showed poor/absent inhibition activities. Collectively, our results confirmed the importance of adamantyl and urea groups in sEH inhibition. Any attempt to replace these groups failed to provide potent sEH inhibitors. In our series of compounds, moreover, the use of a methylene spacer between the adamantyl and the urea group is responsible for increased activity, as observed in compounds **27** and **33**. Substitutions at the indole N-1 are in general well tolerated, with aromatic and nonpolar moieties preferred to aliphatic and/or polar moieties.

### *In Vitro* Pharmacokinetic Evaluation

Considering the data collected from the *in vitro* activity screening, we decided to perform an *in vitro* pharmacokinetic evaluation of the most potent compounds. The results obtained are summarized in Table 2. Compound **6** was selected for pharmacokinetic screening as it is the most potent dual 5-LOX/sEH inhibitor identified, with IC_50_ values in the nanomolar range for both enzymes. Moreover, compound **6** showed low micromolar potencies, also for 5-LOX activity in intact PMNL. On the other hand, in view of the inhibitory potencies versus sEH and their selectivity (being devoid of 5-LOX inhibition), compounds **27**, **28**, **30**, and **36** were also tested for their *in vitro* pharmacokinetic profile.

**Table 2:** Calculated Pharmacokinetic Parameters for the Tested Compounds

*in vitro* pharmacokinetic parameters						
		compounds				
	time (h)	**6**	**27**	**28**	**30**	**36**
achemical stability in phosphate buffer (pH 7.4)	0.5	97.9 ± 3.5	99.2 ± 1.7	102.0 ± 2.7	102.5 ± 1.1	96.3 ± 3.2
	1.0	100.7 ± 4.3	95.5 ± 4.5	101.7 ± 1.9	100.3 ± 1.9	95.2 ± 1.8
	1.5	100.5 ± 3.5	98.9 ± 0.2	99.7 ± 2.3	99.6 ± 0.2	99.8 ± 2.1
	2.0	102.4 ± 0.1	96.4 ± 0.5	102.4 ± 1.8	102.4 ± 3.5	92.4 ± 2.0
amouse plasma stability	1.0	97.3 ± 0.7	100.3 ± 0.2	98.4 ± 3.3	96.2 ± 3.4	98.2 ± 2.0
	2.0	100.7 ± 2.3	102.2 ± 4.8	101.3 ± 3.8	97.0 ± 2.3	96.2 ± 2.8
aUGTs/CYPs “dual-activity” mouse liver microsomal stability	0.25	21.3 ± 0.9	8.4 ± 0.3	91.5 ± 2.2	16.6 ± 1.4	20.4 ± 1.8
	0.5	10.9 ± 0.1	3.2 ± 1.0	69.8 ± 2.9	9.3 ± 0.6	11.9 ± 0.6
	1.0	10.6 ± 0.1	2.1 ± 0.7	53.1 ± 2.8	5.7 ± 0.2	2.4 ± 0.4
b*in vitro* half-life (min)		20.4	11.9	62.9	16.0	11.8
cmicrosomal intrinsic clearance (CL_int,micr_, μL min^–1^ mg^–1^)		67.8	116.8	22.0	86.6	117.9
d*in vivo* intrinsic (hepatic) clearance (CL_int_, mL min^–1^ kg^–1^)		265.4	457.3	86.2	338.9	461.4

a (%) 100 – [concentration at time points min /concentration at 0 min] × 100.

b 0.693/*k*, where *k* is the slope of linear regression of the percentage parent compound remaining against time.

c ln 2/*t*_1/2_ × [volume of incubation medium (μL)/microsomal protein in incubation (mg)].

d CL_int,micr_ × (mg microsome g^–1^ liver) × [liver mass (g)/body mass (kg)].

Initially, the chemical stability of the compounds was studied in phosphate buffer at pH 7.4. All the tested derivatives proved to be highly stable up to 2 h (Table 2). The same stability was evident in mouse plasma at two different time points (1 and 2 h, Table 2). In addition, all the compounds were subjected to an *in vitro* microsomal stability assay. In this assay, the metabolism of the parent compound over time (up to 60 min) was measured by liquid chromatography–mass spectrometry (LC–MS) in the presence of CYP450 and UGTs (uridine 5′-diphospho-glucuronosyltransferases) microsomal systems. The measurement of hepatic metabolism also allowed the determination of different pharmacokinetic parameters such as *in vitro* intrinsic clearance (CL_int,micr_) and to predict *in vivo* intrinsic clearance (CL_int_). The predicted *in vivo* CL_int_ values were calculated using a microsomal protein value of 45 mg per gram of liver tissue and 87 g of liver per kg of body weight. The microsomal stability assay was validated using testosterone (low stability in the presence of NADPH), 2-naphthol (low stability using NADPH and UDPGA cofactors), and 3-(α-acetonylbenzyl)-4-hydroxy coumarin (high stability in dual-activity assay) as positive controls. To detect nonspecific protein binding or heat instability, the compounds were incubated without cofactors (negative control). Moreover, since microsomal concentrations > 2 mg mL^–1^ are not recommended due to a potential negative impact of increased nonspecific binding on the rate of metabolism,^25^ in our assay we used a low concentration of liver microsomes (0.5 mg mL^–1^). Under these conditions, all compounds showed no significant protein binding and high stability in the absence of cofactors.

The results obtained pinpoint compound **28** as the most stable derivative with a CL_int,micr_ of 22.0 μL min^–1^ mg^–1^ (Table 2). All of the other compounds, indeed, underwent a sustained microsomal metabolism with a microsomal intrinsic clearance ranging from 67.8 to 117.9 μL min^–1^ mg^–1^. These results reveal compound **28** as the most suitable for further testing. To discover and characterize compound **28** metabolites generated after incubation with mouse liver microsomes, a strategy integrating high-resolution liquid chromatography and tandem mass spectrometry (LC-MS/MS) data and advanced processing algorithms in the Compound Discoverer software, was employed. The metabolites were tentatively characterized by their accurate mass, fragmentation pattern, and retention times (Table S1).

LC-MS/MS analysis showed that the main metabolites were mono- and dioxidized derivatives. In detail, M1–M2 metabolites (*R*_t_: 9.44 and 9.53 min, respectively) presented the same precursor ion [M – H]^+^ at *m*/*z* 472 (C_30_H_38_N_3_O_2_) and showed a 16 Da mass difference versus the parent compound (Figure S68), indicating a single oxidation reaction.

The fragment ions of M1 and M2 at *m*/*z* 182 (C_11_H_20_ON) and *m*/*z* 165 (C_11_H_17_O^+^) indicated possible oxidation of adamantane ring, as confirmed by fragment at *m*/*z* 149 (C_11_H_17_) derived from the loss of the OH group (Figure S69). On the other hand, M4–M6 metabolites (*R*_t_: 8.36, 8.80, and 8.98, respectively) with [M – H]^+^ at *m*/*z* 488 (C_30_H_38_O_3_N_3_) showed 32 Da mass difference compared to **28** and were proposed as dioxidized derivatives. The hypothesis that the oxidation reactions occurred mainly on the adamantane ring is supported by fragment ions at *m*/*z* 198 (C_11_H_20_NO_2_), *m*/*z* 181 (C_11_H_17_O_2_) and *m*/*z* 163 (C_11_H_15_O) suggesting a double hydroxylation on the alicyclic group (Figure S70). Finally, a minor mono-oxidized metabolite (M3, [M – H]^+^ at *m*/*z* 472 C_30_H_38_O_2_N_3_) was identified at 10.36 min. MS2 analysis gave a fragment at *m*/*z* 281 (C_18_H_21_ON_2_) suggesting a potential oxidation site on the isopropyl-benzyl-indolamine moiety.

**Figure 5:**
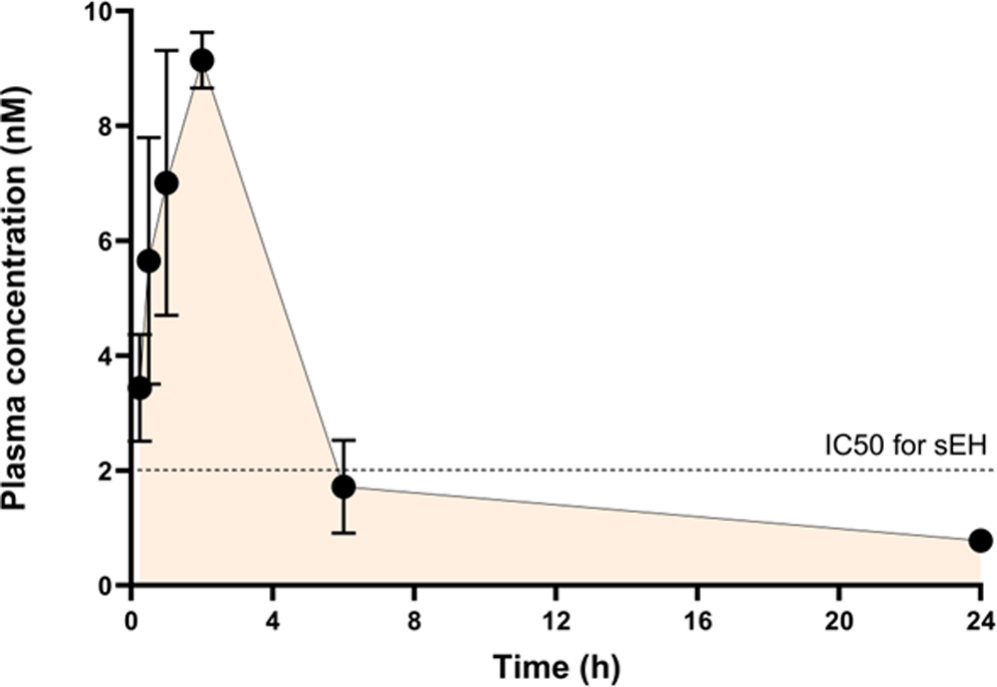
Plasma concentration–time curve after a single dose of compound **28** injected i.p. in mice. Values are represented as mean ± standard deviation (SD) (*n* = 6).

Next, the *in vivo* pharmacokinetic properties of **28** were assessed. To this aim, blood samples were collected from animals at predetermined intervals (0.25, 0.5, 1, 2, 6, and 24 h) after i.p. administration of **28** and, upon extraction, were analyzed by high-performance liquid chromatography/tandem mass spectrometry (HPLC-MS/MS, Table S2) to quantify **28**. Results obtained (Figure 5) show substantial stability of compound **28**, with a plasma half-life in mice of 7.6 h (*T*_max_: 2h; *C*_max_: 4.17 ng mL^–1^; AUC_0*–*∞_: 29.9 ng/mL h).

### Effects of Compound **28** on PG Production in Activated Murine Macrophages

During the inflammatory process, AA is metabolized by COX enzymes to the synthesis of pro-inflammatory PGs. We evaluated the effects of compound **28** on PGE_2_ production in LPS-stimulated J774 murine macrophages. Stimulation of J774 macrophages with LPS (10 μg/mL) for 24 h induced a significant increase of PGE_2_ levels in comparison to unstimulated cells, which is mainly mediated by inducible COX-2 in this experimental setting (Figure S67). The pre-treatment (2 h) of cells with **28** (0.1–10 μM) reduced the PGE_2_ levels in LPS-stimulated macrophages (Figure S67) with an IC_50_ higher than 10 μM, excluding COX-2 inhibition but further supporting the selectivity of **28** for sEH, at least within the AA cascade. Overall, molecular docking, *in vitro* biological investigations, and *in vitro* pharmacokinetic parameters suggest **28** as a promising lead for further *in vivo* pharmacological studies.

### Compound **28** Displays Protective Effects in Mouse Cerulein-Induced Acute Pancreatitis

**Figure 6:**
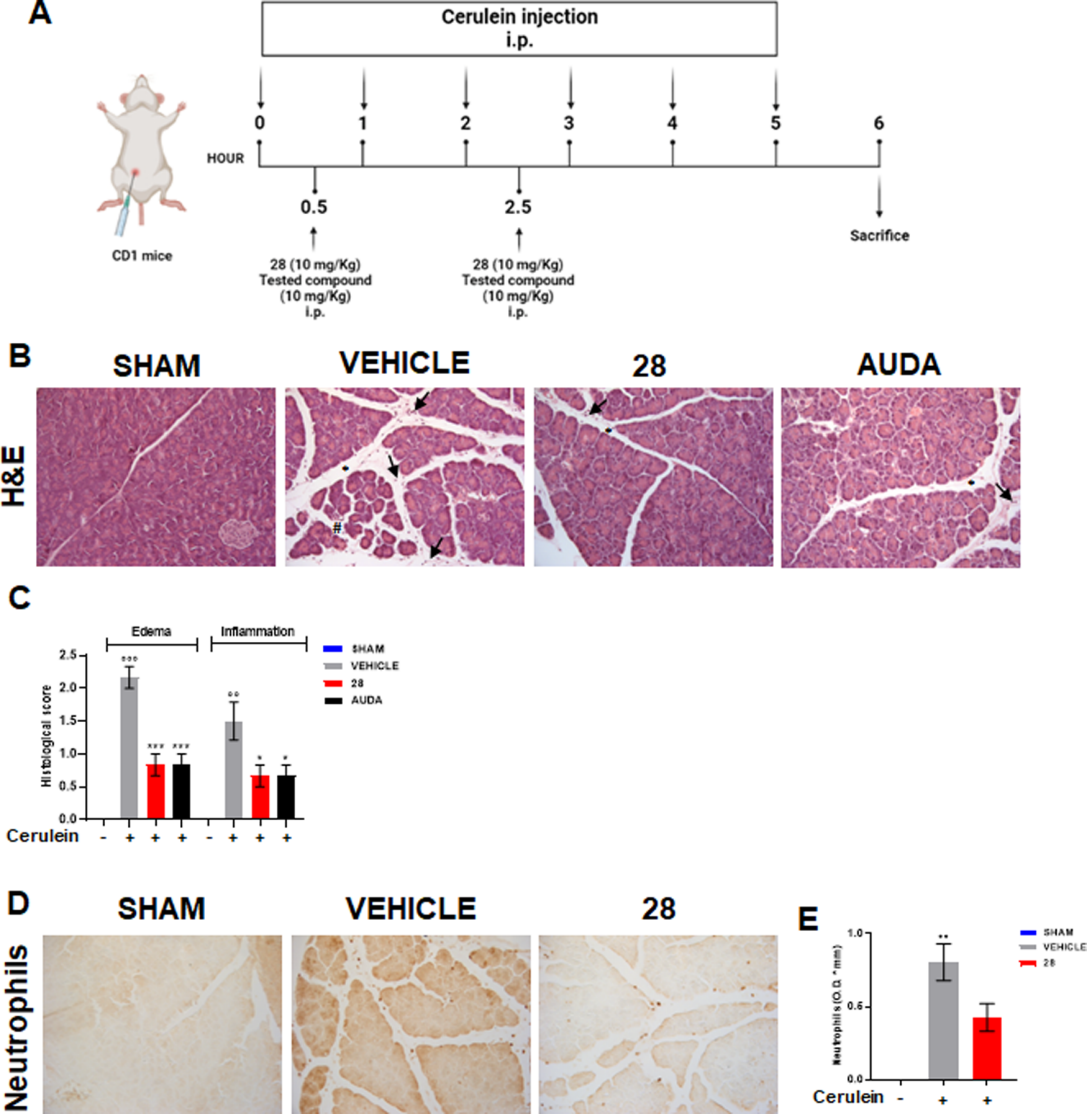
Effect of compound **28** in a murine model of acute pancreatitis. (A) Timescale for cerulein-induced murine pancreatitis. Pancreatitis was induced in mice by i.p. injections of cerulein (50 μg/kg) hourly (5 times). Mice received compound **28** (10 mg/kg, i.p.) or AUDA (10 mg/Kg, i.p.) 0.5 and 2.5 h after the first cerulein injection and were killed 6 h after the first cerulein injection. (B) Pancreas slices were stained for H&E. (C) Evaluation of pancreatic edema and inflammatory infiltration was performed by three-point scoring system. Edema: 0 absent or rare; 1, in the interlobular space; 2, in the intralobular space; 3, the isolated-island shape of pancreatic acinus. Inflammation: 0, absent; 1, mild (infiltration in ducts); 2, moderate (infiltration in parenchyma < 50%); 3, severe (infiltration in parenchyma > 50%). Asterisk = interlobular edema, hash sign = edema, arrows = inflammatory cells. (D) Immunohistochemical analysis of neutrophils in pancreas sections. (E) Semiquantitative determination of neutrophil expression was obtained with ImageJ/Fiji software. Values represent mean ± standard error of the mean (SEM); *n* = 5 mice for each group. Data were analyzed by one-way analysis of variance (ANOVA) plus Bonferroni. Statistical significance is reported as follows: °° *P* < 0.01 and °°° *P* < 0.001 *vs* Sham; * *P* < 0.05 and *** *P* < 0.001 *vs* Vehicle.

AP is a gastrointestinal disorder that starts as a local pancreatic inflammatory response that often leads to systemic inflammation. To date, no specific therapies are available.^26,27^ Recently, it has been demonstrated that genetic or pharmacological inhibition of sEH can modulate the severity of AP,^11,28^ due to the potent anti-inflammatory properties of the EETs that accumulate when sEH is blocked. To evaluate the *in vivo* anti-inflammatory effectiveness of compound **28**, we choose the murine model of AP induced by repeated cerulein injections (Figure 6A). Cerulein is an ortholog of the intestinal hormone cholecystokinin and at high concentrations causes death of acinar cells, edema, and infiltration of inflammatory cells into the pancreas, typical hallmarks observed in human pancreatitis. All animals performed similarly without mortality during the development of pancreatitis. H&E staining of the pancreas in the cerulein plus vehicle group showed interlobular and interstitial edema and immune cell infiltration (Figure 6B,C). In addition, prominent neutrophilia was observed in the pancreas of cerulein plus vehicle-treated animals compared to sham animals (Figure 6D,E). Compound **28** displayed a protective effect evidenced by the reduction of edema, cell infiltration (Figure 6B,C), and neutrophil numbers (Figure 6D,E). Similarly, AUDA, a known sEH inhibitor used as a reference compound, reduced pancreatic injury (Figure 6B,C).

**Figure 7:**
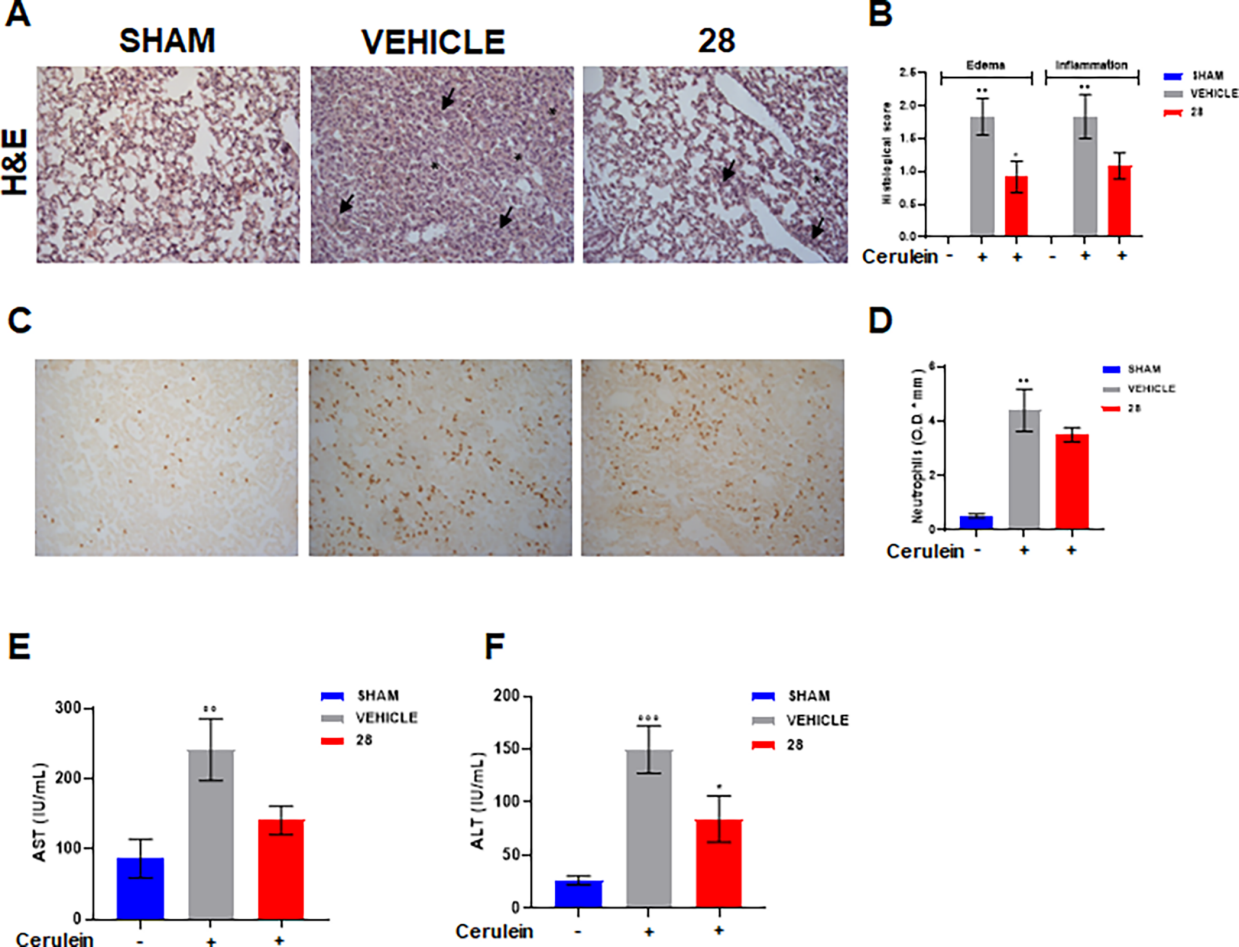
Effects of compound **28** over AP induced lung injury. Pancreatitis was induced in mice by i.p. injections of cerulein (50 μg/kg) hourly (5 times). Mice received compound **28** (10 mg/kg, i.p.) 0.5 and 2.5 h after the first cerulein injection and were killed 6 h after the first cerulein injection. (A) H&E staining of lung sections. (B) Evaluation of lung edema and inflammatory infiltration was performed with three-point scoring system: 0, absent; 1, mild; 2, moderate; 3, severe. Asterisk = edema, arrows = inflammatory cells. Analysis was performed in a blinded manner. (C) Immunohistochemical analysis of neutrophils in lung slices. (D) Semiquantitative determination of neutrophil expression was obtained with ImageJ/Fiji software. Plasma levels of AST (J) and ALT (K). Values represent mean ± SEM; *n* = 5 mice for each group. Data were analyzed by one-way ANOVA plus Bonferroni. Statistical significance is reported as follows: °° *P* < 0.01 and °°° *P* < 0.001 *vs* Sham; * *P* < 0.05 *vs* Vehicle.

Patients with pancreatitis are known to die due to multiorgan failures caused by the development of systemic inflammation as a consequence of activation of various enzymes, cytokines, and vasoactive substance.^29^ In particular, severe parenchyma damage and several biochemical abnormalities develop and reflect alterations in organs such as lungs and liver. Indeed, in our experimental conditions, cerulein administration induced lung injury characterized by pulmonary edema, abundance of inflammatory cell infiltrates (Figure 7A,B), and neutrophilia (Figure 7C,D). Lung inflammation as well as neutrophilia were reduced by the administration of compound **28** (Figure 7A–D). After pancreatitis induction, we also observed an increase of serum AST (Figure 7E) and ALT (Figure 7F) levels that were decreased after treatment with **28**.

### Evaluation of Lipid Mediator Modulation by Compound **28** Using Targeted Metabololipidomics

**Figure 8:**
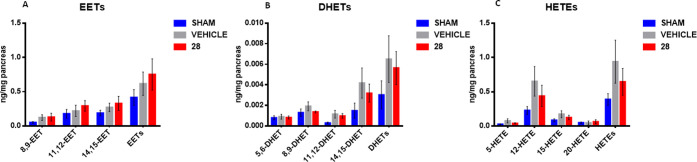
Levels of EETs (A), DHETs (B), and HETEs (C) in pancreas homogenates; graph bar color: blue sham (*n* = 6), gray vehicle (*n* = 6) red compound **28** (*n* = 6); data are expressed as ng/mg of tissue plus standard error of the mean (SEM).

To further explore sEH inhibition by **28**, we proceeded to measure a panel of eicosanoids in pancreas homogenates following vehicle or compound **28** administration by RP-UHPLC-MS/MS. The following eicosanoids were quantified: CYP epoxygenase-derived: 14,15-EET, 11,12-EET, 8,9-EET, and 5,6-EET, sEH-derived vic-diols 14,15-DHET, 11,12-DHET, 8,9-DHET and 5,6-DHET, and LOX/CYP-derived hydroxyeicosatetraenoic acids 20-HETE, 15-HETE, 12-HETE, and 5-HETE. The results highlighted that following compound **28** administration (Figure 8A), a substantial increase in the levels of EETs was observed with respect to vehicle (+21.9%), while, on the other hand, a reduction of DHET levels (Figure 8B) was obtained (−31.5%). The trend of EETs and DHETs modulation suggests that compound **28** suppressed sEH activity *in vivo*, thus reflecting the beneficial *in vivo* anti-inflammatory effect of sEH inhibition, as also previously observed through protection against cytokine-induced pancreatic β-cell-induced disfunction,^30^ and in inhibiting pancreatic cancer growth.^31^ Noteworthy, the inhibition of sEH had an additional effect on the production of LM from other pathways, in fact, treatment with compound **28** reduced the levels of different HETEs (Figure 8C), specifically 15-HETE, 12-HETE, and 5-HETE, which were lower with respect to vehicle-treatment (−13.4%). LOX-derived HETEs are known to promote inflammation, notably, these metabolites, in particular 12-HETE have been positively correlated with pancreatic cancer proliferation.^32^ These observations are in accordance with results from previous studies with sEH inhibitors that resulted in a crosstalk with other branches of AA cascade.^33^ While the observed trend was not able to reach statistical significance, the behavior of LM levels supports the anti-inflammatory effect through sEH inhibition, resulting in stabilizing the pro-resolving EET and reducing the production of LOX-derived pro-inflammatory LM.

## Conclusions

The treatment of AP, a widely diffused gastrointestinal inflammatory disease, represents nowadays an unmet medical need. The therapeutic approach mainly consists of fluid resuscitation,^34^ nutritional support,^35^ antibiotic prophylaxis,^36^ and administration of anti-inflammatory drugs,^37^ while in the most severe cases, surgery is the preferred option.^38^ In particular, nonsteroidal anti-inflammatory drugs (NSAIDs) that act as cyclooxygenase (COX)-1 and -2 inhibitors, were found to be effective in reducing cytokine signaling, pain, and the mortality rate in AP.^37^ However, the well-known drawbacks of the therapies based on traditional NSAIDs pose several questions about their extensive use,^39^ therefore, increasing the interest in the development of new therapeutic agents targeting different pathways in the inflammatory LM cascade. In the present paper, we have proposed and validated the use of sEH inhibitors as suitable AP therapeutics. Starting from a previous dual 5-LOX/sEH inhibitor we have disclosed the structural requirements leading to selective sEH inhibition, mainly represented by the transition from the indoline to the indole nucleus. Preliminary pharmacokinetic *in vitro* experiments identified compound **28** as the most suitable for *in vivo* administration. When challenged in a cerulein-induced AP murine model, compound **28** proved to be able in reducing the inflammatory damage both in pancreas and secondary sites, such as lungs and liver. These results can be also justified on the basis of the long *in vivo* plasma half-life, demonstrated by compound **28**. In accordance, compound **28** showed to modulate *in vivo* sEH-dependent inflammatory lipid mediator level, as assessed by targeted lipidomic studies. Collectively, these results pave the way for the development of potent and selective sEH inhibitors to be profitably used in the treatment of AP.

## Experimental Section

### General

(±)5,6-Epoxy-8*Z*,11*Z*,14*Z*-eicosatrienoic acid (5,6-EET), (±)8,9-epoxy-5*Z*,11*Z*,14*Z*-eicosatrienoic acid (8,9-EET), (±)11,(12)-epoxy-5*Z*,8*Z*,14*Z*-eicosatrienoic acid (11,12-EET), (±)14(15)-epoxy-5*Z*,8*Z*,11*Z*-eicosatrienoic acid (14,15-EET), (±)14(15)-epoxy-5*Z*,8*Z*,11*Z*-eicosatrienoic-16,16,17,17,18,18,19,19,20,20,20-d11 acid (14,15-EET-d11), (±)5,6-dihydroxy-8*Z*,11*Z*,14*Z*-eicosatrienoic acid (5,6-DHET), (±)8,9-dihydroxy-5*Z*,11*Z*,14*Z*-eicosatrienoic acid (8,9-DHET), (±)11,12-dihydroxy-5*Z*,8*Z*,14*Z*-eicosatrienoic acid (11,12-DHET), (±)14,15-dihydroxy-5*Z*,8*Z*,11*Z*-eicosatrienoic acid (14,15-DHET), and (±)11,12-dihydroxy-5*Z*,11*Z*,14*Z*-eicosatrienoic-16,16,17,17,18,18,19,19,20,20,20-d11 acid (11,12-DHET-d11) were purchased from Cayman Chemical (Michigan). All of the other reagents and solvents were purchased from Merck (Milan, Italy). Reactions were performed under magnetic stirring in round-bottom flasks unless otherwise noted. Moisture-sensitive reactions were conducted in oven-dried glassware under a nitrogen stream, using freshly distilled solvents. Thin-layer chromatography (TLC) analysis of reaction mixtures was performed on precoated glass silica gel plates (F254, 0.25 mm, VWR International), while crude products were purified by the Isolera Spektra One automated flash chromatography system (Biotage, Uppsala, Sweden), using commercial silica gel cartridges (SNAP KP-Sil, Biotage). NMR spectra were recorded on a Bruker Avance 400 MHz apparatus, at room temperature. Chemical shifts were reported in δ values (ppm) relative to internal Me_4_Si for ^1^H and ^13^C NMR. *J* values were reported in hertz (Hz). ^1^H NMR peaks were described using the following abbreviations: s (singlet), d (doublet), t (triplet), and m (multiplet). High-resolution mass spectrometry (HR-MS) spectra were recorded by LTQ-Orbitrap-XL-ETD mass spectrometer (Thermo Scientific, Bremen, Germany), equipped with an ESI source. All the final compounds showed a purity ≥95% as assessed by RP-UHPLC-PDA analysis, performed using a Nexera UHPLC system (Shimadzu, Kyoto, Japan) consisting of a CBM-40 lite controller, two LC-40B X3 pumps, an SPD-M 40 photodiode array detector, a CTO-30A column oven and, a SIL-40C X3 autosampler. The chromatographic analysis was accomplished on a Kinetex Evo C18 column, 150 mm × 2.1 mm × 2.6 μm (Phenomenex, Bologna, Italy) maintained at 40 °C. The optimal mobile phase consisted of 0.1% HCOOH/H_2_O v/v (A) and 0.1% HCOOH/ACN v/v (B) delivered at a constant flow rate of 0.3 mL/min. Analysis was performed in gradient elution as follows: 0–20.00 min, 5–95% B; 20.00–25.00 min, isocratic to 95% B; then 5 min for column re-equilibration. Data acquisition was set in the range of 190–800 nm, and chromatograms were monitored at 254 nm.

#### General Procedure A: Reductive Amination (**1**, **13**)

To a solution of 5-NO_2_-indoline or deprotected intermediate **12** (0.1 mmol) in dichloromethane (DCM)/CH_3_COOH (5:1 v/v), 0.2 mmol of the proper aldehyde were added and the mixture was stirred at 60 °C. After 1.5 h, sodium triacetoxyborohydride (0.18 mmol) was added portionwise and the mixture was allowed to reflux for further 3–5 h. After cooling to room temperature, 20 mL of NaOH 1 N solution was added and the organic layer was separated. Then, it was dried over Na_2_SO_4_, filtered, and concentrated. Flash chromatography on silica gel using different mixtures of *n*-hexane/ethyl acetate as mobile phase furnished intermediates **1** and **13**.

#### General Procedure B: Continuous Flow Hydrogenation (**2**, **11**, **21**–**26**, **35**, **47**)

The reduction of the nitro group was performed by continuous flow hydrogenation using the H-Cube hydrogenator and commercially available Pd/C 10% cartridges as catalyst. A 0.1 M solution of the proper nitro intermediate in a mixture of tetrahydrofuran (THF)/CH_3_OH (1:1, v/v) was pumped at a flow rate of 1.0 mL/min. The temperature was set at 30 °C, while the hydrogen inlet pressure was set at 10 bar. After the completion of the reaction, the organic phase was concentrated *in vacuo* and the obtained compounds were used in the next step without further purification.

#### General Procedure C: Urea Formation (**5**–**8**, **12**, **27**–**33**, **36**, **40**, **45**, **48**)

To a solution of the proper amine intermediate (0.1 mmol) in dichloromethane, triphosgene (0.025 mmol) and triethylamine (0.12 mmol) were added and the mixture was stirred for 30 min at room temperature. After the addition of the second amine (1-adamantanemethylamine, 1-adamantylamine, 1-naphthylmethylamine, or 2-(4-methyl-1,4-diazepan-1-yl)ethanamine), the reaction was stirred for 1 h at room temperature. Then, water was added and the mixture was extracted with dichloromethane (3 × 50 mL); the organic layer was dried over Na_2_SO_4_, filtered, and evaporated. Flash chromatography using *n*-hexane/ethyl acetate as mobile phase furnished the corresponding urea compounds.

#### General Procedure D: Hydrolysis (**14**, **37**, **38**)

To a solution of the proper ester intermediate (1 mmol) dissolved in methanol, 5 mL of NaOH 1 M aqueous solution were added. The reaction mixture was stirred at 100 °C until the complete disappearance of the starting material, as evidenced by TLC. The aqueous phase was quenched with 20 mL of HCl 2 M, diluted with ethyl acetate, and extracted. Then, the organic layer was filtered, dried over anhydrous Na_2_SO_4_, and evaporated *in vacuo* affording the final derivatives without further purification.

#### General Procedure E: *N*-Alkylation (**15**–**20**, **41**, **46**)

5-Nitroindole or intermediate **40** or carbazole (0.1 mmol) was dissolved in *N*,*N*-dimethylformamide (DMF), and the mixture was cooled to 0 °C. To this solution, 0.2 mmol of NaH were added and the mixture was allowed to react for 30 min. Then, 0.2 mmol of the proper alkyl bromide were added dropwise and the reaction was stirred at room temperature for 12 h. Afterward, the reaction was quenched with 10% aqueous solution of citric acid and diluted with dichloromethane. The organic layer was separated, dried over anhydrous Na_2_SO_4_, filtered, and concentrated under vacuum. Flash chromatography of the crude mixture using *n*-hexane/ethyl acetate as mobile phase furnished the purified intermediates.

#### General Procedure F: Nitrile Reduction (**39**, **42**)

To a solution of intermediate **31** or **41** (0.5 mmol) in methanol, CoCl_2_ (0.5 mmol) and NaBH_4_ (2 mmol) were added. The temperature was set a 0 °C, and the reaction mixture was stirred for 2 h. Then, the organic phase was concentrated, added with water, and extracted with ethyl acetate (3 × 50 mL). The organic layer was separated, dried over anhydrous Na_2_SO_4_, filtered, and evaporated. Crude products were purified by flash chromatography using ethyl acetate/methanol as mobile phase, furnishing final derivatives **39** and **42**.

#### 1-(4-Fluorobenzyl)-5-nitroindoline (**1**)

Intermediate **1** was obtained from 5-nitroindoline and 4-fluorobenzaldehyde following procedure A. FC in hexane/ethyl acetate 8/2, *R_f_*: 0.48. Yellow oil (92% yield). ^1^H NMR (400 MHz, CDCl_3_): δ: 3.11 (t, 2H, C*H*_2_, *J* = 8.6 Hz); 3.63 (t, 2H, C*H*_2_, *J* = 8.9 Hz); 4.42 (s, 2H, C*H*_2_); 6.38 (d, 1H, aryl, *J* = 8.8 Hz); 7.07 (t, 2H, aryl, *J* = 8.6 Hz); 7.24–7.28 (m, 2H, aryl); 7.93 (s, 1H, aryl); 8.07 (d, 1H, aryl, *J* = 8.8 Hz). ESI-MS **m*/*z** calcd for C_15_H_13_FN_2_O_2_ [(M + H)]^+^: 273.1034; found 273.1039.

#### 1-(4-Fluorobenzyl)indolin-5-amine (**2**)

Intermediate **22** was obtained from **1** following general procedure B. FC in hexane/ethyl acetate 1/1, *R_f_*: 0.41. White solid (95% yield). ^1^H NMR (400 MHz, CDCl_3_): δ: 2.79 (t, 2H, C*H*_2_, *J* = 7.4 Hz); 3.08 (t, 2H, C*H*_2_, *J* = 7.6 Hz); 3.26 (bs, 2H, N*H*_2_); 4.01 (s, 2H, C*H*_2_); 6.26 (d, 1H, aryl, *J* = 8.1 Hz); 6.37 (d, 1H, aryl, *J* = 8.0 Hz); 6.50 (s, 1H, aryl); 6.93 (t, 2H, aryl, *J* = 8.7 Hz); 7.24–7.27 (m, 2H, aryl). ESI-MS **m*/*z** calcd for C_15_H_15_FN_2_ [(M + H)]^+^: 243.1292; found 243.1300.

#### Synthesis of (3*R*,5*R*,7*R*)-*N*-(1-(4-Fluorobenzyl)indolin-5-yl)adamantane-1-carboxamide (**3**)

To a solution of 1-adamantanecarboxylic acid (0.1 mmol) in DCM, 0.12 mmol of HOBt, 0.12 mmol of HBTU, and 0.24 mmol of diisopropylethylamine (DIPEA) were added and the mixture was stirred at room temperature for 30 min. Then, 0.1 mmol of intermediate **2** was added and the reaction was mixed at room temperature for further 12 h. Later, the crude was washed with water (3 × 50 mL), 10% aqueous solution of citric acid (3 × 50 mL), and saturated aqueous solution of NaHCO_3_ (3 × 50 mL). The organic phase was dried over anhydrous Na_2_SO_4_, filtered, and concentrated *in vacuo*. Flash chromatography using *n*-hexane/ethyl acetate (7/3, v/v) furnished the final derivative **3** as a white powder in 61% yield. *R_f_*: 0.48. ^1^H NMR (400 MHz, DMSO): δ: 1.70 (bs, 6H, C*H*_*2*_); 1.88 (bs, 6H, C*H*_*2*_); 2.01 (bs, 3H, C*H*); 2.85 (t, 2H, C*H*_*2*_, *J* = 8.3 Hz); 3.19 (t, 2H, C*H*_*2*_, *J* = 8.1 Hz); 4.21 (s, 2H, C*H*_*2*_); 6.53 (d, 1H, aryl, *J* = 8.4 Hz); 7.15–7.19 (m, 3H, aryl); 7.32 (s, 1H, aryl) 7.37–7.40 (m, 2H, aryl); 8.77 (s, 1H, N*H*). ^13^C NMR (100 MHz, DMSO) δ 28.2, 28.5, 36.6, 39.0, 52.8, 53.5, 107.1, 115.4, 115.6, 118.9, 120.4, 130.0, 130.5, 149.0, 160.6, 163.0, 175.7. HR-MS *m*/*z* calcd for C_26_H_29_FN_2_O [(M + H)]^+^: 405.2337; found 405.2343.

#### Synthesis of 1-((1*S*)-7,7-Dimethyl-2-oxobicyclo[2.2.1]heptan-1-yl)-*N*-(1-(4-fluorobenzyl)indolin-5-yl)methanesulfonamide (**4**)

Intermediate **2** (1.0 mmol) was dissolved in DCM, and 1.2 mmol of 1,8-diazabicyclo[5.4.0]undec-7-ene (DBU) and 1.2 mmol of (1*S*)-(+)-10-camphorsulfonyl chloride were added. The reaction mixture was stirred at room temperature for 2 h and then washed with a saturated solution of NaHCO_3_ (3 × 50 mL). The organic phase was extracted, dried over anhydrous Na_2_SO_4_, filtered, and concentrated under vacuum. The crude product was purified by flash chromatography using dichloromethane/hexane (8/2, v/v) as mobile phase. *R_f_*: 0.45. Yellowish oil (35% yield). ^1^H NMR (400 MHz, CDCl_3_): δ: 0.83 (s, 3H, C*H*_3_); 0.90 (s, 3H, C*H*_3_); 1.38–1.43 (m, 2H, C*H*_2_); 1.96–2.04 (m, 2H, C*H*_2_); 2.06–2.12 (m, 2H, C*H*_2_); 2.40 (dd, 1H, C*H*, *J′* = 2.3; *J″* = 18.6 Hz); 2.72 (d, 1H, C*H*_*2a*_, *J* = 15.3 Hz); 2.89 (t, 2H, C*H*_*2*_, *J* = 8.2 Hz); 3.22–3.27 (m, 2H, C*H*_*2*_); 3.35 (d, 1H, C*H*_*2b*_, *J* = 15.2 Hz); 4.12 (s, 2H, C*H*_*2*_); 6.32 (d, 1H, aryl, *J* = 8.3 Hz); 6.84 (d, 1H, aryl, *J* = 10.2 Hz); 6.95 (t, 2H, aryl, *J* = 8.6 Hz); 7.01 (s, 1H, aryl); 7.23–7.26 (m, 2H, aryl); 7.35 (s, 1H, N*H*). ^13^C NMR (100 MHz, CDCl_3_) δ 19.4, 20.0, 27.1, 27.8, 28.4, 29.7, 42.9, 43.1, 48.3, 49.1, 53.7, 59.9, 115.3, 115.6, 121.6, 123.3, 129.7, 161.0, 163.4, 217.6. HR-MS *m*/*z* calcd for C_25_H_29_N_2_FO_3_S [(M + H)]^+^: 457.1956; found 457.1903.

#### 1-(((3*R*,5*R*,7*R*)-Adamantan-1-yl)methyl)-3-(1-(4-fluorobenzyl)indolin-5-yl)urea (**5**)

Derivative **5** was synthesized starting from **2** and 1-adamantanemethylamine following the general procedure C. FC in hexane/ethyl acetate 1/1, *R_f_*: 0.47. White powder (38% yield). ^1^H NMR (400 MHz, DMSO): δ: 1.45 (bs, 6H, C*H*_*2*_); 1.59 (d, 3H, C*H*_*2*_ and C*H*_*2a*_, *J* = 12.1 Hz); 1.68 (d, 3H, C*H*_*2*_ and C*H*_*2b*_, *J* = 12.0 Hz); 1.94 (bs, 3H, C*H*); 2.77 (d, 2H, C*H*_*2*_, *J* = 5.8 Hz); 2.83 (t, 2H, C*H*_*2*_, *J* = 8.0 Hz); 3.14 (t, 2H, C*H*_*2*_, *J* = 8.0 Hz); 4.16 (s, 2H, C*H*_*2*_); 5.90 (t, 1H, N*H*, *J* = 6.1 Hz); 6.47 (d, 1H, aryl, *J* = 8.4 Hz); 6.91 (d, 1H, aryl, *J* = 8.3 Hz); 7.14–7.18 (m, 3H, aryl); 7.37–7.41 (m, 2H, aryl); 7.95 (s, 1H, N*H*). ^13^C NMR (100 MHz, DMSO) δ 28.2, 28.7, 34.0, 37.1, 51.3, 53.3, 53.8, 107.7, 115.4, 116.5, 117.6, 130.4, 130.6, 132.2, 135.1, 147.6, 156.3, 160.5, 162.9. HR-MS *m*/*z* calcd for C_27_H_32_FN_3_O [(M + H)]^+^: 434.2602; found 434.2566.

#### 1-((3*S*,5*S*,7*S*)-Adamantan-1-yl)-3-(1-(4-fluorobenzyl)indolin-5-yl)urea (**6**)

Obtained from **2** and 1-adamantylamine following the general procedure C. FC in hexane/ethyl acetate 9.8/0.2, *R_f_*: 0.47. Yellowish powder (42% yield). ^1^H NMR (400 MHz, DMSO): δ: 1.63 (bs, 6H, C*H*_*2*_); 1.92 (bs, 6H, C*H*_*2*_); 2.02 (bs, 3H, C*H*); 2.82 (t, 2H, C*H*_*2*_, *J* = 8.2 Hz); 3.13 (t, 2H, C*H*_*2*_, *J* = 8.0 Hz); 4.15 (s, 2H, C*H*_*2*_); 5.66 (s, 1H, N*H*); 6.46 (d, 1H, aryl, *J* = 8.3 Hz); 6.86 (d, 1H, aryl, *J* = 8.9 Hz); 7.14–7.18 (m, 2H, aryl); 7.37–7.40 (m, 3H, aryl); 7.82 (bs, 1H, N*H*). ^13^C NMR (100 MHz, DMSO) δ 28.7, 29.4, 36.6, 42.3, 50.0, 53.3, 53.8, 107.7, 115.4, 115.6, 116.5, 117.5, 130.4, 130.5, 132.2, 135.1, 147.5, 154.9, 160.5, 162.9. HR-MS *m*/*z* calcd for C_26_H_30_FN_3_O [(M + H)]^+^: 421.2416; found 421.2484.

#### 1-(1-(4-Fluorobenzyl)indolin-5-yl)-3-(naphthalen-1-ylmethyl)urea (**7**)

Obtained from **2** and 1-naphthylmethylamine following the general procedure C. FC in hexane/ethyl acetate 9.8/0.2, *R_f_*: 0.45. Yellowish powder (48% yield). ^1^H NMR (400 MHz, DMSO): δ: 2.84 (t, 2H, C*H*_*2*_, *J* = 7.8 Hz); 2.85 (t, 2H, C*H*_*2*_, *J* = 8.0 Hz); 4.17 (s, 2H, C*H*_*2*_); 5.97 (t, 1H, N*H*, *J* = 5.7 Hz); 6.44 (d, 1H, aryl, *J* = 8.4 Hz); 6.90 (d, 1H, aryl, *J* = 7.8 Hz); 7.18 (s, 1H, aryl); 7.48 (d, 2H, aryl, *J* = 8.2 Hz); 7.92 (d, 2H, aryl, *J* = 8.2 Hz); 8.05 (s, 1H, N*H*). ^13^C NMR (100 MHz, DMSO) δ 28.7, 41.3, 53.2, 53.8, 107.7, 115.4, 115.6, 116.9, 118.0, 124.0, 125.8, 126.0, 126.3, 126.7, 127.9, 129.0, 130.40, 130.48, 130.56, 131.4, 131.8, 133.8, 135.1, 136.2, 147.8, 155.9, 162.9. HR-MS *m*/*z* calcd for C_27_H_24_N_3_FO_3_ [(M + H)]^+^: 426.1976; found 426.1979.

#### 1-(1-(4-Fluorobenzyl)indolin-5-yl)-3-(2-(4-methyl-1,4-diazepan-1-yl)ethyl)urea (**8**)

Obtained from **2** and 2-(4-methyl-1,4-diazepan-1-yl)ethanamine following the general procedure C. FC in dichloromethane/methanol 7/3, *R_f_*: 0.41. Yellow oil (45% yield). ^1^H NMR (400 MHz, CD_3_OD): δ: 1.83–1.89 (m, 2H, C*H*_*2*_); 2.41 (s, 3H, C*H*_*3*_); 2.65 (t, 2H, C*H*_*2*_, *J* = 6.3 Hz); 2.72–2.81 (m, 8H, C*H*_*2*_); 2.92 (t, 2H, C*H*_*2*_, *J* = 8.2 Hz); 3.23–3.29 (m, 4H, C*H*_*2*_); 4.20 (s, 2H, C*H*_*2*_); 6.51 (d, 1H, aryl, *J* = 8.3 Hz); 6.93 (d, 1H, aryl, *J* = 8.4 Hz); 7.05–7.09 (m, 3H, aryl); 7.38–7.42 (m, 2H, aryl). ^13^C NMR (100 MHz, CD_3_OD) δ 26.1, 28.1, 37.3, 45.3, 52.6, 53.1, 53.6, 53.8, 56.2, 57.3, 107.2, 114.6, 114.8, 119.2, 120.9, 129.5, 129.7, 130.9, 134.4, 149.2, 158.1, 163.3. HR-MS *m*/*z* calcd for C_24_H_32_FN_5_O [(M + H)]^+^: 426.2664; found 426.2666.

#### Synthesis of 1-(4-Fluorobenzyl)-5-thiocyanatoindoline (**9**)

Intermediate **2** (0.1 mmol) was dissolved in toluene, and 0.1 mmol of triethylamine and 0.2 mmol of carbon disulfide were added. The reaction mixture was stirred at room temperature for 12 h; then, the organic phase was concentrated under vacuum, dissolved in dichloromethane, 0.1 mmol of TEA and 0.1 mmol of ethyl chloroformate were added, and the mixture was stirred for further 12 h at room temperature. The organic layer was washed with a saturated solution of NaHCO_3_ (3 × 50 mL), extracted, dried over anhydrous Na_2_SO_4_, filtered, and concentrated under vacuum. Flash chromatography using *n*-hexane/ethyl acetate (9/1, v/v) as eluent furnished isothiocyanate intermediate **9** as a whitish oil in 58% yield. *R_f_*: 0.48. ^1^H NMR (400 MHz, CDCl_3_): δ: 2.99 (t, 2H, C*H*_2_, *J* = 8.4 Hz); 3.40 (t, 2H, C*H*_2_, *J* = 8.5 Hz); 4.26 (s, 2H, C*H*_2_); 6.37 (d, 1H, aryl, *J* = 8.3 Hz); 6.94 (s, 1H, aryl); 6.97 (d, 1H, aryl, *J* = 5.5 Hz); 7.05 (t, 2H, aryl, *J* = 8.6 Hz); 7.28–7.32 (m, 2H, aryl). ESI-MS *m*/*z* calcd for C_16_H_13_FN_2_S [(M + H)]^+^: 285.0856; found 285.0849.

#### Synthesis of 1-(((3*R*,5*R*,7*R*)-Adamantan-1-yl)methyl)-3-(1-(4-fluorobenzyl)indolin-5-yl)thiourea (**10**)

Isothiocyanate **9** (0.1 mmol) was dissolved in dichloromethane, 0.15 mmol of triethylamine and 0.15 mmol of 1-adamantylamine were added, and the mixture was stirred at room temperature for 30 min. Then, the organic phase was treated with an aqueous solution (10% w/w) of NaHCO_3_ (3 × 100 mL) and subsequently with HCl 2 M (3 × 100 mL). The organic layer was dried on Na_2_SO_4_, filtered, and concentrated *in vacuo*. Crude product was purified by flash chromatography using *n*-hexane/ethyl acetate (9.8/0.2, v/v) as eluent. *R_f_*: 0.43. Yellow oil (38% yield). ^1^H NMR (400 MHz, CDCl_3_): δ: 1.39 (bs, 6H, C*H*_*2*_); 1.52 (d, 3H, C*H*_*2*_ and C*H*_*2a*_, *J* = 11.6 Hz); 1.63 (d, 3H, C*H*_*2*_ and C*H*_*2b*_, *J* = 12.1 Hz); 1.89 (bs, 3H, C*H*); 2.91 (t, 2H, C*H*_*2*_, *J* = 8.4 Hz); 3.29–3.34 (m, 4H, C*H*_*2*_); 4.18 (s, 2H, C*H*_*2*_); 5.89 (bs, 1H, N*H*); 6.40 (d, 1H, aryl, *J* = 6.6 Hz); 6.84 (d, 1H, aryl, *J* = 8.2 Hz); 6.88 (s, 1H, aryl); 6.96 (t, 2H, aryl, *J* = 8.6 Hz); 7.20–7.25 (m, 3H, aryl); 7.43 (s, 1H, N*H*). ^13^C NMR (100 MHz, CDCl_3_) δ 28.18, 28.21, 34.2, 36.9, 40.5, 52.6, 53.4, 56.8, 107.2, 115.4, 115.6, 123.3, 126.1, 129.4, 132.0, 161.0, 163.4, 181.9. HR-MS *m*/*z* calcd for C_27_H_32_FN_3_S [(M + H)]^+^: 450.2374; found 450.2333.

#### *tert*-Butyl 5-Nitroindoline-1-carboxylate (**11**)

Intermediate **11** was obtained from 5-nitroindoline and di-*tert*-butyl dicarbonate following procedure B. FC in hexane/ethyl acetate 8/2, *R_f_*: 0.45. Yellow solid (88% yield). ^1^H NMR (400 MHz, CDCl_3_): δ: 1.60 (s, 9H, C*H*_*3*_); 3.19 (t, 2H, C*H*_2_, *J* = 8.7 Hz); 4.11 (t, 2H, C*H*_2_, *J* = 9.0 Hz); 7.28 (d, 1H, aryl, *J* = 7.7 Hz); 8.02 (s, 1H, aryl); 8.12 (d, 1H, aryl, *J* = 11.0 Hz). ESI-MS **m*/*z** calcd for C_13_H_16_N_2_O_4_ [(M + H)]^+^: 265.1183; found 265.1190.

#### *tert*-Butyl 5-(3-(((3*R*,5*R*,7*R*)-Adamantan-1-yl)methyl)ureido)indoline-1-carboxylate (**12**)

Intermediate **12** was obtained starting from **11** and 1-adamantanemethylamine following the general procedure C. FC in hexane/ethyl acetate 3/7, *R_f_*: 0.48. Yellow oil (45% yield). ^1^H NMR (400 MHz, CDCl_3_): δ: 1.49 (s, 9H, C*H*_*3*_); 1.57 (bs, 6H, C*H*_*2*_); 1.63 (d, 3H, C*H*_*2*_ and C*H*_*2a*_, *J* = 13.0 Hz); 1.72 (d, 3H, C*H*_*2*_ and C*H*_*2b*_, *J* = 12.0 Hz); 1.98 (bs, 3H, C*H*); 2.87 (s, 2H, C*H*_*2*_); 3.05 (t, 2H, C*H*_*2*_, *J* = 8.6 Hz); 3.97 (t, 2H, C*H*_*2*_, *J* = 7.7 Hz); 5.17 (bs, 1H, N*H*); 6.97 (bs, 2H, aryl); 7.25 (bs, 1H, aryl); 7.74 (bs, 1H, N*H*). HR-MS *m*/*z* calcd for C_25_H_35_N_3_O_3_ [(M + H)]^+^: 426.2751; found 426.2745

#### 4-((5-(3-(((3*r*,5*r*,7*r*)-Adamantan-1-yl)methyl)ureido)indolin-1-yl)methyl)benzoate (**13**)

Intermediate **13** was obtained from **12** following the general procedure A. FC in hexane/ethyl acetate 3/7, *R_f_*: 0.46. Yellowish oil (55% yield). ^1^H NMR (400 MHz, CD_3_OD): δ: 1.52 (bs, 6H, C*H*_*2*_); 1.68 (d, 3H, C*H*_*2*_ and C*H*_*2a*_, *J* = 11.6 Hz); 1.78 (d, 3H, C*H*_*2*_ and C*H*_*2b*_, *J* = 12.1 Hz); 1.97 (bs, 3H, C*H*); 2.83 (s, 2H, C*H*_*2*_); 2.95 (t, 2H, C*H*_*2*_, *J* = 7.2 Hz); 3.27 (t, 2H, C*H*_*2*_, *J* = 7.6 Hz); 3.90 (s, 3H, C*H*_3_); 4.27 (s, 2H, C*H*_*2*_); 6.44 (d, 1H, aryl, *J* = 8.0 Hz); 6.92 (d, 1H, aryl, *J* = 8.1 Hz); 7.11 (s, 1H, aryl); 7.50 (d, 2H, aryl, *J* = 8.2 Hz); 7.99 (d, 2H, aryl, *J* = 7.8 Hz). HR-MS *m*/*z* calcd for C_29_H_35_N_3_O_3_ [(M + H)]^+^: 474.2751; found 474.2760.

#### 4-((5-(3-(((3*R*,5*R*,7*R*)-Adamantan-1-yl)methyl)ureido)indolin-1-yl)methyl)benzoic Acid (**14**)

Obtained from **13** following the general procedure D. Precipitated from MeOH/Et_2_O. Whitish powder (67% yield). ^1^H NMR (400 MHz, DMSO): δ: 1.44 (bs, 6H, C*H*_*2*_); 1.60 (d, 3H, C*H*_*2*_ and C*H*_*2a*_, *J* = 11.4 Hz); 1.67 (d, 3H, C*H*_*2*_ and C*H*_*2b*_, *J* = 12.0 Hz); 1.94 (bs, 3H, C*H*); 2.76 (d, 2H, C*H*_*2*_, *J* = 5.9 Hz); 2.85 (t, 2H, C*H*_*2*_, *J* = 8.0 Hz); 3.19 (t, 2H, C*H*_*2*_, *J* = 8.0 Hz); 4.25 (s, 2H, C*H*_*2*_); 5.97 (t, 1H, N*H*, *J* = 5.7 Hz); 6.44 (d, 1H, aryl, *J* = 8.4 Hz); 6.90 (d, 1H, aryl, *J* = 7.8 Hz); 7.18 (s, 1H, aryl); 7.48 (d, 2H, aryl, *J* = 8.2 Hz); 7.92 (d, 2H, aryl, *J* = 8.2 Hz); 8.05 (s, 1H, N*H*). ^13^C NMR (100 MHz, DMSO) δ 28.2, 28.8, 34.0, 37.1, 51.3, 54.1, 107.7, 116.4, 117.5, 128.5, 129.89, 129.95, 130.5, 132.4, 144.4, 147.5, 156.4, 167.7. HR-MS *m*/*z* calcd for C_28_H_33_N_3_O_3_ [(M – H)]^−^: 458.2449; found 458.2852.

#### 1-(4-Fluorobenzyl)-5-nitro-1*H*-indole (**15**)

Intermediate **15** was obtained from 5-nitroindole and 4-fluorobenzyl bromide following the procedure E. FC in hexane/ethyl acetate 8/2, *R_f_*: 0.47. Yellow oil (85% yield). ^1^H NMR (400 MHz, CDCl_3_): δ: 5.35 (s, 2H, C*H*_2_); 6.74 (d, 1H, aryl, *J* = 3.1 Hz); 7.02 (t, 2H, aryl, *J* = 8.1 Hz); 7.08–7.12 (m, 2H, aryl); 7.28–7.31 (m, 2H, aryl); 8.03 (s, 1H, aryl); 8.08 (d, 1H, aryl, *J* = 9.1 Hz); 8.60 (s, 1H, N*H*). ESI-MS **m*/*z** calcd for C_15_H_11_FN_2_O_2_ [(M + H)]^+^: 271.0877; found 271.0884.

#### 1-(4-Isopropylbenzyl)-5-nitro-1*H*-indole (**16**)

Intermediate **15** was synthesized starting from 5-nitroindole and 4-isopropylbenzyl bromide following procedure E. FC in hexane/ethyl acetate 9/1, *R_f_*: 0.45. Yellow oil (85% yield). ^1^H NMR (400 MHz, CDCl_3_): δ: 1.13 (d, 6H, C*H*_*3*_, *J* = 6.9 Hz); 2.74–2.84 (m, 1H, C*H*); 5.20 (s, 2H, C*H*_*2*_); 6.62 (d, 1H, aryl, *J* = 3.8 Hz); 6.95 (d, 2H, aryl, *J* = 8.2 Hz); 7.10 (d, 2H, aryl, *J* = 8.3 Hz); 7.18–7.24 (m, 2H, aryl); 7.99 (d, 1H, aryl, *J* = 9.2 Hz); 8.50 (s, 1H, aryl). HR-MS *m*/*z* calcd for C_18_H1_8_N_2_O_2_ [(M + H)]^+^: 295.1441; found 295.1438.

#### Methyl 4-((5-Nitro-1*H*-indol-1-yl)methyl)benzoate (**17**)

Intermediate **17** was synthesized starting from 5-nitroindole and methyl 4-(bromomethyl)benzoate following the procedure E. FC in hexane/ethyl acetate 1/1, *R_f_*: 0.47. Yellowish oil (72% yield). ^1^H NMR (400 MHz, CD_3_OD): δ: 3.76 (s, 3H, C*H*_*3*_); 5.46 (s, 2H, C*H*_*2*_); 6.69 (s, 1H, aryl); 7.14 (d, 2H, aryl, *J* = 8.2 Hz); 7.33 (d, 1H, aryl, *J* = 7.8 Hz); 7.45 (s, 1H, aryl); 7.85 (d, 2H, aryl, *J* = 8.0 Hz); 7.93 (d, 1H, aryl, *J* = 8.6 Hz); 8.48 (s, 1H, aryl). HR-MS *m*/*z* calcd for C_17_H_14_N_2_O_4_ [(M + H)]^+^: 311.1026; found 311.1028.

#### 1-(4-Fluorophenethyl)-5-nitro-1*H*-indole (**18**)

Intermediate **18** was synthesized starting from 5-nitroindole and methyl 4-fluorophenethyl bromide following the procedure E. FC in dichloromethane/hexane 9.8/0.2, *R_f_*: 0.46. Yellowish oil (62% yield). ^1^H NMR (400 MHz, CDCl_3_): δ: 3.04 (t, 2H, C*H*_*2*_, *J* = 6.9 Hz); 4.30 (t, 2H, C*H*_*2*_, *J* = 6.9 Hz); 6.54 (d, 1H, aryl, *J* = 3.8 Hz); 6.98 (s, 1H, aryl); 6.82–6.86 (m, 3H, aryl); 6.94 (d, 1H, aryl, *J* = 4.2 Hz); 7.15–7.19 (m, 2H, aryl); 8.00 (d, 1H, aryl, *J* = 11.3 Hz); 8.51 (s, 1H, aryl). HR-MS *m*/*z* calcd for C_16_H_13_FN_2_O_2_ [(M + H)]^+^: 285.1034; found 285.1026.

#### 3-(5-Nitro-1*H*-indol-1-yl)propanenitrile (**19**)

Intermediate **19** was synthesized starting from 5-nitroindole and methyl 3-bromopropionitrile following the procedure E. FC in hexane/ethyl acetate 3/7, *R_f_*: 0.45. Yellow oil (85% yield). ^1^H NMR (400 MHz, CDCl_3_): δ: 2.91 (t, 2H, C*H*_*2*_, *J* = 6.6 Hz); 4.54 (t, 2H, C*H*_*2*_, *J* = 6.6 Hz); 6.78 (d, 1H, aryl, *J* = 3.3 Hz); 7.28 (s, 1H, aryl); 7.35–7.40 (m, 2H, aryl); 8.18 (d, 1H, aryl, *J* = 9.1 Hz); 8.62 (s, 1H, aryl). HR-MS *m*/*z* calcd for C_11_H_9_N_2_O_2_ [(M + H)]^+^: 216.0868; found 216.0765.

#### Methyl 4-(5-Nitro-1*H*-indol-1-yl)butanoate (**20**)

Intermediate **20** was synthesized starting from 5-nitroindole and methyl 4-bromobutanoate following the procedure E. FC in dichloromethane/hexane 9/1, *R_f_*: 0.45. Orange oil (62% yield). ^1^H NMR (400 MHz, CD_3_OD): δ: 2.12–2.19 (m, 2H, C*H*_*2*_); 2.33 (t, 2H, C*H*_*2*_, *J* = 6.9 Hz); 3.84 (s, 3H, C*H*_3_); 4.32 (t, 2H, C*H*_*2*_, *J* = 6.9 Hz); 6.73 (d, 1H, aryl, *J* = 3.9 Hz); 7.47 (d, 1H, aryl, *J* = 3.8 Hz); 7.58 (d, 1H, aryl, *J* = 7.8 Hz); 8.57 (s, 1H, aryl). ^13^C NMR (100 MHz, CDCl_3_) δ 25.4, 28.3, 30.8, 34.1, 37.0, 40.2, 45.5, 51.7, 51.9, 101.5, 110.1, 117.4, 119.8, 129.0, 129.2, 129.8, 134.2, 137.2. HR-MS *m*/*z* calcd for C_13_H_14_N_2_O_4_ [(M + H)]^+^: 263.1026; found 263.1019.

#### 1-(4-Fluorobenzyl)-1*H*-indol-5-amine (**21**)

Intermediate **21** was obtained from **15** following procedure B. FC in hexane/ethyl acetate 7/3, *R_f_*: 0.52. Yellow oil (80% yield). ^1^H NMR (400 MHz, CDCl_3_): δ: 3.46 (bs, 2H, N*H*_*2*_); 5.24 (s, 2H, C*H*_2_); 6.40 (d, 1H, aryl, *J* = 3.0 Hz); 6.67 (dd, 1H, aryl, *J′* = 2.2, Hz *J″* = 6.4 Hz); 6.98–7.02 (m, 3H, aryl); 7.06–7.11 (m, 4H, aryl). ESI-MS **m*/*z** calcd for C_15_H_13_N_2_ [(M + H)]^+^: 241.1136; found 241.1129.

#### 1-(4-Isopropylbenzyl)-1*H*-indol-5-amine (**22**)

Intermediate **22** was obtained from **16** following procedure B. FC in hexane/ethyl acetate 3/7, *R_f_*: 0.48. Whitish oil (65% yield). ^1^H NMR (400 MHz, CDCl_3_): δ: 1.28 (d, 6H, C*H*_*3*_, *J* = 6.9 Hz); 2.89–2.97 (m, 1H, C*H*); 3.49 (bs, 2H, N*H*_2_); 5.26 (s, 2H, C*H*_*2*_); 6.41 (s, 1H, aryl); 6.69 (d, 2H, aryl, *J* = 8.7 Hz); 7.00 (s, 1H, aryl); 7.09–7.21 (m, 5, aryl). HR-MS *m*/*z* calcd for C_18_H_20_N_2_ [(M + H)]^+^: 265.1699; found 265.1705.

#### Methyl 4-((5-Amino-1*H*-indol-1-yl)methyl)benzoate (**23**)

Intermediate **23** was obtained from **17** following procedure B. FC in dichloromethane/ethyl acetate 8/2, *R_f_*: 0.48. Yellowish powder (65% yield). ^1^H NMR (400 MHz, CD_3_OD): δ: 3.88 (s, 3H, C*H*_*3*_); 5.40 (s, 2H, C*H*_*2*_); 6.34 (d, 1H, aryl, *J* = 2.9 Hz); 6.68 (d, 1H, aryl, *J* = 10.5 Hz); 7.00 (s, 1H, aryl); 7.05 (d, 1H, aryl, *J* = 8.6 Hz); 7.17–7.20 (m, 3H, aryl); 7.93 (d, 2H, aryl, *J* = 8.3 Hz). HR-MS *m*/*z* calcd for C_17_H_16_N_2_O_2_ [(M + H)]^+^: 281.1285; found 281.1290.

#### 1-(4-Fluorophenethyl)-1*H*-indol-5-amine (**24**)

Intermediate **24** was obtained from **18** following procedure B. FC in dichloromethane/ethyl acetate 1/1, *R_f_*: 0.50. Yellowish solid (55% yield). ^1^H NMR (400 MHz, CDCl_3_): δ: 2.93 (t, 2H, C*H*_*2*_, *J* = 7.2 Hz); 3.44 (bs, 2H, N*H*_2_); 4.13 (t, 2H, C*H*_*2*_, *J* = 7.2 Hz); 6.14 (d, 1H, aryl, *J* = 2.9 Hz); 6.58 (dd, 1H, aryl, *J*′ = 2.2, *J″* = 8.6 Hz); 6.69 (d, 1H, aryl, *J* = 3.0 Hz); 6.80–6.89 (m, 5H, aryl); 7.01 (d, 1H, aryl,, *J* = 8.2 Hz). HR-MS *m*/*z* calcd for C_16_H_15_FN_2_ [(M + H)]^+^: 255.1292; found 255.1299.

#### 3-(5-Amino-1*H*-indol-1-yl)propanenitrile (**25**)

Intermediate **25** was obtained from **19** following procedure B. FC in dichloromethane/ethyl acetate 9.8/0.2, *R_f_*: 0.46. Yellowish powder (62% yield). ^1^H NMR (400 MHz, CDCl_3_): δ: 2.99 (t, 2H, C*H*_*2*_, *J* = 6.8 Hz); 4.57 (t, 2H, C*H*_*2*_, *J* = 6.8 Hz); 5.15 (bs, 2H, N*H*_2_); 6.60 (d, 1H, aryl, *J* = 3.2 Hz); 7.20 (dd, 1H, aryl, *J′* = 2.1, *J″* = 8.7 Hz); 7.47 (d, 1H, aryl, *J* = 3.1 Hz); 7.63–7.68 (m, 2H, aryl). HR-MS *m*/*z* calcd for C_11_H_11_N_3_ [(M + H)]^+^: 186.1026; found 186.1023.

#### Methyl 4-(5-Amino-1*H*-indol-1-yl)butanoate (**26**)

Intermediate **26** was obtained from **20** following procedure B. FC in hexane/ethyl acetate 1/1, *R_f_*: 0.48. Withish oil (77% yield). ^1^H NMR (400 MHz, CD_3_OD): δ: 2.02–2.09 (m, 2H, C*H*_*2*_); 2.23 (t, 2H, C*H*_*2*_, *J* = 7.0 Hz); 3.79 (s, 3H, C*H*_3_); 4.11 (t, 2H, C*H*_*2*_, *J* = 6.8 Hz); 6.24 (d, 1H, aryl, *J* = 3.2 Hz); 6.73 (d, 1H, aryl, *J* = 8.6 Hz); 6.96 (s, 1H, aryl); 7.04 (d, 1H, aryl, *J* = 2.8 Hz); 7.19 (d, 1H, aryl, *J* = 8.6 Hz). HR-MS *m*/*z* calcd for C_13_H_16_N_2_O_2_ [(M + H)]^+^: 233.1285; found 233.1291.

#### 1-(((3*R*,5*R*,7*R*)-Adamantan-1-yl)methyl)-3-(1-(4-fluorobenzyl)-1*H*-indol-5-yl)urea (**27**)

Obtained from **21** and 1-adamantanemethylamine following the general procedure C. FC in hexane/ethyl acetate 1/1, *R_f_*: 0.48. White powder (68% yield). ^1^H NMR (400 MHz, CDCl_3_): δ: 1.45 (bs, 6H, C*H*_*2*_); 1.60–1.63 (m, 3H, C*H*_*2*_ and C*H*_*2a*_); 1.70 (d, 3H, C*H*_*2*_ and C*H*_*2b*_, *J* = 12.0 Hz); 1.97 (bs, 3H, C*H*); 2.94 (d, 2H, C*H*_*2*_, *J* = 6.3 Hz); 3.51 (d, 1H, N*H*, *J* = 4.5 Hz); 4.77 (t, 1H, N*H*, *J* = 6.0 Hz); 5.31 (s, 2H, C*H*_*2*_); 6.17 (s, 1H, aryl); 6.55 (s, 1H, aryl); 7.01 (t, 1H, aryl, *J* = 8.7 Hz); 7.06–7.12 (m, 2H, aryl); 7.18 (d, 1H, aryl, *J* = 3.1 Hz); 7.25 (d, 1H, aryl, *J* = 8.6 Hz); 7.56 (s, 1H, aryl). ^13^C NMR (100 MHz, CDCl_3_) δ 28.2, 34.0, 37.0, 40.2, 49.7, 51.9, 102.0, 110.5, 115.7, 115.9, 117.7, 120.1, 128.4, 129.4, 130.0, 132.9, 134.0, 157.6, 163.5. HR-MS *m*/*z* calcd for C_27_H_30_FN_3_O [(M + H)]^+^: 432.2446; found 432.2430.

#### 1-(((3*R*,5*R*,7*R*)-Adamantan-1-yl)methyl)-3-(1-(4-isopropylbenzyl)-1*H*-indol-5-yl)urea (**28**)

Obtained from **22** and 1-adamantanemethylamine following the general procedure C. FC in dichloromethane/ethyl acetate 8/2, *R_f_*: 0.46. Yellowish powder (58% yield). ^1^H NMR (400 MHz, CDCl_3_): δ: 1.24 (d, 6H, C*H*_*3*_, *J* = 6.9 Hz); 1.45 (bs, 6H, C*H*_*2*_); 1.62 (d, 3H, C*H*_*2*_ and C*H*_*2a*_, *J* = 11.6 Hz); 1.71 (d, 3H, C*H*_*2*_ and C*H*_*2b*_, *J* = 12.1 Hz); 1.97 (bs, 3H, C*H*); 2.86–2.91 (m, 1H, C*H*); 2.94 (s, 2H, C*H*_*2*_); 4.82 (bs, 1H, N*H*); 5.31 (s, 2H, C*H*_*2*_); 6.24 (bs, 1H, N*H*); 6.54 (s, 1H, aryl); 7.07 (d, 3H, aryl, *J* = 12.1 Hz); 7.19 (d, 3H, aryl, *J* = 8.3 Hz); 7.29 (d, 1H, aryl, *J* = 4.7 Hz); 7.55 (s, 1H, aryl). ^13^C NMR (100 MHz, CDCl_3_) δ 23.9, 28.3, 33.8, 34.0, 37.0, 40.2, 50.1, 52.9, 101.7, 110.6, 117.7, 120.0, 126.8, 129.3, 129.57, 129.69, 134.47, 134.67, 148.6, 157.7. HR-MS *m*/*z* calcd for C_30_H_37_N_3_O [(M + H)]^+^: 456.3009; found 456.2971.

#### Methyl 4-((5-(3-(((3*R*,5*R*,7*R*)-Adamantan-1-yl)methyl)ureido)-1*H*-indol-1-yl)methyl)benzoate (**29**)

Obtained from **23** and 1-adamantanemethylamine following the general procedure C. FC in hexane/ethyl acetate 1/1, *R_f_*: 0.48. Yellow oil (39% yield). ^1^H NMR (400 MHz, CDCl_3_): δ: 1.45 (bs, 6H, C*H*_*2*_); 1.60 (d, 3H, C*H*_*2*_ and C*H*_*2a*_, *J* = 11.6 Hz); 1.69–1.73 (m, 3H, C*H*_*2*_ and C*H*_*2b*_); 1.95 (bs, 3H, C*H*); 2.94 (d, 2H, C*H*_*2*_, *J* = 6.3 Hz); 3.91 (s, 2H, C*H*_*3*_); 4.92 (t, 1H, N*H*, *J* = 6.2 Hz); 5.38 (s, 2H, C*H*_*2*_); 6.44 (s, 1H, aryl); 6.56 (s, 1H, aryl); 7.08 (d, 1H, aryl, *J* = 10.5 Hz); 7.14–7.19 (m, 3H, aryl); 7.59 (s, 1H, aryl); 7.98 (d, 2H, aryl, *J* = 8.3 Hz). ^13^C NMR (100 MHz, CDCl_3_) δ 28.3, 34.0, 37.0, 40.2, 50.1, 51.9, 52.2, 102.2, 110.3, 116.9, 119.7, 126.6, 129.4, 129.7, 130.1, 130.4, 134.3, 142.4, 157.6, 166.6. HR-MS *m*/*z* calcd for C_29_H_33_N_3_O_3_ [(M + H)]^+^: 472.2595; found 472.2554.

#### 1-(((3*R*,5*R*,7*R*)-Adamantan-1-yl)methyl)-3-(1-(4-fluorophenethyl)-1*H*-indol-5-yl)urea (**30**)

Obtained from **24** and 1-adamantanemethylamine following the general procedure C. FC in dichloromethane/ethyl acetate 7/3, *R_f_*: 0.48. Yellowish oil (41% yield). ^1^H NMR (400 MHz, CD_3_OD): δ: 1.57 (bs, 6H, C*H*_*2*_); 1.70 (d, 3H, C*H*_*2*_ and C*H*_*2a*_, *J* = 11.5 Hz); 1.79 (d, 3H, C*H*_*2*_ and C*H*_*2b*_, *J* = 12.1 Hz); 2.00 (bs, 3H, C*H*); 2.92 (s, 2H, C*H*_*2*_); 3.08 (t, 2H, C*H*_*2*_, *J* = 6.9 Hz); 4.36 (t, 2H, C*H*_*2*_, *J* = 6.9 Hz); 6.91 (t, 2H, aryl, *J* = 8.8 Hz); 6.98 (s, 1H, aryl); 7.01–7.07 (m, 3H, aryl); 7.26 (d, 2H, aryl, *J* = 8.7 Hz); 7.53 (s, 1H, aryl). ^13^C NMR (100 MHz, CD_3_OD) δ 28.4, 33.7, 35.4, 36.7, 39.9, 51.3, 109.2, 112.8, 114.5, 114.7, 116.3, 128.4, 128.9, 130.2, 130.9, 133.1, 134.8, 158.4, 160.5, 162.9. HR-MS *m*/*z* calcd for C_28_H_32_FN_3_O [(M + H)]^+^: 446.2602; found 446.2575.

#### 1-(((3*R*,5*R*,7*R*)-Adamantan-1-yl)methyl)-3-(1-(2-cyanoethyl)-1*H*-indol-5-yl)urea (**31**)

Intermediate **31** was obtained from **25** and 1-adamantanemethylamine following the general procedure C. FC in hexane/ethyl acetate 7/3, *R_f_*: 0.44. Yellowish oil (65% yield). ^1^H NMR (400 MHz, CDCl_3_): δ: 1.37 (bs, 6H, C*H*_*2*_); 1.53 (d, 3H, C*H*_*2*_ and C*H*_*2a*_, *J* = 11.6 Hz); 1.64 (d, 3H, C*H*_*2*_ and C*H*_*2b*_, *J* = 12.1 Hz); 1.89 (bs, 3H, C*H*); 2.77 (t, 2H, C*H*_*2*_, *J* = 7.0 Hz); 2.87 (s, 2H, C*H*_*2*_); 4.38 (t, 2H, C*H*_*2*_, *J* = 6.7 Hz); 6.47 (d, 1H, aryl, *J* = 2.4 Hz); 7.08 (d, 1H, aryl, *J* = 8.5 Hz); 7.13 (d, 1H, aryl, *J* = 2.6 Hz); 7.23 (d, 1H, aryl, *J* = 8.4 Hz); 7.47 (s, 1H, aryl). HR-MS *m*/*z* calcd for C_23_H_28_N_4_O [(M + H)]^+^: 377.2336; found 377.2340.

#### Methyl 4-(5-(3-(((3*R*,5*R*,7*R*)-Adamantan-1-yl)methyl)ureido)-1*H*-indol-1-yl)butanoate (**32**)

Obtained from **26** and 1-adamantanemethylamine following the general procedure C. FC in hexane/ethyl acetate 3/7, *R_f_*: 0.49. Withish oil (42% yield). ^1^H NMR (400 MHz, CDCl_3_): δ: 1.37 (bs, 6H, C*H*_*2*_); 1.53 (d, 3H, C*H*_*2*_ and C*H*_*2a*_, *J* = 11.6 Hz); 1.62 (d, 3H, C*H*_*2*_ and C*H*_*2b*_, *J* = 12.1 Hz); 1.87 (bs, 3H, C*H*); 2.05–2.12 (m, 2H, C*H*_*2*_); 2.23 (t, 2H, C*H*_*2*_, *J* = 6.7 Hz); 2.85 (s, 2H, C*H*_*2*_); 3.60 (s, 3H, C*H*_*3*_); 4.12 (t, 2H, C*H*_*2*_, *J* = 6.9 Hz); 6.36–6.40 (m, 2H, aryl and N*H*); 7.02–7.04 (m, 2H, aryl); 7.25 (d, 1H, aryl, *J* = 8.6 Hz); 7.45 (s, 1H, aryl). ^13^C NMR (100 MHz, CDCl_3_) δ 25.4, 28.3, 30.8, 34.1, 37.0, 40.2, 45.5, 51.7, 51.9, 101.5, 110.1, 117.4, 119.8, 129.0, 129.2, 129.8, 134.2, 137.2. HR-MS *m*/*z* calcd for C_25_H_33_N_3_O_3_ [(M + H)]^+^: 424.2595; found 424.2556.

#### 1-((3*S*,5*S*,7*S*)-Adamantan-1-yl)-3-(1-(4-fluorobenzyl)-1*H*-indol-5-yl)urea (**33**)

Obtained from **21** and 1-adamantanamine following the general procedure C. FC in hexane/ethyl acetate 1/1, *R_f_*: 0.51. Yellow oil (45% yield). ^1^H NMR (400 MHz, CD_3_OD): δ: 1.62 (bs, 10H, C*H*_*2*_); 1.93–1.96 (m, 15H, C*H*_*2*_ and C*H*); 5.22 (s, 2H, C*H*_*2*_); 6.31 (d, 1H, aryl, *J* = 4.6 Hz); 6.89 (t, 3H, aryl, *J* = 10.4 Hz); 7.02–7.05 (m, 2H, aryl); 7.09 (d, 1H, aryl, *J* = 8.8 Hz); 7.13 (d, 1H, aryl, *J* = 3.1 Hz); 7.41 (s, 1H, aryl). ^13^C NMR (100 MHz, CD_3_OD) δ 29.3, 29.7, 36.2, 42.0, 100.9, 109.4, 112.3, 114.7, 116.3, 128.3, 128.7, 129.2, 131.4, 133.1, 134.2, 156.8, 160.9, 163.4. HR-MS *m*/*z* calcd for C_26_H_28_FN_3_O [(M + H)]^+^: 418.2289; found 418.2247.

#### Synthesis of (4-Fluorophenyl)(5-nitro-1*H*-indol-1-yl)methanone (**34**)

To a solution of 0.1 mmol of 5-nitroindole in dichloromethane/dimethylformamide (1/1, v/v) 4-fluorobenzoyl chloride (0.6 mmol) and triethylamine (0.6 mmol) were added. The mixture was stirred at room temperature for 30 min. Then, water was added and the organic phase was washed three times, dried on Na_2_SO_4_, filtered, and concentrated *in vacuo*. Intermediate **34** was purified by flash chromatography using hexane/ethyl acetate (8/2) as mobile phase. *R_f_*: 0.45. Yellowish oil (65% yield). ^1^H NMR (400 MHz, CDCl_3_): δ: 6.71 (d, 1H, aryl, *J* = 3.7 Hz); 7.19 (t, 2H, aryl, *J* = 8.7 Hz); 7.40 (d, 1H, aryl, *J* = 3.8 Hz); 7.73–7.76 (m, 2H, aryl); 8.21 (dd, 1H, aryl, *J′* = 2.2, *J″* = 9.1 Hz); 8.39 (d, 1H, aryl, *J* = 9.1 Hz); 8.47 (d, 1H, aryl, *J* = 2.2 Hz).

#### (5-Amino-1*H*-indol-1-yl)(4-fluorophenyl)methanone (**35**)

Intermediate **35** was synthesized starting from **34** following the general procedure B. FC in hexane/ethyl acetate 3/7, *R_f_*: 0.42. Yellowish solid (69% yield). ^1^H NMR (400 MHz, CDCl_3_): δ: 3.12 (bs, 2H, N*H*_2_); 6.39 (d, 1H, aryl, *J* = 3.7 Hz); 6.70 (dd, 1H, aryl, *J′* = 2.2, *J″* = 8.7 Hz); 6.79 (d, 1H, aryl, *J* = 2.2 Hz); 7.09–7.15 (m, 3H, aryl); 7.65–7.69 (m, 2H, aryl); 8.10 (d, 1H, aryl, *J* = 8.7 Hz). HR-MS *m*/*z* calcd for C_27_H_28_FN_3_O_2_ [(M + H)]^+^: 255.0928; found 255.0925.

#### 1-(((3*R*,5*R*,7*R*)-Adamantan-1-yl)methyl)-3-(1-(4-fluorobenzoyl)-1*H*-indol-5-yl)urea (**36**)

Obtained from **35** and 1-adamantanemethylamine following the general procedure C. FC in hexane/ethyl acetate 1/1, *R_f_*: 0.45. Withish oil (42% yield). ^1^H NMR (400 MHz, CDCl_3_): δ: 1.41 (bs, 6H, C*H*_*2*_); 1.53 (d, 3H, C*H*_*2*_ and C*H*_*2a*_, *J* = 11.8 Hz); 1.63 (d, 3H, C*H*_*2*_ and C*H*_*2b*_, *J* = 12.1 Hz); 1.89 (bs, 3H, C*H*); 2.89 (s, 2H, C*H*_*2*_); 6.48 (s, 1H, aryl); 7.06–7.19 (m, 4H, aryl); 7.62–7.68 (m, 3H, aryl); 8.19 (d, 1H, aryl, *J* = 8.8 Hz). ^13^C NMR (100 MHz, CDCl_3_) δ 28.2, 33.9, 37.0, 40.2, 52.0, 108.9, 114.1, 115.8, 116.0, 116.9, 119.6, 128.2, 130.5, 131.66, 131.79, 132.9, 134.7, 156.9, 163.6, 166.1, 167.3. HR-MS *m*/*z* calcd for C_27_H_28_FN_3_O_2_ [(M + H)]^+^: 446.2238; found 446.2192.

#### 4-((5-(3-(((3*R*,5*R*,7*R*)-Adamantan-1-yl)methyl)ureido)-1*H*-indol-1-yl)methyl)benzoic Acid (**37**)

Obtained from **29** following the general procedure D. Precipitated from MeOH/Et_2_O as a white powder, (62% yield). ^1^H NMR (400 MHz, CD_3_OD): δ: 1.55 (bs, 6H, C*H*_*2*_); 1.70 (d, 3H, C*H*_*2*_ and C*H*_*2a*_, *J* = 11.7 Hz); 1.79 (d, 3H, C*H*_*2*_ and C*H*_*2b*_, *J* = 11.7 Hz); 1.99 (bs, 3H, C*H*); 2.90 (s, 2H, C*H*_*2*_); 5.45 (s, 2H, C*H*_*2*_); 6.48 (s, 1H, aryl); 7.04 (d, 1H, aryl, *J* = 8.7 Hz); 7.18–7.22 (m, 3H, aryl); 7.29 (d, 1H, aryl, *J* = 3.1 Hz); 7.59 (s, 1H, aryl); 7.94 (d, 2H, aryl, *J* = 8.3 Hz). ^13^C NMR (100 MHz, CD_3_OD) δ 28.4, 36.7, 39.9, 49.2, 51.2, 101.1, 109.5, 112.7, 116.6, 126.3, 129.0, 129.2, 129.7, 131.4, 133.3, 143.6, 158.3, 168.1. HR-MS *m*/*z* calcd for C_28_H_31_N_3_O_3_ [(M – H)]^+^: 456.2293; found 456.2292.

#### 4-(5-(3-(((3*R*,5*R*,7*R*)-Adamantan-1-yl)methyl)ureido)-1*H*-indol-1-yl)butanoic Acid (**38**)

Obtained from **32** following the general procedure D. Precipitated from MeOH/Et_2_O as a white powder, (58% yield). ^1^H NMR (400 MHz, CD_3_OD): δ: 1.43 (bs, 6H, C*H*_*2*_); 1.57 (d, 3H, C*H*_*2*_ and C*H*_*2a*_, *J* = 11.6 Hz); 1.65 (d, 3H, C*H*_*2*_ and C*H*_*2b*_, *J* = 12.1 Hz); 1.87 (bs, 3H, C*H*); 1.94–1.99 (m, 2H, C*H*_*2*_); 2.13 (t, 2H, C*H*_*2*_, *J* = 7.0 Hz); 2.79 (s, 2H, C*H*_*2*_); 4.09 (t, 2H, C*H*_*2*_, *J* = 6.9 Hz); 6.96 (d, 1H, aryl, *J* = 8.7 Hz); 7.06 (s, 1H, aryl); 7.23 (d, 2H, aryl, *J* = 8.7 Hz); 7.43 (s, 1H, aryl). ^13^C NMR (100 MHz, CD_3_OD) δ 25.3, 28.4, 30.2, 33.6, 36.7, 39.9, 44.8, 51.2, 100.4, 109.2, 112.8, 116.4, 128.3, 128.9, 131.0, 133.2, 158.3, 175.2. HR-MS *m*/*z* calcd for C_24_H_31_N_3_O_3_ [(M – H)]^+^: 408.2293; found 408.2298.

#### 1-(((3*R*,5*R*,7*R*)-Adamantan-1-yl)methyl)-3-(1-(3-aminopropyl)-1*H*-indol-5-yl)urea (**39**)

Obtained from **31** following the general procedure F. FC in ethyl acetate/methanol 7/3, *R_f_*: 0.38. Yellowish solid (46% yield). ^1^H NMR (400 MHz, CD_3_OD): δ: 1.45 (bs, 6H, C*H*_*2*_); 1.58 (d, 3H, C*H*_*2*_ and C*H*_*2a*_, *J* = 11.6 Hz); 1.67 (d, 3H, C*H*_*2*_ and C*H*_*2b*_, *J* = 12.1 Hz); 1.88–1.95 (m, 5H, C*H* and C*H*_*2*_); 2.57 (t, 2H, C*H*_*2*_, *J* = 7.0 Hz); 2.79 (s, 2H, C*H*_*2*_); 4.12 (t, 2H, C*H*_*2*_, *J* = 6.8 Hz); 6.27 (s, 1H, aryl); 6.99 (d, 1H, aryl, *J* = 8.7 Hz); 7.09 (s, 1H, aryl); 7.23 (d, 1H, aryl, *J* = 8.7 Hz); 7.42 (s, 1H, aryl). ^13^C NMR (100 MHz, CD_3_OD) δ 28.4, 36.8, 38.1, 39.9, 43.3, 51.2, 100.5, 109.1, 112.6, 116.3, 128.3, 129.0, 131.2, 133.1, 158.3. HR-MS *m*/*z* calcd for C_23_H_32_N_4_O [(M + H)]^+^: 381.2649; found 381.2937.

#### 1-(((3*R*,5*R*,7*R*)-Adamantan-1-yl)methyl)-3-(1-(4-cyanobenzyl)-1*H*-indol-5-yl)urea (**40**)

Intermediate **40** was synthesized starting from 5-aminoindole and 1-adamantanemethylamine following the general procedure C. Precipitated from MeOH/Et_2_O as a whitish powder, (72% yield). ^1^H NMR (400 MHz, CDCl_3_): δ: 1.48 (bs, 6H, C*H*_*2*_); 1.55 (d, 3H, C*H*_*2*_ and C*H*_*2a*_, *J* = 11.1 Hz); 1.66 (d, 3H, C*H*_*2*_ and C*H*_*2b*_, *J* = 12.0 Hz); 1.94 (bs, 3H, C*H*); 2.89 (d, 2H, C*H*_*2*_, *J* = 4.3 Hz); 6.50 (s, 1H, aryl); 7.00 (d, 1H, aryl, *J* = 8.0 Hz); 7.22 (s, 1H, aryl); 7.35 (d, 1H, aryl, *J* = 8.5 Hz); 7.46 (s, 1H, aryl); 8.25 (s, 1H, N*H*). HR-MS *m*/*z* calcd for C_28_H_30_N_4_O [(M + H)]^+^: 439.2492; found 439.2487.

#### 1-(((3*R*,5*R*,7*R*)-Adamantan-1-yl)methyl)-3-(1-(4-cyanobenzyl)-1*H*-indol-5-yl)urea (**41**)

Intermediate **41** was synthesized starting from **40** and 4-(bromomethyl)benzonitrile following the general procedure E. FC in dichloromethane/ethyl acetate 8/2, *R_f_*: 0.44. Yellow oil (55% yield). ^1^H NMR (400 MHz, CDCl_3_): δ: 1.45 (bs, 6H, C*H*_*2*_); 1.58 (d, 3H, C*H*_*2*_ and C*H*_*2a*_, *J* = 11.4 Hz); 1.70 (d, 3H, C*H*_*2*_ and C*H*_*2b*_, *J* = 12.1 Hz); 1.94 (bs, 3H, C*H*); 2.93 (d, 2H, C*H*_*2*_, *J* = 4.5 Hz); 5.36 (s, 2H, C*H*_*2*_); 6.55 (s, 1H, aryl); 6.72 (s, 1H, aryl); 7.11–7.15 (m, 4H, aryl); 7.55–7.60 (m, 3H, aryl). HR-MS *m*/*z* calcd for C_28_H_30_N_4_O [(M + H)]^+^: 439.2492; found 439.2487.

#### 1-(((3*R*,5*R*,7*R*)-Adamantan-1-yl)methyl)-3-(1-(4-(aminomethyl)benzyl)-1*H*-indol-5-yl)urea (**42**)

Obtained from **41** following the general procedure F. FC in ethyl acetate/methanol 8/2, *R_f_*: 0.38. Yellow oil (48% yield). ^1^H NMR (400 MHz, CD_3_OD): δ: 1.56 (bs, 6H, C*H*_*2*_); 1.70 (d, 3H, C*H*_*2*_ and C*H*_*2a*_, *J* = 11.7 Hz); 1.78 (d, 3H, C*H*_*2*_ and C*H*_*2b*_, *J* = 12.1 Hz); 1.99 (bs, 3H, C*H*); 2.90 (s, 2H, C*H*_*2*_); 3.80 (s, 2H, C*H*_*2*_); 5.35 (s, 2H, C*H*_*2*_); 6.43 (s, 1H, aryl); 7.03 (d, 1H, aryl, *J* = 10.7 Hz); 7.11 (d, 2H, aryl, *J* = 8.0 Hz); 7.21 (d, 1H, aryl, *J* = 8.7 Hz); 7.26–7.29 (m, 3H, aryl); 7.56 (s, 1H, aryl). ^13^C NMR (100 MHz, CD_3_OD) δ 28.4, 29.3, 36.8, 39.9, 44.5, 49.3, 51.2, 100.7, 109.6, 112.6, 116.4, 126.8, 127.6, 128.9, 129.2, 131.2, 133.3, 137.3, 140.0, 158.3. HR-MS *m*/*z* calcd for C_28_H_34_N_4_O [(M + H)]^+^: 443.2805; found 443.2748.

#### Synthesis of 1-(4-Fluorophenyl)-5-nitro-1*H*-indole (**43**)

To a solution of 5-nitroindole (0.5 mmol) in dry DMF, under positive nitrogen flow, Cs_2_CO_3_ (1 mmol), CuI (0.07 mmol), and 4-fluoroiodobenzene (0.5 mmol) were added. The mixture was stirred at 120 °C for 12 h, then water was added, and the mixture was extracted with dichloromethane (2 × 50 mL), dried over Na_2_SO_4_, and concentrated *in vacuo*. The crude product was purified by flash chromatography using *n*-hexane/ethyl acetate (8/2, v/v) as eluent. *R_f_*: 0.44. Yellow powder (35% yield). ^1^H NMR (400 MHz, CD_3_OD): δ: 6.83 (d, 1H, aryl, *J* = 3.3 Hz); 7.23–7.30 (m, 2H, aryl); 7.43–7.59 (m, 3H, aryl); 7.86 (d, 1H, aryl, *J* = 8.6 Hz); 8.01 (dd, 1H, aryl, *J′* = 2.3, *J″* 9.1 Hz); 8.55 (s, 1H, aryl). HR-MS *m*/*z* calcd for C_14_H_9_FN_2_O_2_ [(M + H)]^+^: 257.0721; found 257.0725.

#### 1-(4-Fluorophenyl)-1*H*-indol-5-amine (**44**)

Intermediate **43** was dissolved in THF and Pd/C (0.01 mmol) was added. The mixture was stirred at 60 °C for 10 min. Then, NH_2_NH_2_. H_2_O (1.5 mmol) was added and the mixture was stirred at 60 °C for further 12 h. After cooling, ethyl acetate was added and the organic phase was washed with a saturated solution of NaHCO_3_ (3 × 50 mL), dried over anhydrous Na_2_SO_4_, filtered, concentrated, and purified by column chromatography using *n*-hexane/ethyl acetate (1/1) as mobile phase. *R_f_*: 0.45 (65% yield). ^1^H NMR (400 MHz, CD_3_OD): δ: 6.43 (d, 1H, aryl, *J* = 2.5 Hz); 6.70 (dd, 1H, aryl, *J′* = 2.4, *J″* 8.2 Hz); 6.99 (d, 1H, aryl, *J* = 2.5 Hz); 7.14–7.25 (m, 3H, aryl); 7.29–7.40 (m, 3H, aryl). HR-MS *m*/*z* calcd for C_14_H_11_FN_2_ [(M + H)]^+^: 227.0979; found 227.0984.

#### 1-(((3*R*,5*R*,7*R*)-Adamantan-1-yl)methyl)-3-(1-(4-fluorophenyl)-1*H*-indol-5-yl)urea (**45**)

Obtained from **44** following the general procedure C. FC in hexane/ethyl acetate 7/3, *R_f_*: 0.44. Yellow oil (32% yield). ^1^H NMR (400 MHz, CD_3_OD): δ: 1.45 (bs, 6H, C*H*_*2*_); 1.59 (d, 3H, C*H*_*2*_ and C*H*_*2a*_, *J* = 11.6 Hz); 1.66 (d, 3H, C*H*_*2*_ and C*H*_*2b*_, *J* = 12.1 Hz); 1.88 (bs, 3H, C*H*); 2.81–2.82 (m, 2H, C*H*_*2*_); 6.48 (s, 1H, aryl); 7.00 (d, 1H, aryl, *J* = 8.8 Hz); 7.17 (t, 2H, aryl, *J* = 8.7 Hz); 7.26–7.28 (m, 2H, aryl); 7.40–7.43 (m, 2H, aryl); 7.54 (s, 1H, aryl). ^13^C NMR (100 MHz, CD_3_OD) δ 28.4, 36.8, 39.9, 51.2, 51.4, 103.0, 109.8, 112.2, 115.9, 116.1, 116.7, 125.6, 125.7, 128.3, 129.7, 132.4, 132.8, 136.1, 158.0, 159.8, 162.2. HR-MS *m*/*z* calcd for C_26_H_28_FN_3_O [(M + H)]^+^: 418.2289; found 418.2240.

#### 9-(4-Fluorobenzyl)-9*H*-carbazole (**46**)

Intermediate **46** was synthesized starting from carbazole and 4-fluorobenzyl chloride following general procedure E. FC in hexane/ethyl acetate 7/3, *R_f_*: 0.47. Yellow oil (97% yield). ^1^H NMR (400 MHz, CDCl_3_): δ: 5.30 (s, 2H, C*H*_2_); 6.80 (t, 2H, aryl, *J* = 9.0 Hz); 6.94–6.98 (m, 2H, aryl); 7.15 (t, 2H, aryl, *J* = 8.0 Hz); 7.20 (d, 2H, aryl, *J* = 8.4 Hz); 7.32 (t, 2H, aryl, *J* = 8.8 Hz); 8.02 (d, 2H, aryl, *J* = 8.2 Hz). ESI-MS **m*/*z** calcd for C_19_H_14_FN_2_ [(M + H)]^+^: 276.1183; found 276.1177.

#### 9-(4-Fluorobenzyl)-9*H*-carbazol-3-amine (**47**)

Intermediate **47** was obtained following general procedure B, starting from **46**. FC in hexane/ethyl acetate 2/8, *R_f_*: 0.40. Yellowish solid (90% yield). ^1^H NMR (400 MHz, CDCl_3_): δ: 3.48 (bs, 2H, N*H*_2_); 5.31 (s, 2H, C*H*_2_); 6.78–6.86 (m, 3H, aryl); 6.97–7.00 (m, 2H, aryl); 7.04 (d, 1H, aryl, *J* = 8.5 Hz); 7.10 (t, 1H, aryl, *J* = 7.8 Hz); 7.19 (d, 1H, aryl, *J* = 8.2 Hz); 7.30 (t, 1H, aryl, *J* = 7.1 Hz); 7.37 (s, 1H, aryl); 7.93 (d, 1H, aryl, *J* = 7.8 Hz). ESI-MS **m*/*z** calcd for C_19_H_15_FN_2_ [(M + H)]^+^: 291.1292; found 291.1300.

#### 1-(((3*R*,5*R*,7*R*)-Adamantan-1-yl)methyl)-3-(9-(4-fluorobenzyl)-9*H*-carbazol-3-yl)urea (**48**)

Obtained from **47** and 1-adamantanemethylamine following the general procedure C. FC in hexane/ethyl acetate 1/1, *R_f_*: 0.45. Yellowish solid (65% yield). ^1^H NMR (400 MHz, CD_3_OD): δ: 1.47 (bs, 6H, C*H*_*2*_); 1.60 (d, 3H, C*H*_*2*_ and C*H*_*2a*_, *J* = 11.7 Hz); 1.67 (d, 3H, C*H*_*2*_ and C*H*_*2b*_, *J* = 12.1 Hz); 1.89 (bs, 3H, C*H*); 2.83 (d, 2H, C*H*_*2*_, *J* = 6.0 Hz); 5.44 (s, 2H, C*H*_*2*_); 5.93 (t, 1H, aryl, *J* = 6.1 Hz); 6.86 (t, 2H, aryl, *J* = 8.8 Hz); 7.01–7.10 (m, 2H, aryl); 7.18–7.33 (m, 4H, aryl); 7.95 (d, 1H, aryl, *J* = 7.8 Hz); 8.05 (s, 1H, aryl). ^13^C NMR (100 MHz, CD_3_OD) δ 28.4, 33.7, 36.8, 39.9, 45.1, 48.4, 51.3, 53.4, 108.8, 112.1, 114.8, 115.0, 118.7, 119.8, 122.8, 123.1, 125.6, 128.1, 131.5, 133.7, 141.0, 158.1, 160.9, 163.3. HR-MS *m*/*z* calcd for C_31_H_32_FNO [(M + H)]^+^: 482.2602; found 482.2548.

### *In Vitro* Pharmacological Characterization

#### Cell-Free 5-LOX Activity Assay

Human recombinant 5-LOX was expressed in *Escherichia coli* Bl21 (DE3) cells that were transformed with the plasmid pT3-5LO (wt 5-LOX) and purified on an adenosine triphosphate (ATP)-agarose column by affinity chromatography.^40,41^ In a first step, *E. coli* was lysed in 50 mM triethanolamine/HCl pH 8.0 with ethylenediamine tetraacetic acid (EDTA) (5 mM), soybean trypsin inhibitor (60 μg/mL), phenylmethanesulfonyl fluoride (1 mM), dithiothreitol (1 mM), and lysozyme (1 mg/mL) and sonicated 3 times for 15 s. The homogenate was centrifuged at 40,000*g* for 20 min at 4 °C. The supernatant was transferred to an ATP-agarose column (Sigma-Aldrich, Deisenhofen, Germany), which was sequentially washed with phosphate-buffered saline (PBS) pH 7.4, 1 mM EDTA, 50 mM phosphate buffer pH 7.4, 0.5 M NaCl, 1 mM EDTA, and finally 50 mM phosphate buffer pH 7.4 plus 1 mM EDTA. The enzyme was eluted with 50 mM phosphate buffer pH 7.4, 1 mM EDTA, and 20 mM ATP.

Purified 5-LOX (0.5 μg in PBS pH 7.4 containing 1 mM EDTA) was preincubated with vehicle (DMSO) or test compounds for 15 min on ice. 5-LOX product formation was started by the addition of 20 μM arachidonic acid (Sigma-Aldrich) and CaCl_2_ (2 mM) and stopped by the addition of an equal volume of ice-cold methanol containing PGB_1_ (200 ng) as internal standard after 10 min at 37 °C. Major 5-LOX products (all-*trans* isomers of LTB_4_ and 5-HETE) were extracted on Sep-Pak C18 35 cc Vac Cartridges (Waters, Milford, MA), separated by RP-HPLC on a Nova-Pak C18 Radial-Pak Column (4 μm, 5 mm × 100 mm, Waters) under isocratic conditions (73% methanol/27% water/0.007% trifluoroacetic acid) at a flow rate of 1.2 mL/min and detected at 235 and 280 nm.^40^

#### 5-LOX Product Formation in Human PMNL

Human PMNL were freshly isolated from leukocyte concentrates that were obtained from the Institute for Transfusion Medicine of the University Hospital Jena (Germany). Thus, venous blood was collected in heparinized tubes (16 U heparin/mL blood), with informed consent of registered male and female healthy adult volunteers (18–65 years) who were fasted for at least 12 h. These volunteers regularly donated blood (every 8–12 weeks) and were physically inspected by a clinician. They had not taken antibiotics or anti-inflammatory drugs for more than 10 days before blood donation and were free of apparent infections, inflammatory disorders, or acute allergic reactions. Leukocytes were concentrated by centrifugation (4000*g*/20 min/20 °C) of freshly withdrawn blood. Erythrocytes were removed by dextran sedimentation and hypotonic lysis, and the supernatant was subjected to density gradient centrifugation on a lymphocyte separation medium (LSM 1077, GE Healthcare, Freiburg, Germany). PMNL were obtained from the cell pellet. Freshly isolated PMNL (5 × 10^6^) suspended in PBS pH 7.4 with 1 mg/mL glucose were preincubated with the test compounds for 15 min on ice. 5-LOX product formation in PMNL was triggered by addition of Ca^2+^ ionophore A23187 (2.5 μM; Sigma-Aldrich) followed by incubation for 10 min at 37 °C. The reaction was stopped with an equal volume of methanol containing PGB_1_ (200 ng) as internal standard. Major 5-LOX products (all-trans isomers of LTB and 5-HETE, and additionally LTB_4_) were extracted and analyzed by RP-HPLC as described for the determination of cell-free 5-LOX activity.

#### Analysis of Cell-Free sEH Activity

Human recombinant sEH was expressed in Sf9 insect cells and purified by affinity chromatography. Briefly, Sf9 cells were infected with a recombinant baculovirus and lysed after 72 h in 50 mM NaHPO_4_ pH 8, 300 mM NaCl, 10% glycerol, 1 mM EDTA, 1 mM phenylmethanesulfonyl fluoride, 10 μg/mL leupeptin, and 60 μg/mL STI by sonication (3 × 10 s, 4 °C). Sequential centrifugation at 20,000*g* (10 min, 4 °C) and 100,000*g* (60 min, 4 °C) yielded a supernatant, which was subjected to benzylthio-sepharose affinity chromatography. Elution with 4-fluorochalcone oxide in PBS pH 7.4 with 1 mM dithiothreitol and 1 mM EDTA yielded sEH, which was dialyzed and concentrated. Purified sEH (60 ng) in 25 mM Tris HCl pH 7 with 0.1 mg/mL bovine serum albumin (BSA) was preincubated with the vehicle (DMSO) or test compounds for 1 min at room temperature. The sEH substrate PHOME (20 μM, Cayman Chemicals) was added to start the enzymatic reaction, which was stopped after 60 min in the dark by the addition of ZnSO_4_ (200 mM). The formation of the fluorescent product 6-methoxynaphthaldehyde was measured using a NOVOstar fluorescence microplate reader (BMG Labtech, Ortenberg, Germany), with excitation at 330 and emission at 465 nm.

#### PG Production in Activated Macrophages

Murine monocyte/macrophage J774 cell line was obtained from the American Type Culture Collection (ATTC TIB 67). The cell line was grown in adhesion in Dulbecco’s modified Eagle’s medium (DMEM) supplemented with glutamine (2 mM, Aurogene Rome, Italy), Hepes (25 mM, Aurogene Rome, Italy), penicillin (100 U/mL, Aurogene Rome, Italy), streptomycin (100 μg/mL, Aurogene Rome, Italy), fetal bovine serum (FBS, 10%, Aurogene Rome, Italy), and sodium pyruvate (1.2%, Aurogene Rome, Italy) (DMEM completed). The cells were plated at a density of 1 × 10^6^ cells in 75 cm^2^ culture flasks and maintained at 37 °C under 5% CO_2_ in a humidified incubator until 90% confluence. The culture medium was changed every 2 days. Before a confluent monolayer appeared, subculturing cell process was carried out. Cells (0.5 × 10^6^ cells/mL) were pretreated for 2 h in the absence or presence of **28** (0.1–10 μM) and then stimulated, for 24 h, with lipopolysaccharide (LPS) from *E. coli*, Serotype 0111:B4, (10 μg/mL; 100 μL in DMEM completed with FBS, Sigma-Aldrich, Milan, Italy).^42^ The supernatants were collected for the measurement of PGE_2_ levels by ELISA assay (Cayman Chemical, Vinci-Biochem, Vinci, Italy).

### Computational Details

The ligand structures were constructed by Build Panel of Maestro (version 11), applying: OPLS3 force field,^43^ the Polak–Ribier conjugate gradient algorithm (PRCG, 9 × 10^7^ steps, maximum derivative less than 0.001 kcal/mol), generalized Born/surface area (GB/SA) solvent treatment^44^ to mimic the presence of H_2_O. Then, the structures were processed with LigPrep:^45^ all possible tautomers, stereoisomers, and protonation states, at a pH of 7.0 ± 1.0, were produced. For the docking calculations, the experimental structures of 5-LOX (PDB ID: 3O8Y)^46^ and sEH (PDB ID: 3I28)^24^ were treated by Protein Preparation Wizard:^47,48^ buffer ions, water molecules, and ligand were deleted; bond order assigned and all hydrogens were added; missing side chains, loops, and residue alternate positions were checked; the side chain charges were assigned based on their pK_a_ at physiological pH.

For investigation of ligand binding against 5-LOX, the Induced Fit Docking^48−50^ was applied, using the extended protocol, generating 80 ligand–protein poses at XP precision. Two rounds of calculations were performed using the top-ranked conformation of each ligand from the first round as input geometry for the second run. The grid center was placed on Fe^2+^, using an inner box of 10 Å, automatically generating the outer one. The conformational search parameters were sampling of ring conformations of the ligands and applying an energy window of 2.5 kcal/mol. The default values were employed for Prime refinement stage. The Glide software^51,52^ was employed for docking calculations of ligand *vs* sEH. The docking methodology was validated by docking the co-crystallized ligand 34N with sEH and then comparing the docked and experimental conformations (RMSD = 0.408 Å).^53−55^ An inner (10 Å) and outer (17 Å) receptor grid boxes were centered on *x*, *y*, and *z* coordinates: 75.42, −9.30, 68.12. First, a Standard Precision (SP) was used with default parameters to produce one pose per ligand. The so-obtained poses from the SP prediction were employed as input conformations for two rounds of Extra Precision (XP) mode calculations: flexible ligand; nitrogen inversion and ring conformation sampling with an energy cutoff of 2.5 kcal/mol; just allowing amide bond trans conformation. The enhanced sampling mode was applied, keeping 10,000 poses/ligand for the initial docking step, 1000 poses/ligand for energy minimization, and 1600 maximum output structures/ligand. The scaling factor for van der Waals radii was set at 0.8, whereas at 0.15 for the partial charge cutoff. Post-docking optimization was performed on docked conformations, filtering 100 maximum number of poses and using a cutoff of 0.5 kcal/mol to reject obtained minimized docked poses. The energy contributions of aromatic bonds and intramolecular H-bond reward, Epik state penalty were accounted for in the calculations. The analysis docking outcomes and figure preparation were made by Maestro (version 11).

### Chemical and Metabolic Stability

#### Instrumentation and LCMS Conditions

The chemical and metabolic stabilities of compounds **6**, **27**, **28**, **30**, and **36** were monitored using a UHPLC system (Shimadzu, Kyoto, Japan) consisting of a CBM-40 Lite controller, two LC-40B X3 solvent delivery modules, an SPD-M40 photodiode array detector, a CTO-30A column oven and, a SIL-40AC X3 autosampler. The UHPLC system was coupled online to LCMS-2020 single quadrupole mass spectrometer (Shimadzu, Kyoto, Japan) equipped with an electrospray ionization (ESI) source operating in positive mode.

The chromatographic separation was accomplished on a Luna Omega 1.6 μm Polar C18 100 Å, 100 mm × 2.1 mm (Phenomenex, Bologna, Italy) maintained at 40 °C. The optimal mobile phase consisted of 0.1% HCOOH/H2O v/v (A) and 0.1% HCOOH/ACN v/v (B) delivered at a constant flow rate of 0.3 mL min^–1^. Analysis was performed in gradient elution as follows: 0–8.00 min, 30–95% B; 8.00–10.00 min, isocratic to 95% B, then 5 min for column re-equilibration.

The ESI was operated in positive mode, with the following parameters: interface temperature, 350 °C; interface voltage, 4.5 kV; DL (desolvation line) temperature, 250 °C; block heater temperature, 200 °C; nebulizing gas flow (N2), 1.5 L min^–1^; drying gas pressure, 15 L min^–1^. Quantification was performed in SIM (single-ion monitoring) mode.

For the calibration curves, the primary stock solutions (10 mM) of compounds **6**, **27**, **28**, **30**, and **36** were prepared in DMSO. The working standard solutions were prepared by serial dilution of the stock solutions in methanol to obtain necessary concentrations (0.25–10 μM). A solution containing an internal standard (IS: tolbutamide) was prepared at 10 μM in methanol. Quantitation of compounds **6**, **27**, **28**, **30**, and **36** was performed using linear regression of the response ratios obtained from the calibration curve to calculate the corresponding amount (**6**: *y* = 1.97175*x* + 0.53508, *R*^2^ = 0.9998; **27**: *y* = 2.90980*x* + 0.03831, *R*^2^ = 0.9993; **28**: *y* = 2.05962*x* + 0.02242, *R*^2^ = 0.9994; **30**: *y* = 1.98080*x* + 0.14962, *R*^2^ = 0.9997; **36**: *y* = 4.59526*x* – 0.13459, *R*^2^ = 0.9991).

### Chemical Stability

1.25 μL of test compounds (5 μM final incubation concentration) was incubated in 200 μL of phosphate buffer at pH 7.4. At each specified time point (0, 0.5, 1, 1.5, and 2 h), the analyte was removed into 200 μL of ice-cold methanol containing internal standard (tolbutamide) to stop degradation. Finally, the concentration of the test compound was quantified by LC-MS. The percentage of compound remaining (relative to the 0 min time point) at the individual time points is then reported. All experiments were performed in triplicate.

### Plasma Stability

Mouse plasma is warmed to 37 °C for 10 min, mixed, and centrifuged to pellet any aggregated protein. Biotransformation is initiated by adding 1.25 μL of compound solution (5 μM final incubation concentration), and the incubation was carried out at 37 °C for 120 min in a Thermomixer comfort (Eppendorf, Hamburg, Germany). At each specified time point (0, 60, and 120 min), compounds were removed into 200 μL of ice-cold methanol containing IS to stop degradation. Finally, the concentration of the test compound was quantified by LC-MS. All experiments were performed in triplicate. Procaine (low stability) and procainamide (high stability) were used as controls.

### *In Vitro* Drug Metabolism Using Mouse Liver Microsomes

For microsomal stability assay, 25 μL of 5 mg mL^–1^ mouse (CD-1) microsomes (Thermo Fisher Scientific, Bremen, Germany) were preincubated for 5 min at 37 °C with alamethicin and 2.5 μL of each sample (5 μM). The reaction started by adding a 50 μL mixture of 10 mM NADPH and UDP-GlcUA as cofactors (1:1 v/v) and was carried out at 37 °C for 15, 30, and 60 min in a Thermomixer comfort. The reaction was stopped by the addition of 200 μL of ice-cold methanol containing IS, and then samples were centrifuged at 14,680 rpm at 25 °C for 7 min (Eppendorf microcentrifuge 5424, Hamburg, Germany). The supernatants were collected and injected in LC-MS.

The control at 0 min was obtained by the addition of the organic solvent immediately after incubation with microsomes. Testosterone, 2-naphthol, and 3-(α-acetonylbenzyl)-4-hydroxy coumarin were used as the positive controls, while the negative control was prepared by incubation for up to 60 min without cofactors. The extent of metabolism is expressed as a percentage of the parent compound turnover. All experiments were performed in triplicate.

### Metabolite Identification by Liquid Chromatography–Mass Spectrometry

LC-MS/MS analysis was performed on a Dionex UltiMate 3000 RSLC Systems coupled online to a Q-Exactive hybrid quadrupole Orbitrap mass spectrometer (Thermo Fisher Scientific, Bremen, Germany) equipped with a heated electrospray ionization probe (HESI II).

The separation was performed in reversed-phase mode with a Luna Omega 1.6 μm Polar C18 100 Å, 100 mm × 2.1 mm (Phenomenex, Bologna, Italy). The column temperature was set at 40 °C, and the flow rate was 0.3 mL min^–1^. The mobile phase consisted of (A) H_2_O and (B) ACN, both acidified with 0.1% HCOOH (v/v). The following gradient was used: 0.01–10.00 min, 2–100% B; 10.01–12.00 min isocratic to 100% B; 12.01–13.00 min, 2% B; then 5 min for column re-equilibration.

The ESI was operated in positive mode. The MS was calibrated by Thermo calmix Pierce calibration solutions in both polarities. Full MS (150–1500 *m*/*z*) and data-dependent MS/MS were performed at a resolution of 70,000 and 17,500 full width at half-maximum (FWHM) respectively, and normalized collision energy (NCE) values of 20, 40, and 60 were used. Source parameters: Sheath gas pressure, 50 arbitrary units; auxiliary gas flow, 13 arbitrary units; spray voltage, +3.5 kV, −2.8 kV; capillary temperature, 320 °C; auxiliary gas heater temperature, 350 °C.

A node-based processing workflow was custom-built in Compound Discoverer software v.3.3 (Thermo Fisher Scientific) to search and identify compound **28** metabolites. The workflow subtracted chemical background using blank samples, performed retention time alignment (0.2 min), and detected expected compounds and biotransformation products (mass tolerance: 5 ppm) with resolution-aware isotope pattern matching. Mass defect filter and Fragment ion search (FISh) Scoring were applied. Each annotation was further manually reviewed considering HCD spectra, molecular formula, isotopic pattern, and FISh coverage.

### *In Vivo* Assays

Male CD-1 mice (33–39 g, 8 weeks, Charles River Laboratories; Calco, Italy) were fed with standard rodent chow and water and acclimated for 4 days at a 12 h light and 12 h dark schedule in a constant air-conditioned environment (21 ± 2 °C). The mice were randomly assigned to groups, and experiments were carried out during the light phase. Experimental procedures were conducted in conformity with Italian (D.L. 26/2014) and European (directive 2010/63/EU) regulations on the protection of animals used for scientific purposes and approved by the Italian Ministry.

### Cerulein-Induced Pancreatitis

Acute pancreatitis (AP) was induced in mice by intraperitoneal (i.p.) injections of cerulein (50 μg/kg) hourly (5 times).^56^ The groups were: (a) Sham group which received saline i.p. hourly (×5); (b) cerulein + vehicle group which was treated i.p. hourly (×5) with cerulein (50 μg/kg, suspended in saline solution) and the vehicle (0.5 mL of DMSO 2%, i.p.) 30 min and 2.5 h after first cerulein administration (Figure 3A); (c) cerulein + **28** compound group which was treated i.p. hourly (×5) with cerulein (50 μg/kg, suspended in saline solution) and **28** (10 mg/kg, i.p.) 30 min and 2.5 h after first cerulein administration; and (d) cerulein + tested compound group which was treated i.p. hourly (×5) with cerulein (50 μg/kg, suspended in saline solution) and tested compound (AUDA, 10 mg/kg, i.p.) 30 min and 2.5 h after first cerulein administration. The mice were killed 6 h after the first cerulein injection. Blood samples were obtained by direct intracardiac puncture. Pancreases and lungs were removed immediately, frozen, and stored at −80 °C until assayed.

### Histological Examination

Portions of these organs were also fixed in formaldehyde for histological and immunohistochemical examination. Pancreas and lung sections (6 μm) were stained for hematoxylin & eosin (H&E). Evaluation of pancreas edema and inflammation signs were performed with a three-point scoring system. Edema: 0, absent or rare; 1, in the interlobular space; 2, in the intralobular space; 3, the isolated-island shape of pancreatic acinus. Inflammation: 0, absent; 1, mild (infiltration in ducts); 2, moderate (infiltration in parenchyma < 50%); 3, severe (infiltration in parenchyma > 50%). Analysis was performed in a blinded manner. Evaluation of lung edema and inflammatory infiltration was performed with three-point scoring system: 0, absent; 1, mild; 2, moderate; 3, severe.

### Immunohistochemistry

Pancreas and lung slides were processed to remove paraffin, rehydrated, and incubated with 3% hydrogen peroxide (Sigma-Aldrich, Milan, Italy) for 15 min to quench the endogenous peroxidase activity. Tissue sections were then incubated with normal goat serum (Vector Laboratories, Newark, California) for 20 min to reduce nonspecific antibody interactions. Sections were incubated with primary antibody diluted in 0.01 M PBS, 2.5% normal serum (anti-Neutrophils rat monoclonal antibody, dilution 1:100, Abcam) for 30 min. After rinsing with PBS 0.01 M for 5 min, slides were incubated with biotinylated goat anti-rat IgG (Vector Laboratories, Newark, California), diluted 1:200, for 30 min. After rinsing with PBS 0.01 M for 5 min, slides were incubated with VECTASTAIN ABC Reagent (Vector Laboratories, Newark, California) for 30 min. Color development was performed using SIGMAFAST 3,3′-diaminobenzidine solution (Sigma-Aldrich, Milan, Italy), and sections were analyzed using Leica Microsystem with a magnification of 20x. Semiquantitative determination of protein expression was obtained with ImageJ/Fiji software.

### Biochemical Assays

Liver injury was assessed by measuring the rise in plasma levels of alanine aminotransferase (ALT, a specific marker for hepatic parenchymal injury) and aspartate aminotransferase (AST, a nonspecific marker for hepatic injury) 6 h after cerulein injection by a clinical laboratory. Results are expressed in international units per milliliter.

### *In Vivo* Pharmacokinetic Profile of **28** Compound

For analysis of plasma levels of **28**, mice (6 per group) received 10 mg/kg i.p. injection in a volume of 500 μL of DMSO 2% in saline. After selected time points, the mice were sacrificed (CO_2_ atmosphere) and blood (approximately 0.7–0.9 mL) was collected by intracardiac puncture using citrate as an anticoagulant. Then, plasma was obtained by centrifugation at 800*g* at 4 °C for 10 min and immediately frozen at −80 °C. The analyses were performed using a Shimadzu Nexera UHPLC (Kyoto, Japan) consisting of two LC-30AD pumps, a SIL-30AC autosampler, a CTO-20AC column oven, and a CBM-20A controller. Chromatographic conditions have previously been reported in the Chemical and Metabolic Stability section.

The chromatographic system was coupled online to a triple quadrupole LCMS-8050 (Shimadzu) equipped with an electrospray ionization (ESI) source operating in positive mode. Quantification was performed in scheduled multiple reaction monitoring (MRM) mode. The transitions (Table S2) were *m*/*z* 456 > 149.2 (quantifier ion), 456 > 166.2 (qualifier ion) for compound **28**, and *m*/*z* 458 > 149.2 (quantifier ion), 458 > 166.2 (qualifier ion) for IS. Dwell time, interface temperature, DL temperature, and heat block temperature were set to 100 ms, 300, 250, and 350 °C, respectively. Nebulizing gas, heating gas, and drying gas flow were set to 3, 10, and 10 L min^–1^, respectively. All of the data was collected in the centroid mode and acquired and processed using Lab Solution workstation software.

Stock solutions of **28** and IS were prepared in DMSO (1 mg mL^–1^). The standards of **28** were prepared by dilution of the stock solution with methanol to obtain the working stock solutions in plasma of the following concentrations: 0.2, 0.5, 1, 5, 10, 20, and 50 ng mL^–1^ for calibration curve. Calibration standards were prepared by spiking 100 μL of mouse plasma with a suitable working solution of **28** and IS to obtain a final concentration of 5 ng mL^–1^ of the IS in each sample.

For the extraction of **28** from mouse plasma, the method of protein precipitation was employed. Briefly, the frozen plasma samples were thawed at room temperature. 200 μL of ice-cold acetonitrile containing IS was added to 100 μL of plasma. The extraction was performed by vortex mixing for 5 min, followed by centrifugation for 10 min at 14,600 rpm at 25 °C. The supernatants were collected, filtered by 0.45 μM RC-membranes, and then injected in UHPLC-MS/MS.

### Targeted Analysis of Lipid Mediators by Liquid Chromatography–Tandem Mass Spectrometry

#### Sample Extraction

The extraction of targeted lipid mediators from pancreas was performed as follows: tissues were lyophilized and weighted, 10 mg was homogenized and deproteinized with 500 μL of ice-cold 10% *v*/*v* MeOH spiked with labeled standards, and subsequently diluted with 500 μL of phosphate-buffered saline (PBS). Samples were then loaded on solid-phase extraction cartridges, Strata-X reversed-phase SPE columns (100 mg/3 mL) (Phenomenex, Bologna, Italy). Cartridges were conditioned with 3 mL of MeOH and equilibrated with 3 mL of ddH_2_O. Following loading, washed with 1 mL of 10% MeOH *v*/*v*, and finally eluted with 1.5 mL of MeOH. Eluted samples were dried with a SpeedVac (Savant, Thermo Scientific, Milan, Italy) and dissolved prior to UHPLC-MS/MS analyses in 50 μL of UHPLC solvent A.

### UHPLC-MS/MS Parameters

The analyses were acquired using a Shimadzu Nexera UHPLC (Kyoto, Japan) coupled online to a triple quadrupole LCMS-8050 (Shimadzu) equipped with an electrospray ionization (ESI) source operating in ESI negative mode. The separation was performed on a Kinetex EVO C18 150 mm × 2.1 mm, 2.6 μm (100 Å) (Phenomenex, Bologna, Italy). The flow rate was set to 0.4 mL/min, and the column oven was set at 40 °C. The mobile phases were, respectively: (A) ACN/H_2_O 45:55 acidified with 0.02% CH_3_COOH *v*/*v* and (B) ACN/IPA 50:50. The following gradient was used: 0 min, 0%B, 0–12 min 60%B, 12.10 min, 60–99%B, 12.10–13.05 isocratic to 99% B; returning to 0% B in 0.1. MS source parameters: desolvation Line (DL) 250 °C, interface: 300 °C, block heater 350 °C, nebulizing (N_2_), drying (N_2_), and heating (Air) gas pressures: 3, 10, 10 L/min. Each analyte was optimized by infusing standard solution at 0.2 mg/mL, multiple reaction monitoring (MRM) mode was used. Detailed MRM parameters are available in Table S3. Authentic and isotope labeled standards were acquired from Cayman Chemicals (Ann Arbor, Michigan): (±)5,6-dihydroxy-8*Z*,11*Z*,14*Z*-eicosatrienoic acid (5,6 DHET), (±)8,9-dihydroxy-5*Z*,11*Z*,14*Z*-eicosatrienoic acid (8,9 DHET), (±)11,12-dihydroxy-5*Z*,8*Z*,14*Z*-eicosatrienoic acid (11,12 DHET), (±)14,15-dihydroxy-5*Z*,8*Z*,11*Z*-eicosatrienoic acid (14,15 DHET), (±)5-hydroxy-6E,8*Z*,11*Z*,14*Z*-eicosatetraenoic acid (5 HETE), (±)12-hydroxy-5*Z*,8*Z*,10*E*,14*Z*-eicosatetraenoic acid (12 HETE), (±)15-hydroxy-5*Z*,8*Z*,11*Z*,13*E*-eicosatetraenoic acid (15 HETE), 20-hydroxy-5*Z*,8*Z*,11*Z*,14*Z*-eicosatetraenoic acid (20-HETE), (±)5,6-epoxy-8*Z*,11*Z*,14*Z*-eicosatrienoic acid (5,6 EET), (±)8,9-epoxy-5*Z*,11*Z*,14*Z*-eicosatrienoic acid (8,9 EET), (±)11,(12)-epoxy-5*Z*,8*Z*,14*Z*-eicosatrienoic acid (11,12 EET), (±)14(15)-epoxy-5*Z*,8*Z*,11*Z*-eicosatrienoic acid (14,15 EET), (±)14(15)-epoxy-5*Z*,8*Z*,11*Z*-eicosatrienoic-16,16,17,17,18,18,19,19,20,20,20-d11 acid (14,15-EET-d11), (±)11,12-dihydroxy-5*Z*,11*Z*,14*Z*-eicosatrienoic-16,16,17,17,18,18,19,19,20,20,20-d11 acid (11,12-DHET-d11), 5*S*-hydroxy-6*E*,8*Z*,11*Z*,14*Z*-eicosatetraenoic-5,6,8,9,11,12,14,15-d8 acid (5(*S*)-HETE-d8), 15(*S*)-hydroxy-5*Z*,8*Z*,11*Z*,13*E*-eicosatetraenoic-5,6,8,9,11,12,14,15-d8 acid (15(*S*)-HETE-d8).

### Data Analysis

A noncompartmental pharmacokinetic analysis of compound **28** was performed using PK solver.^57^ The maximum concentration (*C*_max_) and time to reach *C*_max_ (*T*_max_) were determined from the plasma concentration versus time data. The area under the plasma concentration–time curve from time 0 to infinity concentration (AUC_0–∞_) was determined using the linear trapezoidal method. The elimination half-life (*T*_1/2_) was calculated using ln(2)/*k*_e_.

The *in vivo* results are expressed as mean ± SEM of the mean of n observations, where n represents the number of animals or number of experiments performed at different days. Statistical evaluation was performed by one-way or two-way ANOVA using GraphPad InStat (GraphPad Software, Inc., San Diego, CA) followed by a Bonferroni post hoc test for multiple comparisons, respectively. Post hoc tests were run only when F achieved P < 0.05, and there was no significant variance in the homogeneity. P value < 0.05 was used to define statistically significant differences between mean values.

## Supporting Information Available

NMR spectra and HPLC traces of synthesized compounds (Figures S1–S63); three-dimensional model of the interactions given by some of the synthesized compounds with sEH (Figures S64–S66); effect of **28** on PG production in LPS-stimulated murine macrophages (Figure S67); observed metabolites of **28** compound in mouse liver microsomes (Table S1); MS2 fragmentation spectra of compound **28** (Figures S68–S70); optimal LC-MS/MS parameters for **28** compound quantification (Table S2); optimized MRM parameters for the quantification of lipid mediators (Table S3); and representative MRM traces of monitored eicosanoids (Figure S71) (PDF)
Atomic coordinates for complex **3**/5-LOX (PDB)
Atomic coordinates for complex **4**/5-LOX (PDB)
Atomic coordinates for complex **5**/5-LOX (PDB)
Atomic coordinates for complex **6**/5-LOX (PDB)
Atomic coordinates for complex **7**/5-LOX (PDB)
Atomic coordinates for complex **8**/5-LOX (PDB)
Atomic coordinates for complex **3**/sEH (PDB)
Atomic coordinates for complex **5**/sEH (PDB)
Atomic coordinates for complex **6**/sEH (PDB)
Atomic coordinates for complex **7**/sEH (PDB)
Atomic coordinates for complex **10**/sEH (PDB)
Atomic coordinates for complex **14**/sEH (PDB)
Atomic coordinates for complex **27**/sEH (PDB)
Atomic coordinates for complex **28**/sEH (PDB)
Atomic coordinates for complex **29**/sEH (PDB)
Atomic coordinates for complex **30**/sEH (PDB)
Atomic coordinates for complex **32**/sEH (PDB)
Atomic coordinates for complex **33**/sEH (PDB)
Atomic coordinates for complex **36**/sEH (PDB)
Atomic coordinates for complex **37**/sEH (PDB)
Atomic coordinates for complex **38**/sEH (PDB)
Atomic coordinates for complex **39**/sEH (PDB)
Atomic coordinates for complex **42**/sEH (PDB)
Atomic coordinates for complex **45**/sEH (PDB)
Atomic coordinates for complex **48**/sEH (PDB)
Molecular formula strings (CSV)

## Supplementary Material

jm3c00831_si_001.pdf

jm3c00831_si_002.pdb

jm3c00831_si_003.pdb

jm3c00831_si_004.pdb

jm3c00831_si_005.pdb

jm3c00831_si_006.pdb

jm3c00831_si_007.pdb

jm3c00831_si_008.pdb

jm3c00831_si_009.pdb

jm3c00831_si_010.pdb

jm3c00831_si_011.pdb

jm3c00831_si_012.pdb

jm3c00831_si_013.pdb

jm3c00831_si_014.pdb

jm3c00831_si_015.pdb

jm3c00831_si_016.pdb

jm3c00831_si_017.pdb

jm3c00831_si_018.pdb

jm3c00831_si_019.pdb

jm3c00831_si_020.pdb

jm3c00831_si_021.pdb

jm3c00831_si_022.pdb

jm3c00831_si_023.pdb

jm3c00831_si_024.pdb

jm3c00831_si_025.pdb

jm3c00831_si_026.pdb
