# Supplementary material for: Design, Synthesis, and Pharmacological Characterization of a Potent Soluble Epoxide Hydrolase Inhibitor for the Treatment of Acute Pancreatitis
Source: J Med Chem. 2023 Jun 19;66(13):9201–22. doi: 10.1021/acs.jmedchem.3c00831 (PMC10350924; doi:10.1021/acs.jmedchem.3c00831)
Supplement: Supplementary file 1 — jm3c00831_si_001.pdf [file jm3c00831_si_001.pdf]

SUPPORTING INFORMATION

Design, synthesis and pharmacological characterization of a potent soluble epoxide hydrolase inhibitor for the treatment of acute pancreatitis

Simona Musella,[‡,#] Danilo D'Avino,[¥,#] Lukas Klaus Peltner,[¶,#] Veronica Di Sarno,[‡] Ida Cerqua,[¥] Fabrizio Merciai,[‡] Vincenzo Vestuto,[‡] Tania Ciaglia,[‡] Gerardina Smaldone,[‡] Francesca Di Matteo,[‡] Simone Di Micco,[₸] Valeria Napolitano,[‡] Giuseppe Bifulco,[‡] Giacomo Pepe,[‡] Eduardo Maria Sommella,[‡] Manuela Giovanna Basilicata,[‡] Giovanna Aquino,[‡] Isabel M. Gomez-Monterrey,[¥] Pietro Campiglia,[‡,₸] Carmine Ostacolo,[¥] Fiorentina Roviezzo,[¥] Oliver Werz,[¶] Antonietta Rossi,[¥,*] Alessia Bertamino.[‡,*]

[¥] Department of Pharmacy, University Federico II of Naples, Via D. Montesano 49, 80131, Naples, Italy.

[‡] Department of Pharmacy, University of Salerno, Via G. Paolo II 132, 84084, Fisciano, Salerno, Italy.

[¶] Department of Pharmaceutical/Medicinal Chemistry, Institute of Pharmacy, Friedrich-Schiller-University, Philosophenweg 14, D-07743 Jena, Germany.

[₸] European Biomedical Research Institute (EBRIS), Via S. De Renzi 50, 84125, Salerno, Italy.

Corresponding Authors: Antonietta Rossi (antrossi@unina.it) and Alessia Bertamino (abertamino@unisa.it)

Table of content:

1. **Figures S1-S63:** NMR spectra and HPLC traces of synthesized compounds....................**S2-S63**
2. **Figures S64-S66:** Three-dimensional model of the interactions given by some of the synthesized compounds with sEH..**S64-S66**
3. **Figure 67:** Effect of **28** on PG production in LPS-stimulated murine macrophages………………**S67**
4. **Table S1:** Observed metabolites of **28** compound in mouse liver microsomes……………………**S68**
5. **Figures 68-70:** MS2 fragmentation spectra of compound **28**…………………………………**S69-S71**
6. **Table S2:** Optimal LC-MS/MS parameters for **28** compound quantification……………………....**S72**
7. **Table S3**: Optimized MRM parameters for the quantification of lipid mediators…………………..**S73**
8. **Figure S71:** Representative MRM traces of monitored eicosanoids..………………………………**S74**

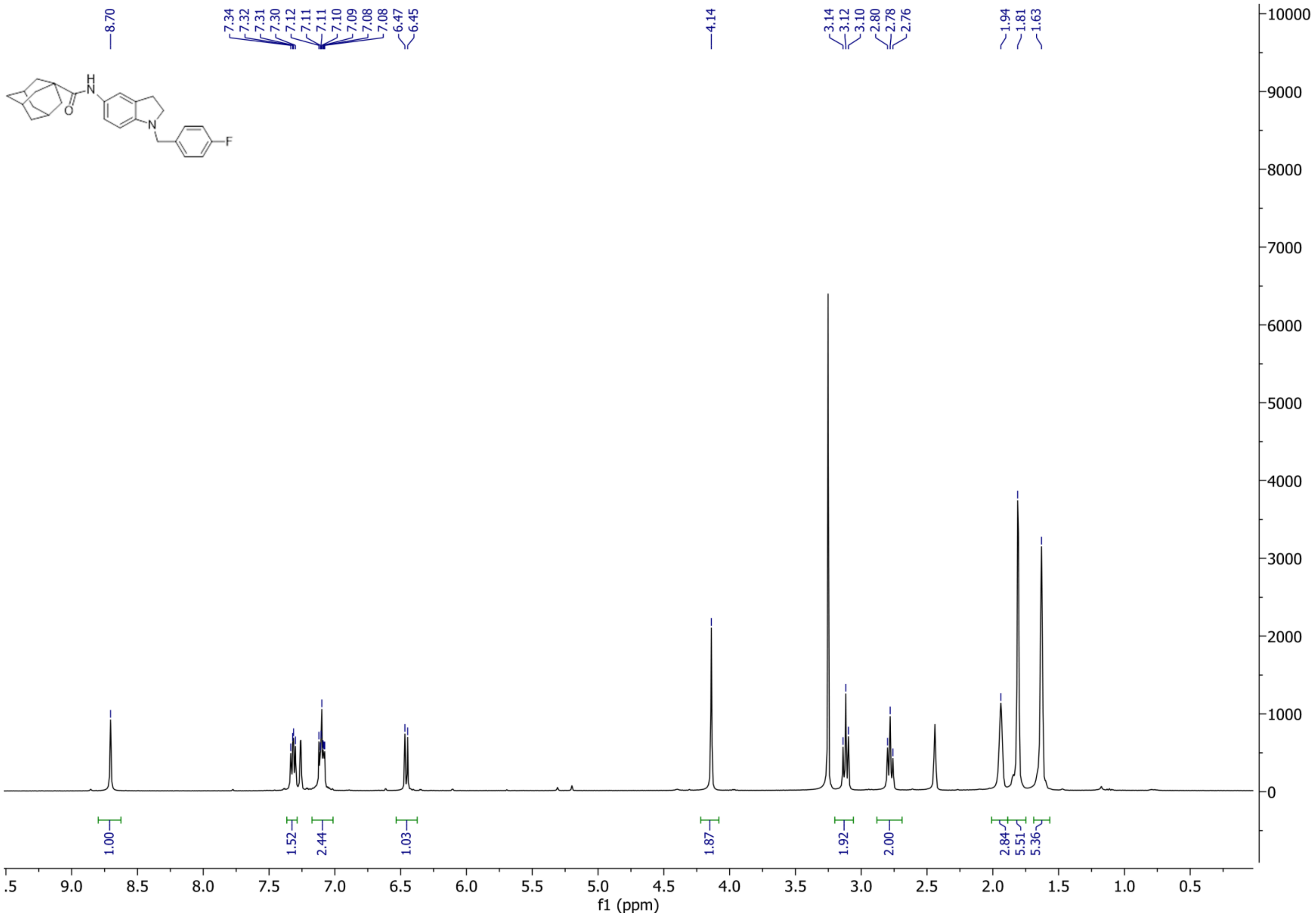

Figure S1: 1H NMR spectra of compound **3**

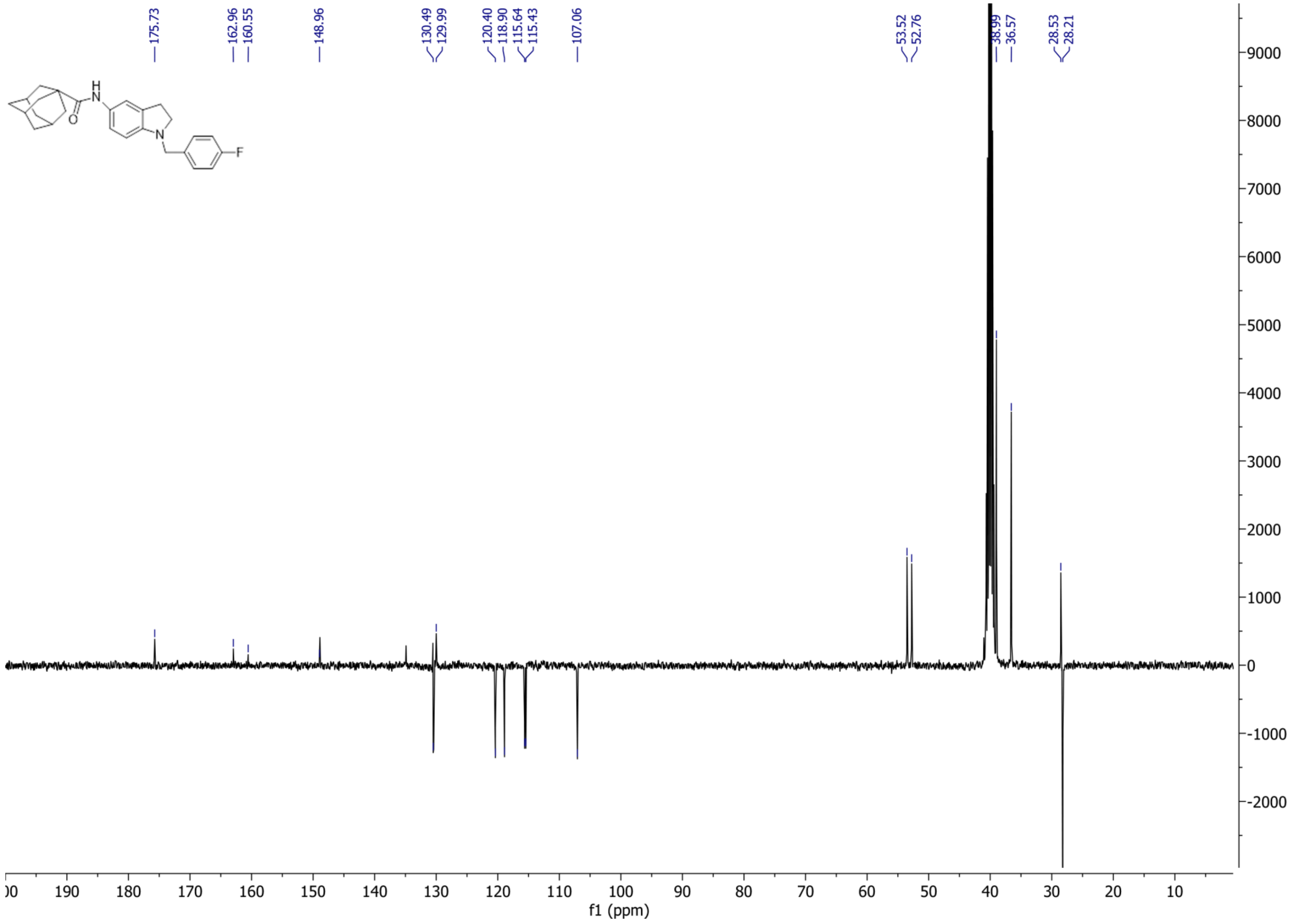

Figure S2: DEPT spectra of compound **3**

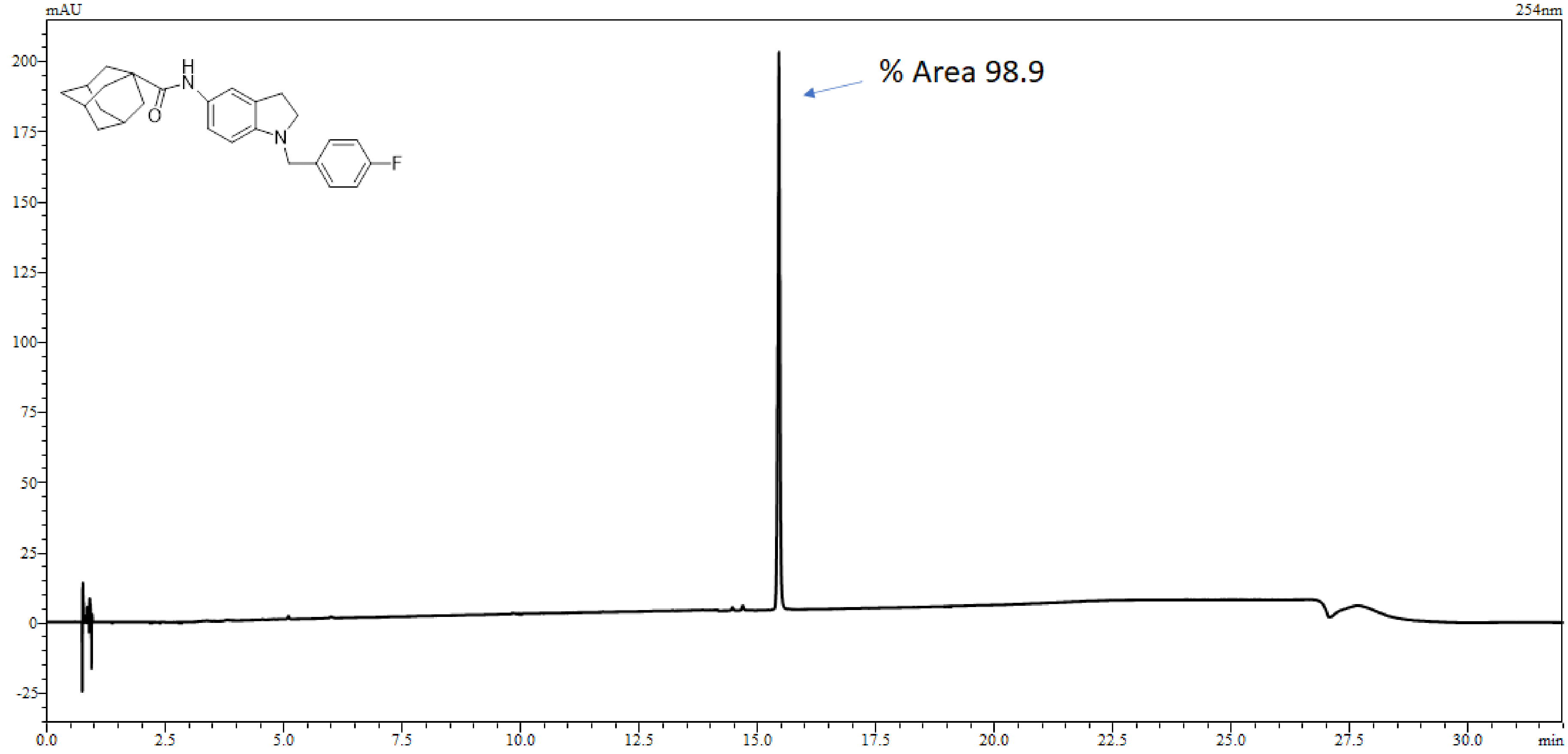

Figure S3: HPLC spectra of compound **3**

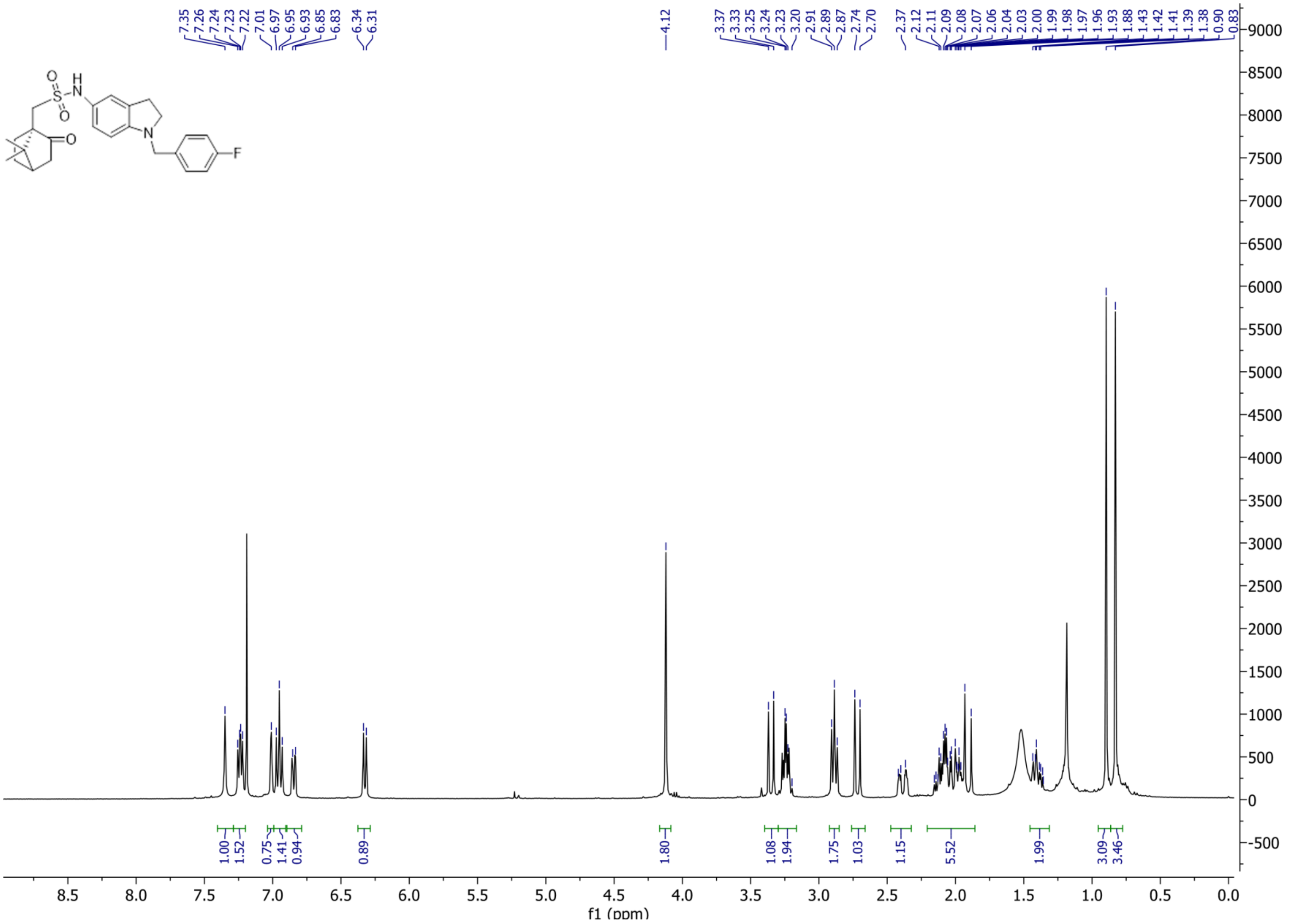

Figure S4: 1H NMR spectra of compound **4**

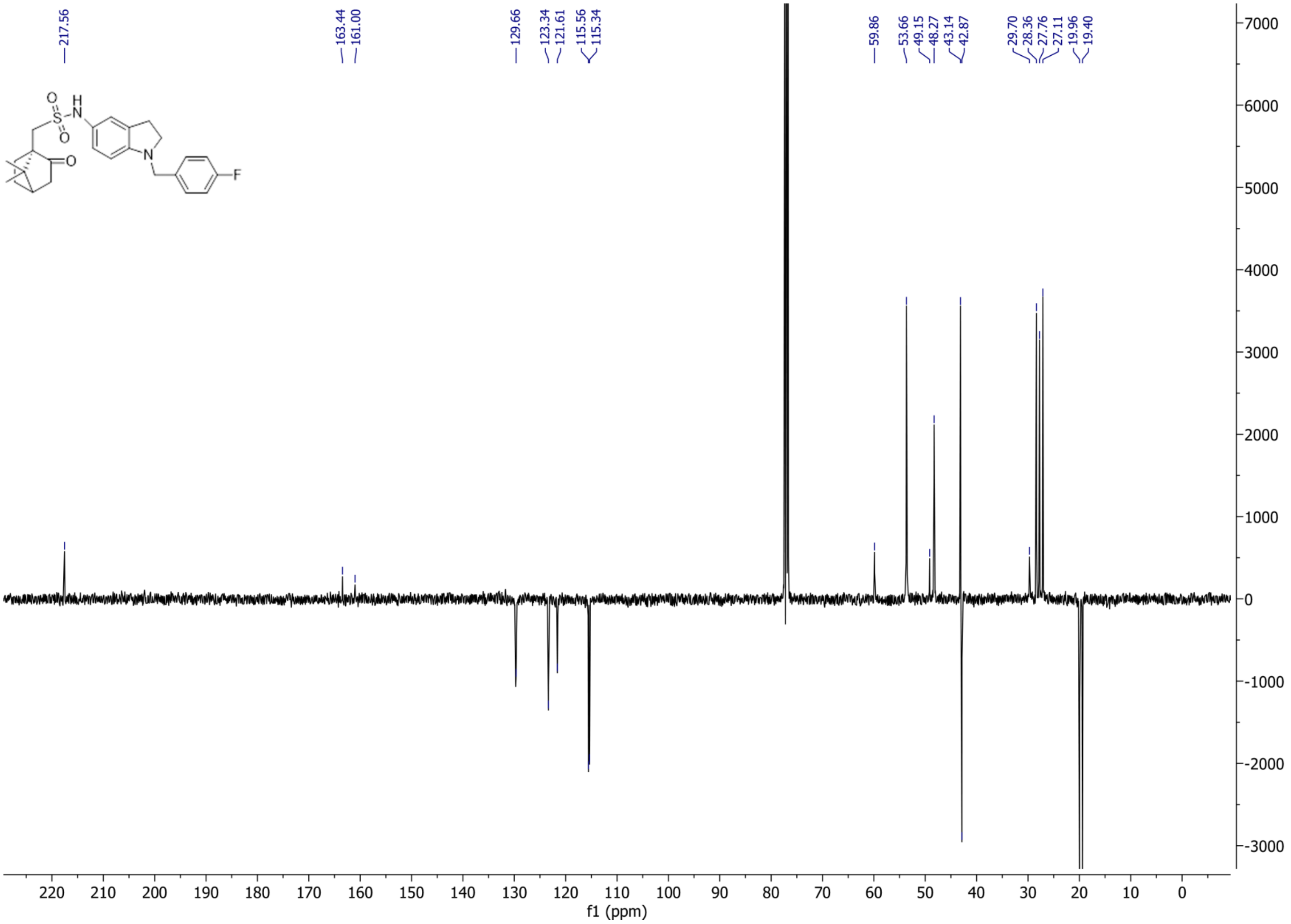

Figure S5: DEPT spectra of compound **4**

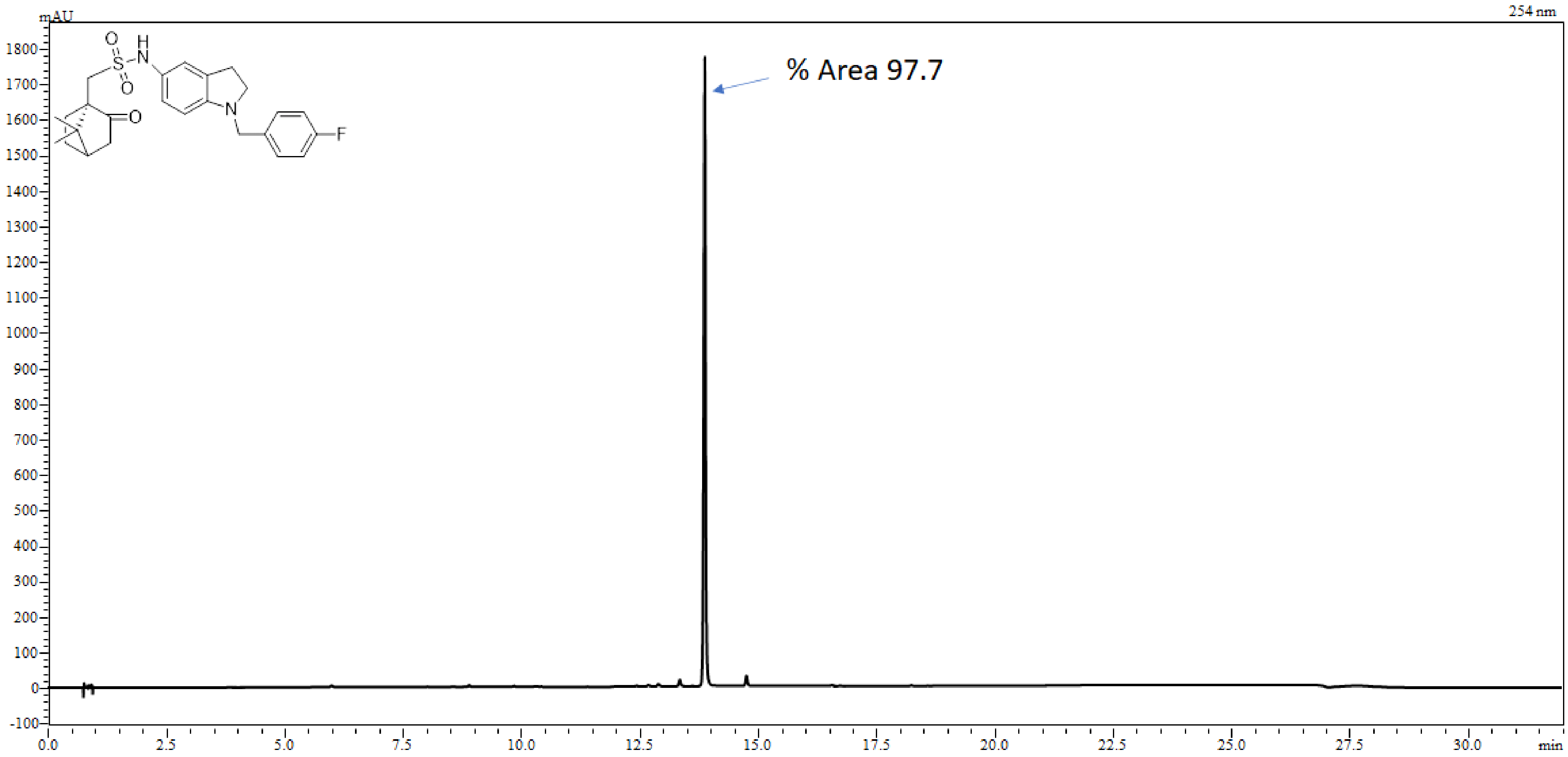

Figure S6: HPLC spectra of compound **4**

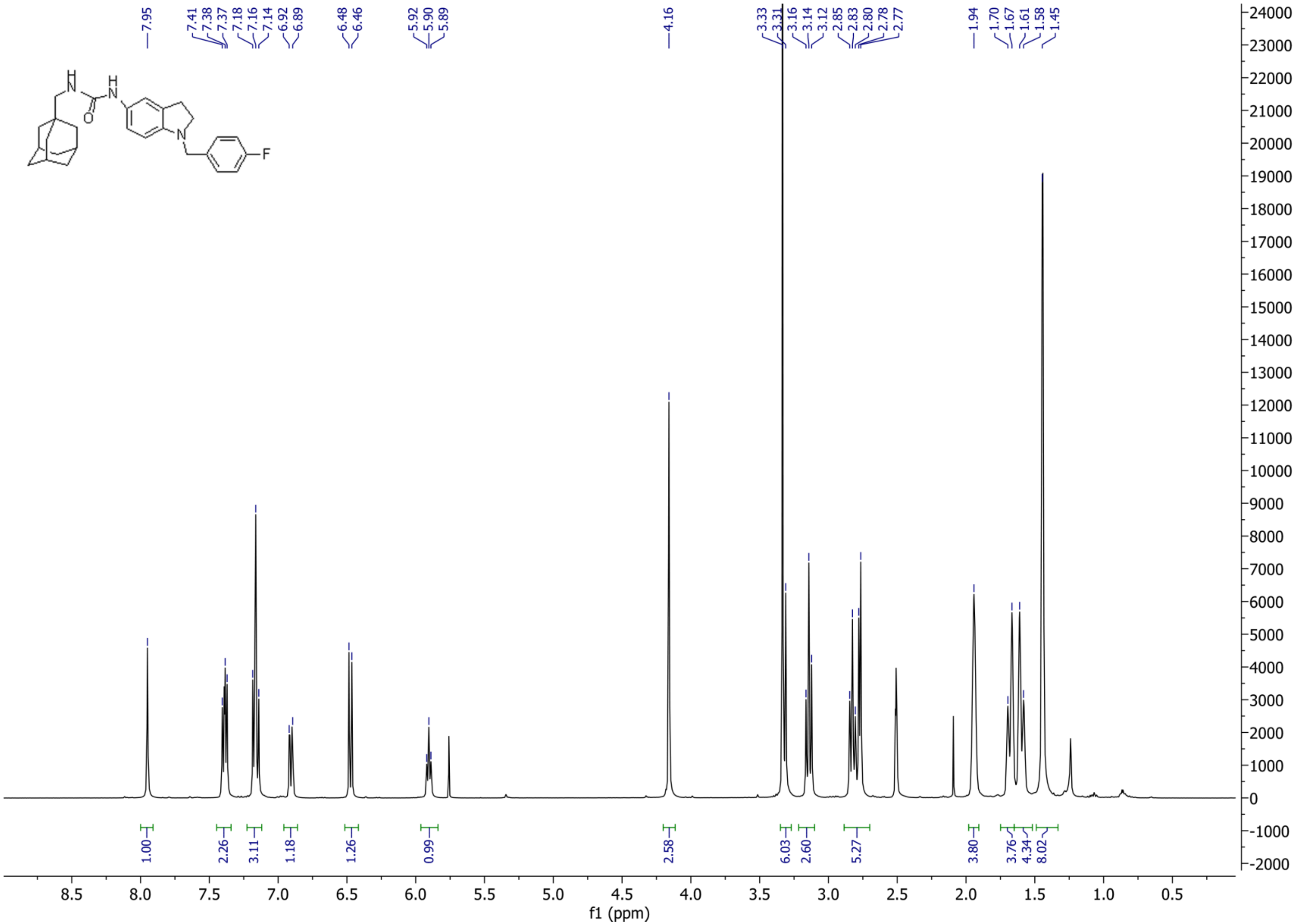

Figure S7: 1H NMR spectra of compound **5**

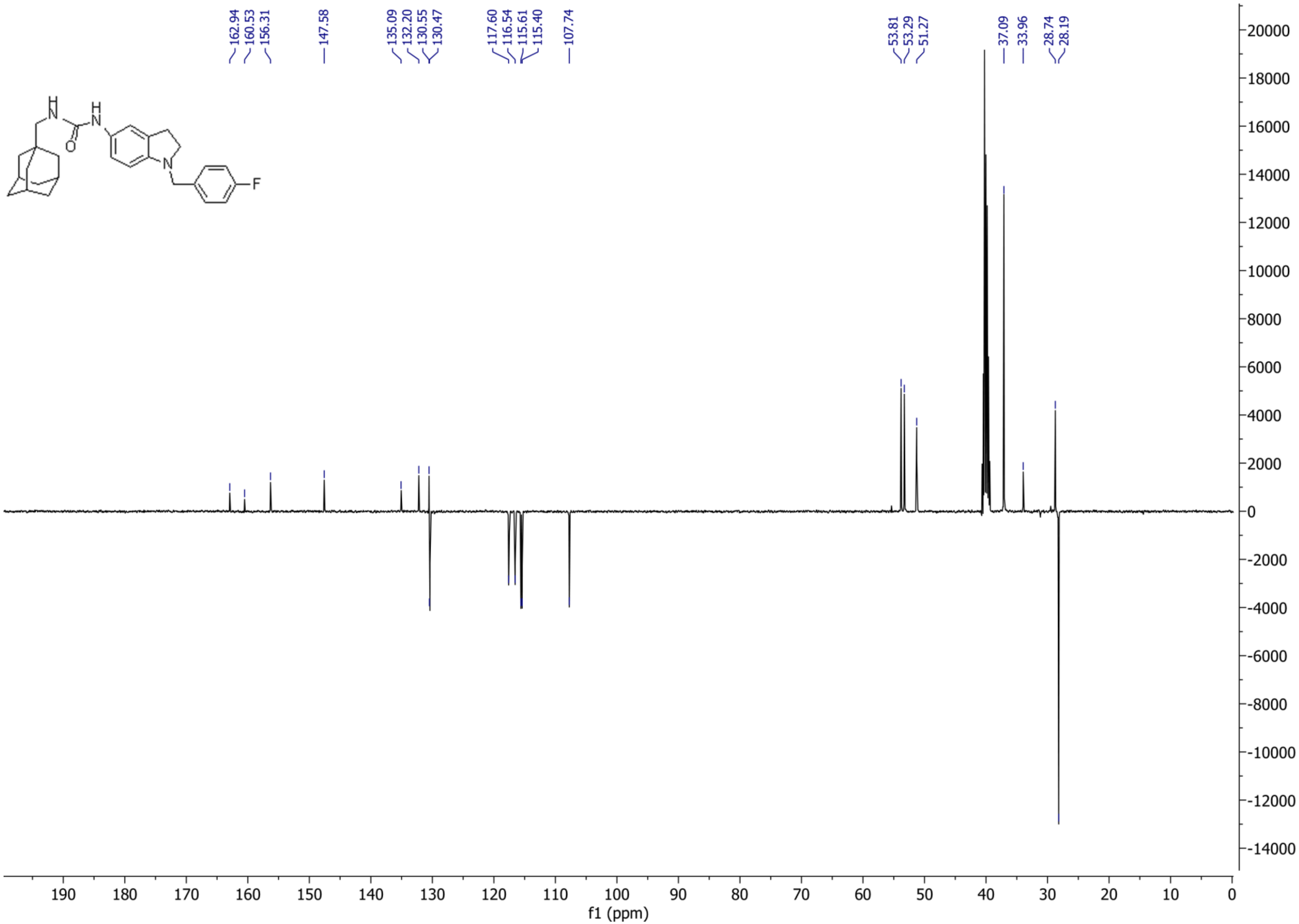

Figure S8: DEPT spectra of compound **5**

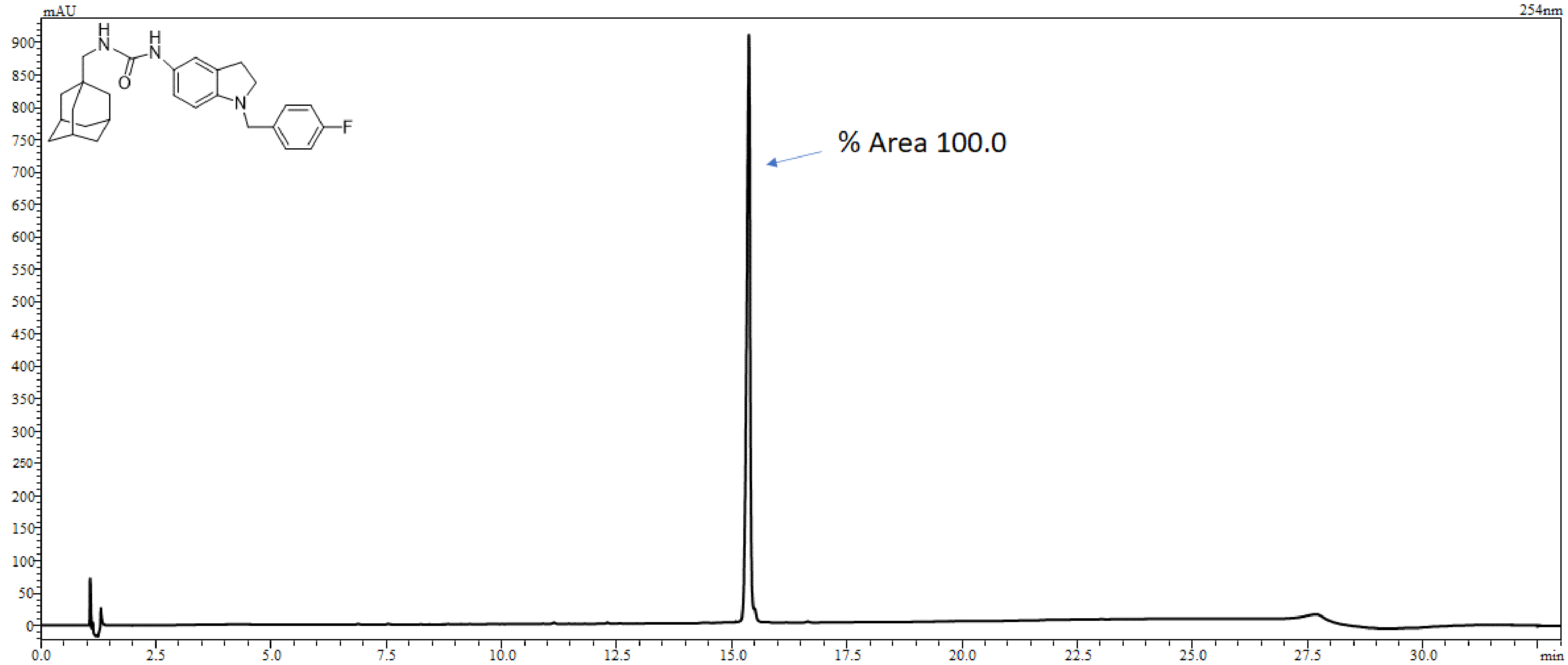

Figure S9: HPLC spectra of compound **5**

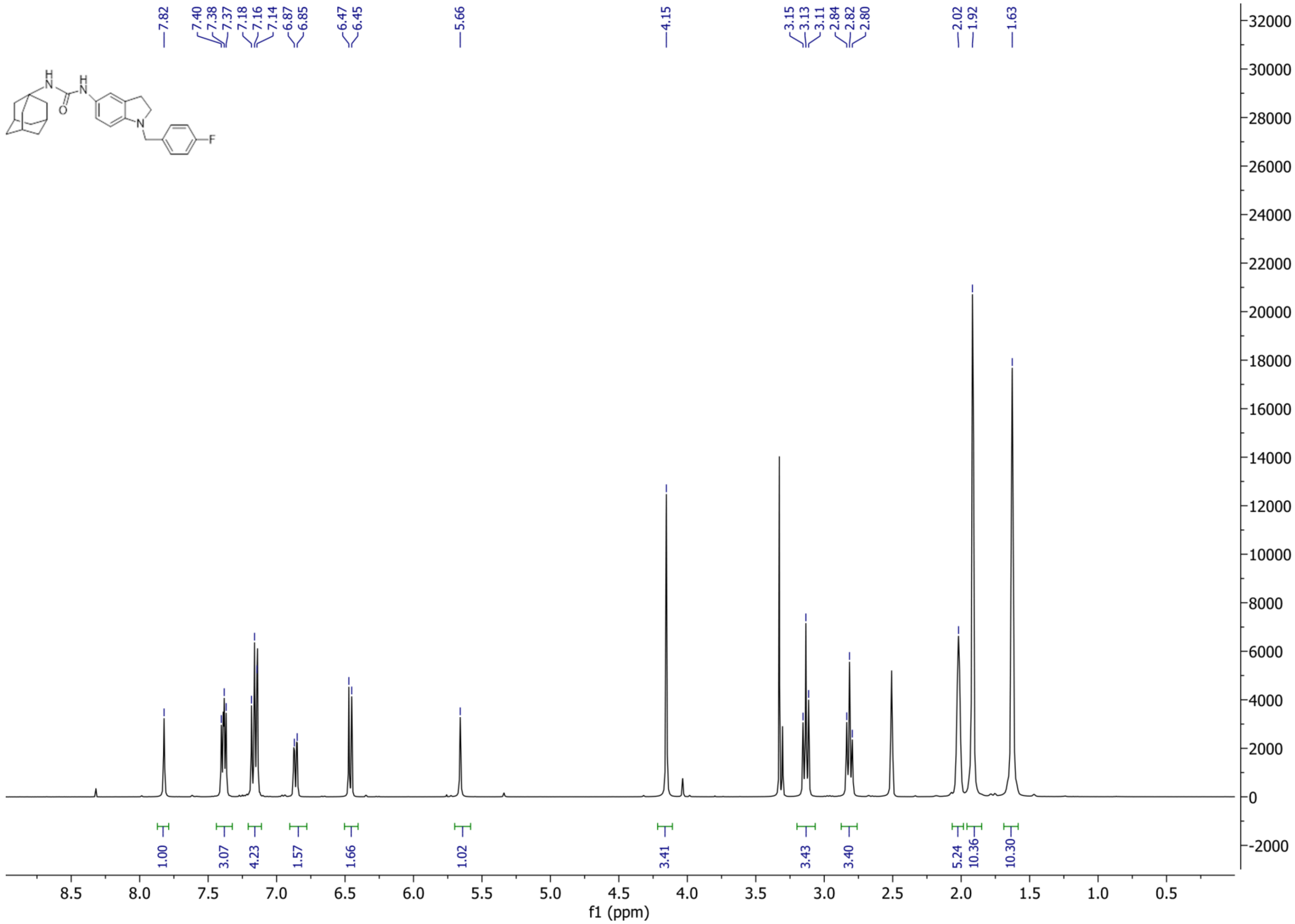

Figure S10: 1H NMR spectra of compound **6**

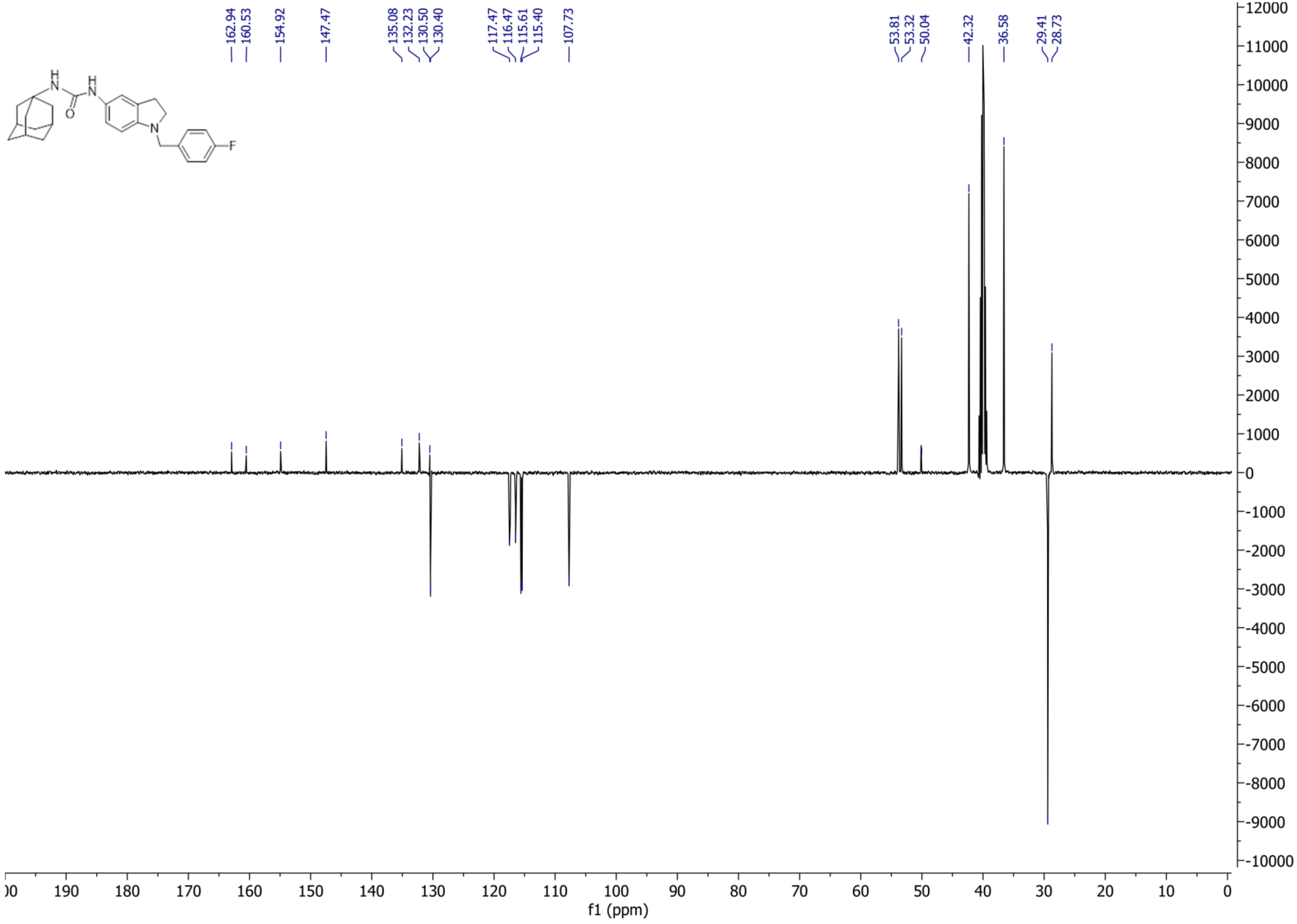

Figure S11: DEPT spectra of compound **6**

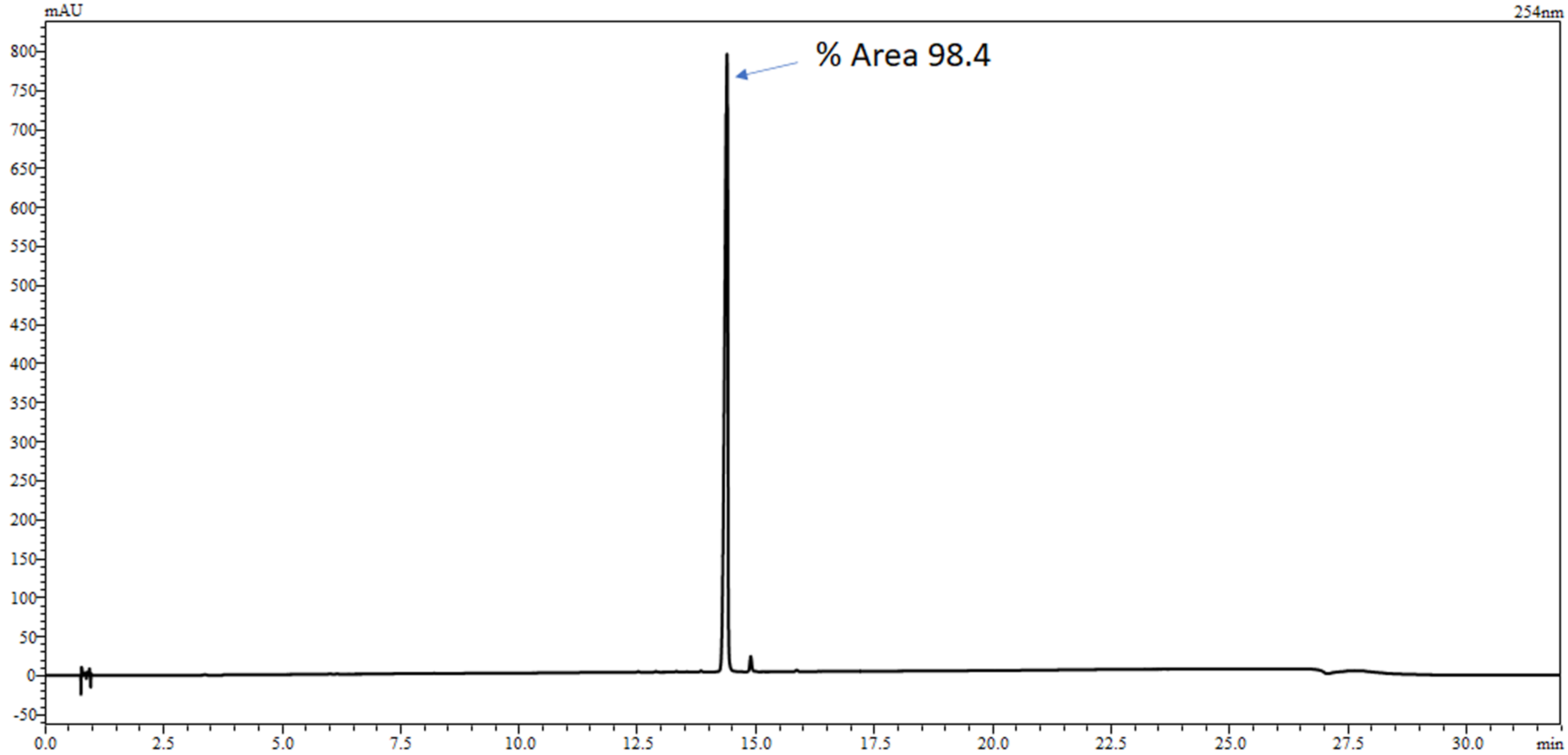

Figure S12: HPLC spectra of compound **6**

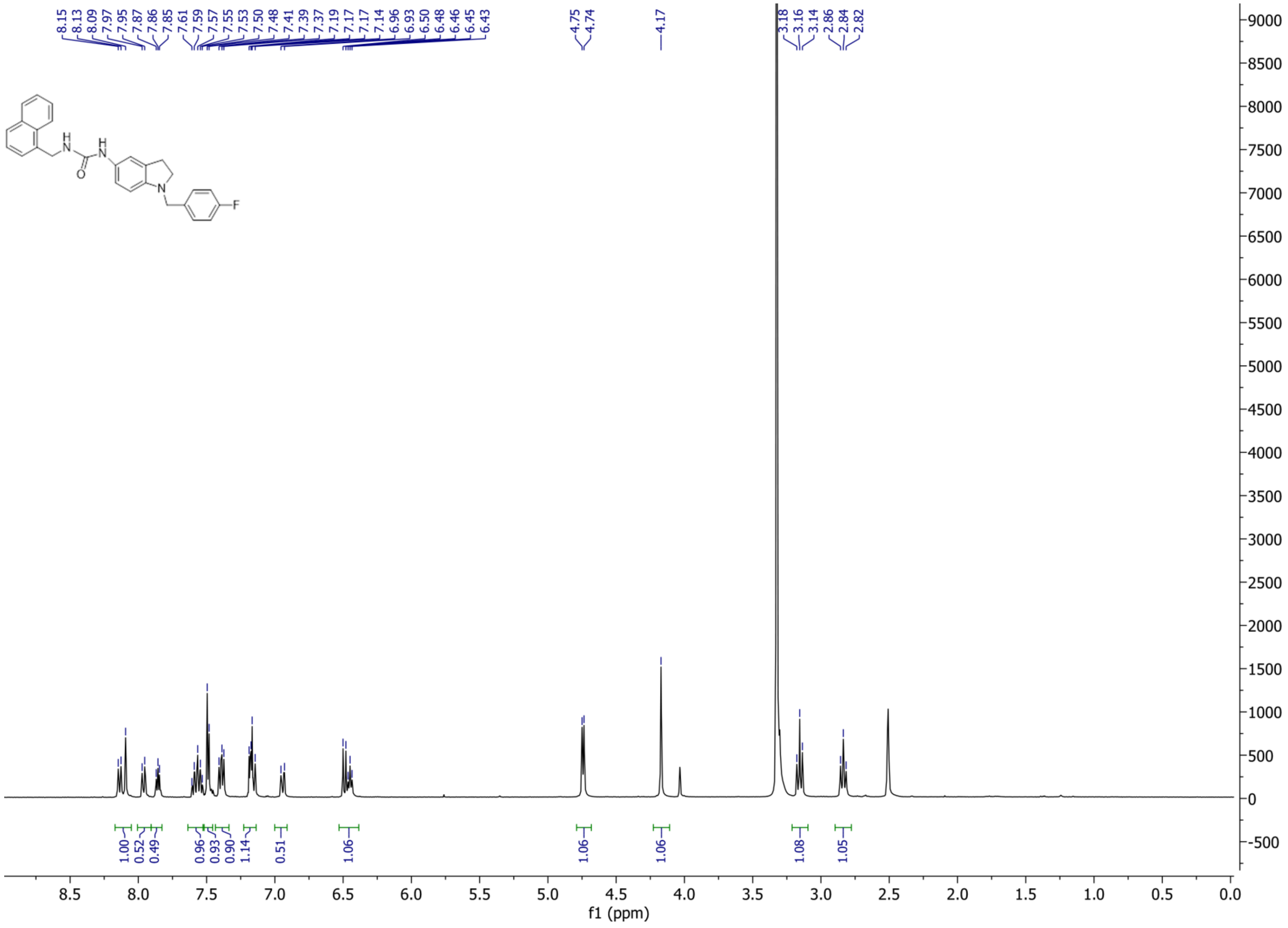

Figure S13: 1H NMR spectra of compound **7**

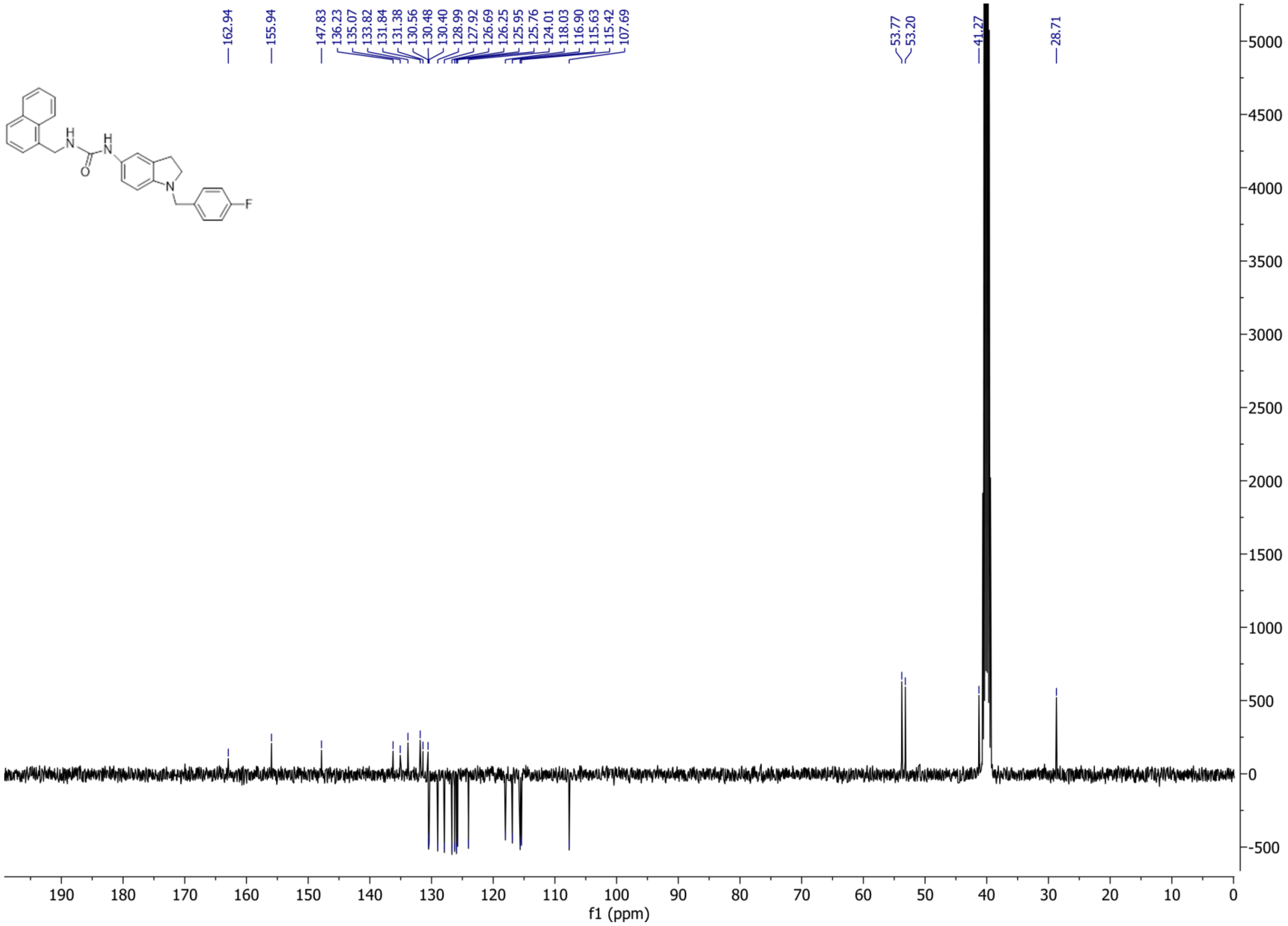

Figure S14: DEPT spectra of compound **7**

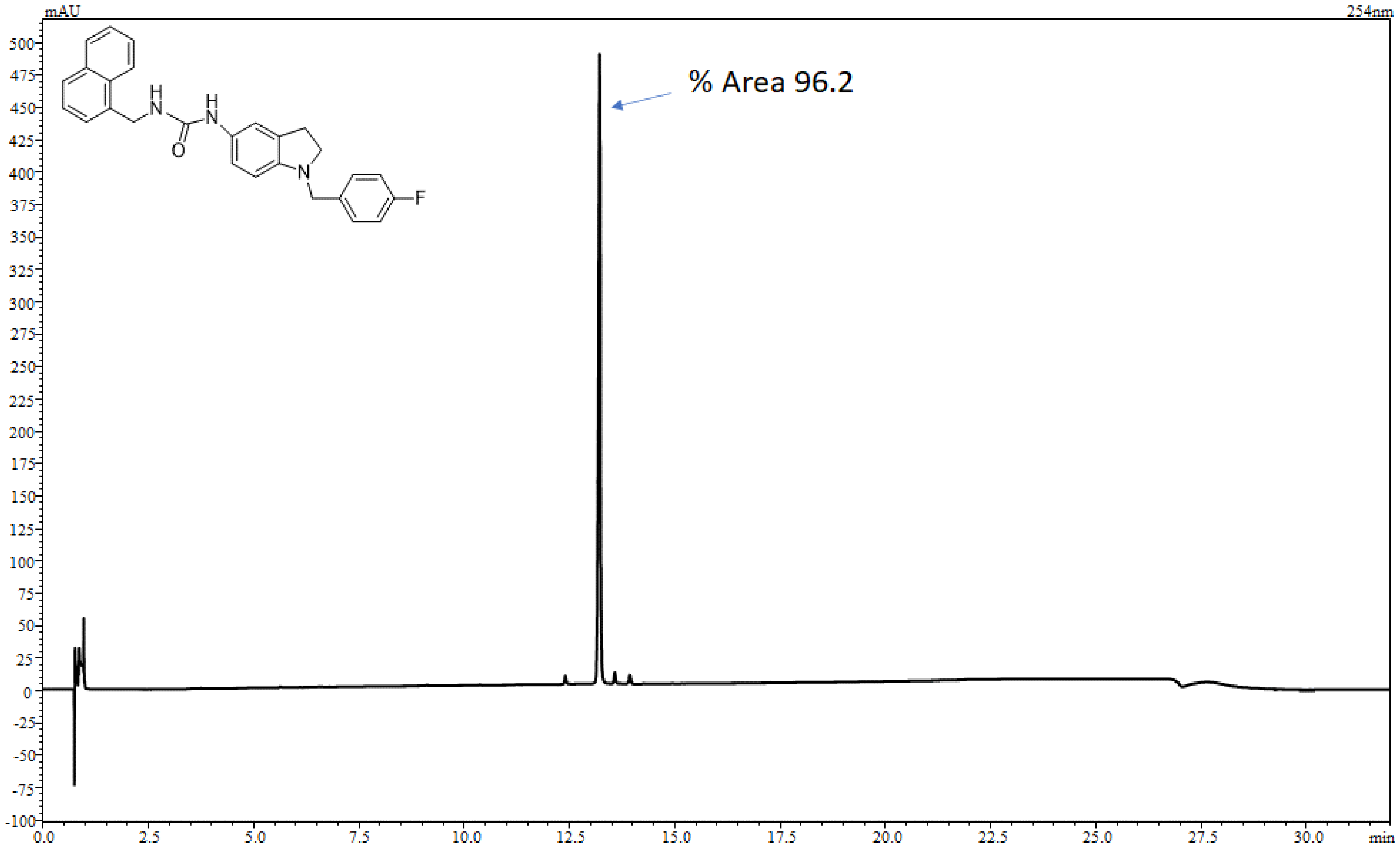

Figure S15: HPLC spectra of compound **7**

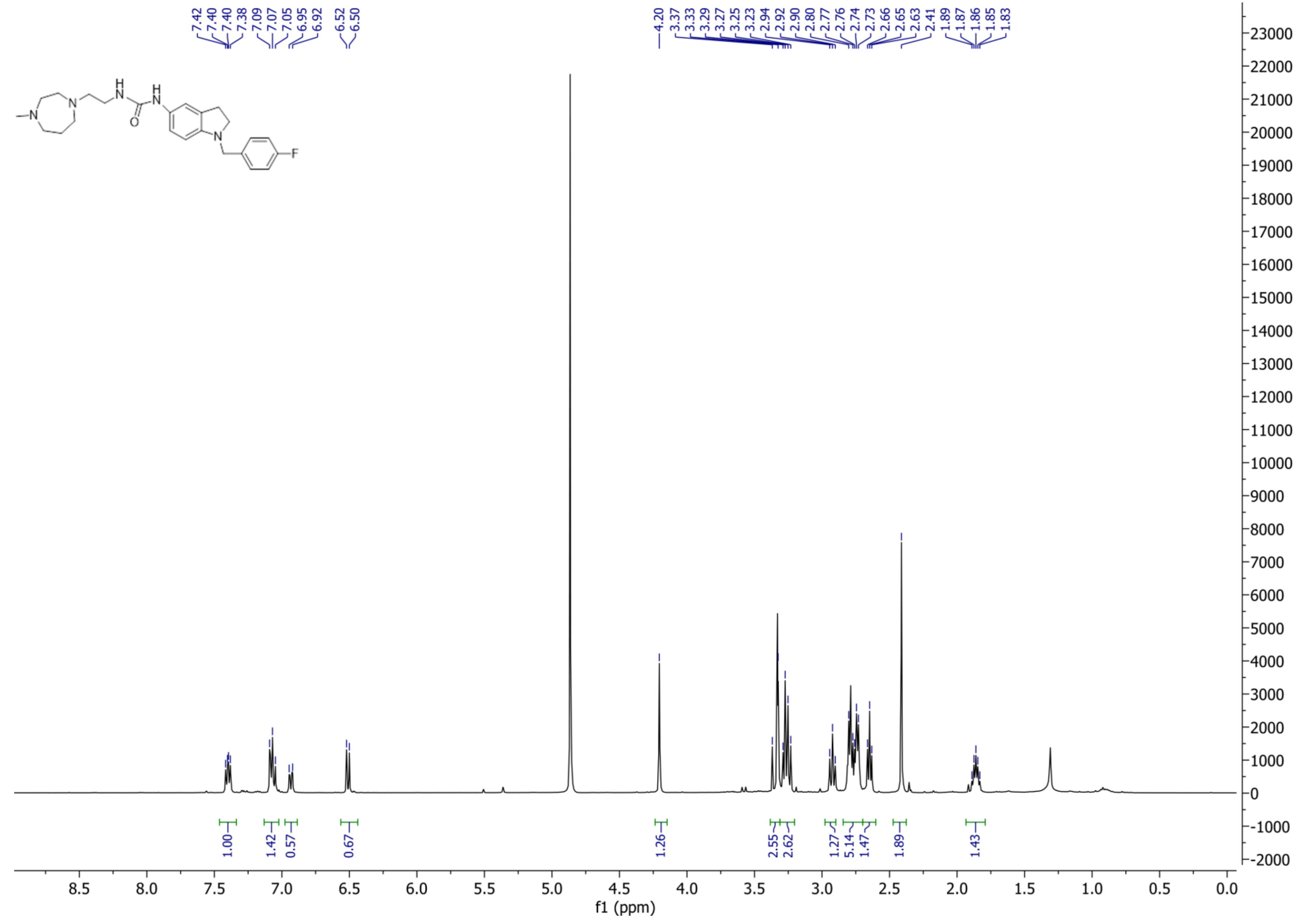

Figure S16: 1H NMR spectra of compound **8**

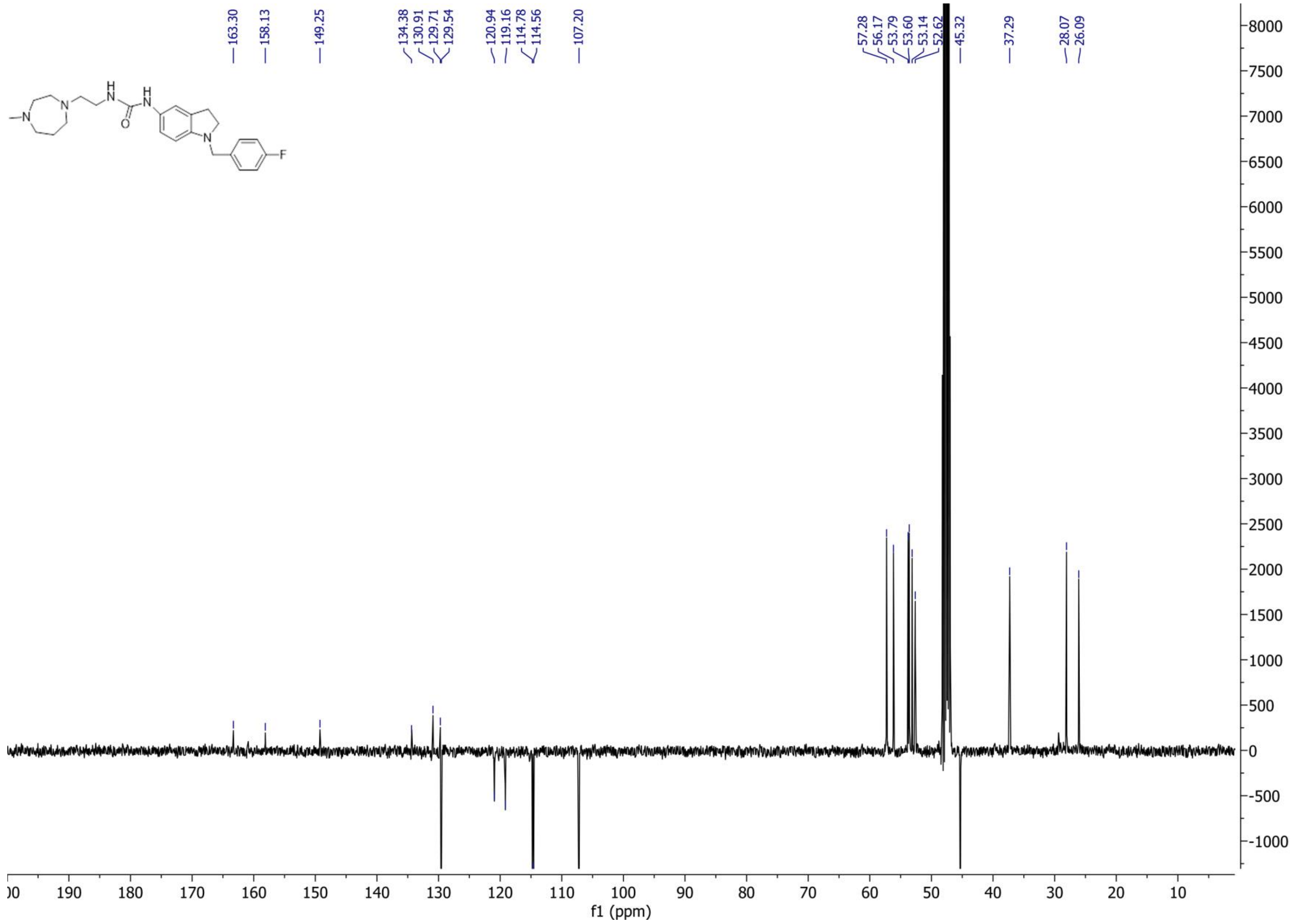

Figure S17: DEPT spectra of compound **8**

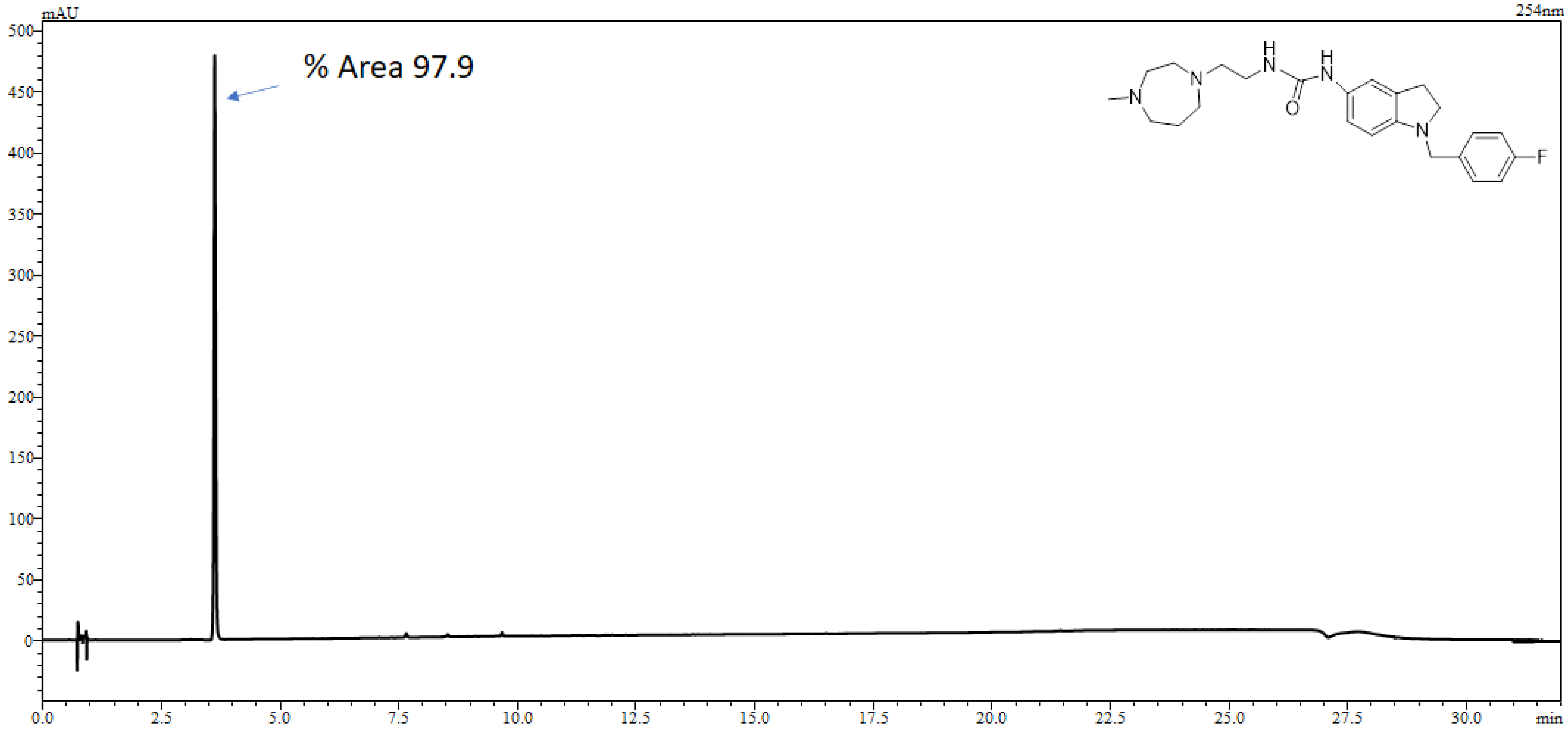

Figure S18: HPLC spectra of compound **8**

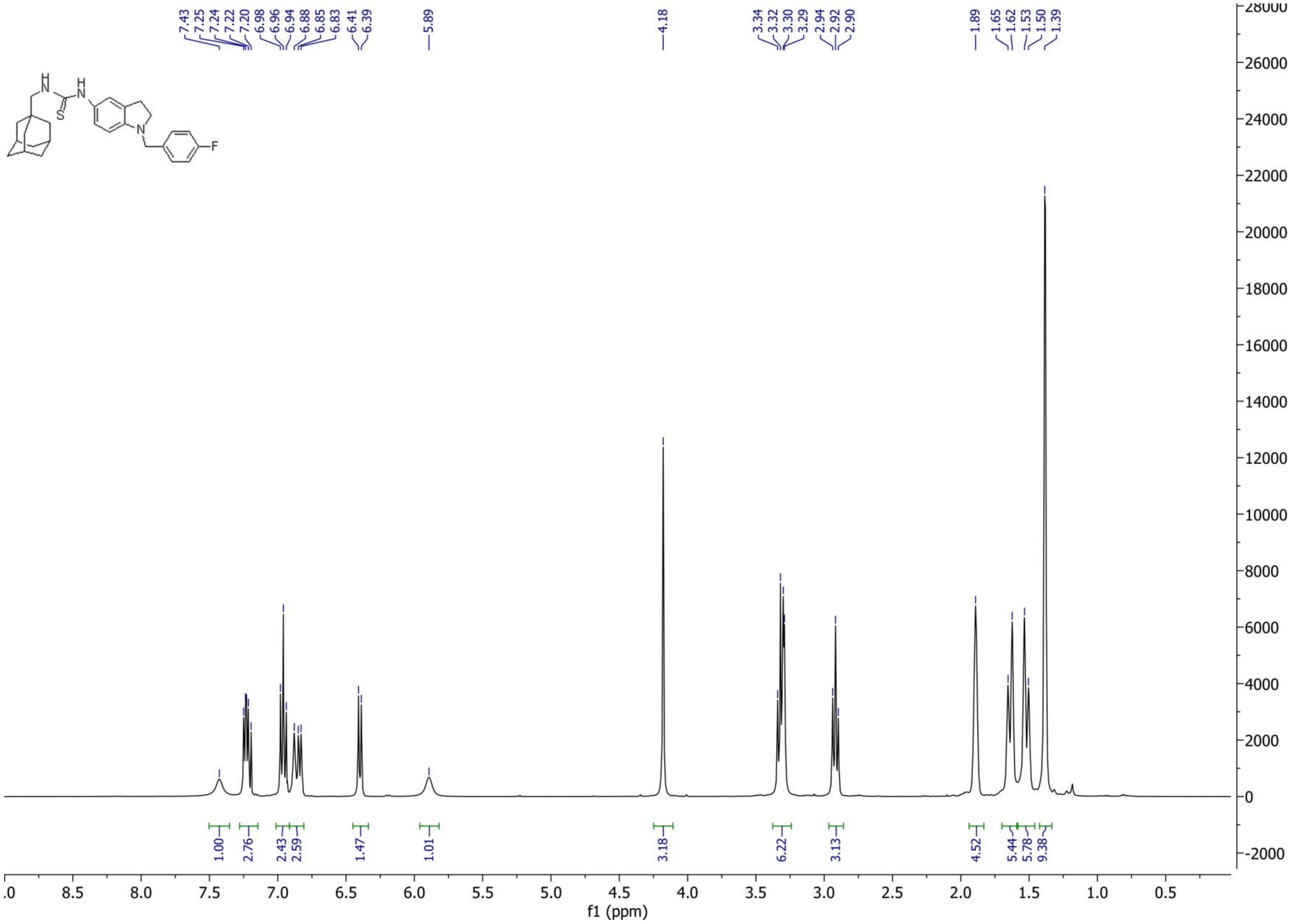

Figure S19: 1H NMR spectra of compound **10**

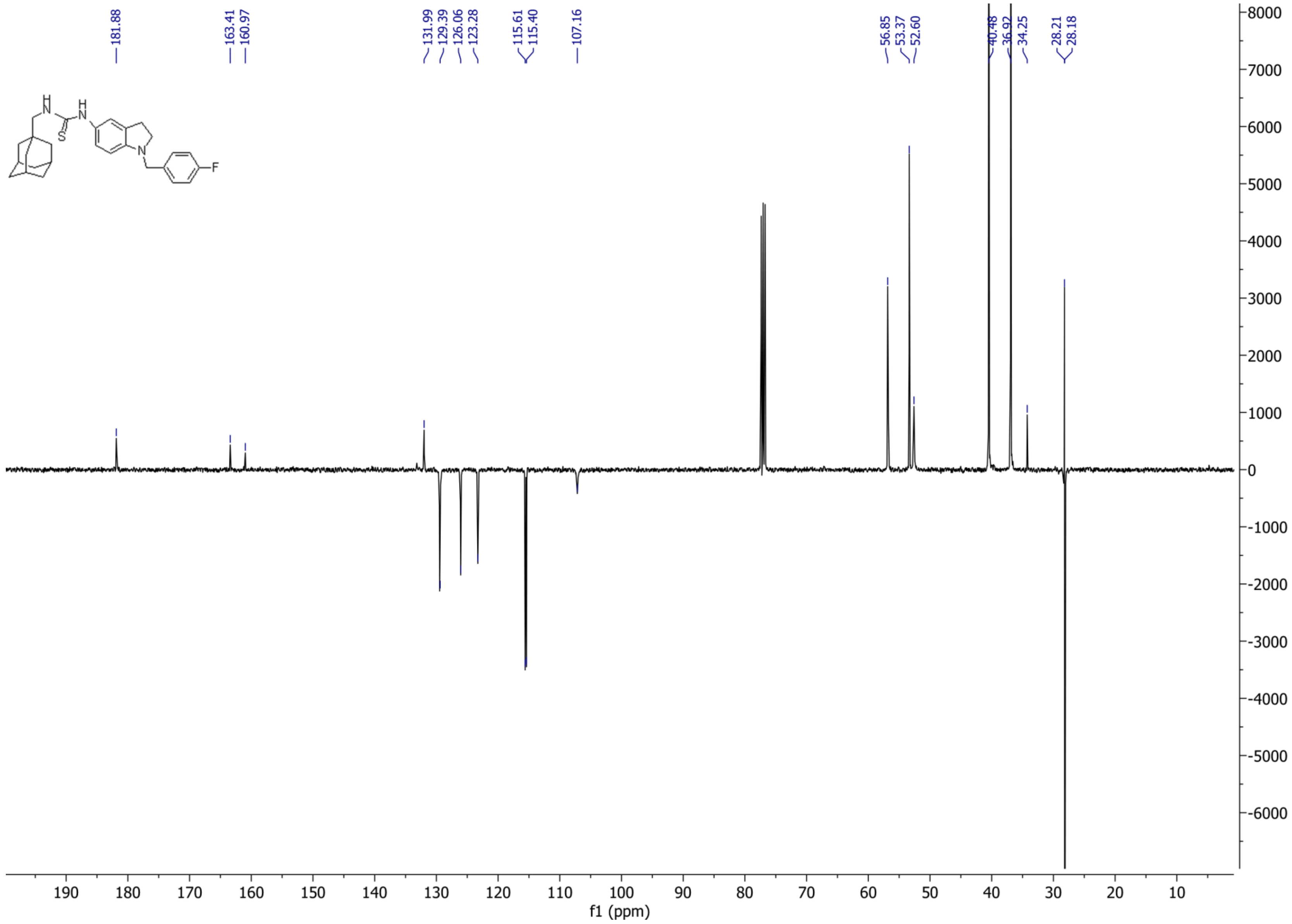

Figure S20: DEPT spectra of compound **10**

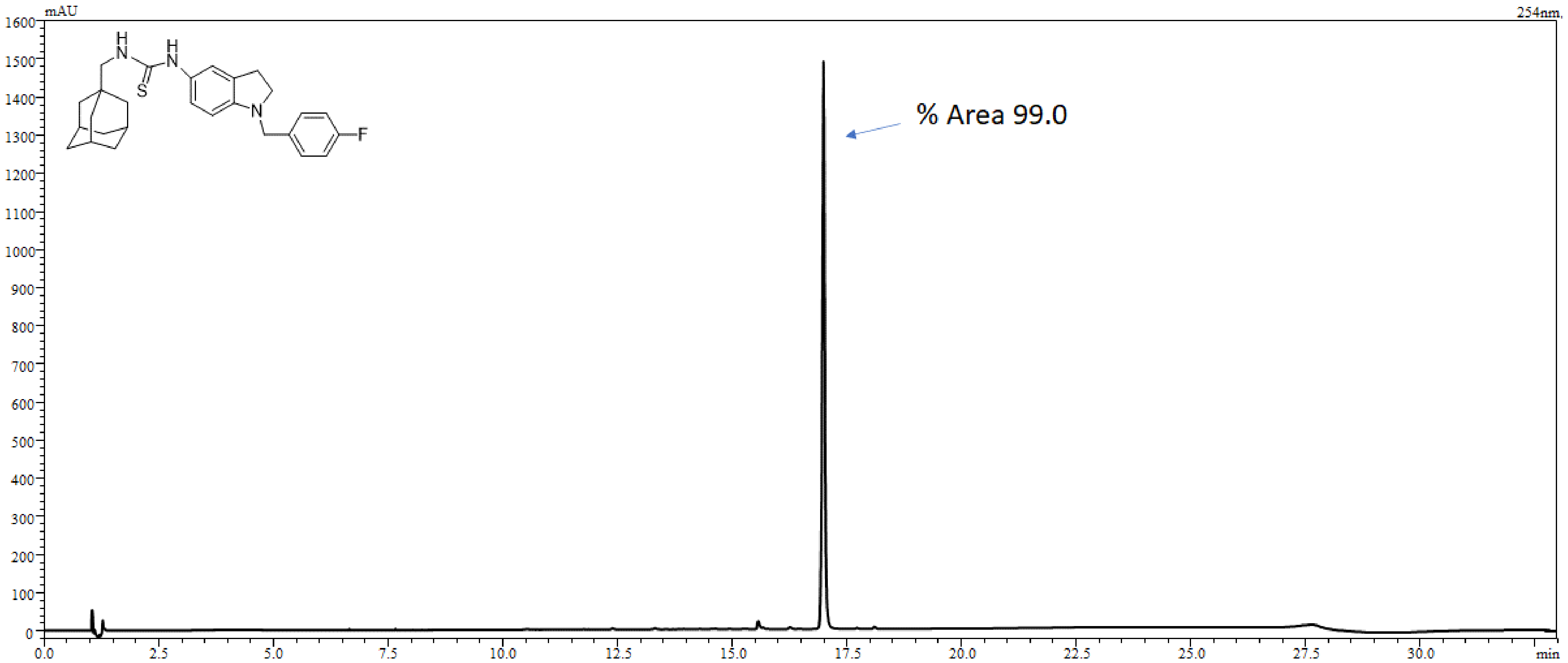

Figure S21: HPLC spectra of compound **10**

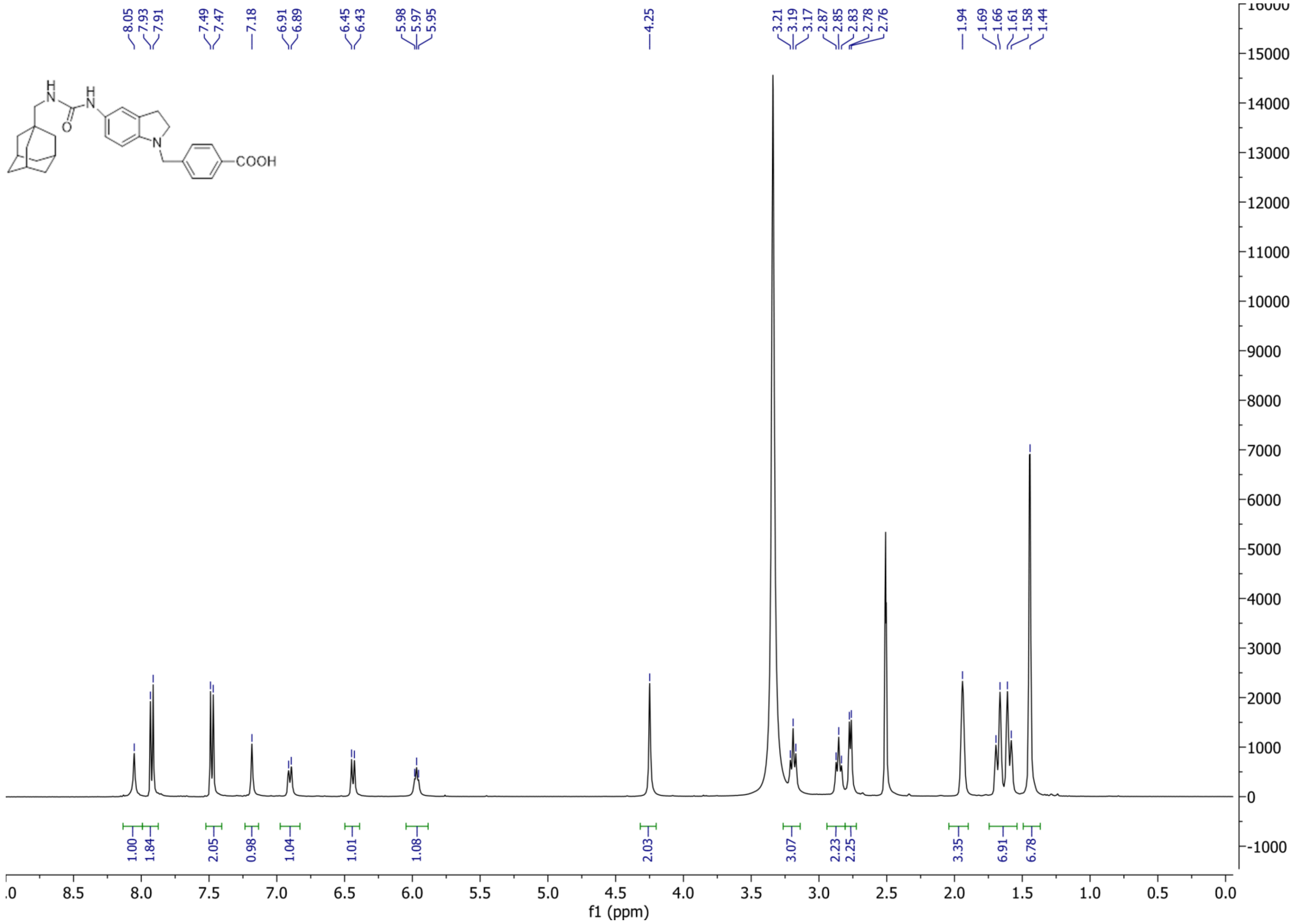

Figure S22: 1H NMR spectra of compound **14**

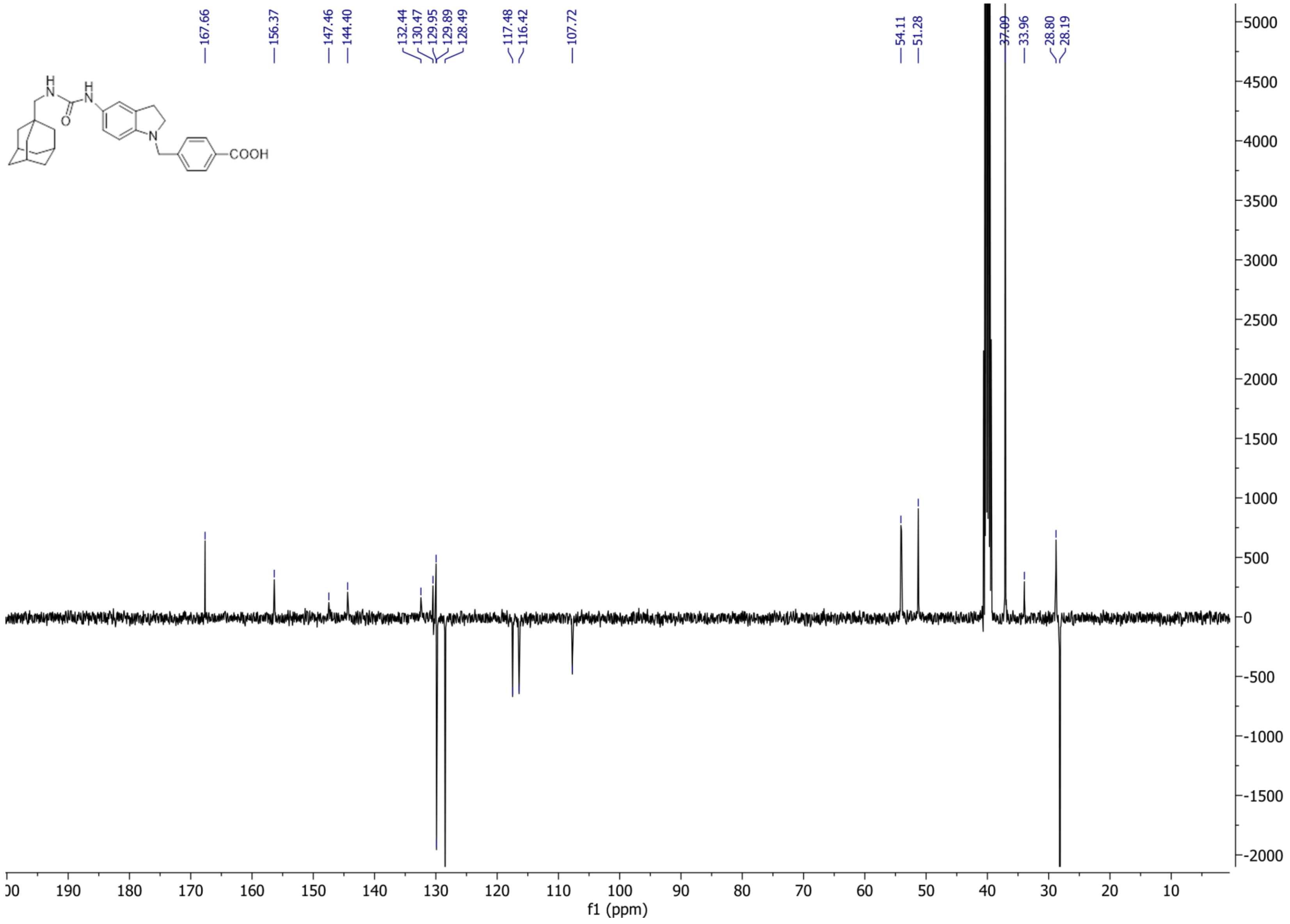

Figure S23: DEPT spectra of compound **14**

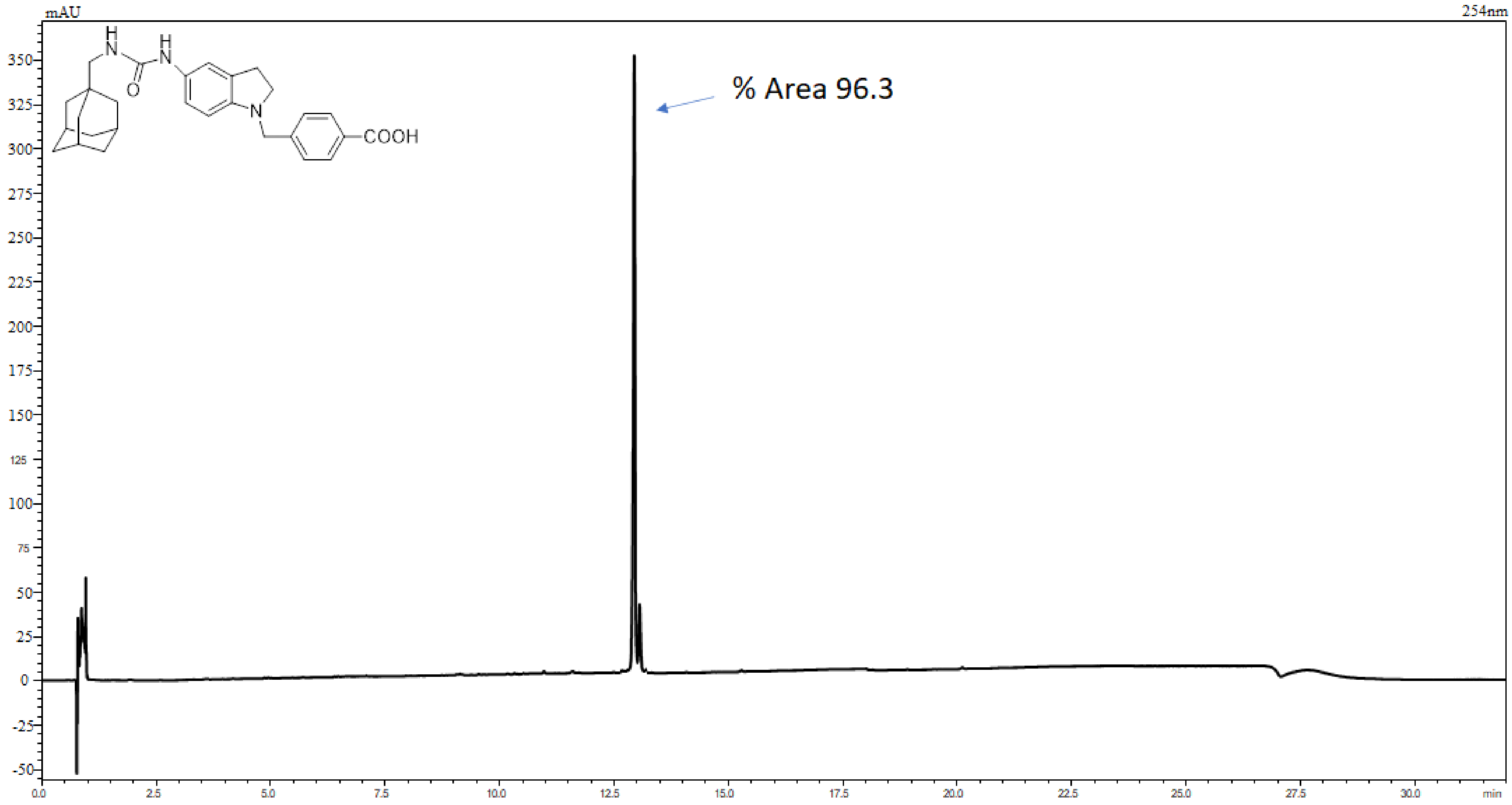

Figure S24: HPLC spectra of compound **14**

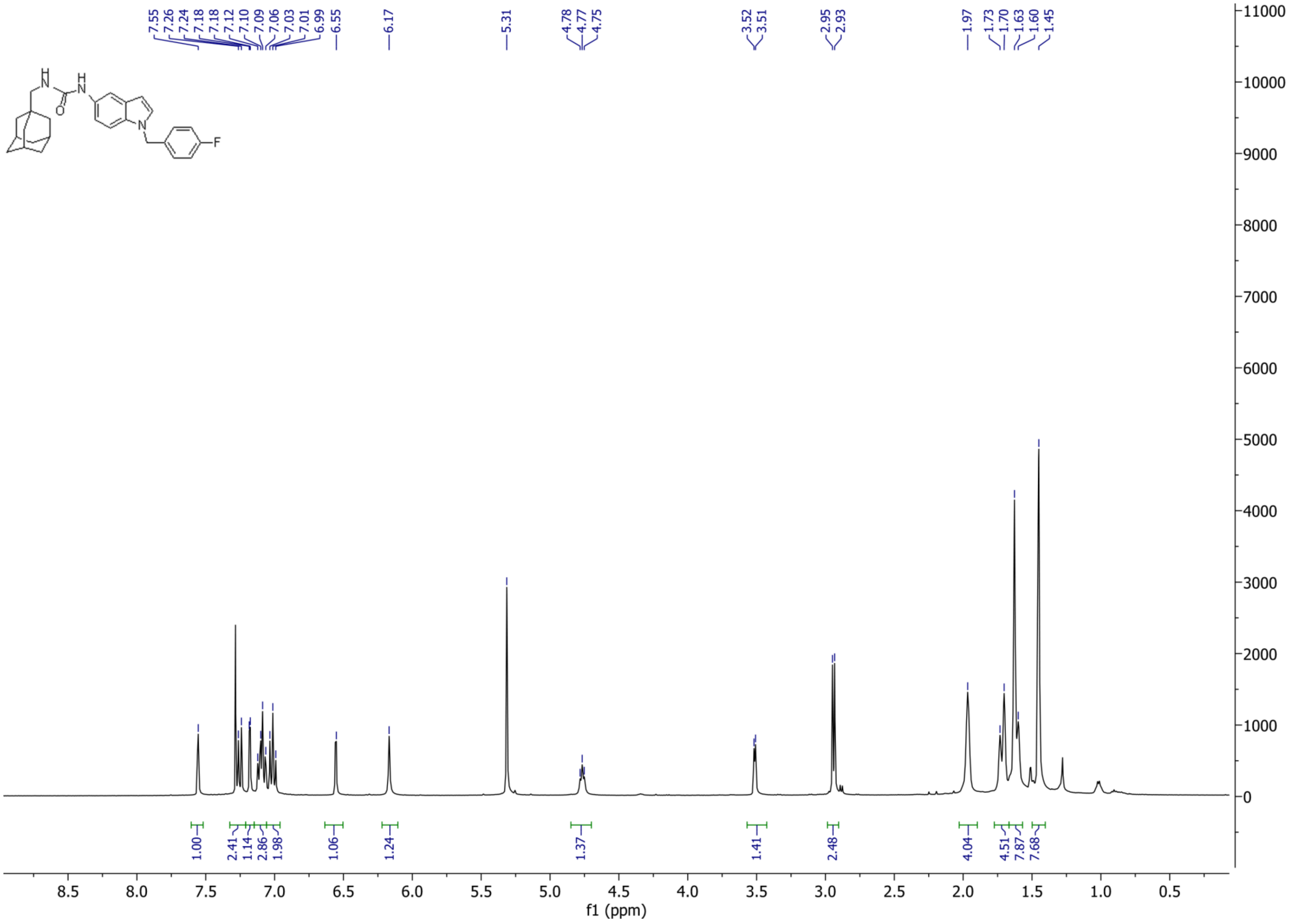

Figure S25: 1H NMR spectra of compound **27**

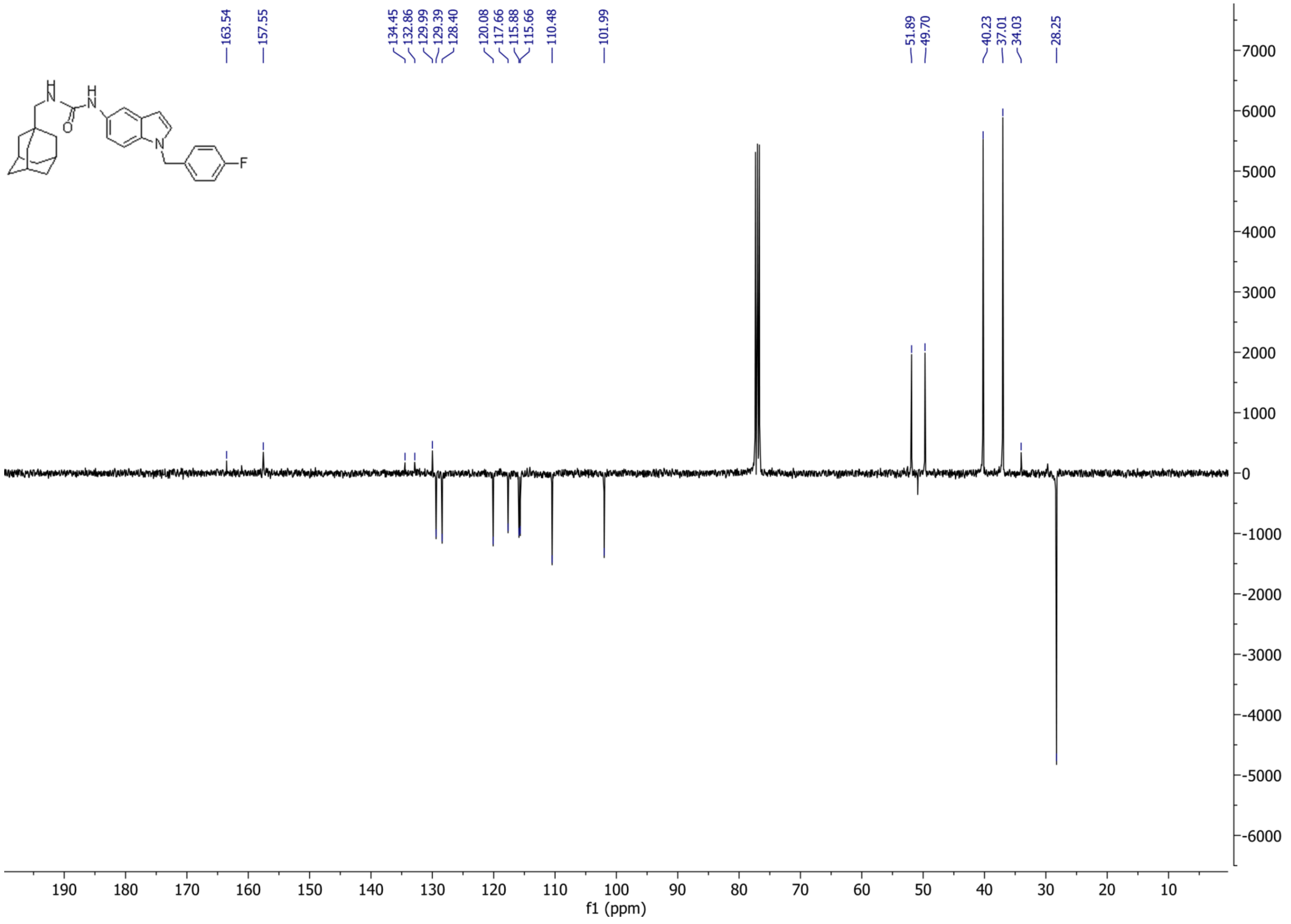

Figure S26: DEPT spectra of compound **27**

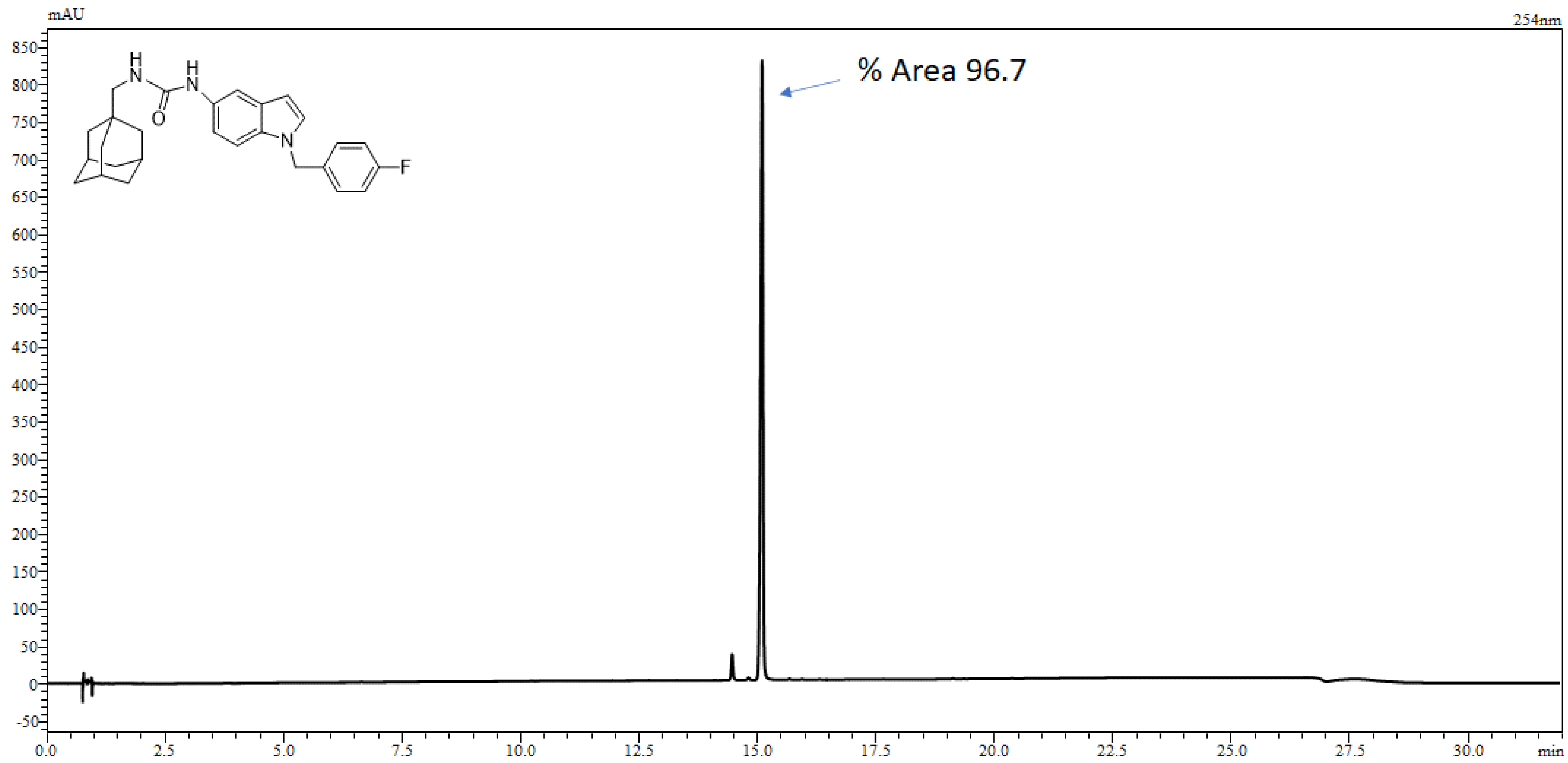

Figure S27: HPLC spectra of compound **27**

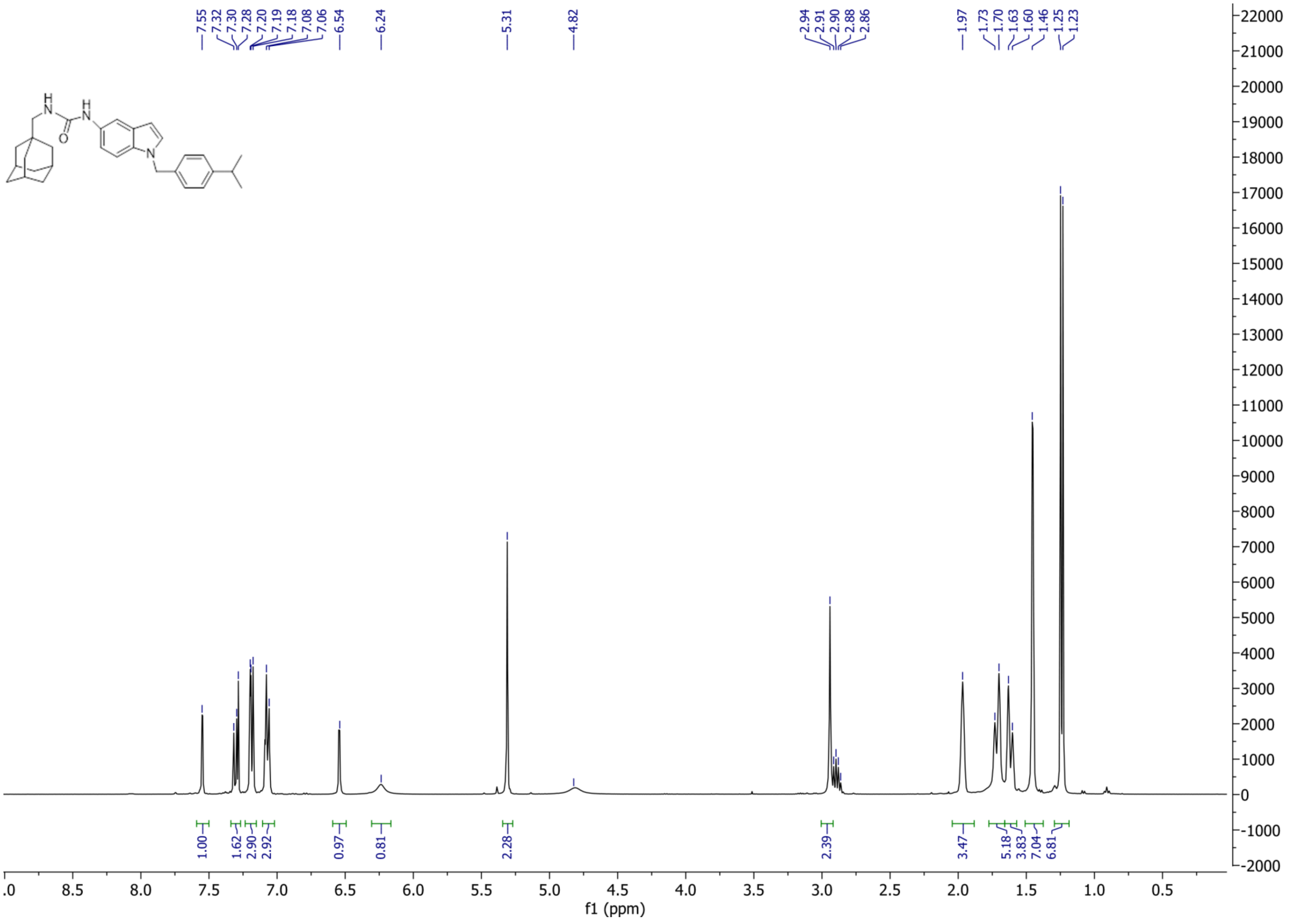

Figure S28: 1H NMR spectra of compound **28**

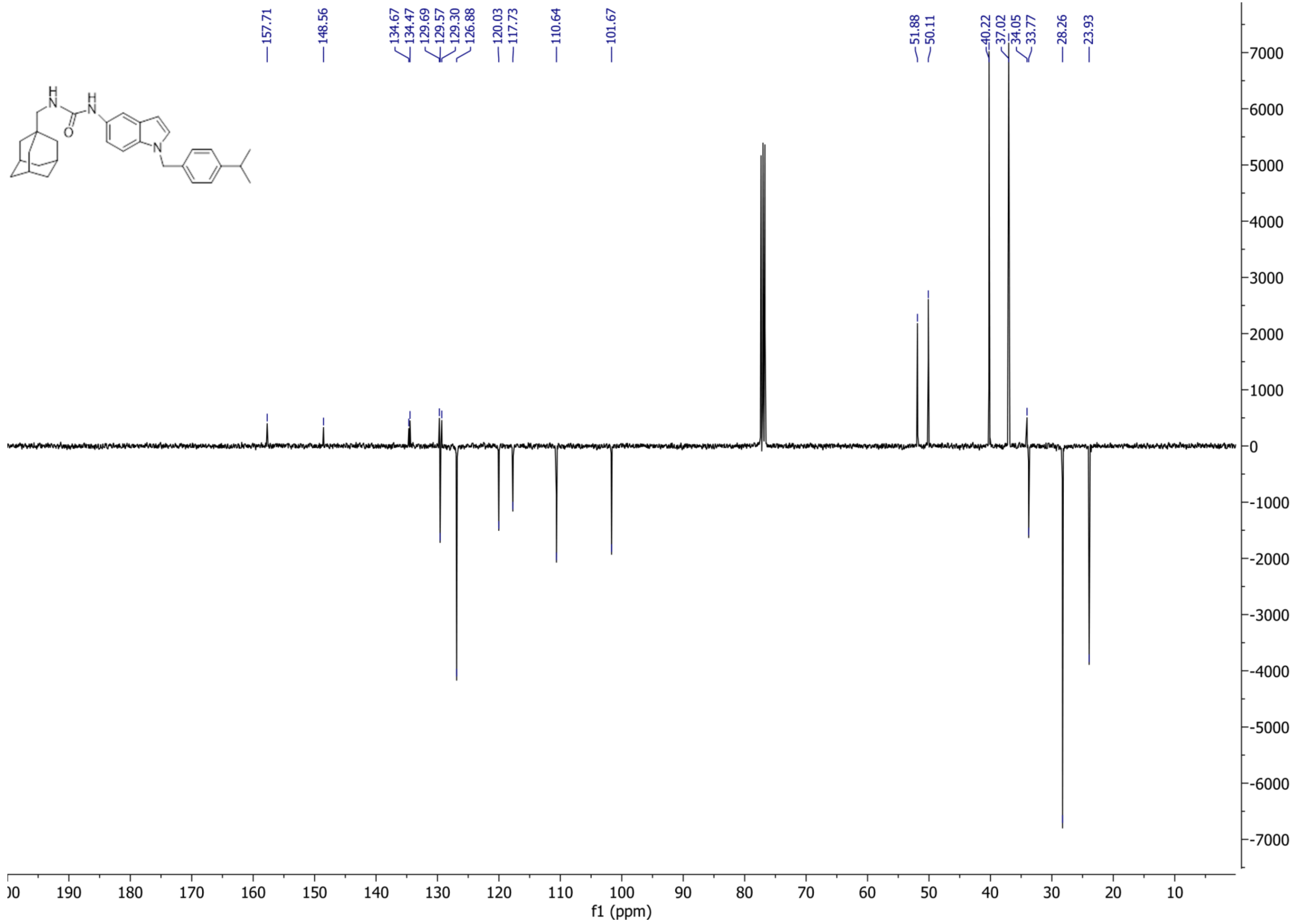

Figure S29: DEPT spectra of compound **28**

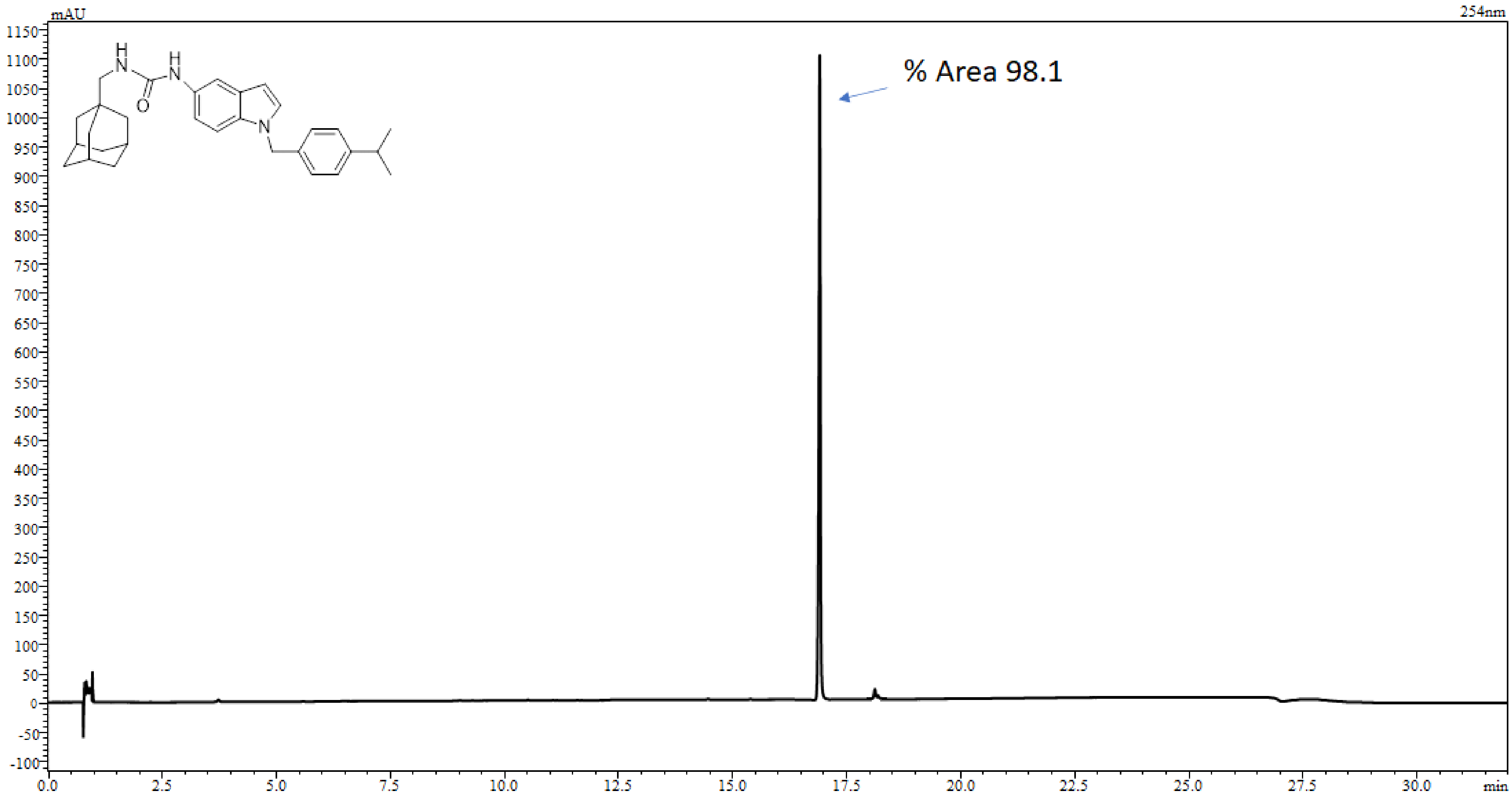

Figure S30: HPLC spectra of compound **28**

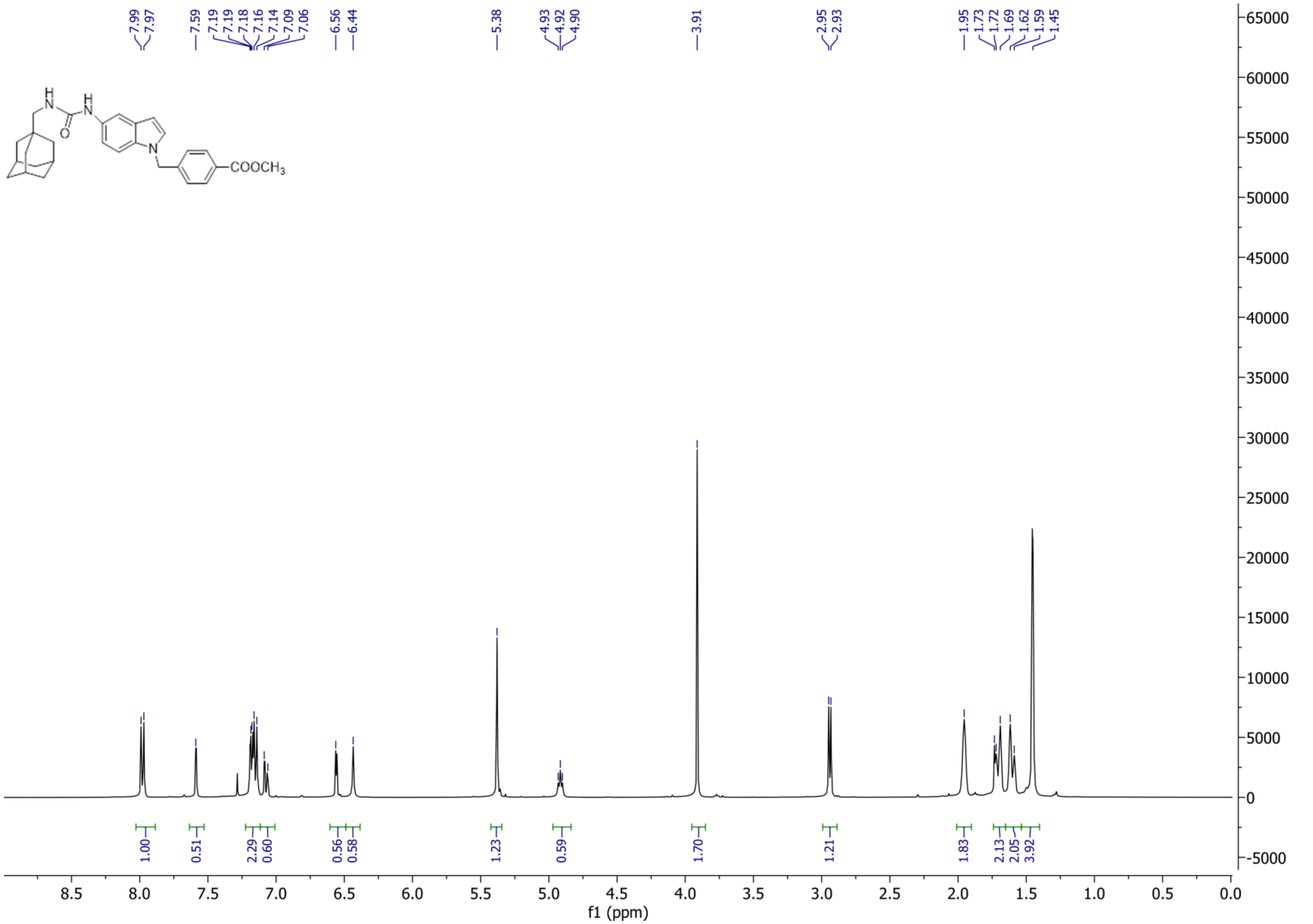

Figure S31: 1H NMR spectra of compound **29**

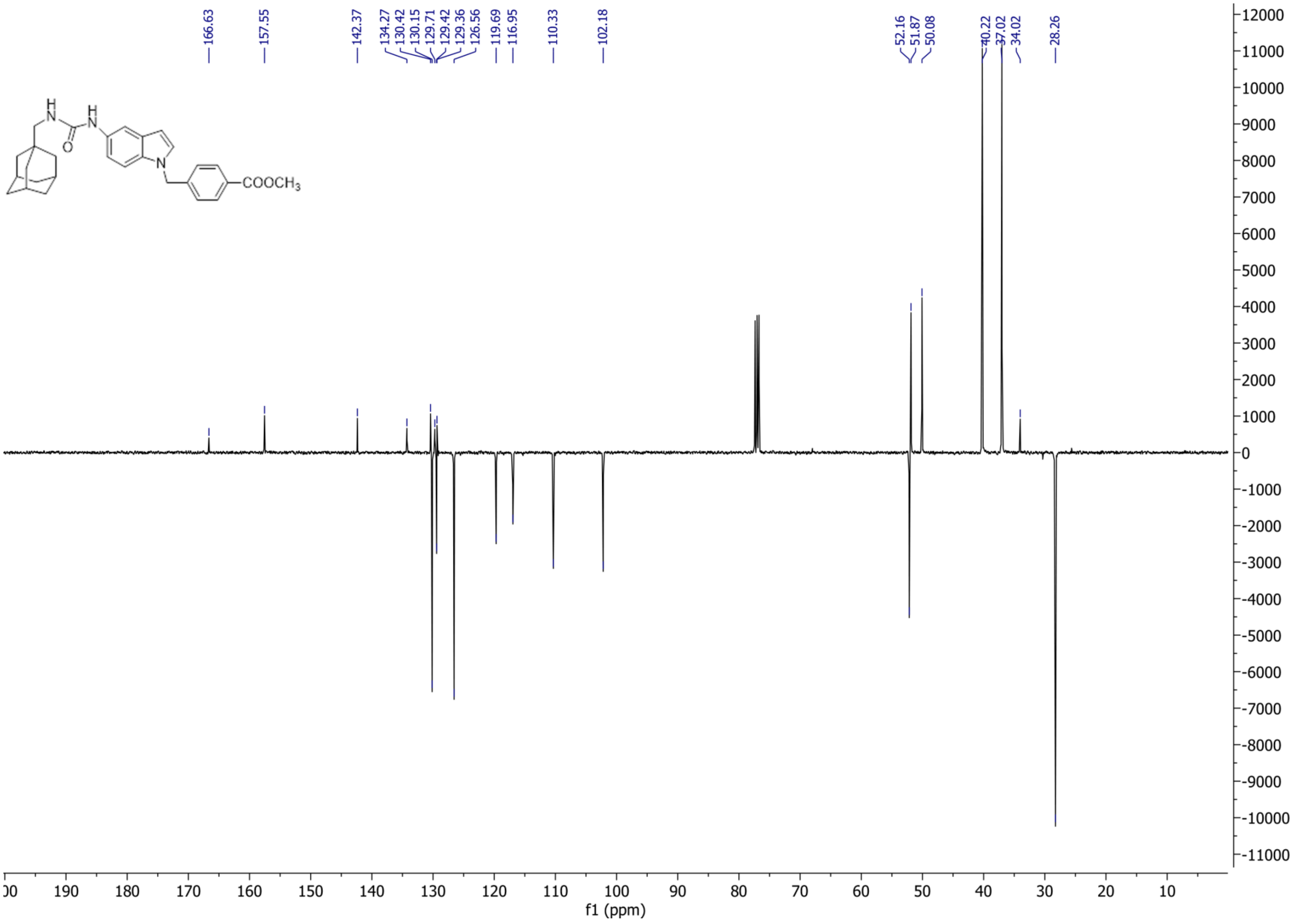

Figure S32: DEPT spectra of compound **29**

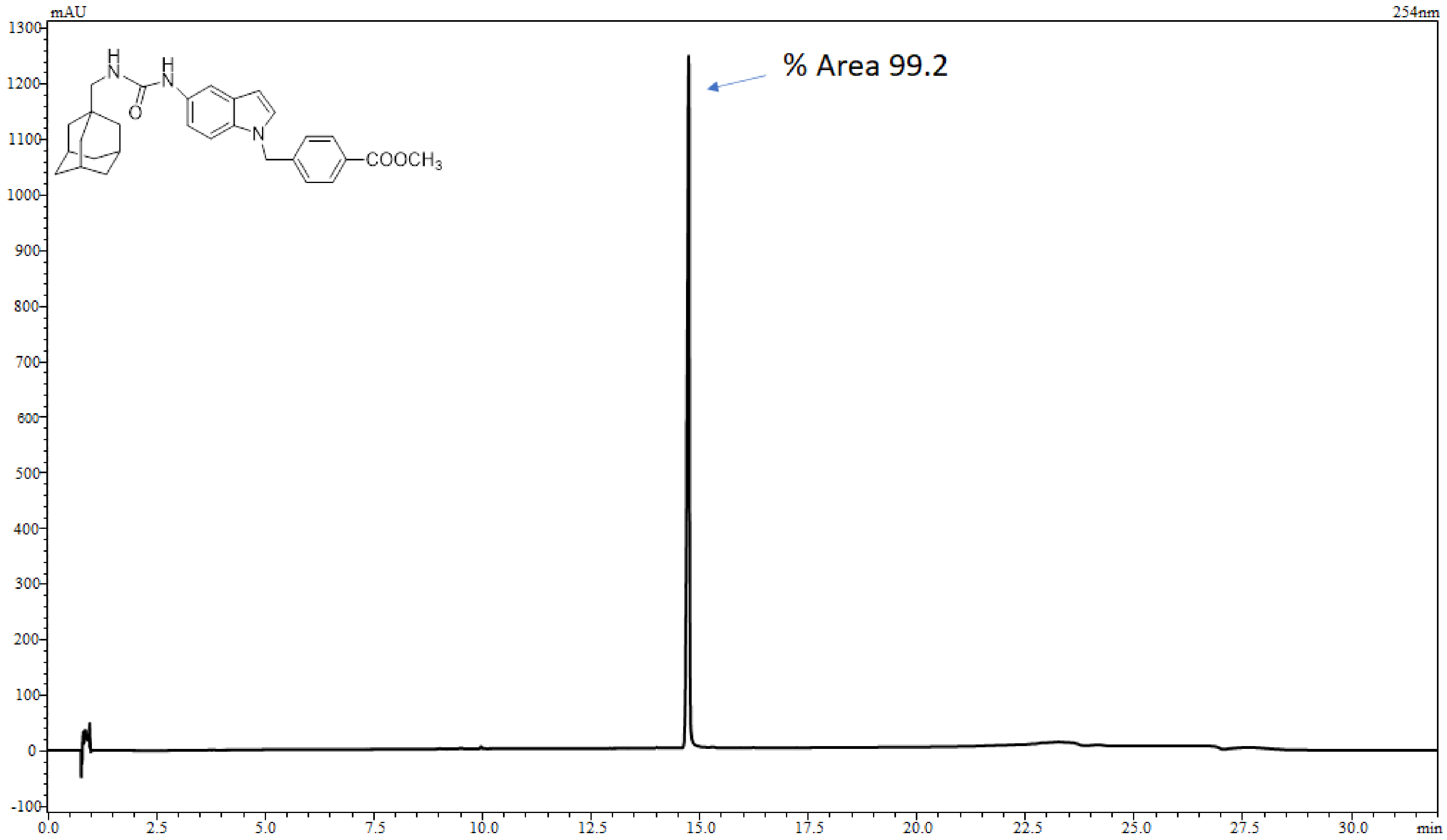

Figure S33: HPLC spectra of compound **29**

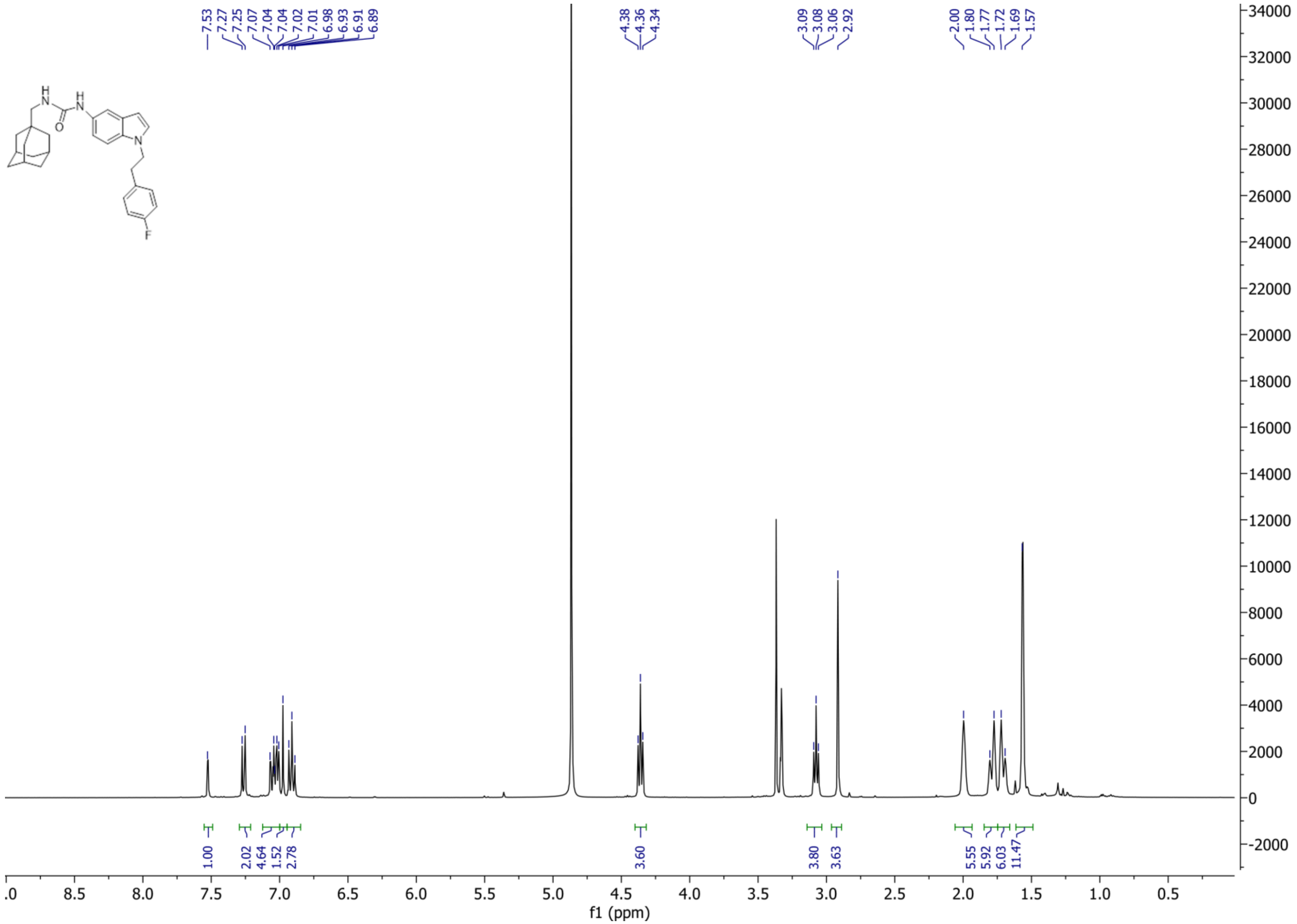

Figure S34: 1H NMR spectra of compound **30**

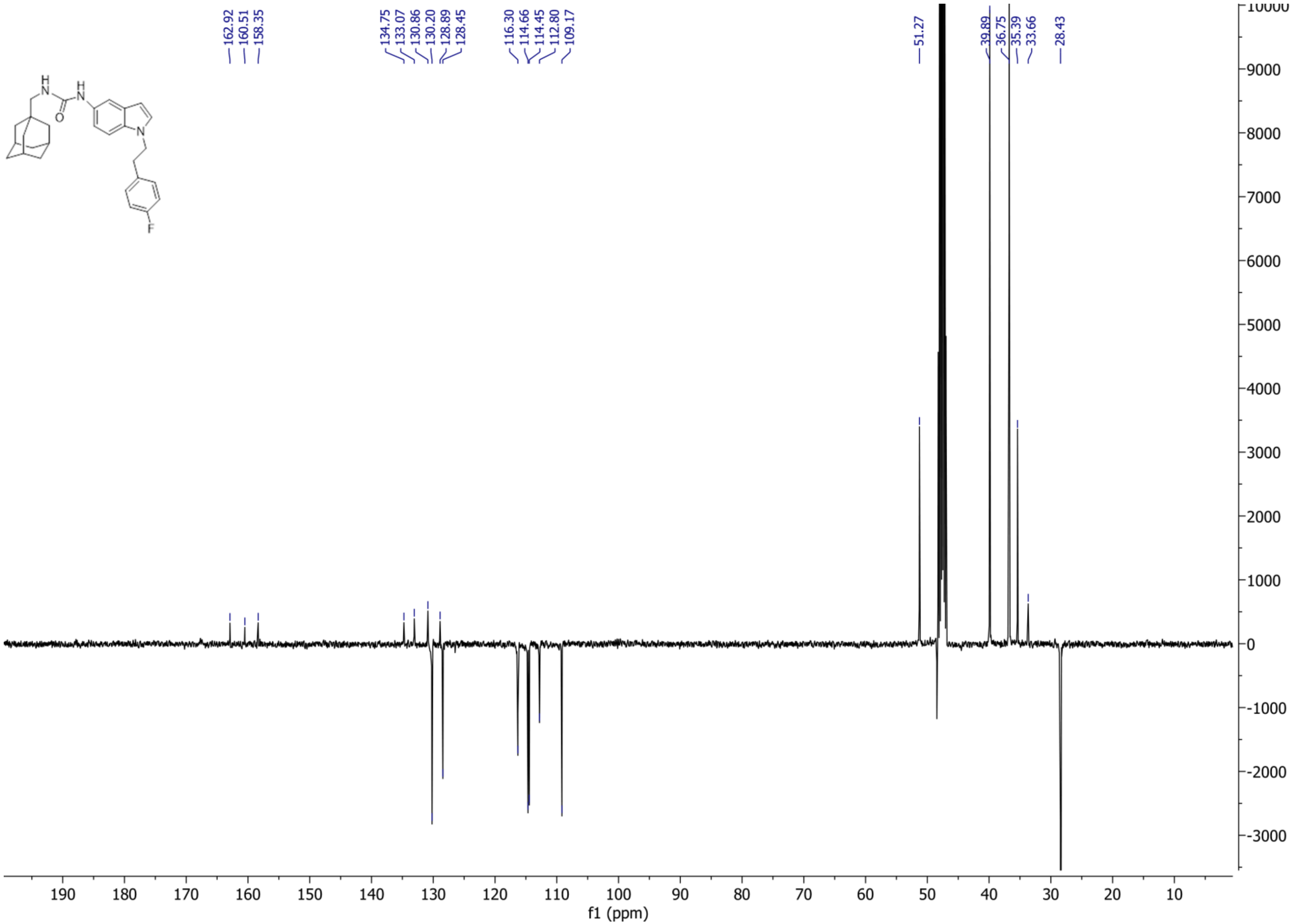

Figure S35: DEPT spectra of compound **30**

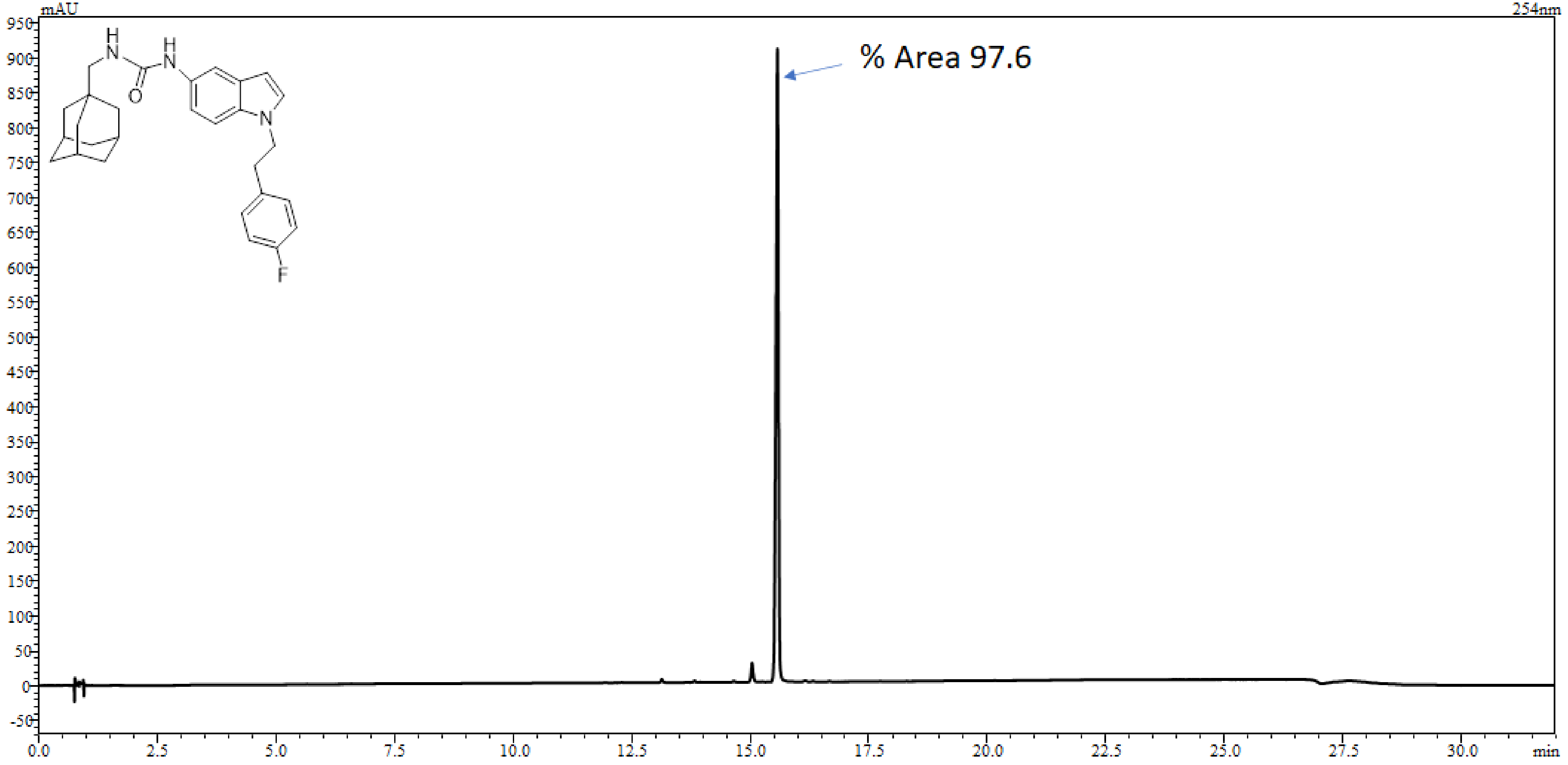

Figure S36: HPLC spectra of compound **30**

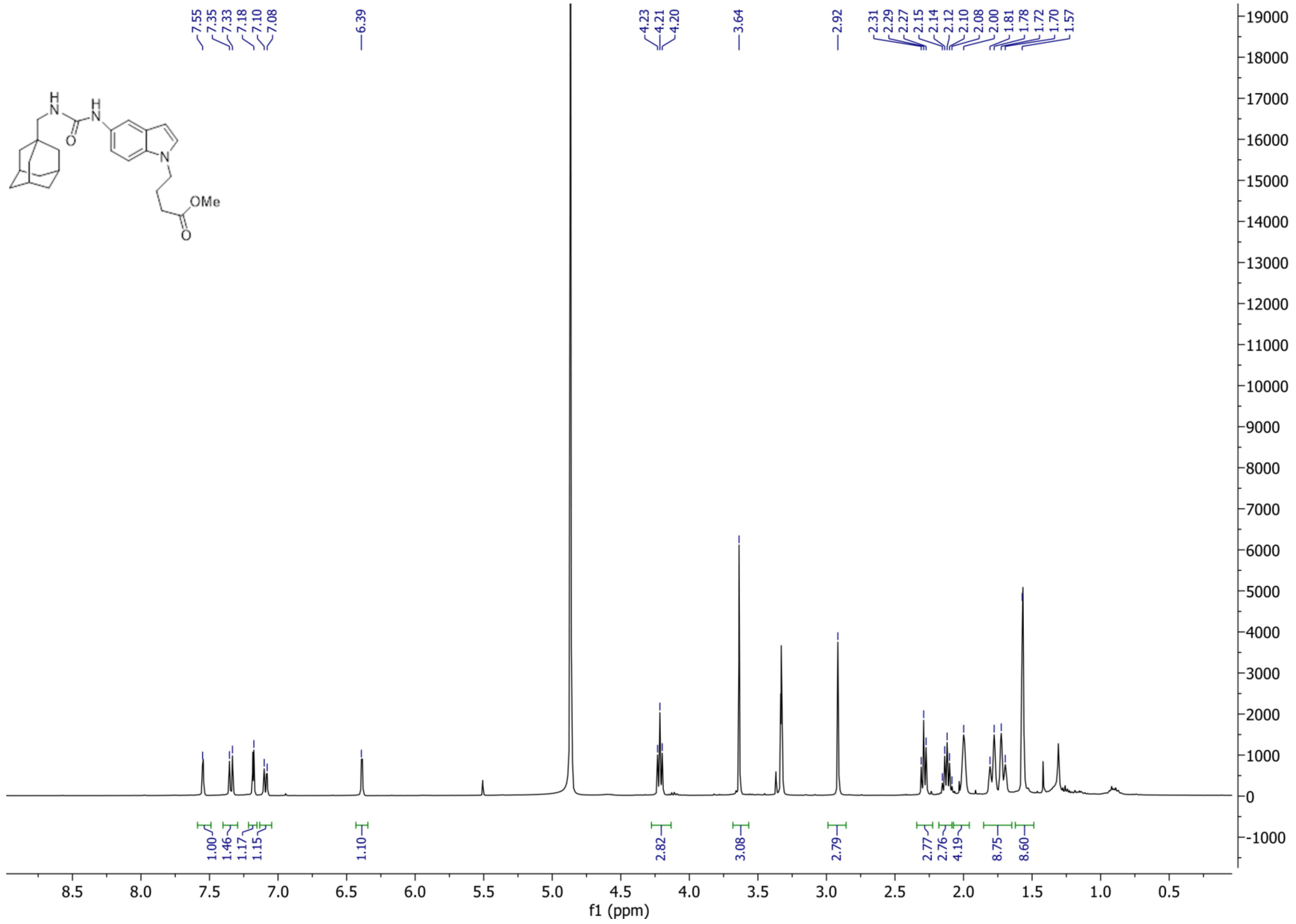

Figure S37: 1H NMR spectra of compound **32**

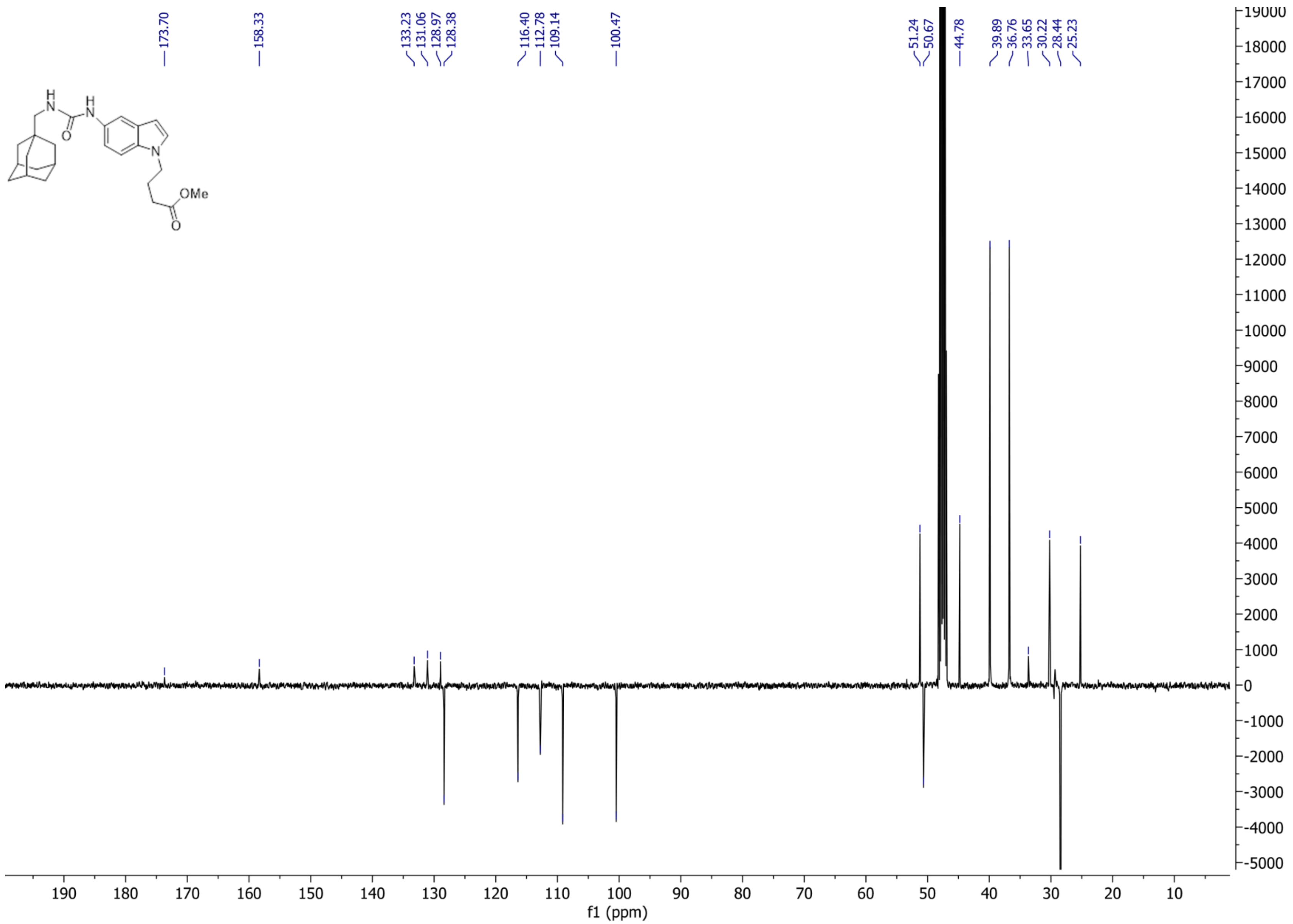

Figure S38: DEPT spectra of compound **32**

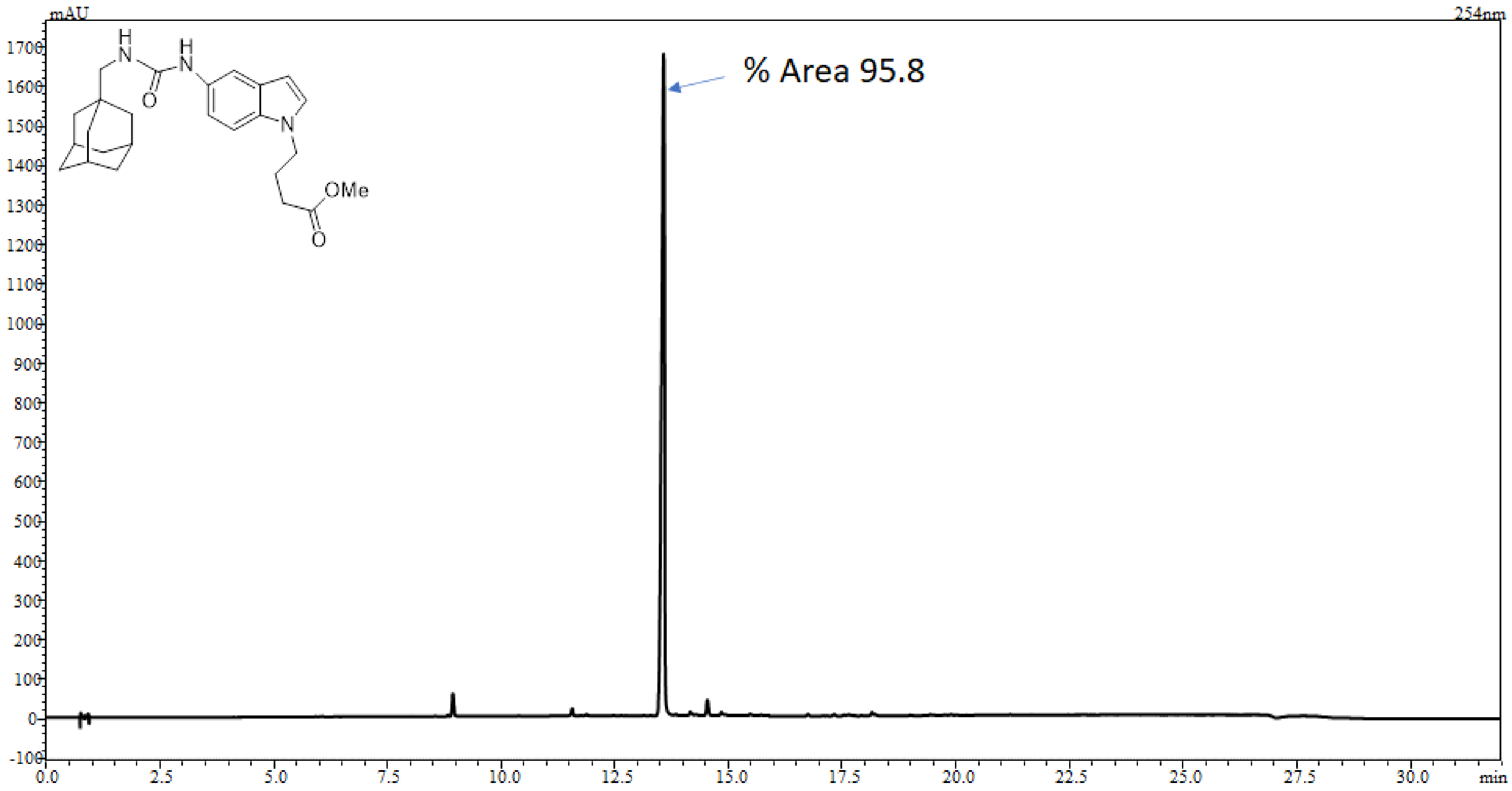

Figure S39: HPLC spectra of compound **32**

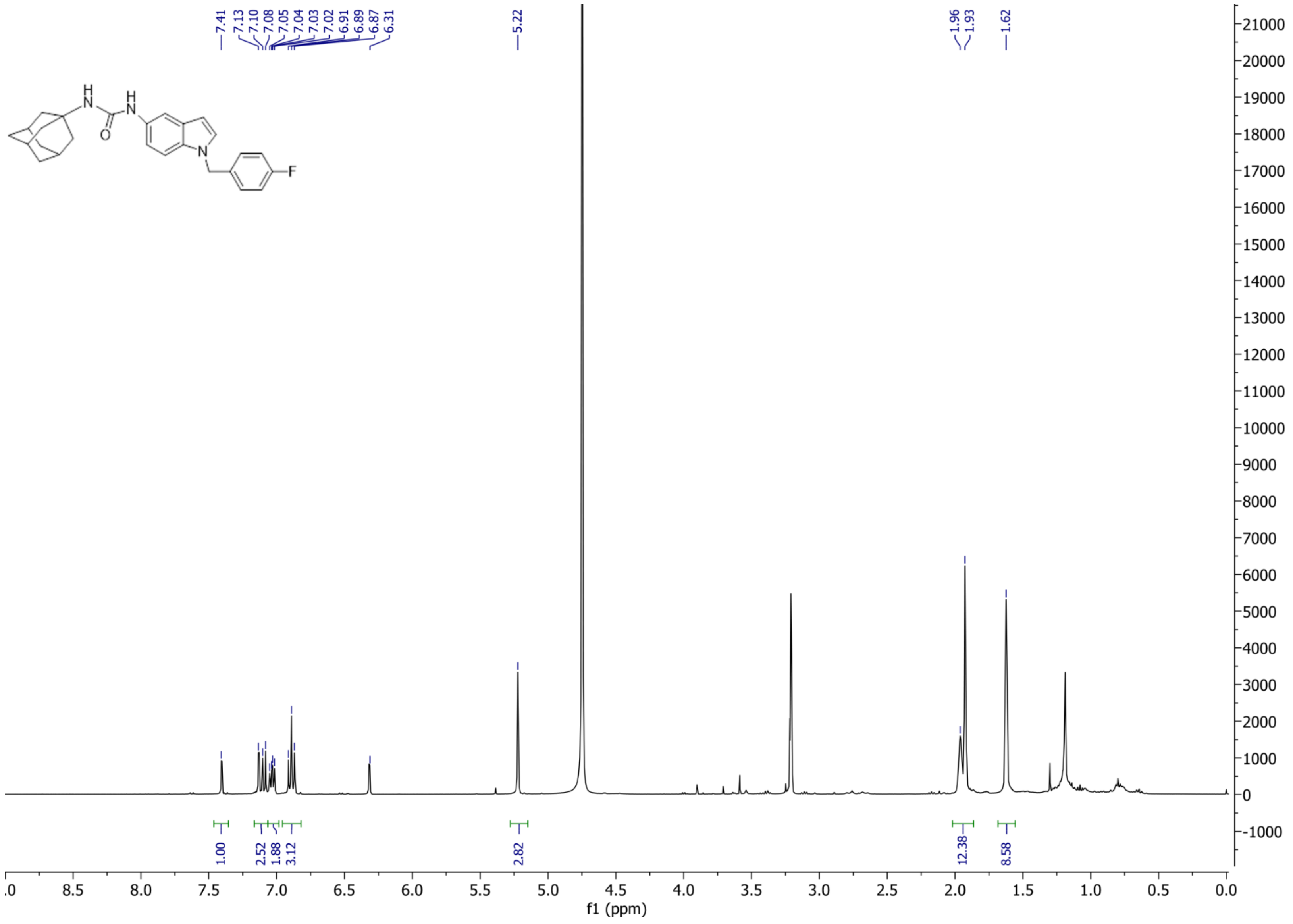

Figure S40: 1H NMR spectra of compound **33**

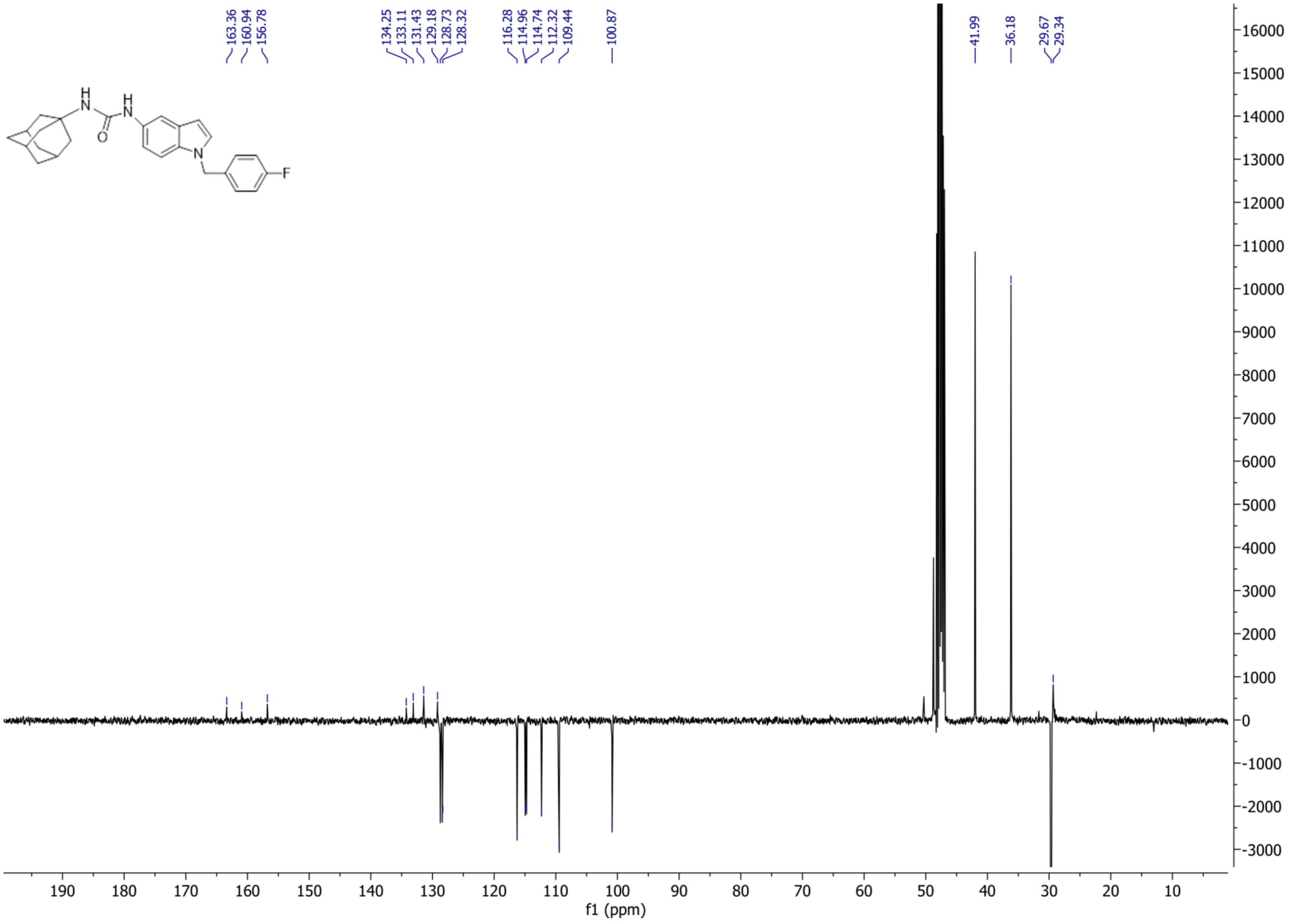

Figure S41: DEPT spectra of compound **33**

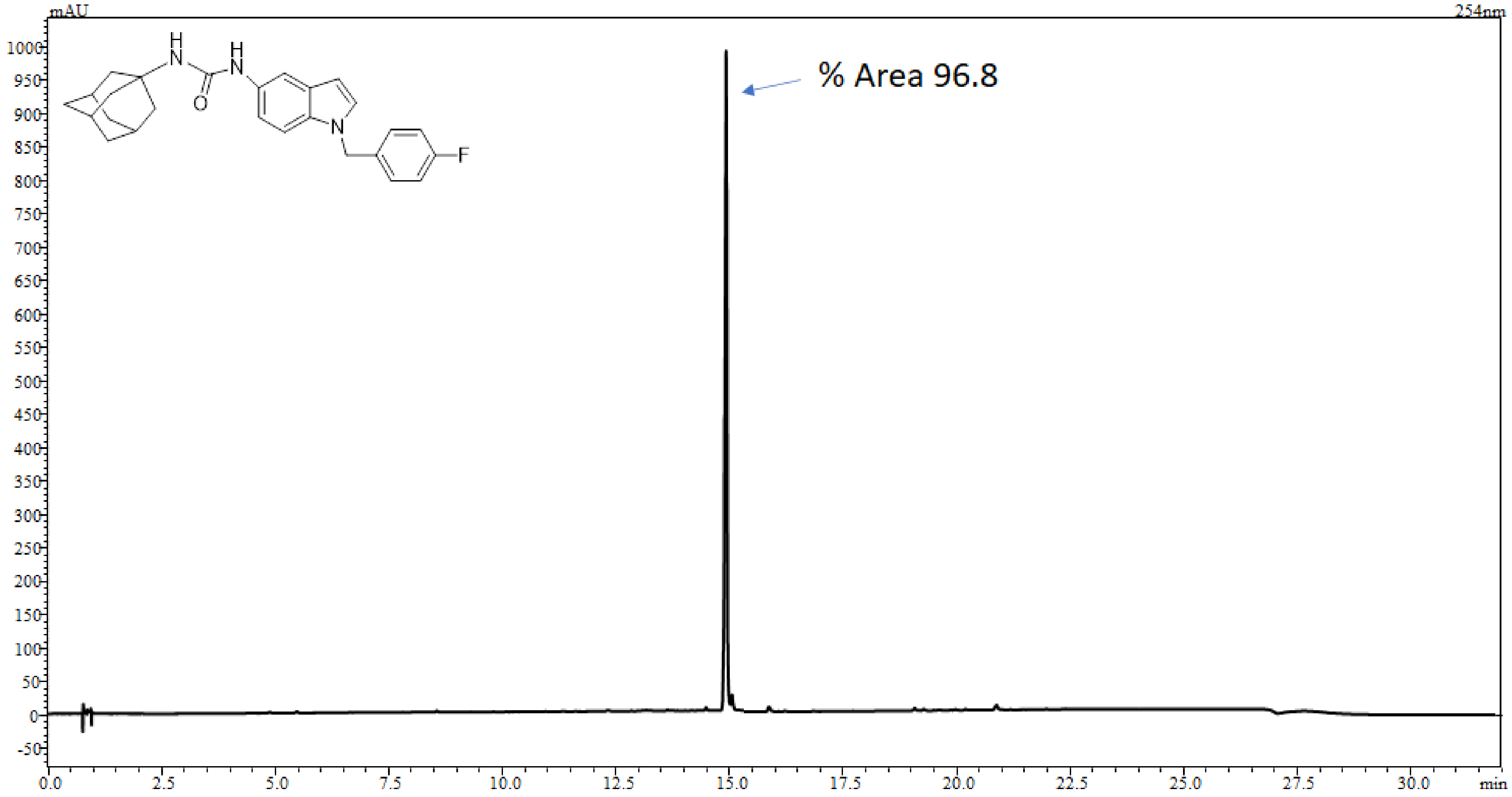

Figure S42: HPLC spectra of compound **33**

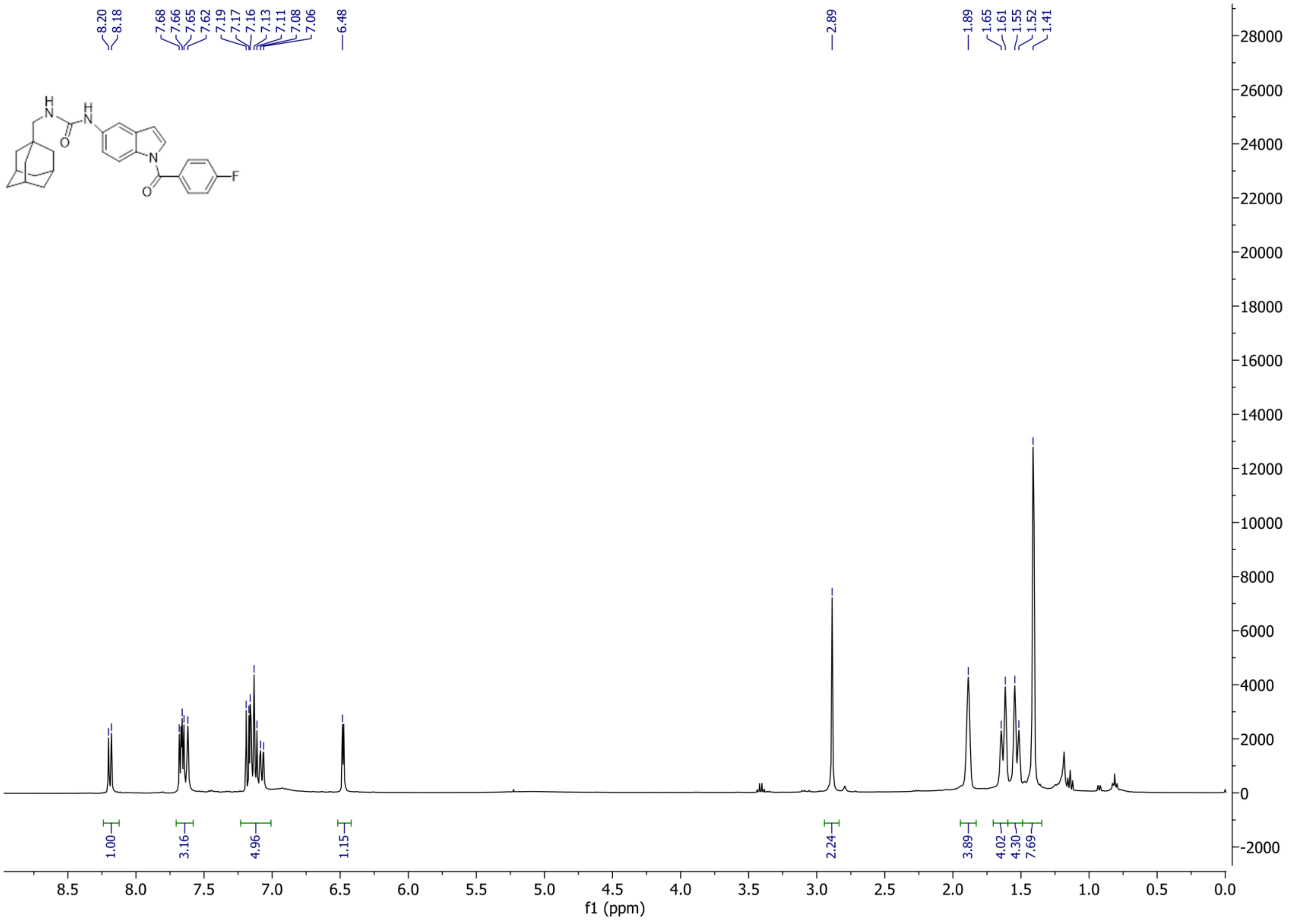

Figure S43: 1H NMR spectra of compound **36**

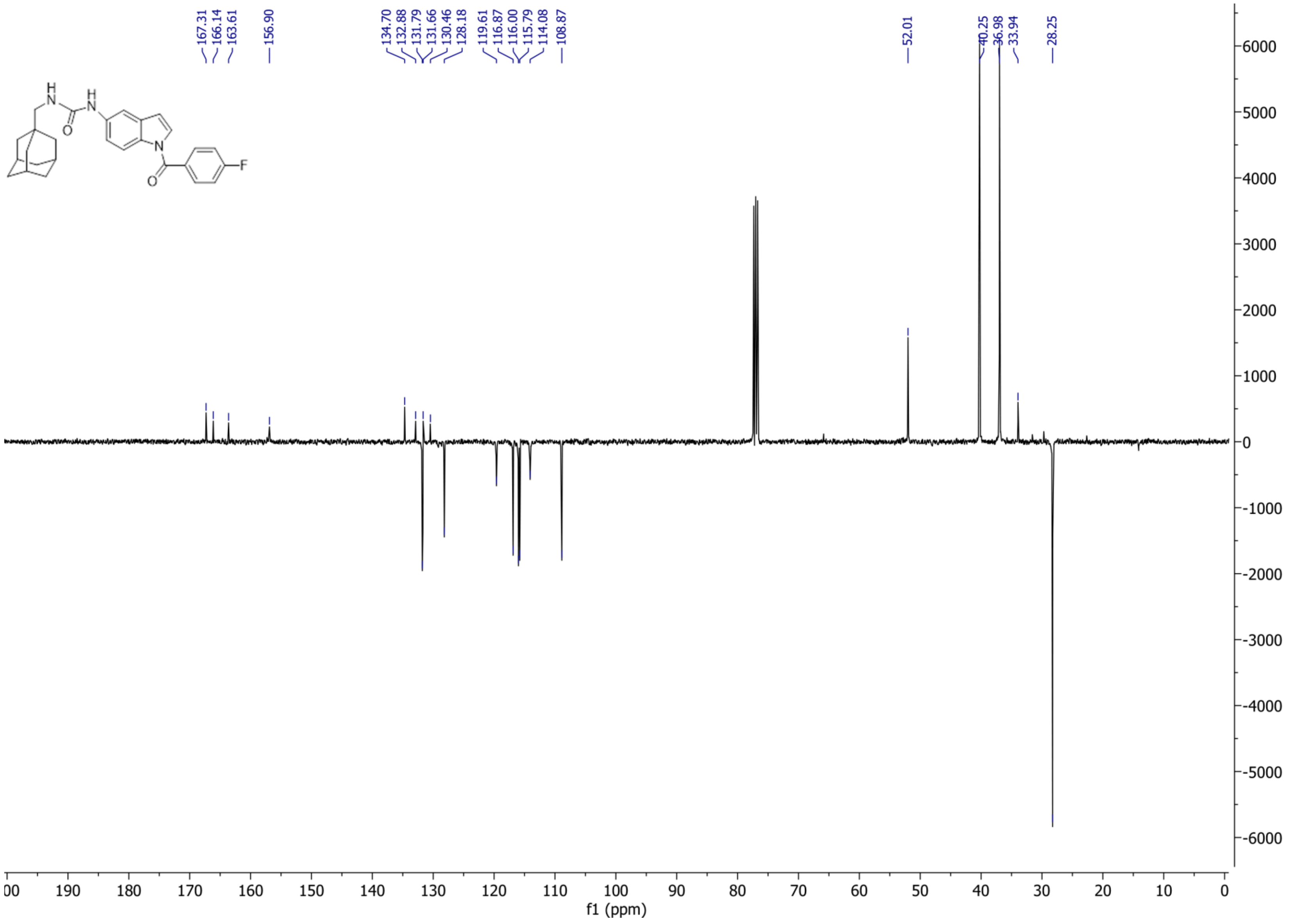

Figure S44: DEPT spectra of compound **36**

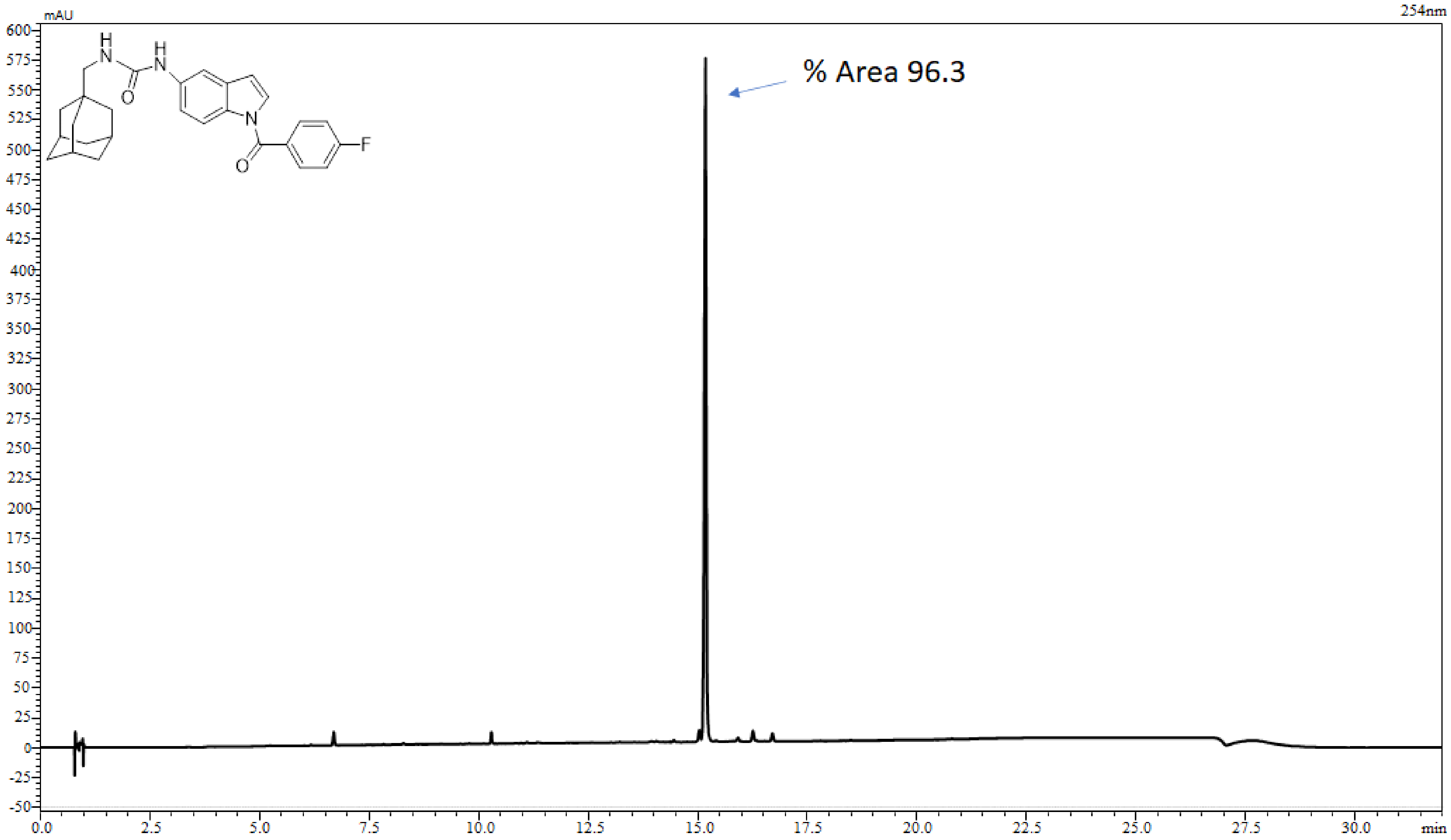

Figure S45: HPLC spectra of compound **36**

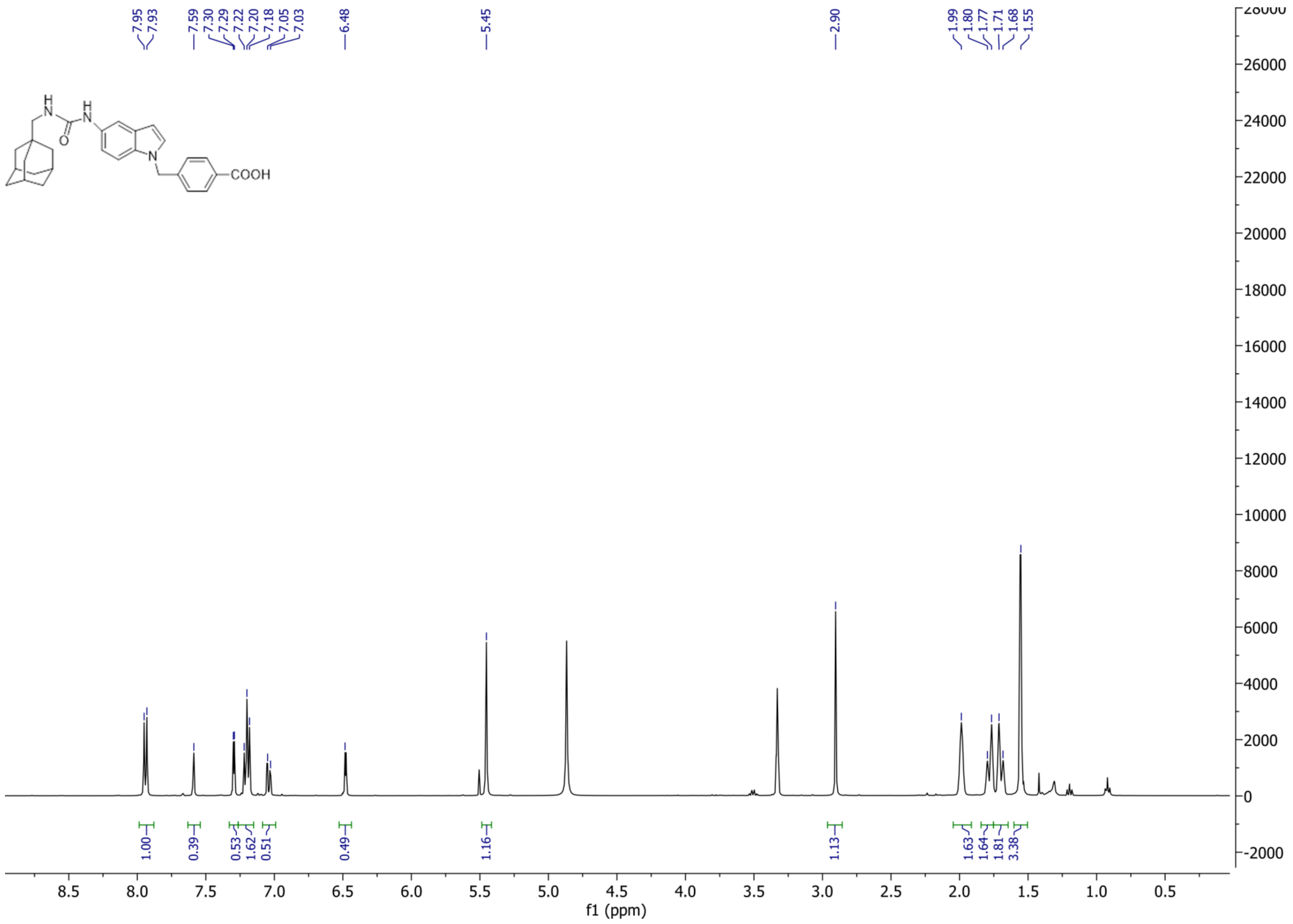

Figure S46: 1H NMR spectra of compound **37**

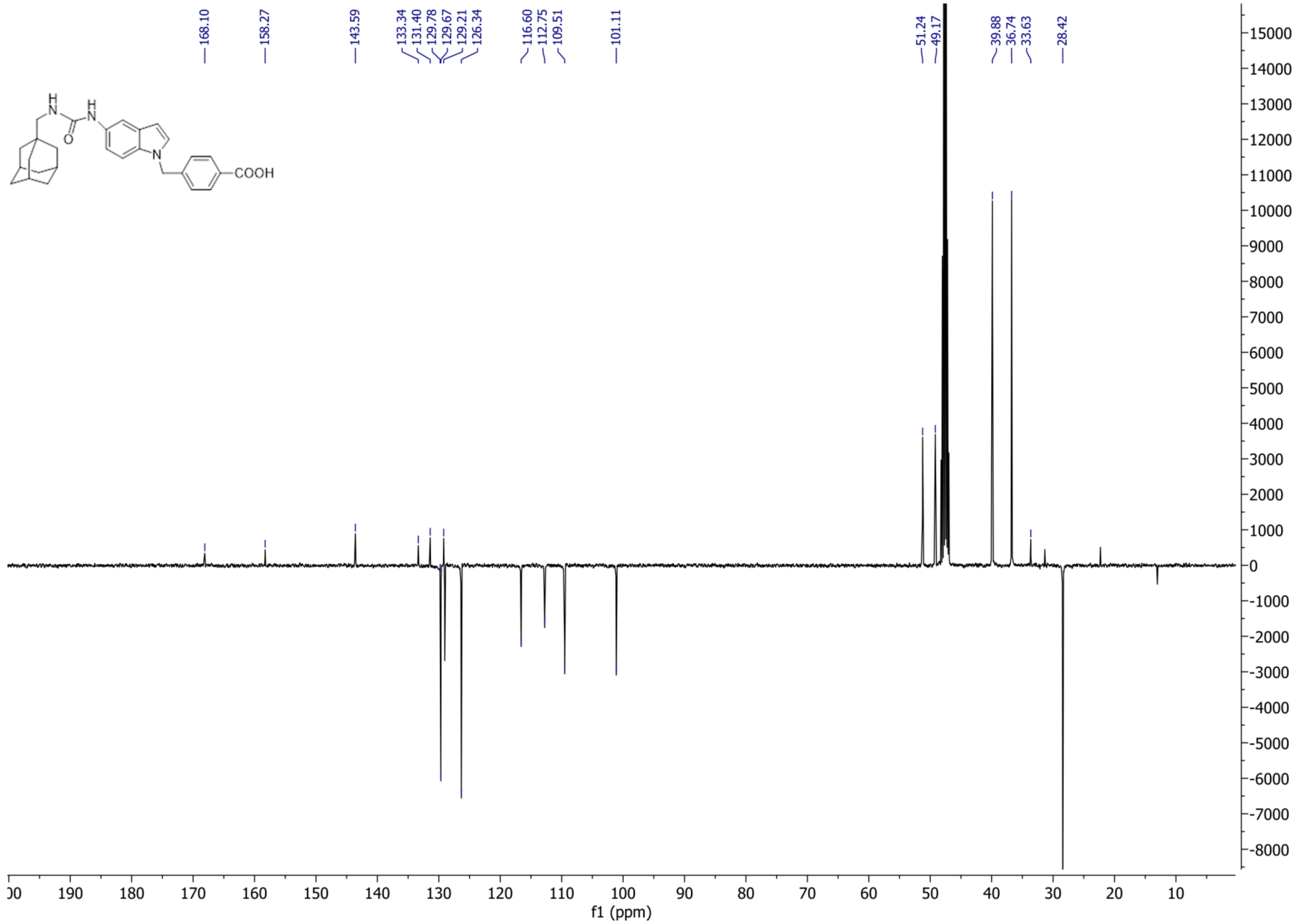

Figure S47: DEPT spectra of compound **37**

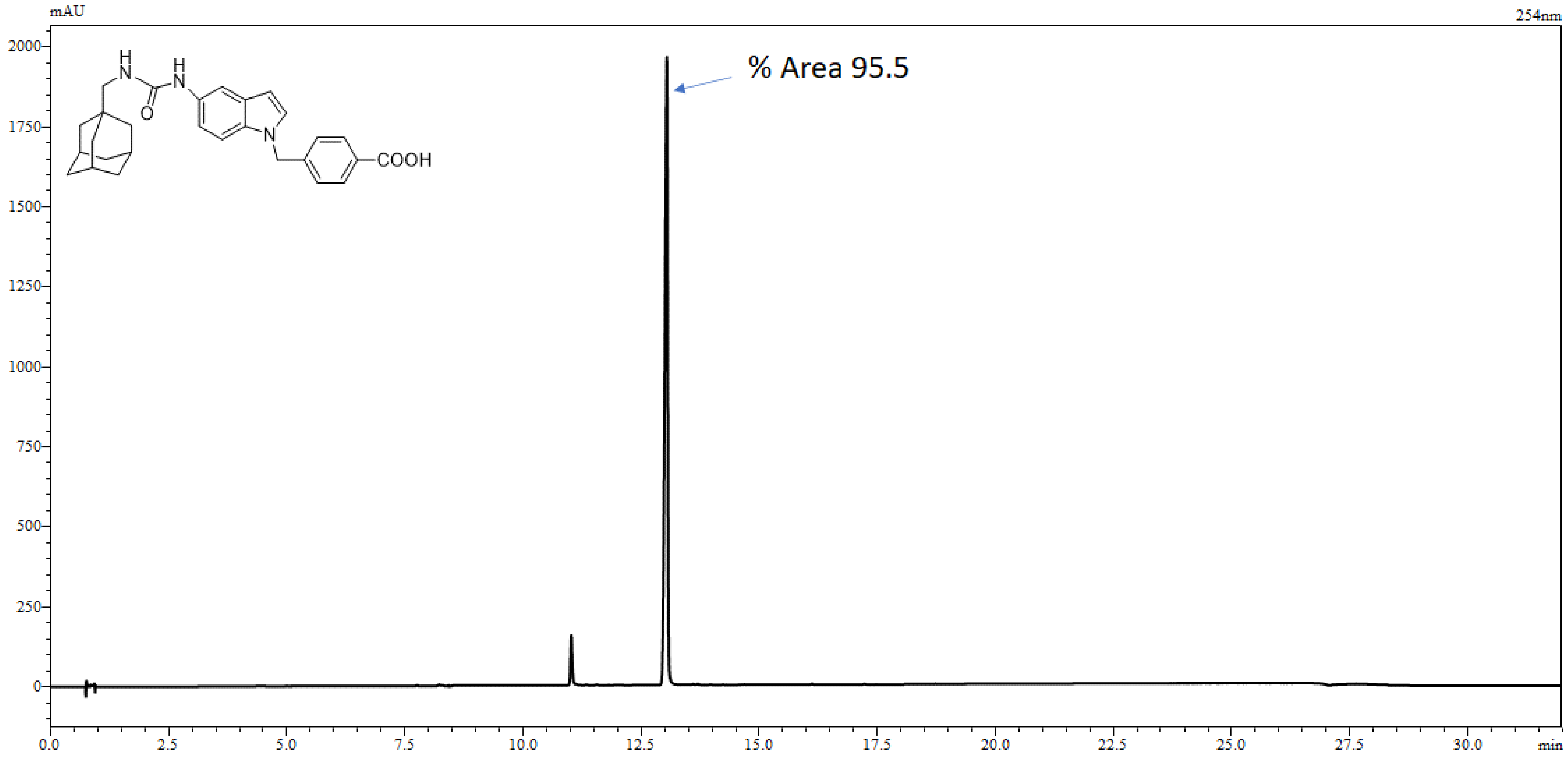

Figure S48: HPLC spectra of compound **37**

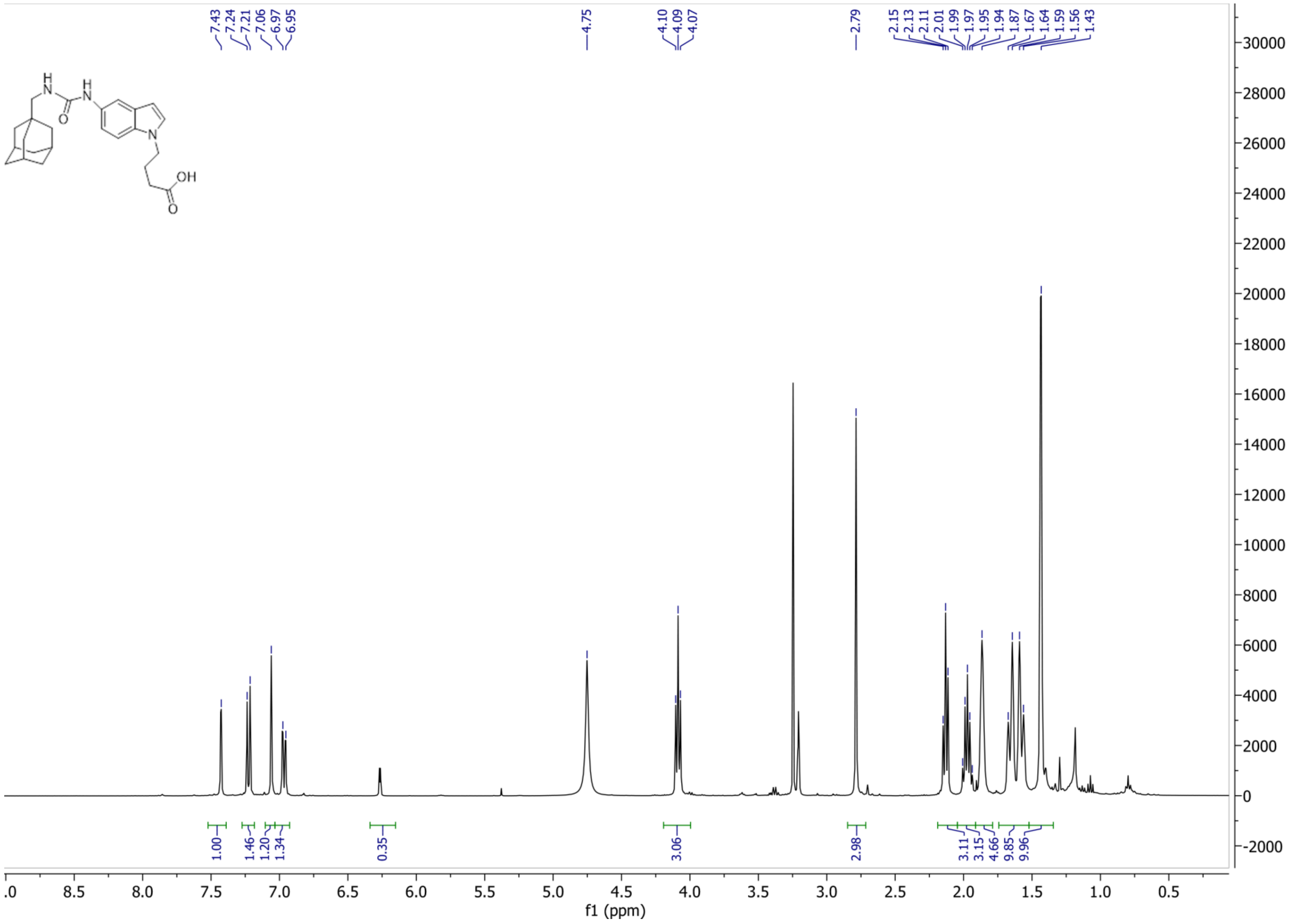

Figure S49: 1H NMR spectra of compound **38**

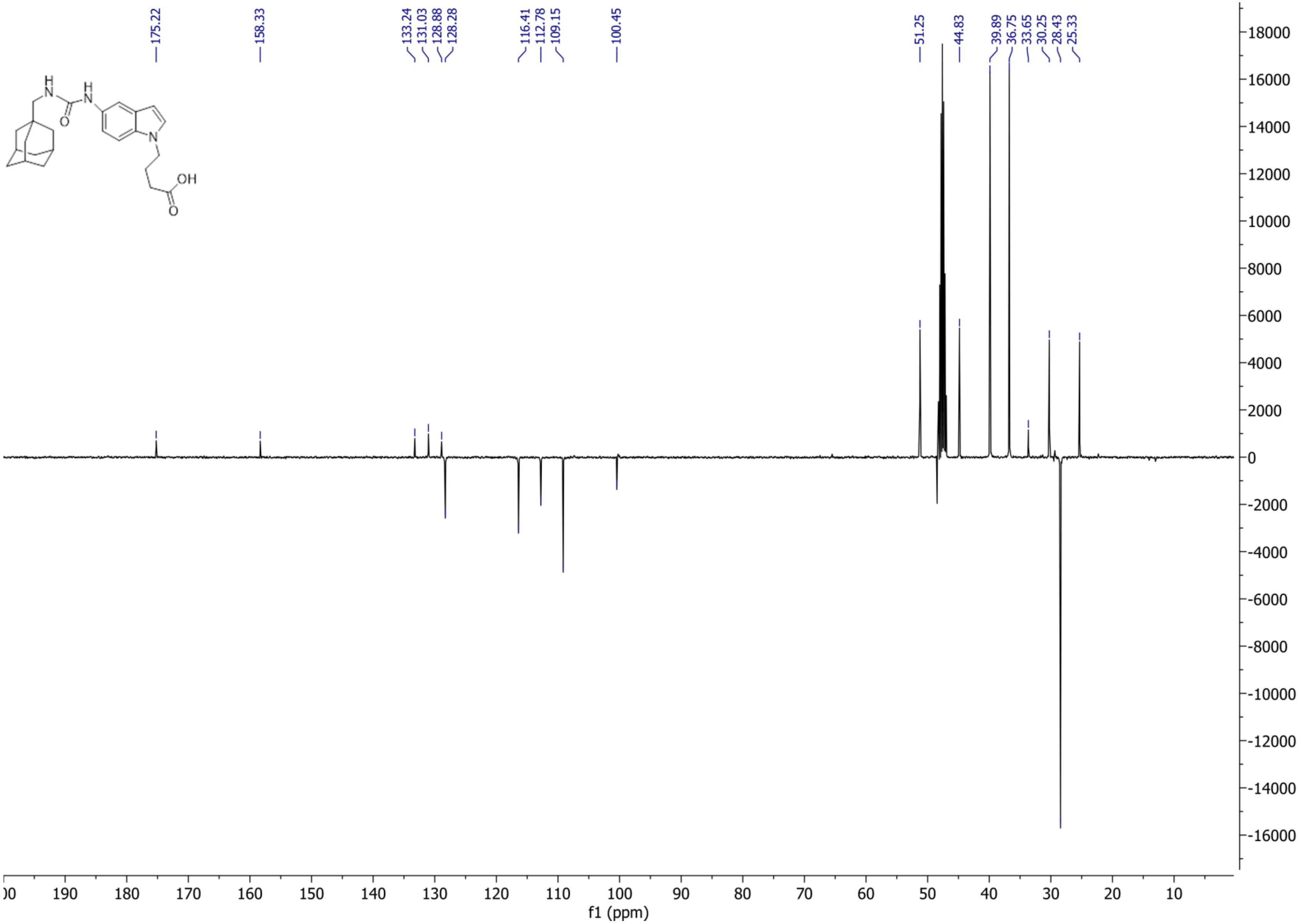

Figure S50: DEPT spectra of compound **38**

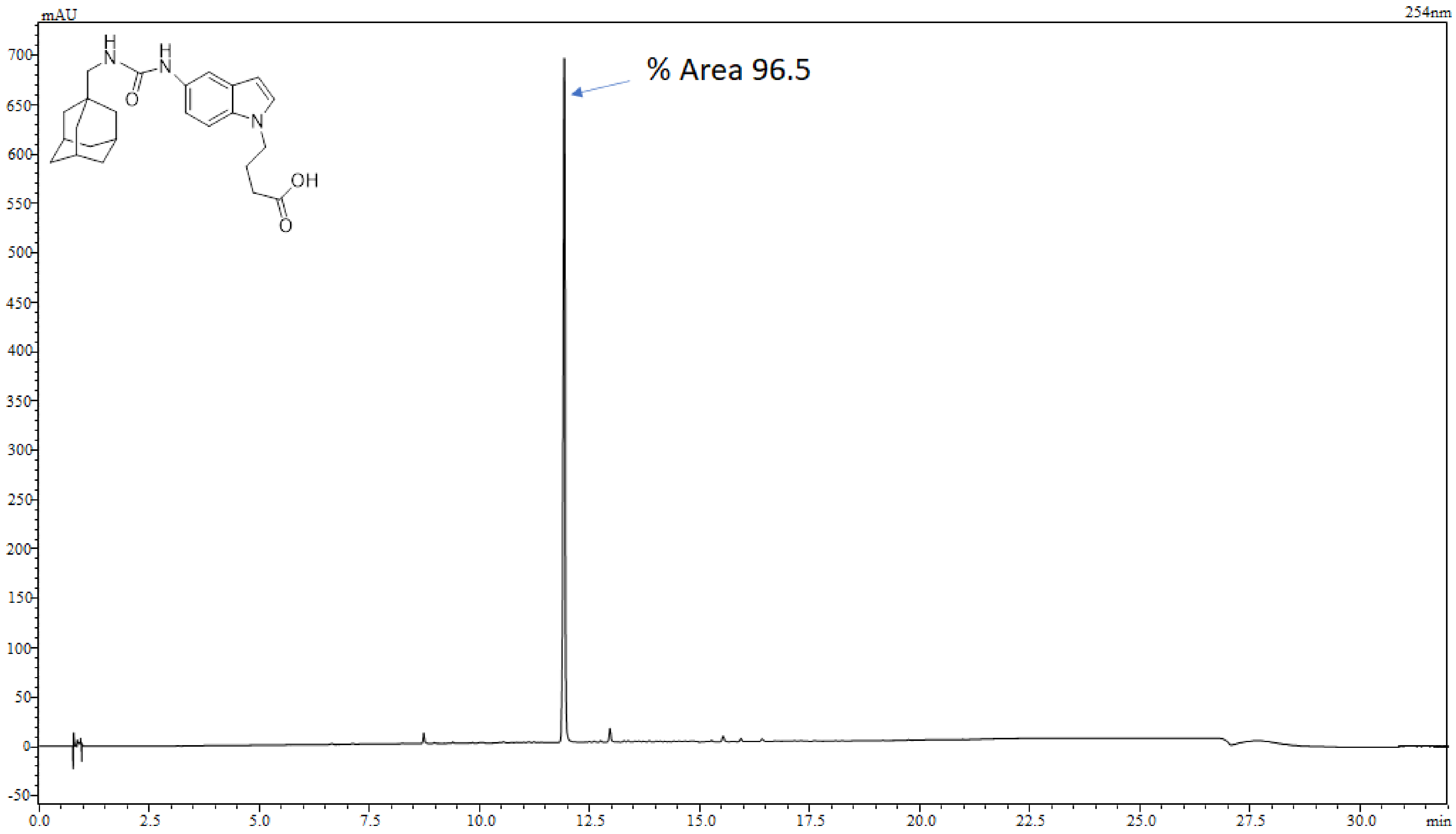

Figure S51: HPLC spectra of compound **38**

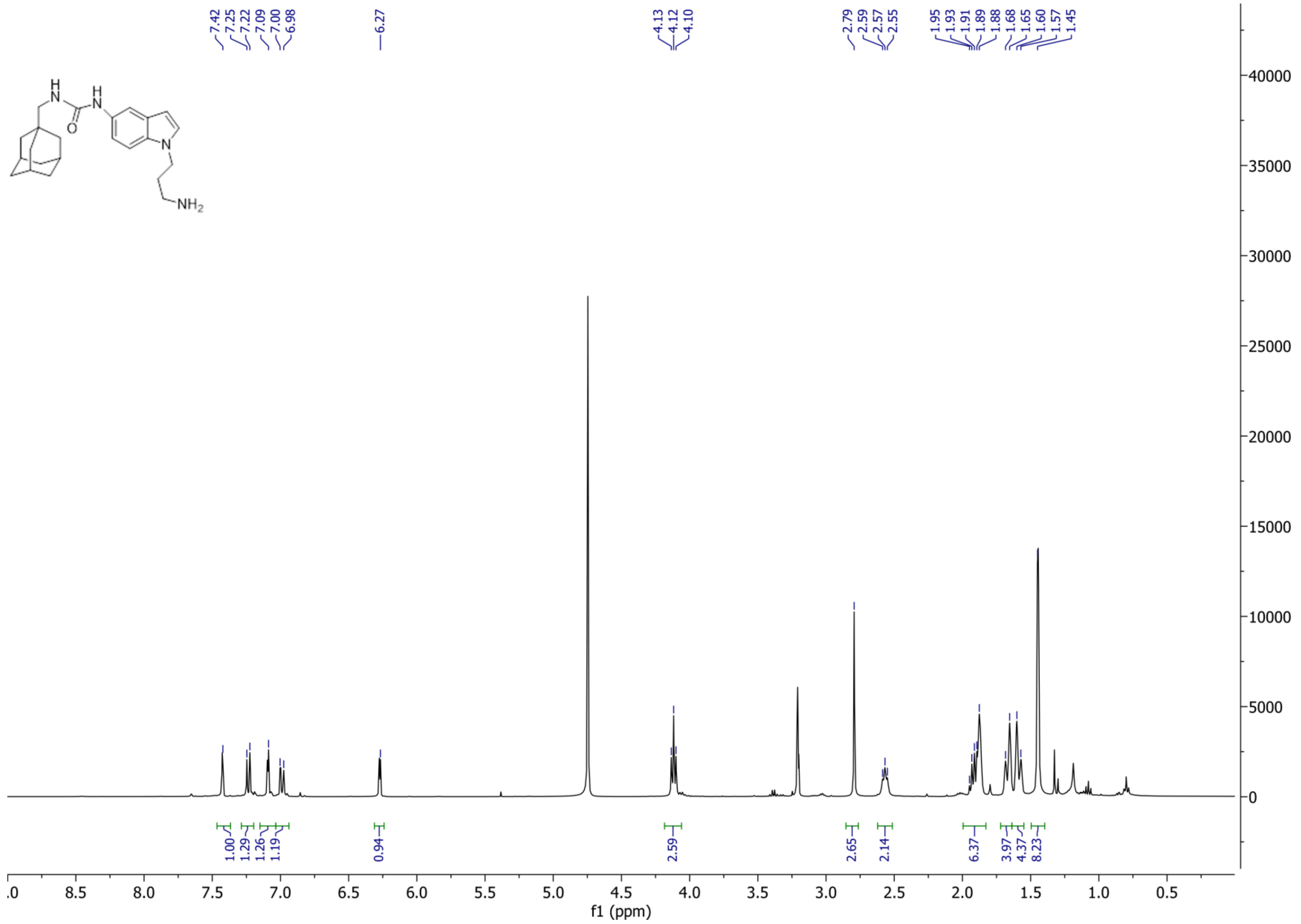

Figure S52: 1H NMR spectra of compound **39**

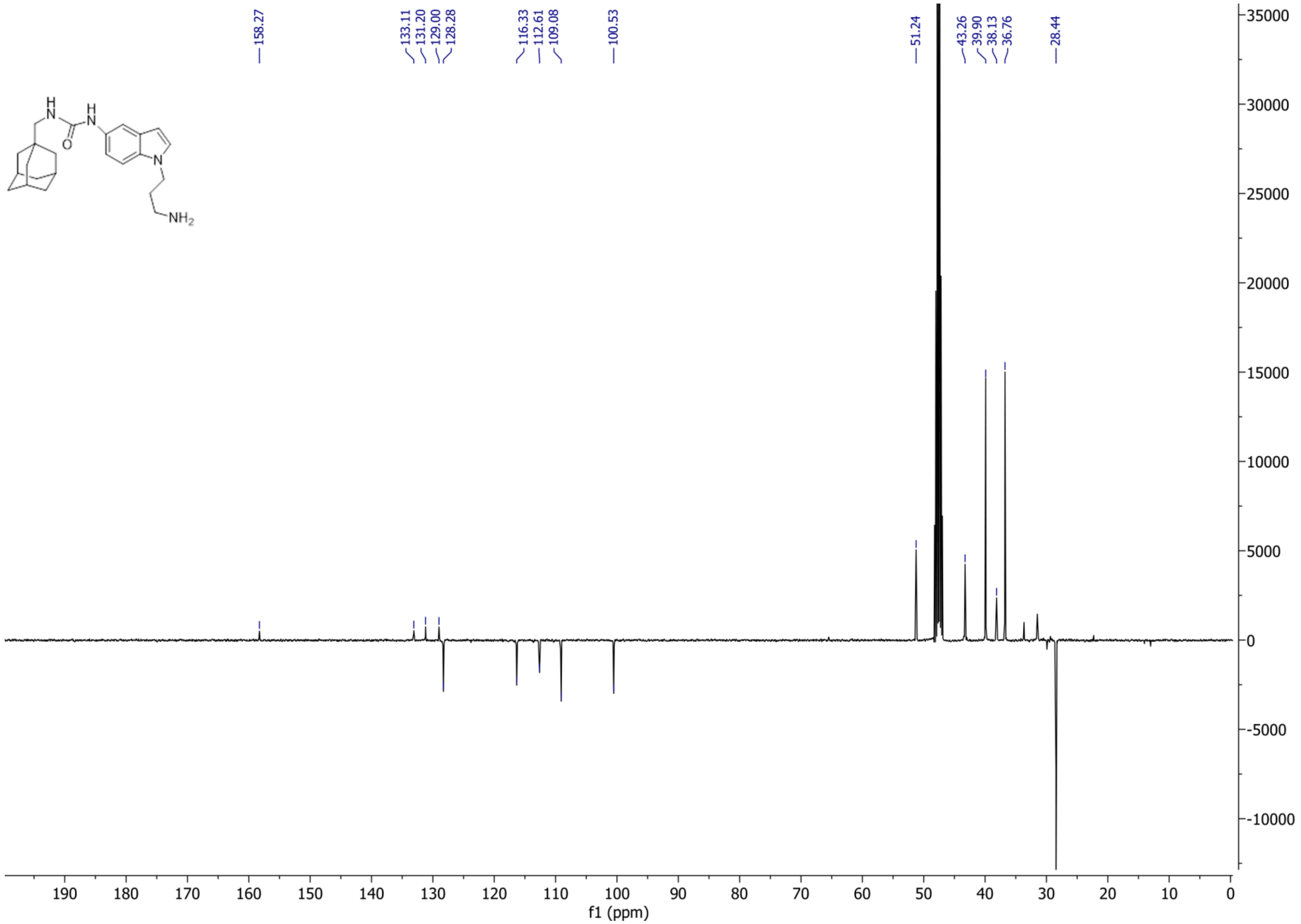

Figure S53: DEPT spectra of compound **39**

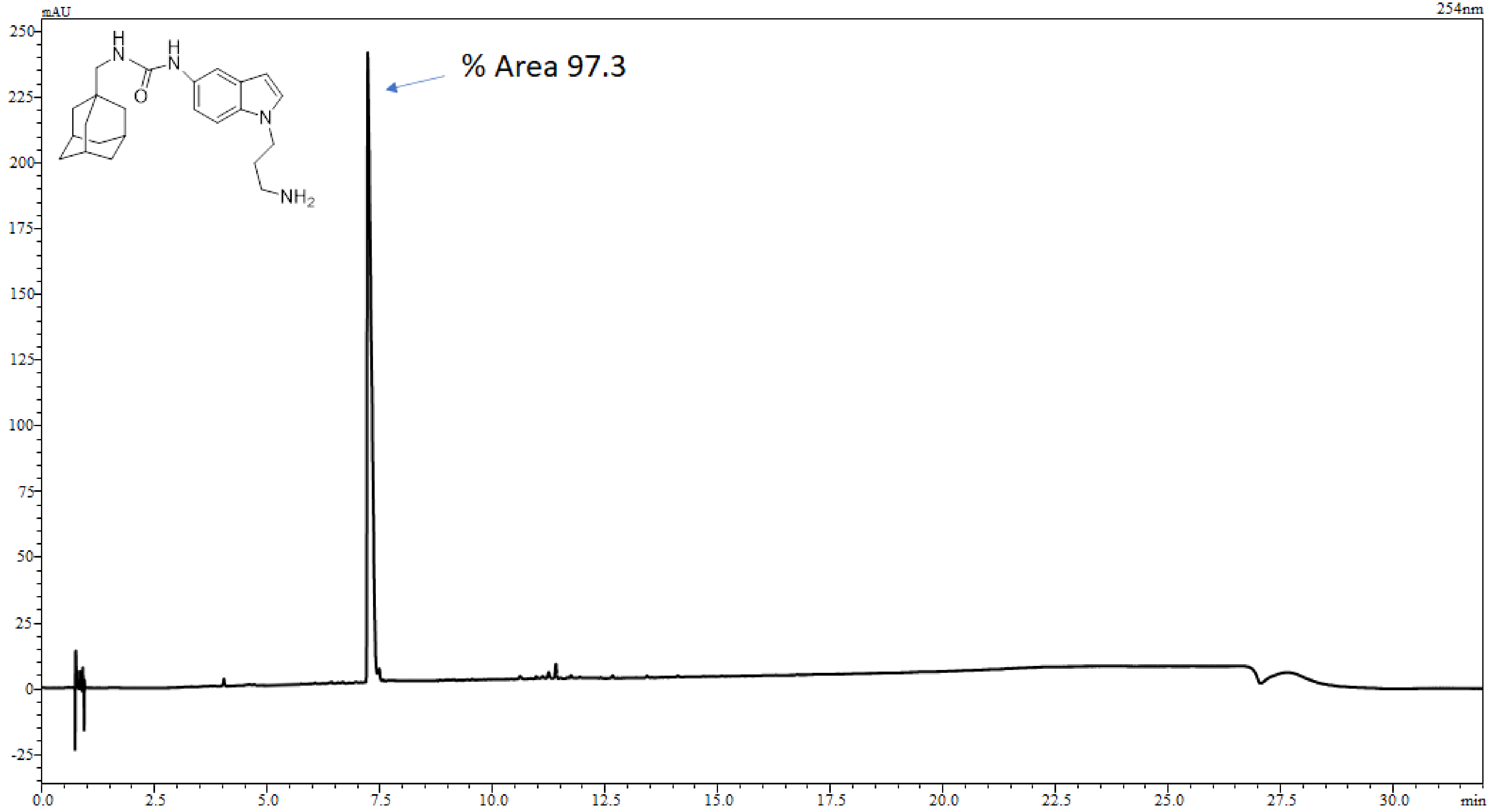

Figure S54: HPLC spectra of compound **39**

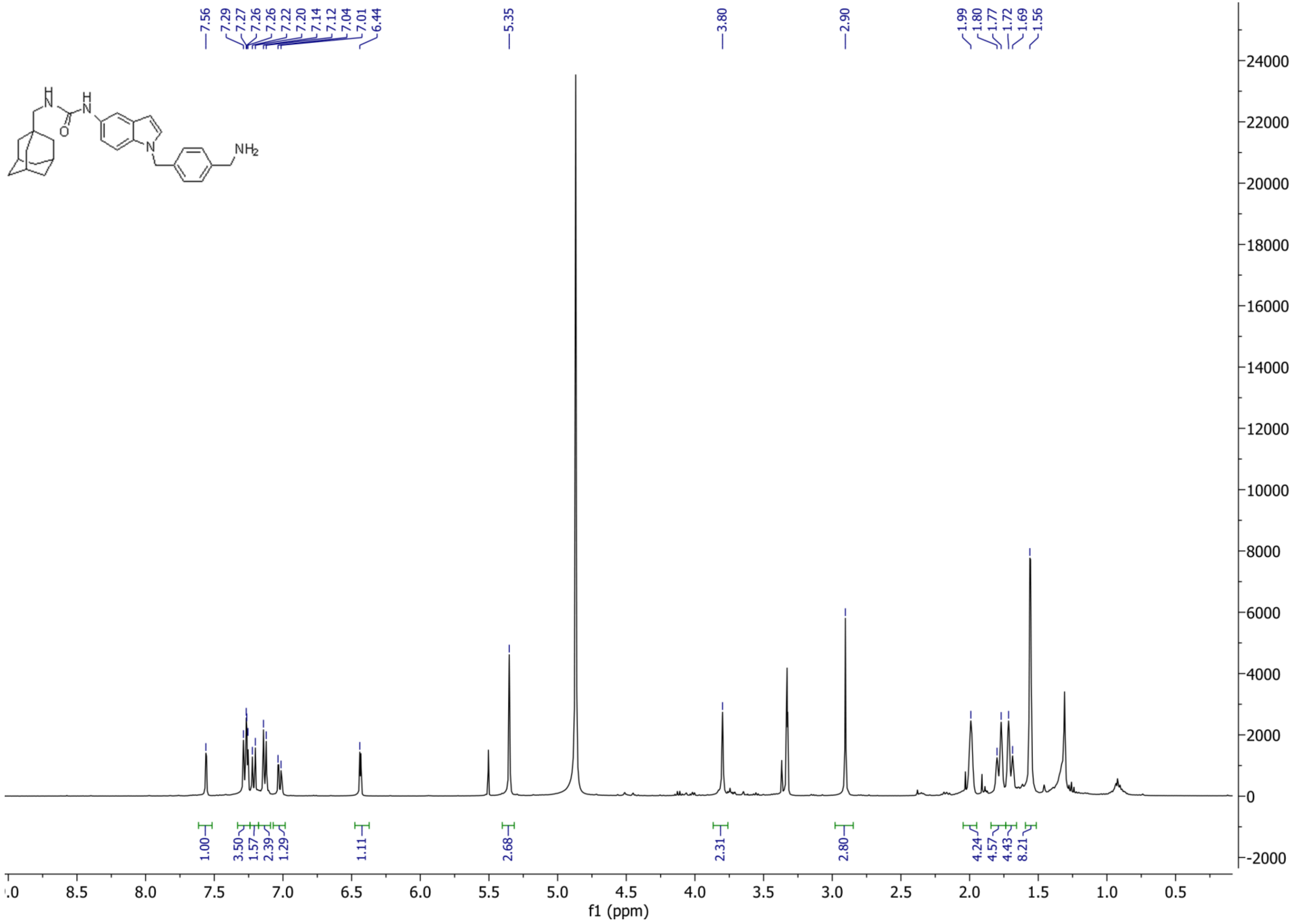

Figure S55: 1H NMR spectra of compound **42**

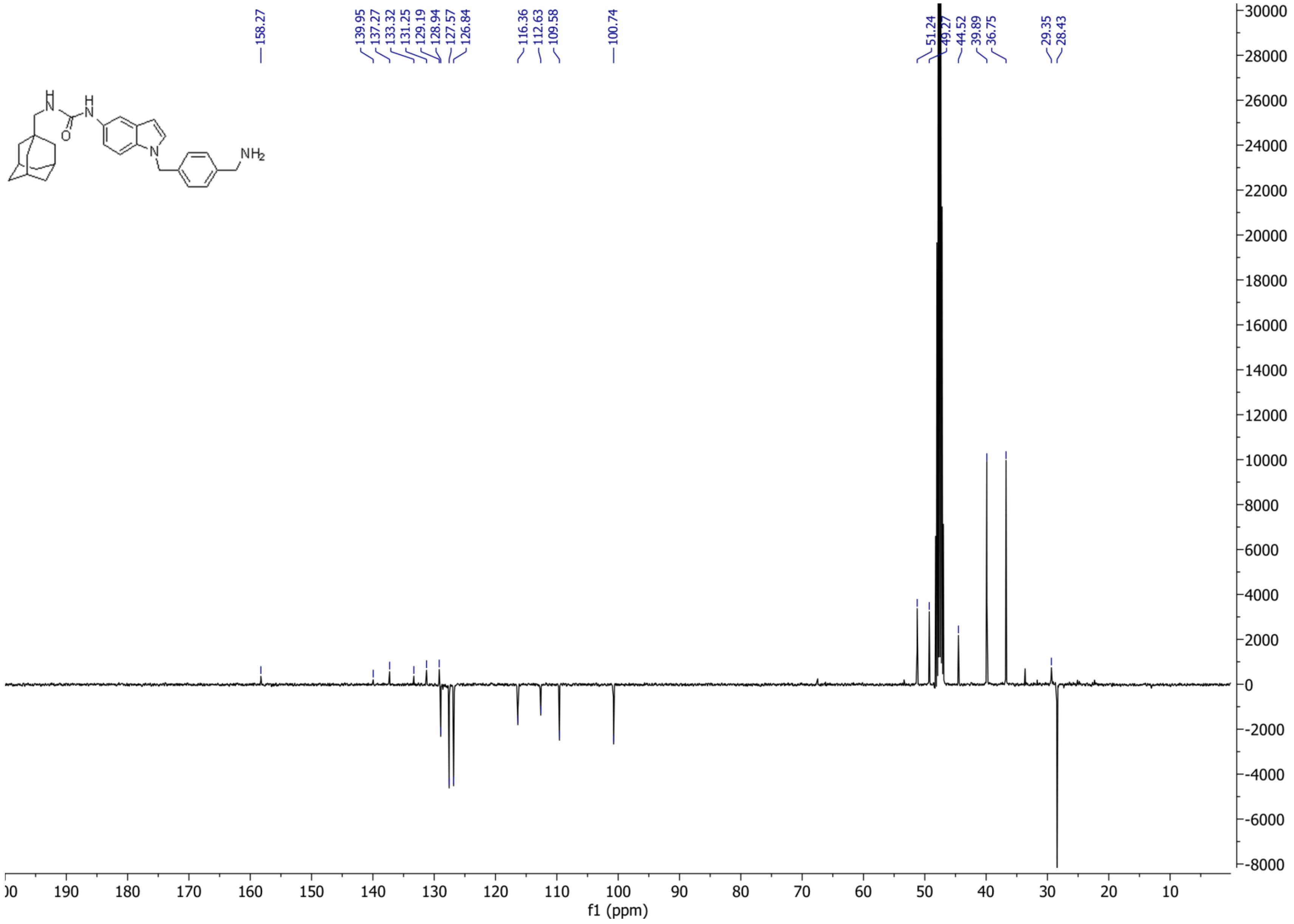

Figure S56: DEPT spectra of compound **42**

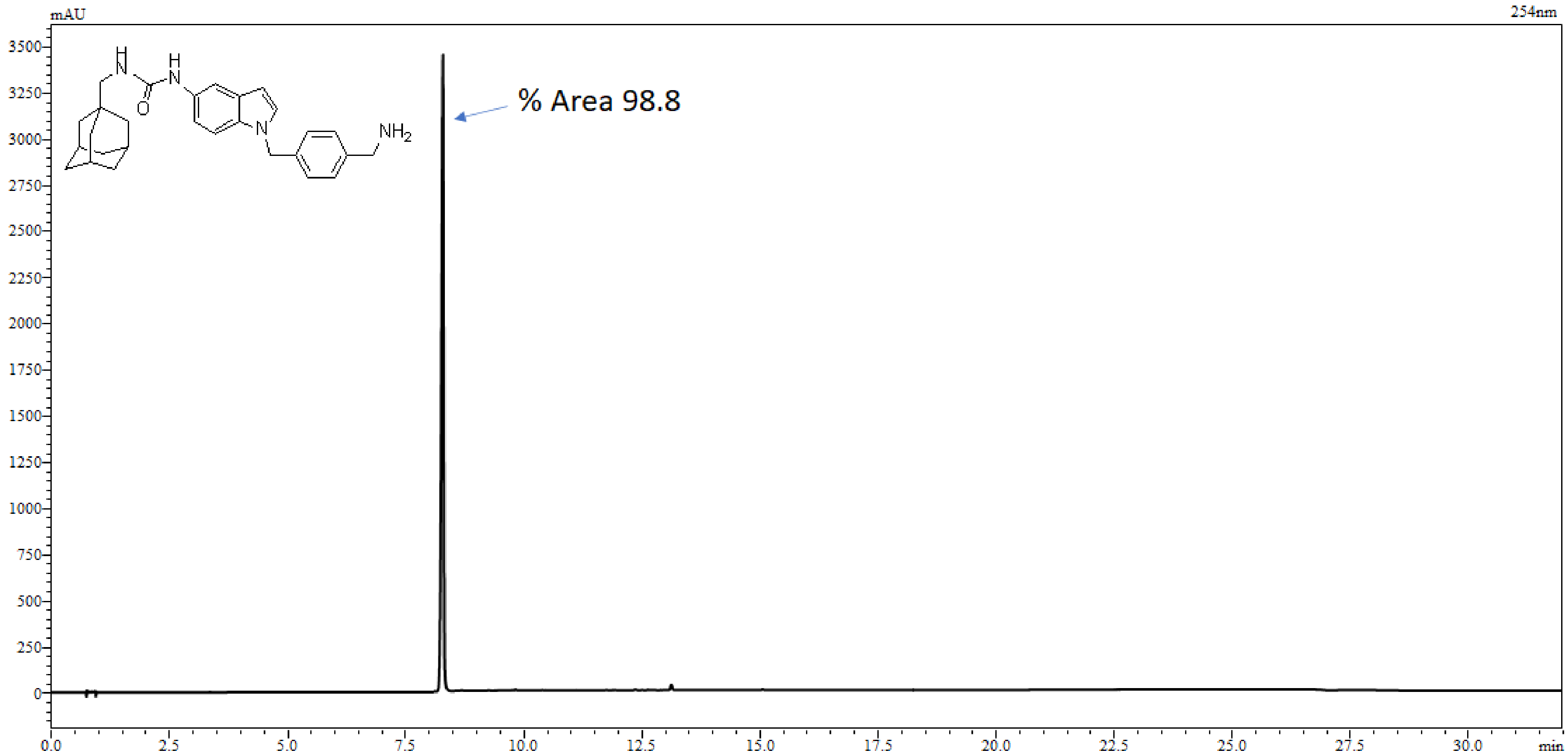

Figure S57: HPLC spectra of compound **42**

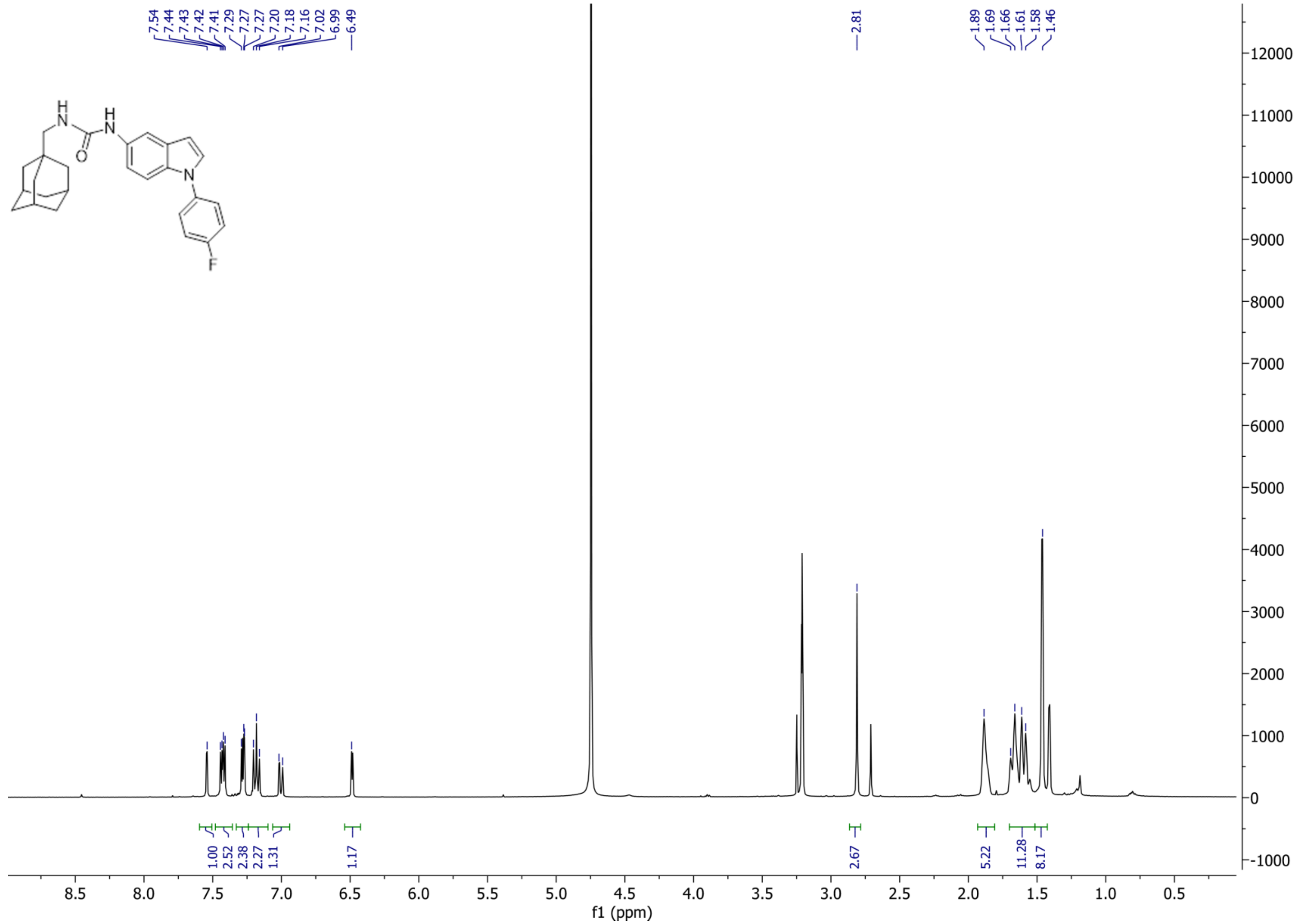

Figure S58: 1H NMR spectra of compound **45**

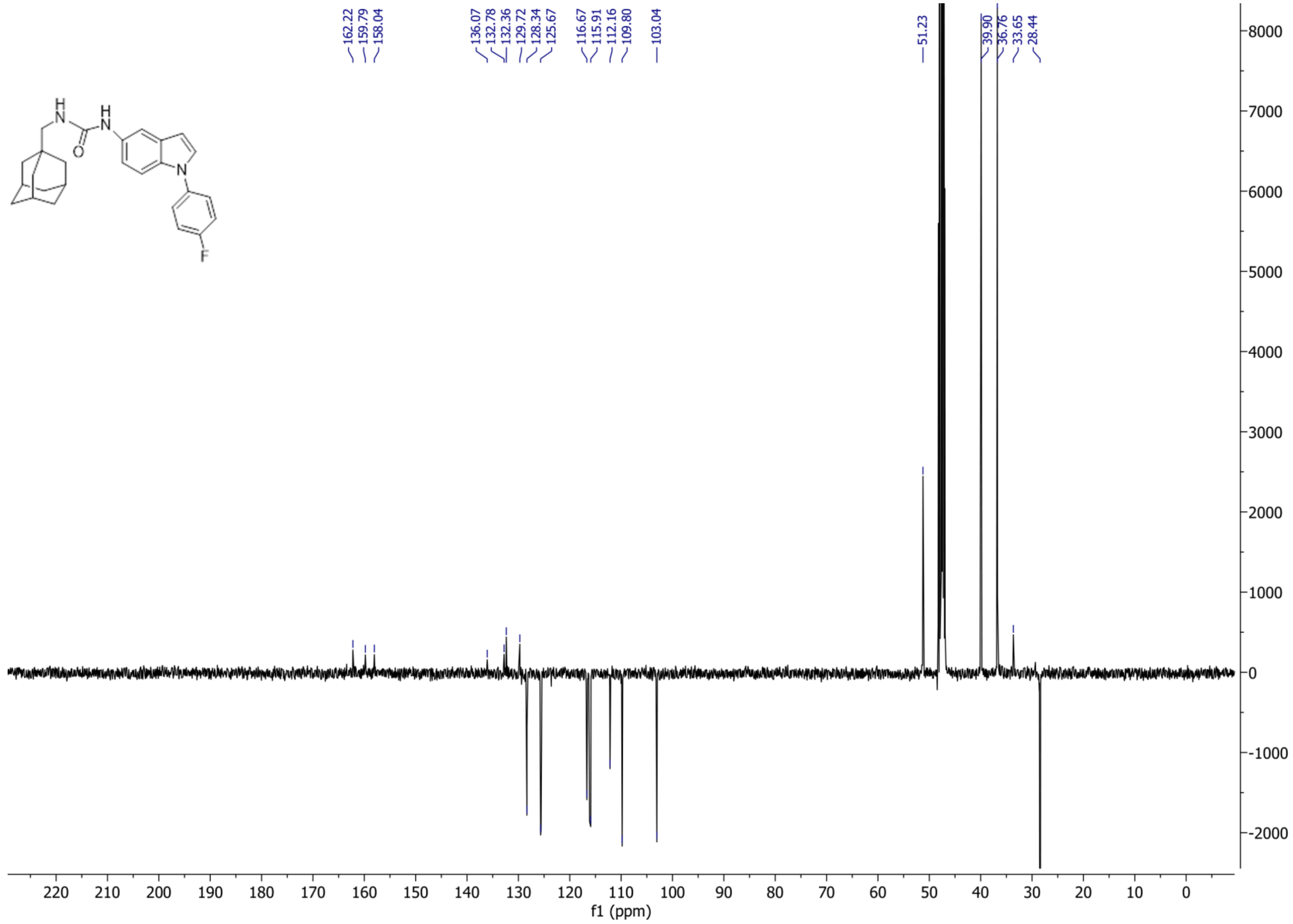

Figure S59: DEPT spectra of compound **45**

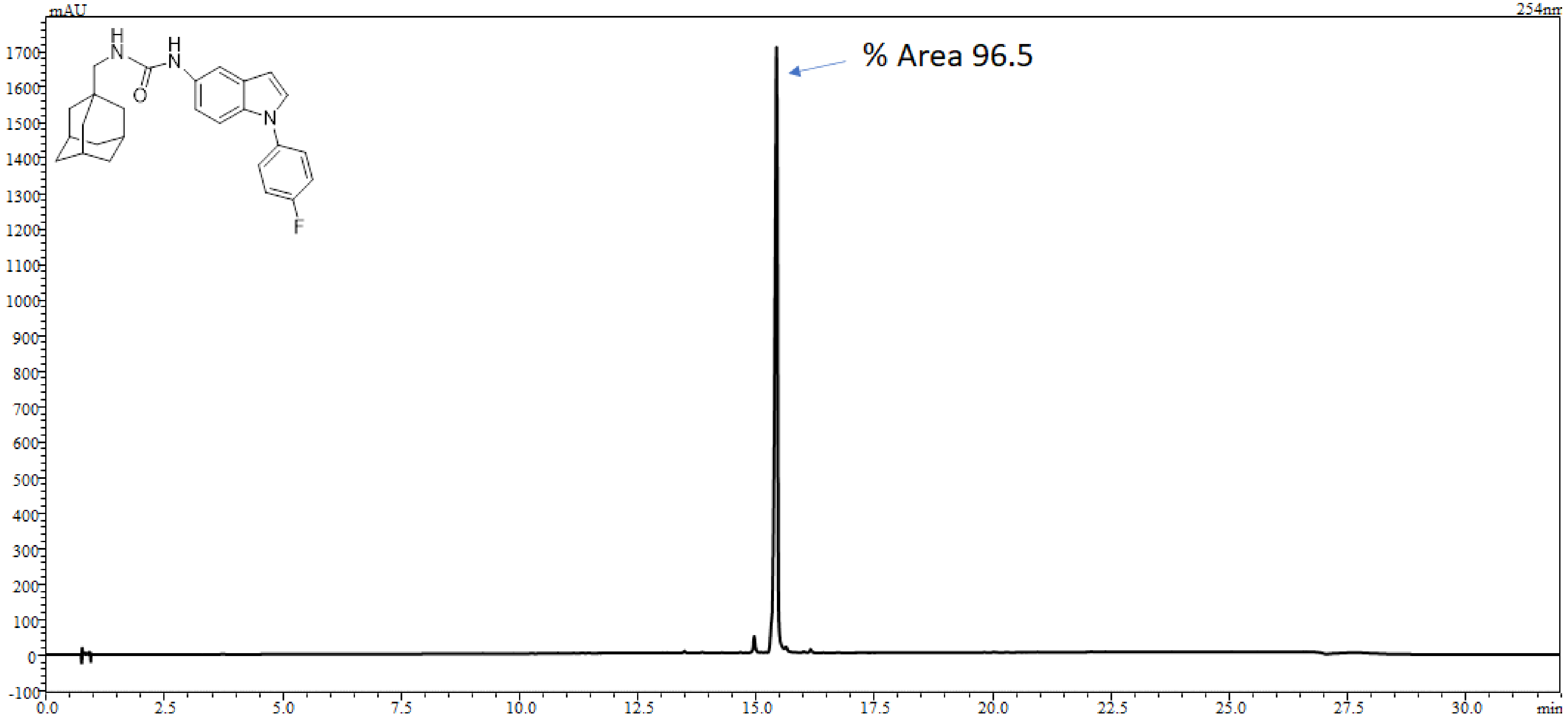

Figure S60: HPLC spectra of compound **45**

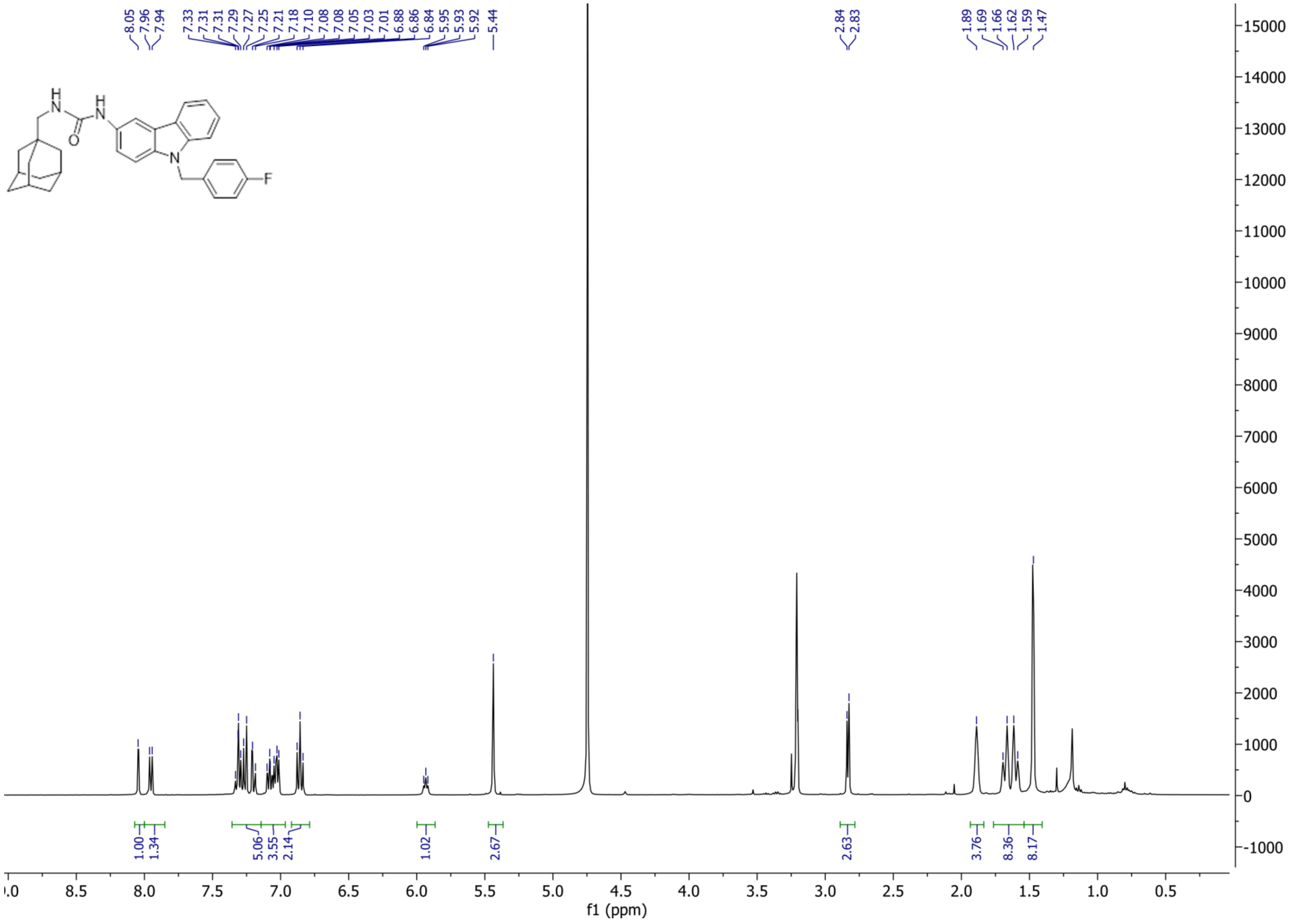

Figure S61: 1H NMR spectra of compound **48**

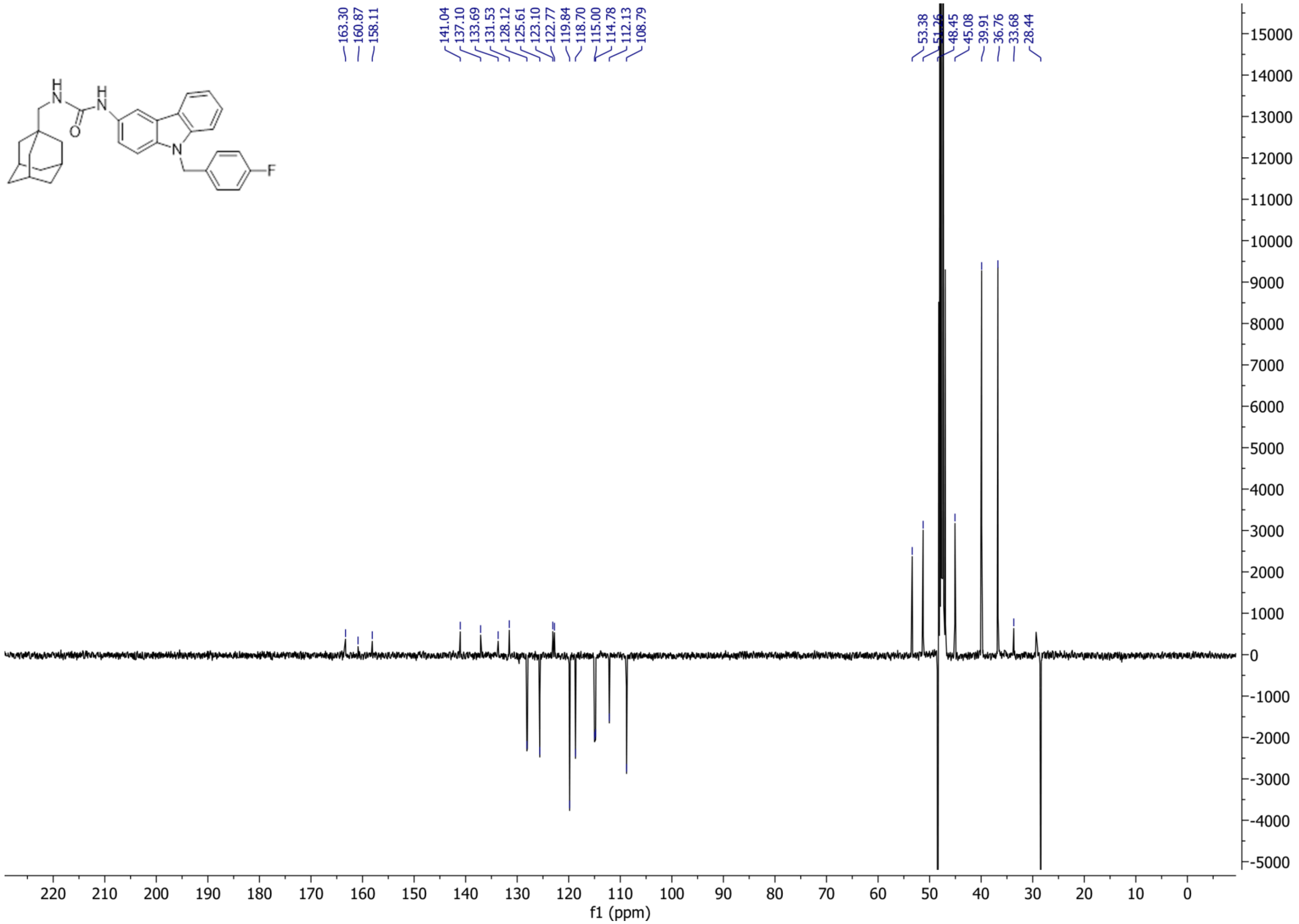

Figure S62: DEPT spectra of compound **48**

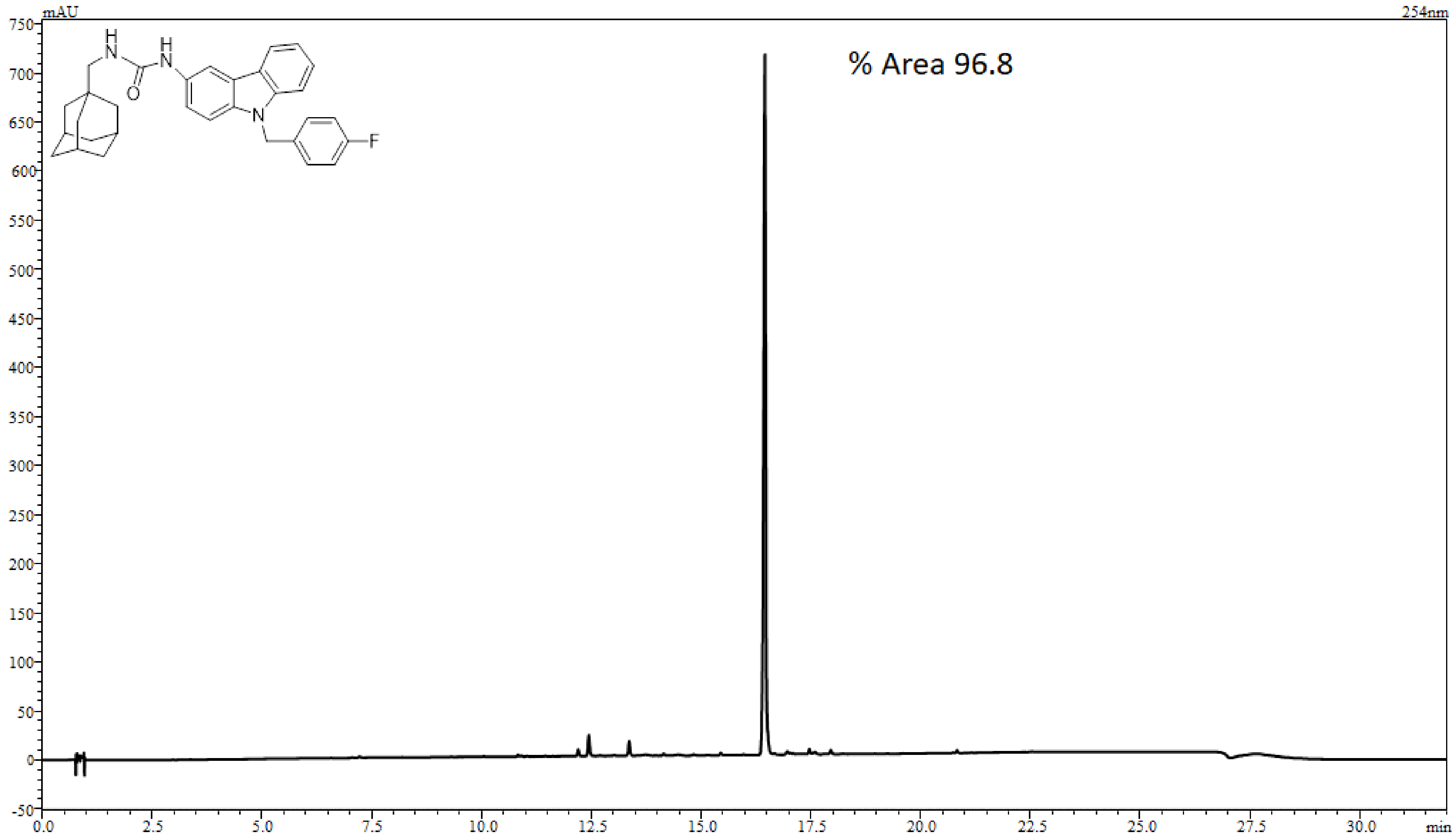

Figure S63: HPLC spectra of compound **48**

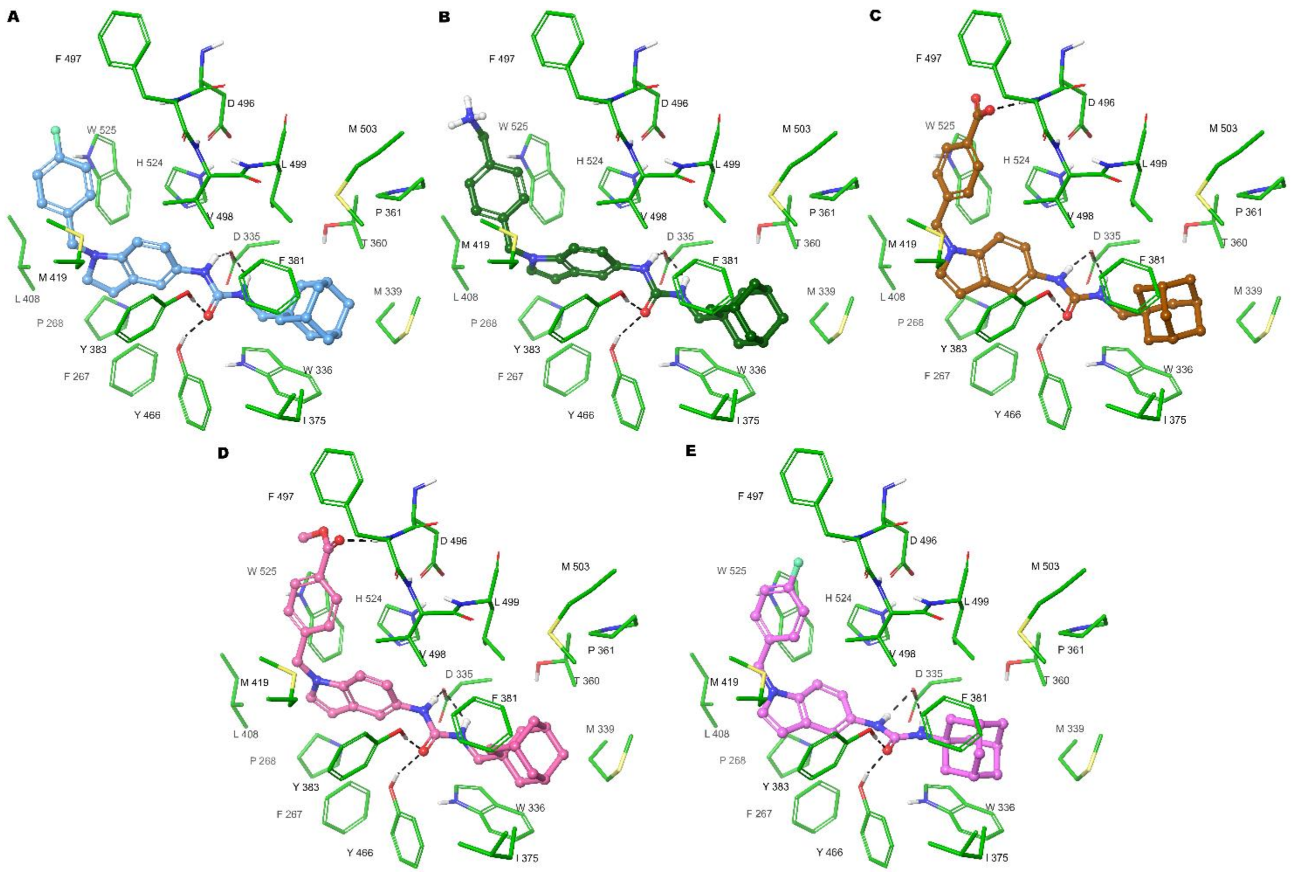

Figure S64. Three-dimensional model of the interactions given by **5** (A), **42** (B), **37** (C), **29** (D) and **33** (E) with sEH. The protein is depicted by tube (colored: C, green; polar H, white; N, dark blue; O, red). The small molecules are represented by sticks (faded azure for **5**, forest green for **42**, brown for **37**, faded salmon for **29**, faded magenta for **33**) and balls (colored: C, as for the sticks; polar H, white; N, dark blue; O, red; S, yellow). The dashed black lines indicate the hydrogen bonds between the ligand and protein.

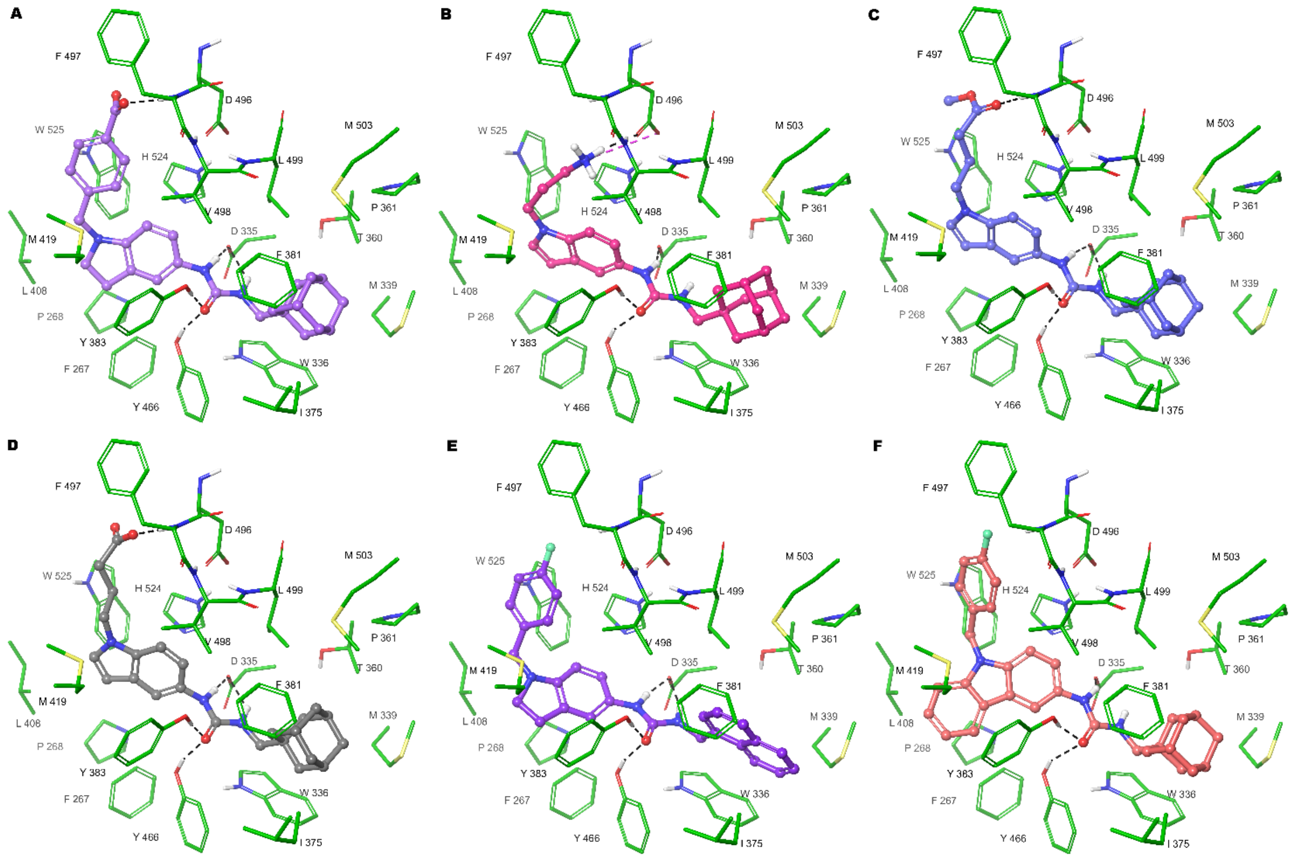

Figure S65. Three-dimensional model of the interactions given by **14** (A), **39** (B), **32** (C), **38** (D), **7** (E) and **48** (F) with sEH. The protein is depicted by tube (colored: C, green; polar H, white; N, dark blue; O, red). The small molecules are represented by sticks (faded violet for **14**, salmon-pink for **39**, grape for **32**, grey for **38**, violet for **7**; faded red for **48**) and balls (colored: C, as for the sticks; polar H, white; N, dark blue; O, red; S, yellow). The dashed black lines indicate the hydrogen bonds between the ligand and protein.

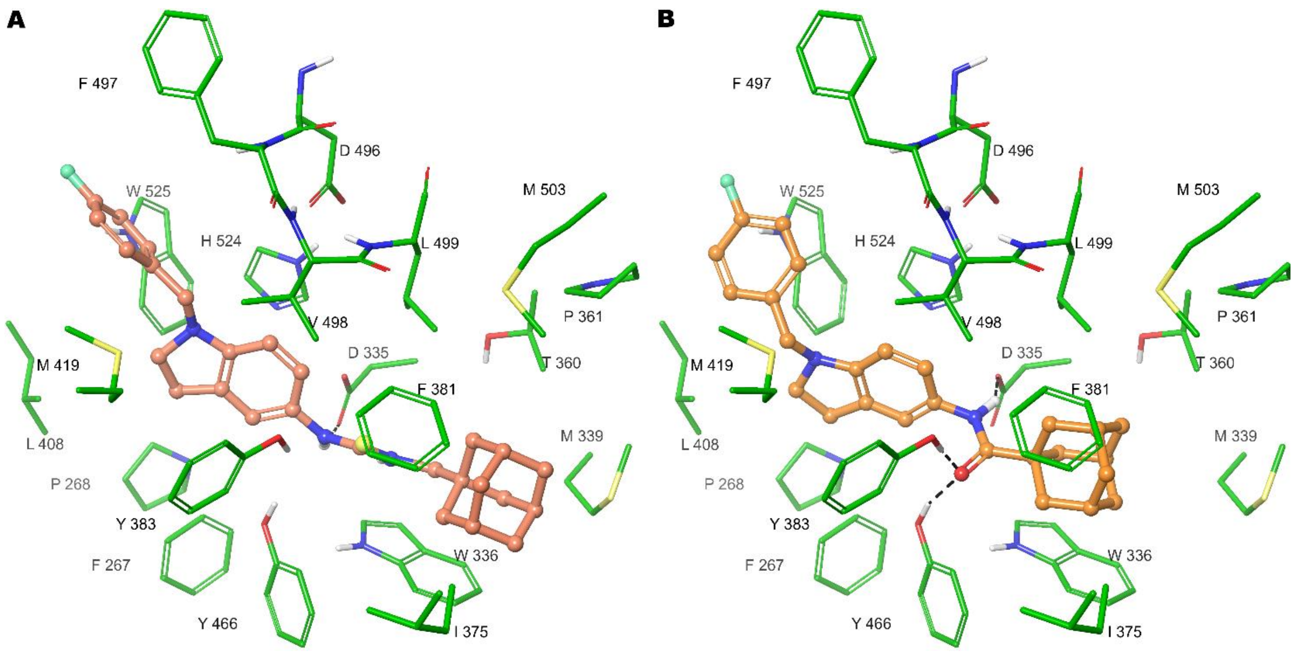

Figure S66. Three-dimensional model of the interactions given by **10** (A) and **3** (B), with sEH. The protein is depicted by tube (colored: C, green; polar H, white; N, dark blue; O, red). The small molecules are represented by sticks (faded red-orange for **10**, orange for **3**) and balls (colored: C, as for the sticks; polar H, white; N, dark blue; O, red; S, yellow). The dashed black lines indicate the hydrogen bonds between the ligand and protein.

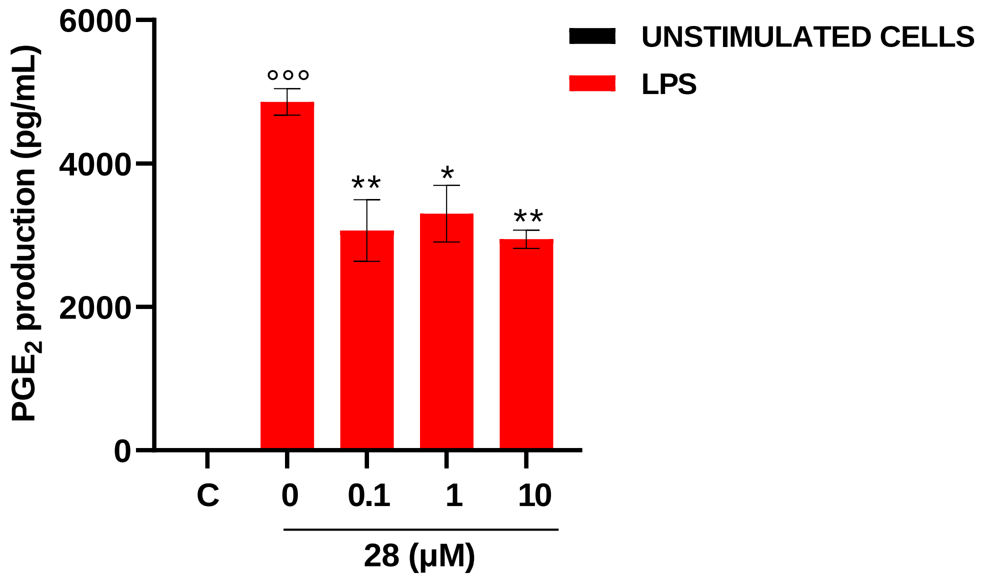

Figure S67: Effect of 28 on PG production in LPS-stimulated murine macrophages. J774 cells were pre-treated for 2 h with compound 28 (0-10 µM) and then stimulated for 24 h with LPS (10 µg/mL) to induce COX-2. The supernatants were collected for the measurement of PGE_2 levels by ELISA assay. Values represent means ± S.E.M.; n = 3 experiments. Data were analyzed by one-way ANOVA plus Bonferroni. Statistical significance is reported as follows °°° $P < 0.001$ vs. unstimulated cells, and * $p < 0.05$ and ** $P < 0.01$ vs. LPS alone.

Table S1. Observed metabolites of **28** compound in mouse liver microsomes.

Compound	Rt (min)	$[M-H]^+$	Fragment ions	Error (ppm)	Molecular Formula	Trasformation
28	11.05	456.3008	291.1476; 265.1693; 263.1534; 166.1589; 149.1323; 133.1011; 105.0701; 93.0702	-1.02	$C_{30}H_{37}ON_3$	Parent Compound
M1	9.44	472.2958	291.1478; 265.1695; 182.1539; 165.1273; 147.1166; 133.1011; 105.0701; 91.0546	-0.75	$C_{30}H_{37}O_2N_3$	Oxidation
M2	9.53	472.2958	265.1696; 182.1540; 165.1274; 147.1167; 133.1012; 105.0701; 91.0546	0.03	$C_{30}H_{37}O_2N_3$	Oxidation
M3	10.36	472.2960	281.1646; 166.1590; 149.1323; 133.1011; 93.0702	-1.84	$C_{30}H_{37}O_2N_3$	Oxidation
M4	8.36	488.2909	265.1695; 198.1487; 181.1220; 163.1115; 145.1013; 133.1012; 105.0700; 93.0702	-0.24	$C_{30}H_{37}O_3N_3$	Oxidation, Oxidation
M5	8.80	488.2911	470.2816; 265.1695; 198.1488; 181.1224; 163.1116; 145.1011; 133.1012; 105.0702; 93.0702	-0.56	$C_{30}H_{37}O_3N_3$	Oxidation, Oxidation
M6	8.98	488.2907	470.2822; 265.1692; 198.1491; 181.1221; 163.1116; 145.1011; 133.1012; 105.0701; 93.0703	-0.18	$C_{30}H_{37}O_3N_3$	Oxidation, Oxidation

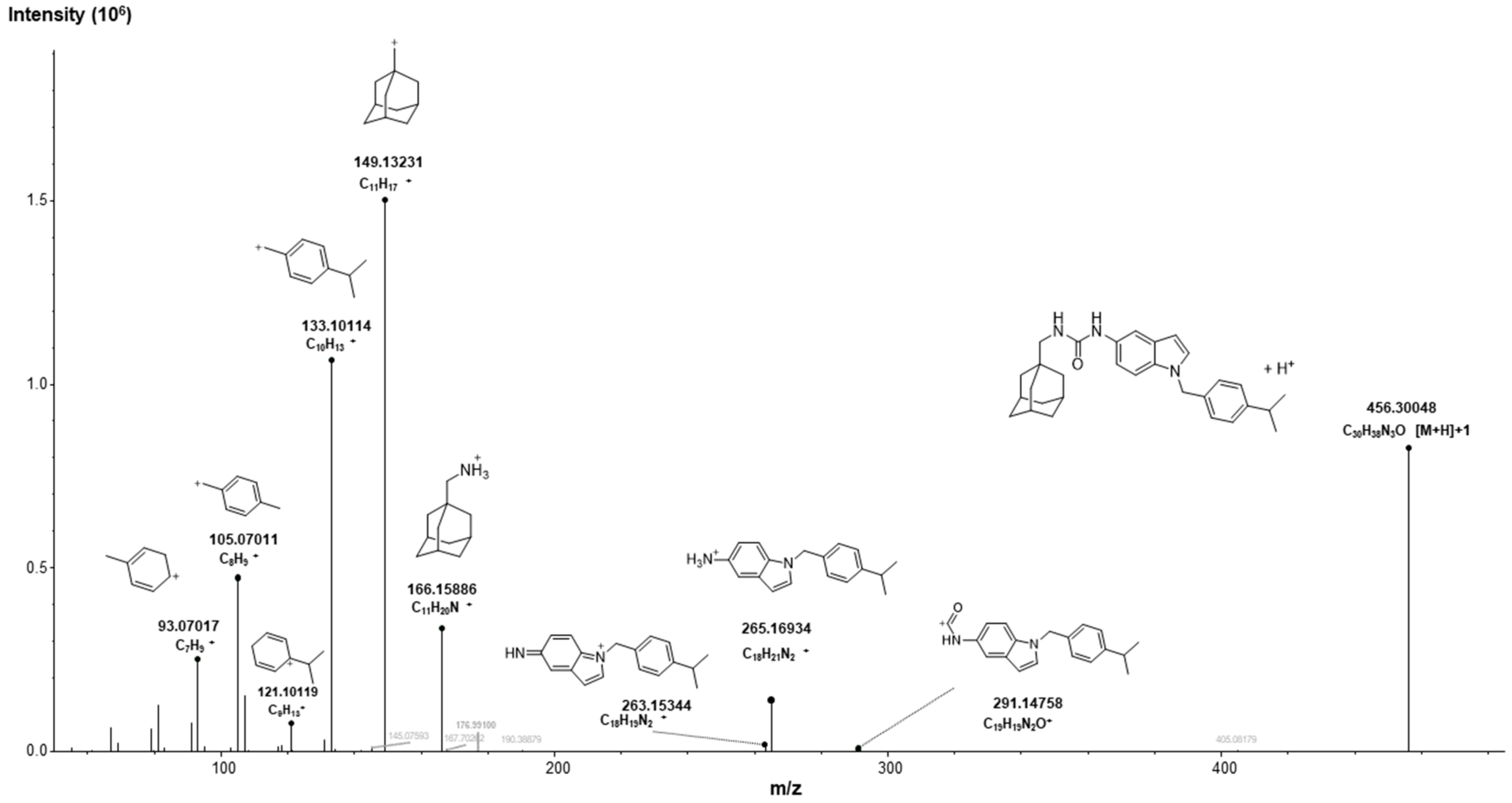

Figure S68. MS2 spectrum with the fragmentation pathways of the parent compound.

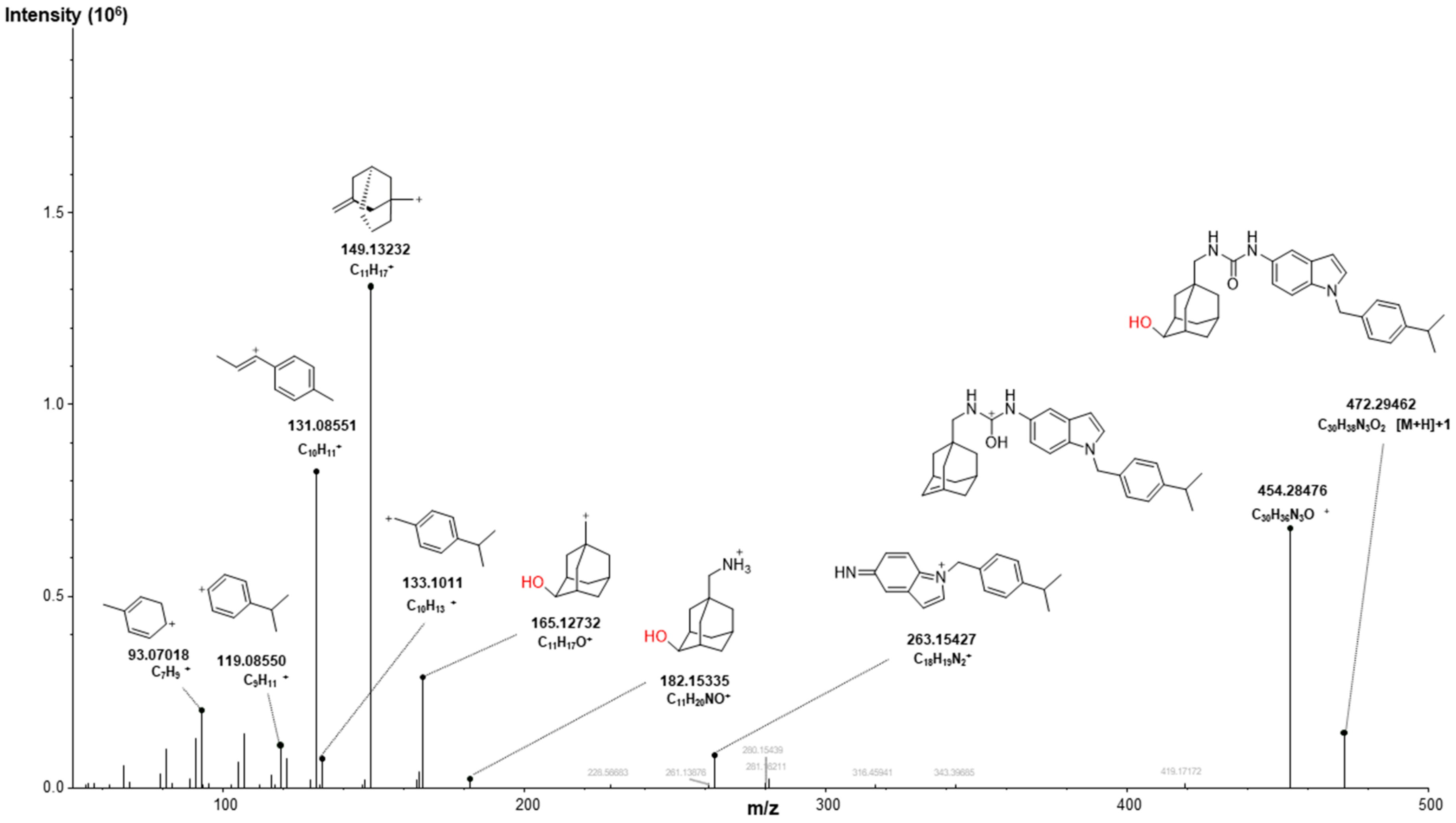

Figure S69. MS2 spectrum with the fragmentation pathways of **M2** metabolite.

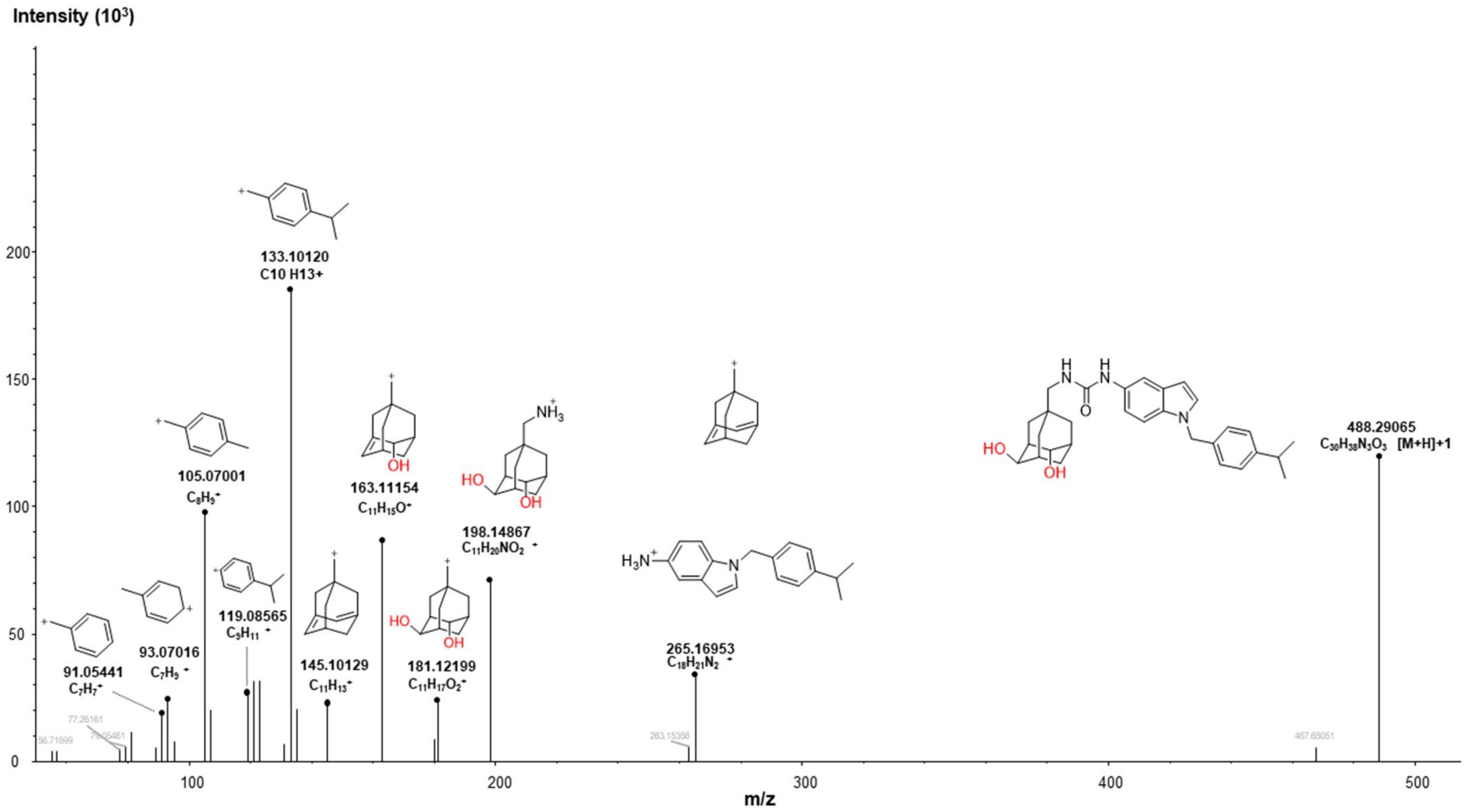

Figure S70. MS2 spectrum with the fragmentation pathways of **M4** metabolite.

Table S2. Optimal LC-MS/MS parameters for compound **28** quantification.

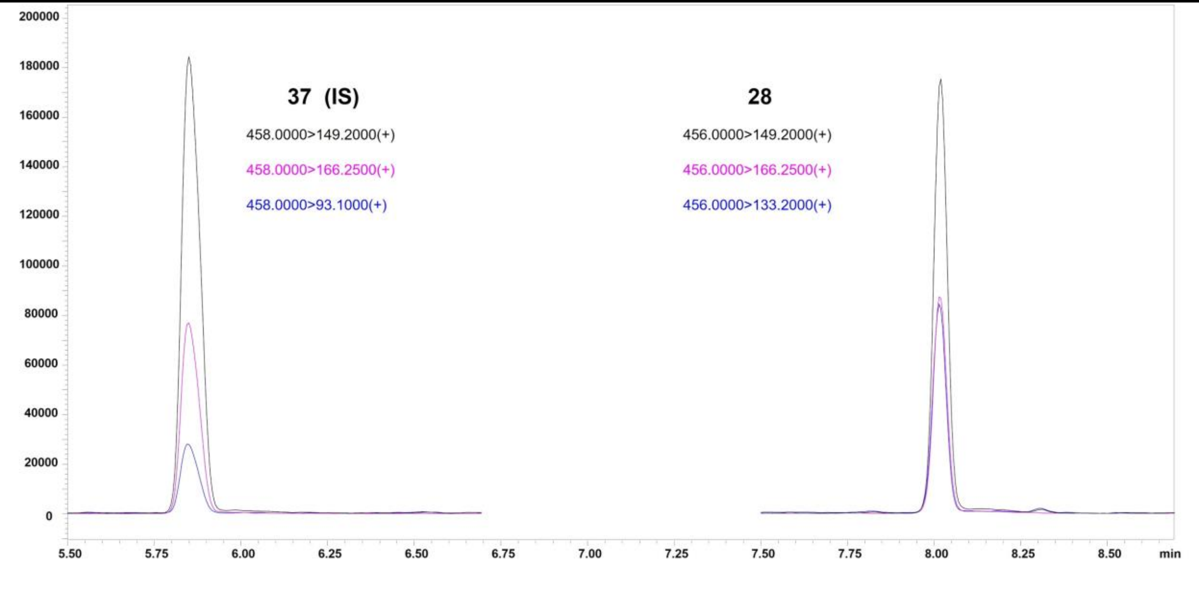

Analyte	*m/z* (MS1)	*m/z* (MS2)	Q1 Pre bias (V)	Collision energy (V)	Q3 Pre Bias (V)
**28**	456.0	149.2	-24.0	-31.0	-25.0
		166.2	-24.0	-23.0	-16.0
		133.2	-24.0	-39.0	-22.0
**SI 37**	458.0	149.2	-25.0	-32.0	-26.0
		166.2	-14.0	-24.0	-16.0
		93.1	-14.0	-46.0	-15.0

Table S3: Optimized MRM parameters for the quantification of lipid mediators (*= qualifier ion)

Name	Precursor (m/z)	Product (m/z)	Dwell Time (msec)	Q1 Pre Bias (V)	CE	Q3 Pre Bias (V)
**14,15-DHET**	337.0	207.3	10	14	19	13
**11,12-DHET-d11**	348.2	167.3	10	11	20	10
**8,9-DHET**	337.0	127.2	10	14	23	12
	337.0	185.2*	10	18	19	11
**11,12-DHET**	337.0	167.2	10	14	21	10
**20-HETE**	319.2	289.2	10	17	18	13
	319.2	245.4*	10	17	16	16
**5,6-DHET**	337.1	145.2	10	14	18	14
**12-HEPE**	317.2	179.3	10	10	14	11
	317.2	208.3*	10	13	14	21
**15-HEPE**	317.2	219.3	10	17	13	10
	317.2	247.2*	10	10	15	16
**5-HEPE**	317.2	115.2	10	10	14	11
**15-HETE**	319.1	219.3	10	10	14	14
	319.1	175.3*	10	17	15	11
**15-HETE-d8**	327.3	226.3	10	13	14	10
**12-HETE**	319.1	179.2	10	10	28	15
	319.1	208.2*	10	17	13	15
	319.1	135.3*	10	10	14	17
**5-HETE**	319.1	115.2	10	17	14	11
**5-HETE-d8**	327.3	309.3	10	13	13	14
	327.3	116.2*	10	13	16	11
**14,15-EET-d11**	330.2	175.4	10	18	15	18
**5,6-EET**	319.1	191.3	10	13	13	19
**8,9-EET**	319.0	155.3	10	13	13	15
	319.0	127.3*	10	13	16	12
**11,12-EET**	319.0	167.3	10	13	15	16
	319.0	208.2*	10	13	13	13
**14,15-EET**	319.0	219.3	10	13	12	22
	319.0	175.4*	10	17	15	17

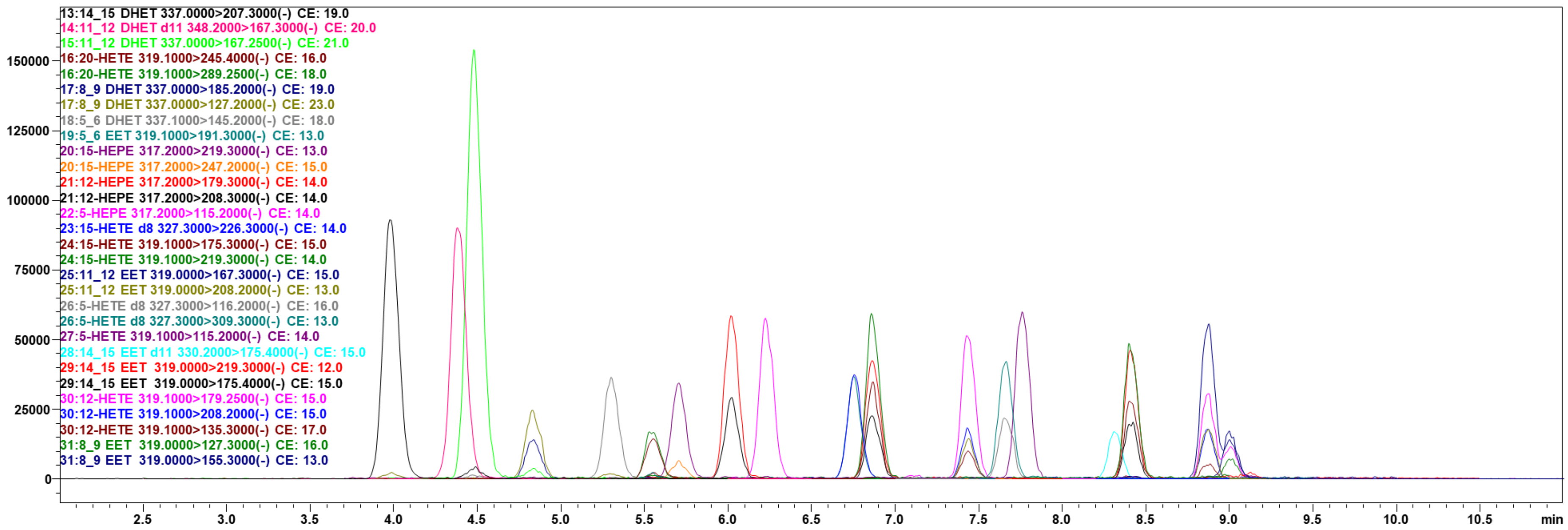

Figure S71: Representative MRM traces of monitored eicosanoids (table S3)
